# Photoelectrochemical Iron(III) Catalysis for Late‐Stage C─H Fluoroalkylations

**DOI:** 10.1002/anie.202504143

**Published:** 2025-05-07

**Authors:** Vladimir Motornov, Sven Trienes, Simonetta Resta, João C. A. Oliveira, Zhipeng Lin, Zhi Liu, Tristan von Münchow, Claudia Stückl, Lutz Ackermann

**Affiliations:** ^1^ Wöhler Research Institute for Sustainable Chemistry (WISCh) Georg‐August‐Universität Tammannstraße 2 37077 Göttingen Germany; ^2^ German Center for Cardiovascular Research (DZHK) Potsdamer Strasse 58 10785 Berlin Germany; ^3^ Institut für Anorganische Chemie Georg‐August‐Universität Tammannstraße 4 37077 Göttingen Germany

**Keywords:** C─H Functionalization, Electrophotochemistry, Fluoroalkylation, Iron catalysis, Sustainable chemistry

## Abstract

Chemo‐ and site‐selective functionalization of complex molecules poses a fundamental challenge. Herein, we introduce a resource‐economic photoelectrocatalysis strategy to enable versatile direct fluoroalkylations catalyzed by Earth‐abundant iron and paired with the hydrogen evolution reaction (HER). Notably, the devised approach proved amenable to versatile late‐stage C─H fluoroalkylations of bio‐relevant heterocycles, such as xanthines, nucleobases, and nucleosides. Mechanistic studies supported a ligand‐to‐metal charge transfer‐induced formation of the fluoroalkyl radical.

## Graphical abstract

Efficient electrophotochemical C─H fluoroalkylation of complex bioactive substrates with abundant fluoroalkyl carboxylic acids employing an inexpensive iron(III) catalyst accompanied by hydrogen evolution reaction has been developed.

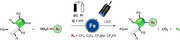

## Introduction

**Figure 1:**
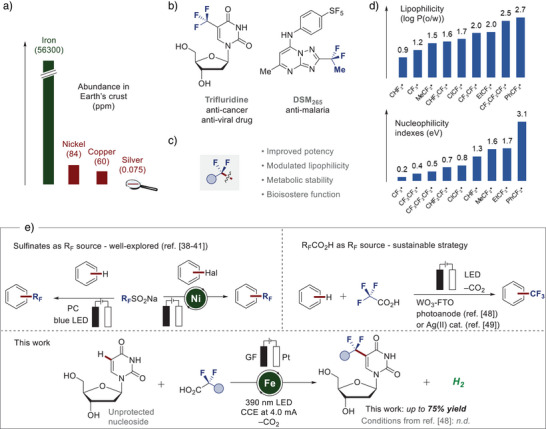
Motivation and strategy for the late‐stage C─H fluoroalkylation. a) Natural abundance of iron and other transition metals in comparison. b) Representative fluoroalkyl‐containing compounds with pharmaceutical relevance. c) Beneficial effects of fluoroalkyl‐substitution on pharmacological properties. d) Lipophilicity indexes log *P*(o/w) for selected fluoroalkyl radicals. Calculations were performed at the ωB97X‐D + SMD(Octanol or Water)/def2‐QZVPP//ωB97X‐D/def2‐TZVPP level of theory (top) and calculated relative nucleophilicity of different fluoroalkyl radicals at the ωB97X‐D + SMD(Acetonitrile)/def2‐QZVPP//ωB97X‐D/def2‐TZVPP level of theory. Values take the malononitrile radical as reference (bottom). e) Electrochemical approaches to C─H fluoroalkylation using sulfinate and carboxylic acid reagents, and this work: Late‐stage C─H fluoroalkylations of complex biomolecules and drug compounds enabled by photoelectrochemical iron(III)‐catalysis. PC = photocatalyst, R_F_ = fluoroalkyl group ‐CF_2_Alk, FTO = fluorine‐doped tin oxide glass. CCE = constant current electrolysis.

Synergetic combination of photoexcitation with electron transfer by anodic oxidation bears unique potential for novel reaction manifolds that go beyond individual photo‐ or electrochemistry.^[^ ^1^, ^2^, ^3^ ^]^ Visible light photocatalysis has surfaced as a transformative platform for molecular synthesis to achieve single‐electron transfer (SET)‐enabled reactions. Despite major advances, photocatalyzed functionalization reactions often suffer from expensive catalysts or stoichiometric amounts of chemical oxidants.^[^ ^4^, ^5^, ^6^, ^7^, ^8^, ^9^, ^10^ ^]^ In contrast, electrochemistry has been recognized as an enabling tool to promote proton and electron transfer via the hydrogen evolution reaction (HER) devoid of sacrificial oxidants.^[^ ^11^, ^12^, ^13^ ^]^ Although both strategies have proven to be enabling, the merger of photochemistry with electrocatalysis has the largely untapped potential to harness the energy of both light and electricity to reach extreme redox potentials under exceedingly mild conditions.^[^ ^14^, ^15^, ^16^ ^]^ In terms of homogeneous catalysis, the recent decade has witnessed considerable momentum in unleashing the power of Earth‐abundant 3d transition metal catalysis.^[^ ^17^, ^18^ ^]^ Iron is the most abundant transition metal in the Earth's crust. It is, hence, the metal of choice for late‐stage functionalization^[^ ^19^, ^20^, ^21^ ^]^ of bioactive molecules, given its exceptionally low toxicity as reflected by no limitations in terms of the permitted daily exposure (PDE) of elemental impurities in common drugs (Figure 1).^[^ ^22^, ^23^, ^24^ ^]^

Fluoroalkylations play a crucial role in drug development to adjust and enhance the desired pharmacological properties, reflected by the successful implementation of numerous fluoroalkyl‐bearing pharmaceuticals (Figure 1b).^[^ ^25^, ^26^, ^27^, ^28^, ^29^, ^30^, ^31^, ^32^, ^33^ ^]^ Hence, fluoroalkyl moieties can significantly alter lipophilicity, acidity, bioavailability, metabolic stability, and biological activity, while the exhibited effect of different fluoroalkyl groups is markedly distinct (Figure 1c).^[^ ^25^, ^26^, ^27^, ^28^, ^29^, ^30^, ^31^, ^32^, ^33^ ^]^ As a consequence, we commenced our studies by assessing the lipophilicity (characterized by log *P* values)^[^ ^34^, ^35^ ^]^ of a series of fluoroalkyl radicals through DFT calculation (Figure 1d). Considering the broad range of lipophilicity, the chemo‐ and site‐selective incorporation of such moieties to complex biorelevant molecules could provide a valuable tool to precisely alter lipophilicity in drug design. However, for the introduction of these units the individual reactivity, specifically the radical philicity,^[^ ^36^, ^37^, ^38^ ^]^ poses challenges toward the development of generality‐oriented reaction conditions. Hence, we quantified the radical nucleophilicity of various fluoroalkyl radicals, revealing the lowest nucleophilicity for the trifluoromethyl radical, whereas the nucleophilic character increases significantly across the series of considered species, reaching the highest value for the CF_2_Ph radical (Figure 1d). Despite significant advancements in the domain of C─H fluoroalkylation, these approaches remain severely limited to C─H trifluoromethylation.^[^ ^39^, ^40^, ^41^, ^42^, ^43^, ^44^, ^45^, ^46^, ^47^, ^48^, ^49^, ^50^, ^51^, ^52^, ^53^, ^54^ ^]^ Conversely, C─H fluoroalkylation of biorelevant compounds beyond trifluoromethylation has proven particularly challenging.^[^ ^49^, ^50^, ^51^, ^52^, ^53^, ^54^ ^]^ As a consequence, the full potential for the tuning of pharmacological properties via fluoroalkylation as a highly demanded tool in drug development remains untapped.

Recently, electrochemical C─H fluoroalkylation were realized on biomolecules^[^ ^41^, ^42^, ^43^, ^44^ ^]^ or aryl halides^[^ ^45^ ^]^ using costly fluoroalkyl sulfinates R_F_SO_2_Na. However, the most attractive strategy would arguably be based on inexpensive carboxylic acids as readily available fluoroalkyl sources, thereby combining a cost‐economic building block with an easily available catalyst and an efficient oxidation. Despite major advances, radical formation from fluoroalkylated acids represents a major challenge due to their relatively high oxidation potentials,^[^ ^39^ ^]^ translating into, high cell potential with rather poor functional group tolerance, along with undesired by‐products.^[^ ^51^ ^]^ In this context, a complex WO_3_‐doped FTO glass photoanode was designed for light‐enabled anode oxidation with trifluoroacetates.^[^ ^52^ ^]^ Unfortunately, this complex electrode is difficult to prepare and not commercially available, while redox‐active phthalimido esters^[^ ^50^ ^]^ or pyridine *N*‐oxides auxiliaries,^[^ ^49^ ^]^ were exploited to access fluoroalkyl radicals via decarboxylation. Very recently, photoinduced LMCT‐decarboxylation of CF_3_CO_2_Na allowed for iron‐catalyzed trifluoromethylation under ambient conditions,^[^ ^48^ ^]^ albeit with overstoichiometric amounts of the oxidant, limiting arguably its practical use. Alternatively, activation of sodium trifluoroacetate under ambient conditions with moderate efficiency was achieved by using a well‐defined silver(II) complex.^[^ ^53^ ^]^ In sharp contrast, a user‐friendly homogeneous fluoroalkylation of sensitive and pharmaceutically important substrates and compatible with various solvents, has unfortunately thus far proven elusive.

We, herein, report on a unified strategy for C─H fluoroalkylation enabled by homogeneous ferra‐photoelectrocatalysis. The unique approach proved amenable to a broad fluoroalkyl radical philicity range featuring fluorinated carboxylic acids as versatile and readily available radical precursors. Importantly, the chemo‐ and site‐selective late‐stage functionalization of structurally complex biomolecules proved viable, including unprotected nucleosides (Figure 1e).

## Results and Discussion

**Figure 2:**
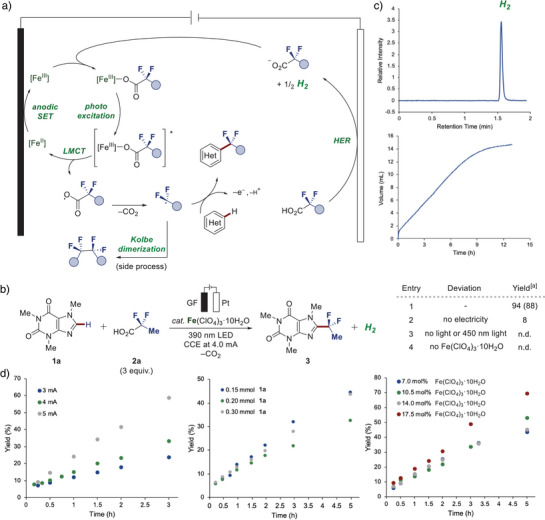
Conceptualization and development of ferra‐photoelectrocatalysis. a) Mechanism manifold for homogenous photoelectrochemical C─H fluoroalkylation. b) Model reaction and control experiments. Reaction conditions: Undivided cell, heteroarene **1a** (0.30 mmol), fluorinated carboxylic acid **2a** (0.90 mmol), Fe(ClO_4_)_3_•10H_2_O (0.06 mmol), LiClO_4_ (0.067 M), MeCN (3 mL), glassy carbon anode (GC) and platinum plate cathode (Pt), CCE at 4.0 mA, 390 nm light irradiation, 1000 rpm stirring rate. [a] Yields were determined by ^19^F NMR with PhCF_3_ as internal standard; isolated yields are shown in parenthesis. c) Detection of molecular hydrogen via GC‐TCD headspace analysis (top) and quantitative monitoring of gas evolution (bottom). d) Kinetic analysis with substrate **1a** and **2a** revealing the current as rate limiting factor.

**Figure 3:**
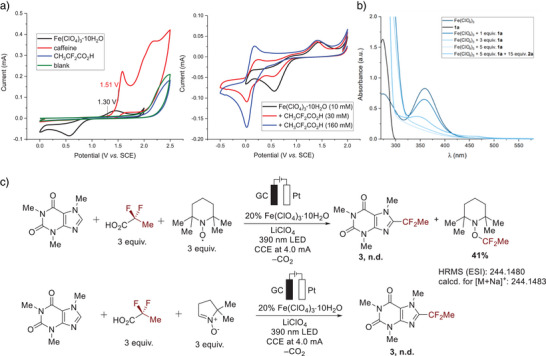
Studies of the LMCT process. a) Cyclic voltammetry curves of a model reaction under standard conditions (left) and CV studies on complexation of Fe(III) salt with carboxylic acid: Undivided cell, caffeine **1a** (0.30 mmol), fluorinated carboxylic acid **2a** (0.90 mmol), Fe(ClO_4_)_3_•10H_2_O (0.06 mmol), LiClO_4_ (0.067 M), MeCN (3 mL). b) UV/Vis spectra of the model reaction. c) Radical trap experiments.

Encouraged by the diverse characteristics featured by fluoroalkyl groups, we conceived the expedient incorporation of such moieties to biorelevant molecules by merging LMCT‐enabled decarboxylation of fluorinated carboxylic acids for the release of fluoroalkyl radicals with readily tunable electrooxidation, thus entering a photoelectrocatalysis regime (Figure 2). Hence, we devised reaction parameters consisting of iron(III) perchlorate as the pre‐catalyst applying galvanostatic electrolysis at 4.0 mA in a user‐friendly undivided cell with 390 nm irradiation for the efficient difluoroethylation of **1a** with fluorinated carboxylic acid **2a** to afford **3** in 88% yield (Figure 2b, entry 1). Here, electricity (entry 2), light irradiation (entry 3), and the iron salt pre‐catalyst (entry 4) were proven to be indispensable. Furthermore, in line with the envisioned ferra‐photoelectrocatalysis manifold, HER was evidenced as the primary cathodic process by the detection of molecular hydrogen in the headspace via gas chromatography (GC‐TCD) analysis along with the gas evolution monitored during the ferra‐photoelectrocatalysis (Figure 2c). Additional insight was provided by a kinetic analysis. Here, electricity was revealed as rate‐determining factor, thus characterizing the current as an expedient mean to restrain the competing Kolbe‐type decarboxylative dimerization of **2a** by controlling the rate of fluoroalkyl radical release (Figure 2d). To gain further insight into the mechanism, we conducted cyclic voltammetry studies, revealing an iron(III)/iron(II) half‐oxidation peak at +1.30 V in MeCN, thus, supporting a ligand‐to‐metal charge transfer (LMCT) pathway via iron(III) carboxylate intermediates (Figure 3).^[^ ^55^, ^56^ ^]^ Iron(III) species exhibit a UV/Vis band with an absorbance maximum around 362 nm (band gap 3.43 eV), which is slightly affected by complexation with substrate **1a** (Figure 3b). Carboxylate coordination has a negligible influence on the absorbance wavelength. Importantly, formation of the radical was supported by radical trap experiments (Figure. 3c). Likewise, EPR spectroscopy analysis confirmed the presence of the proposed radical species in the crude reaction mixture (see Supporting Information for full details).

**Figure 4:**
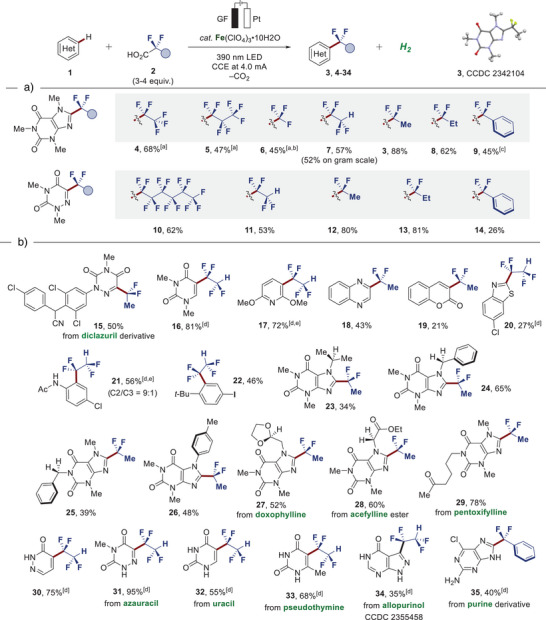
Robustness of homogenous ferra‐photoelectrocatalysis. a) Fluoroalkyl versatility. b) Assessment of chemo‐ and site‐selectivity. Reaction conditions: Undivided cell, heteroarene **1** (0.30 mmol), fluorinated carboxylic acid **2** (0.90 mmol), Fe(ClO_4_)_3_•10H_2_O (0.06 mmol), LiClO_4_ (0.067 M), MeCN (3 mL), glassy carbon anode (GC) and platinum plate cathode (Pt), 6–12 h. CCE at 4.0 mA, 390 nm light irradiation, 1000 rpm stirring rate. Isolated yields. [a] *n*‐Bu_4_NBF_4_ (0.1 M) as supporting electrolyte, 36 h. [b] GF anode. [c] Reaction time 18 h. [d] DMSO/H_2_O (2:1) as a solvent. [e] CPE at 1.5 V; reaction time 30 h.

Intrigued by the resource‐economic nature of the uncovered ferra‐photoelectrocatalysis manifold, we examined the compatibility of diverse fluoroalkyl carboxylic acids for the C─H fluoroalkylation of **1a** and **1b**. To our delight, the desired functionalized products **4**–**14** were yielded efficiently, despite the broad radical philicity spectrum of fluoroalkyl radical species involved. In addition to the exhibited generality in terms of amenable fluorinated carboxylic acids, the delineated strategy proved to be scalable, as showcased by the effective direct functionalization of **1a** to afford **7** on a gram scale (Figure 4) Subsequently, we studied the compatibility of the ferra‐photoelectrocatalysis with various bio‐relevant nitrogen‐ and oxygen‐containing heterocycles, providing access to the fluoroalkylation products **15–20** with excellent site selectivity (Figure 4b), while an aniline derivative and an iodoarene were also tolerated to furnish the desired products **21** and **22**. Next, given that the transformation involves radical species, we were interested to examine whether secondary α‐amino and benzylic C─H bonds, characterized by low bond dissociation energies (BDE), are tolerated.

Gratifyingly, the anticipated products **23**, **24**, **25**, and **26** were obtained featuring such reactive sites, thus showcasing the exceptional chemo‐selectivity of the devised approach. Additionally, hydrolysis‐sensitive dioxolane proved to be compatible, as demonstrated by the efficient formation of doxophylline derivative **27**. Furthermore, we evaluated the robustness with respect to electrophilic carbonyl and ester groups, as present in the xanthine‐derived drug compounds acefylline ester and pentoxifylline, granting access to **28** and **29**. Subsequently, the applicability toward late‐stage C─H fluoroalkylation of diverse nucleobases and nucleobase analogs featuring unprotected N–H sites was pursued, while the functionalized heteroarenes **30**, **31**, **32**, **33**, **34**, and **35** were obtained in up to 95% yield with excellent site‐ and chemo‐selectivity (Figure 4b). The connectivities of products **3** and **34** were unambiguously confirmed by X‐ray diffraction analysis.^[^ ^57^ ^]^

**Figure 5:**
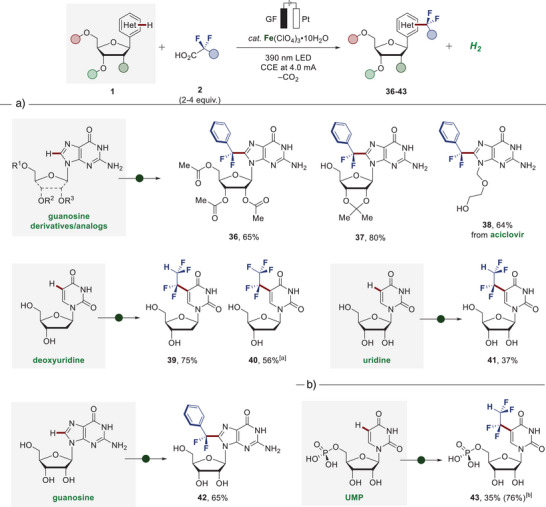
Late‐stage C─H fluoroalkylation. a) Compatibility with diverse nucleosides und nucleoside analogs. b) Selective direct fluoroalkylation of unprotected uridine‐5′‐monophosphate (UMP). Reaction conditions: Undivided cell, heteroarene **1** (0.30 mmol), fluorinated carboxylic acid **2** (0.90–1.20 mmol), Fe(ClO_4_)_3_•10H_2_O (0.06 mmol), LiClO_4_ (0.20–0.30 mmol), DMSO/H_2_O (2:1 v/v, 3 mL), glassy carbon anode (GC) and platinum plate cathode (Pt), 6–12 h. CCE at 4.0 mA, 390 nm light irradiation, 1000 rpm stirring rate. Isolated yields. [a] *n*‐Bu_4_NBF_4_ (0.1 M) as supporting electrolyte, 36 h. [b] ^19^F NMR yield with PhCF_3_ as internal standard in parenthesis.

Considering that fluorine‐containing nucleosides and nucleoside analogs hold marked importance in medicinal chemistry, particularly as antiviral and anticancer agents,^[^ ^25^, ^26^, ^27^, ^28^, ^29^, ^30^, ^31^, ^32^, ^33^, ^58^ ^]^ we were intrigued by exploring our strategy to tackle the formidable challenge toward direct fluoroalkylation of nucleosides. We first examined the photoelectrocatalysis for the functionalization of acetyl and acetal‐protected guanosine, successfully obtaining the desired products **36** and **37** with up to 80% yield (Figure 5) Similarly, the approach proved amenable to the late‐stage functionalization of the antiviral drug aciclovir to yield **38**, thereby displaying tolerance to a free hydroxyl and amino functionalities. Next, we directed our efforts to the C─H fluoroalkylation of entirely unprotected nucleosides. Remarkably, the functionalization of deoxyuridine, uridine, and guanosine was successfully realized, delivering the modified nucleosides **39**, **40**, **41**, and **42** in up to 75% yield. Notably, compounds **40** and **41** represent analogs of the anticancer agent trifluridine. As the ultimate testing ground for chemo‐selectivity, compatibility with unprotected uridine‐5′‐monophosphate (UMP) was demonstrated, providing direct access to the non‐natural UMP derivative **43**, thus underscoring the unique potential of the disclosed ferra‐photoelectrocatalysis for the C─H fluoroalkylation of densely functionalized biomolecules (Figure 5b).

## Conclusion

In conclusion, we have detailed a ferra‐photoelectrocatalysis strategy for direct fluoroalkylation with versatile fluorinated carboxylic acids, allowing for late‐stage functionalization of medicinally relevant scaffolds, including approved drug compounds and entirely unprotected nucleosides. Notably, the delineated approach is distinguished by excellent resource economy, being coupled with the valuable hydrogen evolution reaction (HER), thus highlighting opportunities toward green energy solutions. We believe that the presented strategy will encourage further studies featuring sustainable ferra‐photoelectrocatalysis and provide an enabling tool in the development of novel fluoroalkylated drug candidates.

## Supporting Information

The authors have cited additional references within the Supporting Information.^[^ ^59^, ^60^, ^61^, ^62^, ^63^, ^64^, ^65^, ^66^, ^67^, ^68^, ^69^, ^70^, ^71^, ^72^ ^]^

## Conflict of Interests

The authors declare no conflict of interest.

## Supporting information



Supporting Information

Supporting Information

## Data Availability

The data that support the findings of this study are available in the supplementary material of this article.
