# Supplementary material for: Photoelectrochemical Iron(III) Catalysis for Late‐Stage C─H Fluoroalkylations
Source: Angew Chem Int Ed Engl. 2025 May 7;64(25):e202504143. doi: 10.1002/anie.202504143 (PMC12171357; doi:10.1002/anie.202504143)

# Supplementary materials for

## Photoelectrochemical Iron(III) Catalysis for Late-Stage C–H Fluoroalkylations

Vladimir Motornov[a], Sven Trienes[a,b], Simonetta Resta[a], João C. A. Oliveira[a,b], Zhipeng Lin[a], Zhi Liu[a], Tristan von Münchow[a], Claudia Stückl[c], and Lutz Ackermann*[a,b]

[a] Wöhler Research Institute for Sustainable Chemistry (WISCh), Georg-August-Universität, Tammannstraße 2, 37077, Göttingen, Germany.

[b] German Center for Cardiovascular Research (DZHK), Potsdamer Straße 58, 10785, Berlin, Germany.

[c] Institut für Anorganische Chemie, Tammannstraße 4, 37077 Göttingen, Germany.

* Email: Lutz.Ackermann@chemie.uni-goettingen.de

## Table of contents

General .......... 2
Optimization of reaction conditions .......... 3
General procedure 1 for late-stage radical fluoroalkylation in MeCN .......... 5
General procedure 2 for late-stage radical fluoroalkylation in DMSO/$H_2O$ .......... 5
Characterization data .......... 6
List of unsuccessful examples .......... 18
Gram-scale experiment .......... 18
Kolbe dimerization of $PhCF_2CO_2H$ .......... 18
Radical trap experiments .......... 19
On/off experiments .......... 20
Cyclic voltammetry, UV/Vis and spectroelectrochemistry measurements .......... 21
ICP-MS analysis of iron(III) perchlorate decahydrate .......... 24
Gas evolution study .......... 25
Kinetic analysis .......... 26
Computation studies .......... 29
X-ray crystallography .......... 36
EPR detection of free fluoroalkyl radical .......... 50
References .......... 51
NMR spectra .......... 52

# General

All solvents used for the reactions were HPLC grade. All commercially available chemicals were purchased from commercial suppliers and were used as received. $^{1}H$, $^{13}C$ and $^{19}F$ NMR spectra were measured at ambient temperature using 5 mm diameter NMR tubes. $^{13}C$ NMR spectra were proton decoupled. The chemical shift values ($\delta$) are reported in ppm relative to $Me_4Si$ (0 ppm for $^{1}H$, $^{13}C$ NMR) or $CFCl_3$ (0 ppm for $^{19}F$ NMR). Coupling constants ($J$) are reported in Hertz. For $^{19}F$ NMR yields, $PhCF_3$ was used as an internal standard. Electrocatalysis was conducted using a Metrohm MULTI AUTOLAB M204 potentiostat in constant current mode. Platinum electrodes (10 mm × 15 mm × 0.25 mm, 99.9%; obtained from ChemPur® Karlsruhe, Germany) and glassy carbon (GC) electrodes (25 mm × 10 mm × 1.5 mm), SIGRACELL®GFA 6 EA, obtained from SGL Carbon (Wiesbaden, Germany), were connected using stainless steel adapters. Yields refer to isolated compounds, estimated to be >95% pure as determined by $^{1}H$ NMR. Column chromatography was performed using silica gel 60 (40–63 µm) from Merck. NMR spectra were recorded on a Bruker Avance III 300, Bruker Avance Neo 300, Bruker Avance III HD 400, Bruker Avance III HD 500, or Bruker Avance Neo 600 in the solvent indicated; chemical shifts (δ) are given in ppm relative to the residual solvent peak. All IR spectra were recorded on a Bruker FT-IR Alpha device. High resolution mass spectrometry (HRMS) was measured with APEX IV 7T FTICR. Melting points were measured using Stuart melting point apparatus SMP3, Barloworld Scientific; values are uncorrected. A setup (left) used for the electrophotochemical reactions consisting of two 75 W violet 390 nm LED Kessil lamps, a potentiostat, two fans and a stirring plate and a cell (right) are shown on Fig. 1 below. Iron perchlorate hydrate "$Fe(ClO_4)_3{\cdot}xH_2O$" was purchased from Sigma-Aldrich; iron content corresponding to the formula x = 10.08±0.10 was determined by complexometric titration using EDTA and salicylic acid as an indicator. ICP-MS analysis showed less than 50 ppm content of other metals (Cu, Ni, Co).

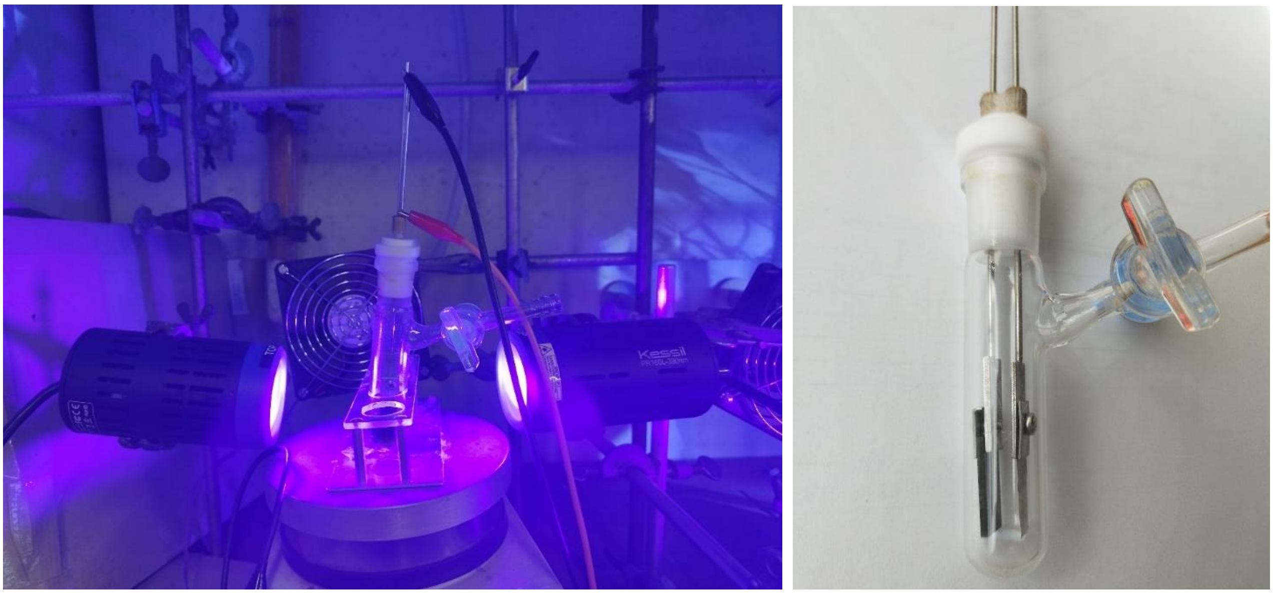

**Figure S1**. Setup (left) and undivided cell (right) used for the electrophotochemical C–H fluoroalkylation.

## Optimization of reaction conditions

**Table S1.** Optimization of electrophotochemical C–H 1,1-difluoroethylation of caffeine.[a]

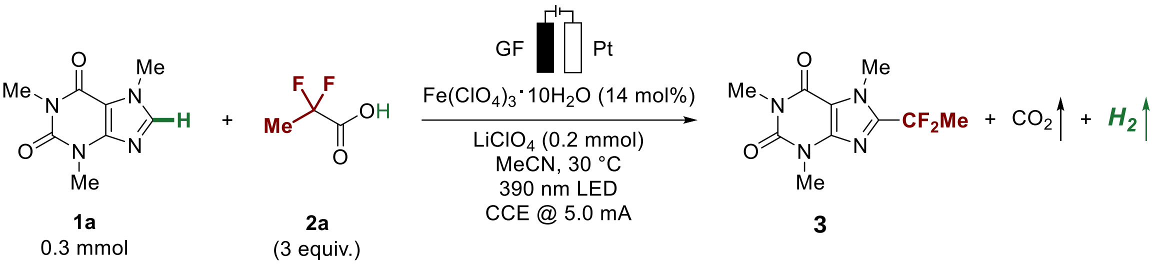


| Entry | deviation from starting conditions | Yield of **3** (%)[b] |
|---|---|---|
| 1 | none | 50 |
| 2 | 4 mA current | 62 (59) |
| 3 | 10 mA current | 13 |
| 4 | 3 mA current | 39 |
| 5 | 4 mA current, GC anode | 79 (80) |
| 6 | 4 mA current, RVC anode | 54 |
| 7 | 4 mA current, GC anode, 10 h | 66 |
| 8 | no $LiClO_4$ | <5 |
| **9** | **4 mA current, GC anode, 20% [Fe]** | **94 (88)** |
| 10 | 4 mA current, GC anode, 7% [Fe] | 51 |
| 11 | no $Fe(ClO_4)_3$, or no light, or 450 nm light | n.d. |
| 12 | no electricity | 8 |
| 13 | $Fe(NO_3)_3·9H_2O$ as a catalyst | n.d. |
| 14 | $CoSO_4·7H_2O$, or $Ni(BF_4)_2·6H_2O$ or $Cu(ClO_4)_2·6H_2O$ as a catalyst | n.d. |
| 15 | 4 mA current, GC anode, $MeCF_2CO_2Na$ instead of **2a** | n.d. |

[a] Starting reaction conditions: caffeine (0.3 mmol), $MeCF_2CO_2H$ (3 equiv.), MeCN (3 mL), graphite felt anode, Pt cathode, $LiClO_4$ (0.3 mmol), CCE @ 5 mA, 390 nm light, 6 h, stirring rate 500 rpm. [b] Yields were determined by $^{19}F$ NMR with $PhCF_3$ as an internal standard; isolated yields are shown in parenthesis.

Note: for poorly soluble substrates such as nucleobases and nucleosides change of the solvent to DMSO/$H_2O$ (2:1) was necessary for the efficient fluoroalkylation (see general procedure 2 below).

Control experiment on the use of the electro-free iron photocatalysis[44] for activation of fluoroalkylated acids besides trifluoroacetate confirms that using photoelectrochemical conditions (Table S1) is significantly more efficient for introduction of $MeCF_2$ and $HCF_2CF_2$. Control experiment on the use of direct metal-free electrolysis of TFA or $HCF_2CF_2CO_2H$ afforded no fluorinated deoxyuridine product under reported conditions.[47] Moreover, recently designed novel $WO_3$-based photoanode[48] was not applicable to this complex substrate. Thus, the developed procedure is advantageous in terms of functional group tolerance.

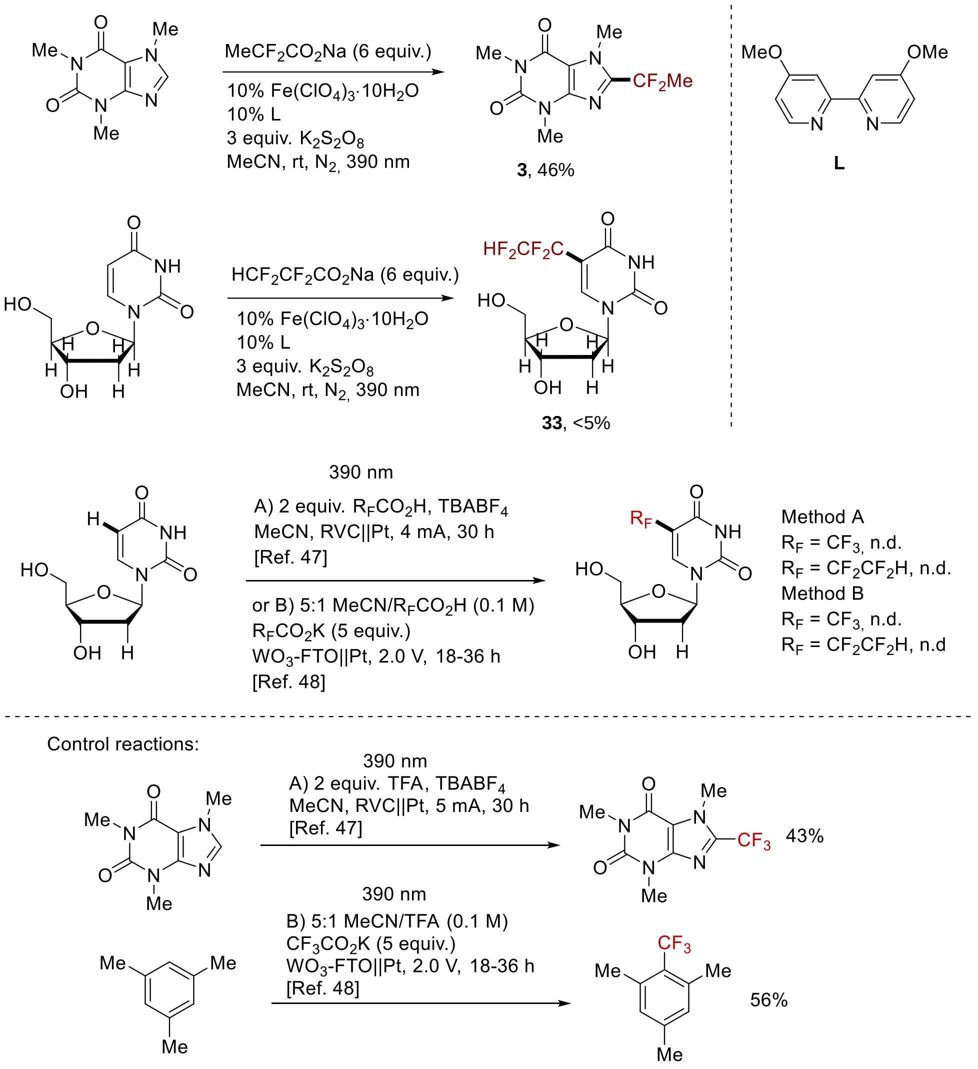


**Figure S2**. Control experiments of comparison to other methods.

## General procedure 1 for late-stage radical fluoroalkylation in MeCN

A 15 mL Schlenk tube (undivided electrochemical cell) charged with a solid mixture of substrate (0.3 mmol), $LiClO_4$ (21 mg, 0.2 mmol), $Fe(ClO_4)_3 \cdot 10H_2O$ (32 mg, 0.06 mmol, 20 mol% unless stated otherwise) and a fluoroalkyl carboxylic acid if solid (0.6-1.2 mmol, 2.0-4.0 equiv.), equipped with a stirring bar (ca. 9 × 2 × 2 mm), Pt cathode (25 × 10 × 0.25 mm) and a glassy carbon (GC) anode (25 × 10 × 1.5 mm) was evacuated and backfilled with nitrogen three times. Then, MeCN (3 mL) or a solution of fluoroalkyl carboxylic acid if liquid (0.6-1.2 mmol, 2.0-4.0 equiv.) in MeCN was added under positive pressure of nitrogen, and the mixture was intensively stirred (1000 rpm) under 390 nm light and constant current electrolysis at 4 mA until a sharp increase of potential from ca. 2.5 to > 4 V is observed by potentiometric curve. Then, the mixture was exposed to air, diluted with 30 mL EtOAc, and washed with 5 mL of 10% $Na_2CO_3$ solution to remove iron residues. Organic layer was separated, dried by anhydrous $Na_2SO_4$, evaporated under reduced pressure and the crude product was purified by column chromatography on silica gel to give the corresponding products.

## General procedure 2 for late-stage radical fluoroalkylation in DMSO/$H_2O$

A 15 mL Schlenk tube (undivided electrochemical cell) equipped with a stirring bar (ca. 9 × 2 × 2 mm), Pt cathode (25 × 10 × 0.2 mm) and glassy carbon (GC) anode (25 × 10 × 1.5 mm) was charged with the substrate (0.3 mmol), $LiClO_4$ (32 mg, 0.3 mmol), and $Fe(ClO_4)_3 \cdot 10H_2O$ (32 mg, 0.06 mmol, 20 mol% unless stated otherwise). Then, the tube was evacuated and backfilled with nitrogen three times. A solution of fluoroalkyl carboxylic acid (0.6-1.2 mmol, 2.0-4.0 equiv.) in DMSO/$H_2O$ mixture (2:1 v/v) (3 ml) was added under positive pressure of nitrogen, and the mixture was intensively stirred (1000 rpm) under 390 nm light and constant current electrolysis at 4 mA for 12 h. Then, the mixture was exposed to air, diluted with 20 mL EtOAc, and 5 mL of saturated aqueous $NaHCO_3$ solution was added. Aqueous layer was extracted with EtOAc (4 × 30 mL), combined organic layers were dried over anhydrous $Na_2SO_4$, evaporated under reduced pressure and the crude product was purified by column chromatography on silica gel to afford the corresponding fluoroalkylation products.

## Characterization data

### 8-(1,1-Difluoroethyl)-1,3,7-trimethyl-3,7-dihydro-1*H*-purine-2,6-dione 3

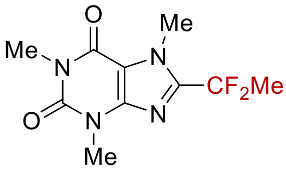


Product **3** was obtained from caffeine (58.5 mg, 0.3 mmol) and 2,2-difluoropropanoic acid (3 equiv.) according to general procedure 1. Column chromatography (hexane/EtOAc 3:1 to 1:1) afforded **3** (68 mg, 88%) as a white solid. X-ray quality crystal was obtained by slow evaporation from EtOAc. CCDC 2342104.

**$^{1}$H NMR** (300 MHz, $CDCl_3$) δ 4.15 (t, *J* = 1.5 Hz, 3H, Me), 3.56 (s, 3H, Me), 3.41 (s, 3H, Me), 2.15 (t, *J* = 19.1 Hz, 3H, $MeCF_2$) ppm; **$^{13}$C NMR** (75 MHz, $CDCl_3$) δ 155.8, 151.7, 146.5, 145.4 (t, *J* = 32.2 Hz, $C_q$–$CF_2$), 118.5 (t, *J* = 234.4 Hz, $CF_2$), 109.4, 33.5 (t, *J* = 3.7 Hz, Me), 29.9 (Me), 28.2 (Me), 23.1 (t, *J* = 24.9 Hz, $MeCF_2$) ppm; **$^{19}$F NMR** (282 MHz, $CDCl_3$) δ -87.2 (q, *J* = 19.1 Hz) ppm; **IR** (ATR): ṽ = 1706, 1656, 1547, 1388, 1178, 1120, 915, 902, 749, 664 $cm^{-1}$; **mp** = 153–155°C; **HRMS** (ESI) *m/z* calcd for $C_{10}H_{13}N_4O_2F_2^+$: 259.1001 $[M+H]^+$, found 259.1010.

### 1,3,7-Trimethyl-8-(perfluoroethyl)-3,7-dihydro-1*H*-purine-2,6-dione 4

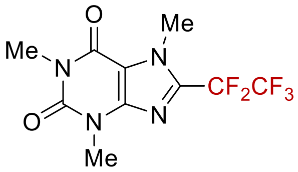


Product **4** was obtained from caffeine (58.5 mg, 0.3 mmol) and perfluoropropionic acid (4.0 equiv.) according to the modified general procedure 1 using $TBABF_4$ (0.3 mmol) as electrolyte, with a reaction time of 36 h. Column chromatography (hexane/EtOAc 4:1 to 3:1) afforded the title compound (64 mg, 68%) as a white solid.

**$^{1}$H NMR** (300 MHz, $CDCl_3$) δ 4.18 (t, *J* = 1.9 Hz, 3H, Me), 3.58 (s, 3H, Me), 3.41 (s, 3H, Me); **$^{13}$C NMR** (75 MHz, $CDCl_3$) δ 155.4, 151.2, 146.8, 137.6 (t, *J* = 28.7 Hz, $C_q$-$CF_2$), 118.1 (qt, *J* = 286.6, 35.8 Hz, $CF_3$), 110.0, 109.2 (tq, *J* = 251.0, 40.1 Hz, $CF_2$), 33.6 (t, *J* = 4.0 Hz, Me), 29.8 (Me), 28.1 (Me); **$^{19}$F NMR** (282 MHz, $CDCl_3$) δ -82.7 (t, *J* = 2.8 Hz, 3F), -111.3 – -111.7 (m, 2F); **mp** = 113–115 °C; **IR** (ATR): ṽ = 1704, 1670, 1329, 1203, 1156, 1112, 996, 968, 929, 740 $cm^{-1}$; **HRMS** (ESI) *m/z* calcd for $C_{10}H_{10}F_5N_4O_2^+$: 313.0718 $[M+H]^+$, found 313.0723.

### 1,3,7-trimethyl-8-(perfluoropropyl)-3,7-dihydro-1*H*-purine-2,6-dione 5

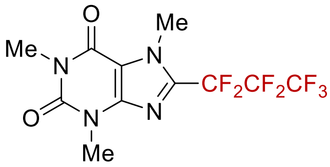


Product **5** was obtained from caffeine (58.5 mg, 0.3 mmol) and heptafluorobutyric acid (4.0 equiv.) according to the modified general procedure 1 using $TBABF_4$ (0.3 mmol) as electrolyte, with a reaction time of 36 h. Column chromatography (hexane/EtOAc 4:1 to 3:1) afforded the title compound (51 mg, 47%) as a white solid.

**$^{1}$H NMR** (300 MHz, $CDCl_3$) δ 4.18 (t, *J* = 1.9 Hz, 3H), 3.58 (s, 3H), 3.41 (s, 3H) ppm; **$^{13}$C NMR** (75 MHz, $CDCl_3$) δ 155.4, 151.2, 147.0, 137.6 (t, *J* = 29.0 Hz), 119.6 (qt, *J* = 288.3, 33.7 Hz, $CF_3$), 113.0 – 104.4 (m, $CF_2$**$CF_2$**$CF_3$), 111.3 (tt, *J* = 257.2, 32.7 Hz, **$CF_2$**$CF_2CF_3$), 110.2, 33.8 (dt, *J* = 4.4, 2.2 Hz, Me), 29.9 (Me), 28.2 (Me) ppm; **$^{19}$F NMR** (282 MHz, $CDCl_3$) δ -80.0 (t, *J* = 9.5 Hz, 3F), -109.8 (q, *J* = 9.5 Hz, 2F), -125.6 (s, 2F) ppm; **mp** = 96–98 °C; **IR** (ATR): ṽ = 1706, 1671, 1546, 1427, 1343, 1192, 1122, 949, 880, 741 $cm^{-1}$; **HRMS** (ESI) *m/z* calcd for $C_{11}H_9F_7N_4O_2Na^+$: 385.0506 $[M+Na]^+$, found 385.0499.

### 1,3,7-Trimethyl-8-(1,1,2,2-tetrafluoroethyl)-3,7-dihydro-1*H*-purine-2,6-dione 6

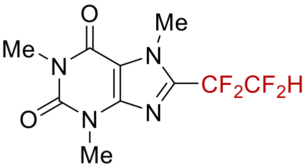


Product **6** was obtained from caffeine (58.5 mg, 0.3 mmol) and 3*H*-tetrafluoropropionic acid (3.0 equiv.) according to the general procedure 1 using 0.3 mmol of $LiClO_4$. Column chromatography (hexane/EtOAc 3:1 to 1:1) afforded the title compound (50 mg, 57%) as a white solid.

**$^{1}$H NMR** (300 MHz, $CDCl_3$) δ 6.40 (tt, *J* = 52.8, 4.8 Hz, 1H, $\underline{H}CF_2CF_2$), 4.18 (t, *J* = 1.8 Hz, 3H, Me), 3.55 (s, 3H, Me), 3.40 (s, 3H, Me) ppm; **$^{13}$C NMR** (75 MHz, $CDCl_3$) δ 155.4, 151.3, 146.8, 139.8 (t, *J* = 28.9 Hz), 110.8 (tt, *J* = 251.0, 27.4 Hz, $CF_2$), 109.6, 108.9 (tt, *J* = 252.0, 32.4 Hz, $CF_2H$), 33.5 (Me), 29.8 (Me), 28.1 (Me) ppm; **$^{19}$F NMR** (282 MHz, $CDCl_3$) δ -113.9 (dddd, *J* = 10.6, 6.7, 4.3, 2.0 Hz, 2F), -137.0 – -137.6 (m, 2F) ppm; **mp** = 139–140 °C; **IR** (ATR): ṽ = 1712, 1667, 1550, 1455, 1232, 1115, 1009, 820, 763, 742 $cm^{-1}$. **HRMS** (ESI) *m/z* calcd for $C_{10}H_{11}F_4N_4O_2^+$: 295.0813 $[M+H]^+$, found 295.0809.

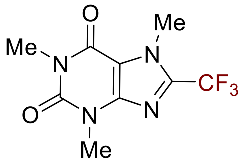

**1,3,7-Trimethyl-8-(trifluoromethyl)-3,7-dihydro-1H-purine-2,6-dione 7**

Product **7** was obtained from caffeine (58.5 mg, 0.3 mmol) and trifluoroacetic acid (4 equiv.) according to modified general procedure 1 (using graphite felt anode, $TBABF_4$ (0.3 mmol) as electrolyte, and with a reaction time of 36 h). Column chromatography (hexane/EtOAc, 3:1 to 1:1) afforded **7** (35 mg, 45%) as a white solid. **$^{1}$H NMR** (300 MHz, $CDCl_3$) δ 4.15 (q, *J* = 1.3 Hz, 3H, Me), 3.58 (s, 3H, Me), 3.41 (s, 3H, Me) ppm; Characterization details matched previously reported data.[40]

**8-(1,1-Difluoropropyl)-1,3,7-trimethyl-3,7-dihydro-1*H*-purine-2,6-dione 8**

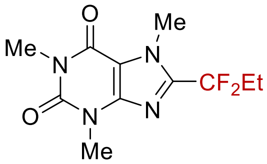

Product **8** was obtained from caffeine (58.5 mg, 0.3 mmol) and 2,2-difluorobutanoic acid (3.0 equiv.) according to the general procedure 1. Column chromatography (hexane/EtOAc 3:1 to 1:1) afforded the title compound (51 mg, 62%) as a white solid.

**$^{1}$H NMR** (300 MHz, $CDCl_3$) δ 4.14 (t, *J* = 1.7 Hz, 3H, Me), 3.54 (s, 3H, Me), 3.39 (s, 3H, Me), 2.46 (tq, *J* = 17.3, 7.5 Hz, 2H, $CF_2CH_2$), 1.16 (t, *J* = 7.5 Hz, 3H, Me) ppm; **$^{13}$C NMR** (75 MHz, $CDCl_3$) δ 155.6, 151.5, 146.5, 145.0 (t, *J* = 32.1 Hz, $\underline{C_q}$-$CF_2$), 119.3 (t, *J* = 237.7 Hz, $CF_2$), 109.1, 33.3 (t, *J* = 4.1 Hz, Me), 29.7 (Me), 29.2 (t, *J* = 23.8 Hz, $CF_2\underline{CH_2}$), 28.0 (Me), 6.1 (t, *J* = 5.3 Hz, $CF_2CH_2\underline{CH_3}$) ppm; **$^{19}$F NMR** (282 MHz, $CDCl_3$) δ -96.3 (m) ppm; **mp** = 123–124 °C; **IR** (ATR): ṽ = 1705, 1657, 1545, 1230, 1164, 1043, 977, 962, 747, 407 $cm^{-1}$; **HRMS** (ESI) *m/z* calcd for $C_{11}H_{15}F_2N_4O_2^+$: 273.1158 $[M+H]^+$, found 273.1167.

**8-(Difluoro(phenyl)methyl)-1,3,7-trimethyl-3,7-dihydro-1*H*-purine-2,6-dione 9**

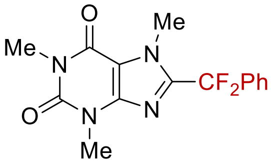

Product **9** was obtained from caffeine (58.5 mg, 0.3 mmol) and α,α-difluorobenzeneacetic acid (3.0 equiv.) according to the general procedure 1, with a reaction time of 18 h. Column chromatography (hexane/EtOAc 3:1 to 1:1) followed by recrystallization from pentane/EtOAc (ca. 20:1) afforded the title compound (43 mg, 45%) as a white amorphous solid.

**$^{1}$H NMR** (300 MHz, $CDCl_3$) δ 7.57 – 7.40 (m, 5H, Ph), 4.04 (t, *J* = 1.6 Hz, 3H, Me), 3.50 (s, 3H, Me), 3.38 (s, 3H, Me) ppm; **$^{13}$C NMR** (75 MHz, $CDCl_3$) δ 155.7, 151.6, 146.8, 145.7 (t, *J* = 34.5 Hz, $\underline{C_q}$-$CF_2$), 133.9 (t, *J* = 25.9 Hz, $\underline{C_q}$-$CF_2$), 131.2 (t, *J* = 2.0 Hz), 128.7, 125.9 (t, *J* = 5.7 Hz), 116.4 (t, *J* = 241.7 Hz, $CF_2$), 109.3, 33.5 (t, *J* = 3.3 Hz, Me), 30.0 (Me), 28.1 (Me) ppm; **$^{19}$F NMR** (282 MHz, $CDCl_3$) δ -89.8 (s) ppm. **IR** (ATR): ṽ = 1706, 1665, 1545, 1444, 1290, 1080, 1041, 764, 747, 695 $cm^{-1}$. **HRMS** (ESI) *m/z* calcd for $C_{15}H_{15}N_4O_2F_2^+$: 321.1158 $[M+H]^+$, found 321.1168.

**2,4-Dimethyl-6-(perfluorohexyl)-1,2,4-triazine-3,5(2*H*,4*H*)-dione 10**

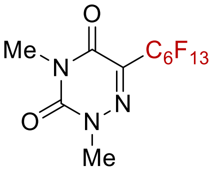

Product **10** was prepared according to the general procedure 1 using 2,4-dimethyl-1,2,4-triazine-3,5(2*H*,4*H*)-dione (42.3 mg, 0.3 mmol) and perfluoroheptanoic acid (3.0 equiv.). Purification by column chromatography (hexane/EtOAc 6:1) afforded the title compound as a white solid (85.2 mg, 62%).

**$^{1}$H NMR** (600 MHz, $CDCl_3$): δ 3.73 (s, 3H, Me), 3.37 (s, 3H, Me) ppm; **$^{13}$C NMR** {$^{1}$H, $^{19}$F} (151 MHz, $CDCl_3$): 152.6 ($C_q$), 148.6 ($C_q$), 130.5 ($C_q$), 118.5 ($C_q$), 116.2 ($C_q$), 112.6 ($C_q$), 111.2 ($C_q$),

110.4 ($C_q$), 108.7 ($C_q$), 40.6 (Me), 27.7 (Me); **$^{19}$F NMR** (565 MHz, $CDCl_3$) δ -80.95 (t, *J* = 10.0 Hz, 3F), -112.5 (t, *J* = 14.4 Hz, 2F), -120.7 – -120.9 (m, 2F), -121.4 – -121.6 (m, 2F), -122.7 – -122.8 (m, 2F), -126.1 – -126.2 (m, 2F) ppm; **mp** = 46–48 °C; **IR** (ATR): $\tilde{\nu}$ = 1737, 1678, 1441, 1238, 1195, 1145, 1012, 745, 517, 482 $cm^{-1}$; **HRMS** (ESI): *m/z* calcd. for $C_{11}H_6N_3O_2F_{13}Na^+$: 482.0145 $[M+Na]^+$, found 482.0149.

**2,4-Dimethyl-6-(1,1,2,2-tetrafluoroethyl)-1,2,4-triazine-3,5(2*H*,4*H*)-dione 11**

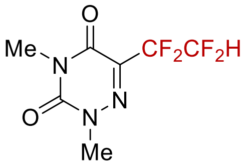

Product **11** was prepared according to the general procedure 1 using 2,4-dimethyl-1,2,4-triazine-3,5(2*H*,4*H*)-dione (42.3 mg, 0.3 mmol) and 2,2,3,3-tetrafluoropropanoic acid (3.0 equiv.). Purification by column chromatography (hexane/EtOAc 5:1) afforded the title compound as a white solid (49.3 mg, 53%).

**$^1$H NMR** (400 MHz, $CDCl_3$) δ 6.47 (tt, *J* = 53.3, 5.6 Hz, 1H, $CF_2CF_2H$), 3.73 (s, 3H, Me), 3.37 (s, 3H, Me) ppm; **$^{13}$C NMR** (101 MHz, $CDCl_3$) δ 154.0 ($C_q$), 148.5 ($C_q$), 132.1 (t, *J* = 25.2 Hz, $C_q$), 111.7 (tt, *J* = 253.2, 27.8 Hz, $C_q$), 109.1 (tt, *J* = 251.4, 32.6 Hz, CH), 40.6 (Me), 27.6 (Me) ppm. **$^{19}$F NMR** (377 MHz, $CDCl_3$) δ -120.9 (td, *J* = 8.4, 5.6 Hz, 2F), -137.7 (dt, *J* = 53.3, 8.4 Hz, 2F) ppm; **mp** = 75–77 °C; **IR** (ATR): $\tilde{\nu}$ = 1731, 1666, 1447, 1218, 1104, 1080, 993, 802, 740, 519 $cm^{-1}$; **HRMS** (ESI): *m/z* calcd. for $C_8H_{11}N_3O_3F_4Na^+$: 296.0629 $[M+MeOH+Na]^+$, found 296.0639.

**6-(1,1-Difluoroethyl)-2,4-dimethyl-1,2,4-triazine-3,5(2*H*,4*H*)-dione 12**

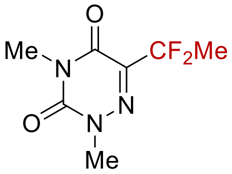

Product **12** was prepared according to the general procedure 1 using 2,4-dimethyl-1,2,4-triazine-3,5(2*H*,4*H*)-dione (42.3 mg, 0.3 mmol) and 2,2-difluoropropanoic acid (3.0 equiv.). Purification by column chromatography (hexane/EtOAc 3.5:1) afforded the title compound as a colorless oil (49.3 mg, 80%).

**$^1$H NMR** (400 MHz, $CDCl_3$) δ 3.67 (s, 3H), 3.35 (s, 3H), 1.96 (t, *J* = 18.7 Hz, 1H) ppm; **$^{13}$C NMR** (101 MHz, $CDCl_3$) δ 153.5 ($C_q$), 149.0 ($C_q$), 136.7 (t, *J* = 29.8 Hz, $\underline{C}$-$CF_2$), 118.7 (t, *J* = 240.3 Hz, $CF_2$), 40.1 ($CH_3$), 27.4 ($CH_3$), 22.5 (t, *J* = 26.1 Hz, $CF_2\underline{Me}$) ppm; **$^{19}$F NMR** (377 MHz, $CDCl_3$) δ -93.1 (q, *J* = 18.7 Hz) ppm; **IR** (ATR): $\tilde{\nu}$ = 1730, 1667, 1439, 1331, 1190, 1131, 1025, 929, 742, 520 $cm^{-1}$; **HRMS** (ESI): *m/z* calcd. for $C_7H_{10}N_3O_2F_2^+$: 206.0736 $[M+H]^+$, found 206.0739.

**6-(1,1-Difluoropropyl)-2,4-dimethyl-1,2,4-triazine-3,5(2*H*,4*H*)-dione 13**

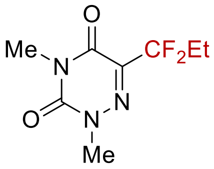

Product **13** was prepared according to the general procedure 1 using 2,4-dimethyl-1,2,4-triazine-3,5(2*H*,4*H*)-dione (42.3 mg, 0.3 mmol) and 2,2-difluorobutanoic acid (3.0 equiv.). Purification by column chromatography (hexane/EtOAc 5:1) afforded the title compound as a white solid (53.3 mg, 81%).

**$^1$H NMR** (400 MHz, $CDCl_3$) δ 3.69 (s, 3H, Me), 3.36 (s, 3H, Me), 2.32 (tq, *J* = 17.1, 7.5 Hz, 2H, $CF_2\underline{CH_2}CH_3$), 1.05 (t, *J* = 7.5 Hz, 3H, $CH_2\underline{CH_3}$) ppm; **$^{13}$C NMR** (101 MHz, $CDCl_3$) δ 153.7 ($C_q$), 149.0 ($C_q$), 136.4 (t, *J* = 29.1 Hz, $\underline{C_q}$-$CF_2$), 119.7 (t, *J* = 243.6 Hz, $CF_2$), 40.2 (Me), 28.6 (t, *J* = 24.9 Hz, $CH_2$), 27.5 (Me), 6.3 (t, *J* = 5.2 Hz, Me) ppm; **$^{19}$F NMR** (377 MHz, $CDCl_3$) δ -102.3 (t, *J* = 17.1 Hz) ppm; **IR** (ATR): $\tilde{\nu}$ = 1724, 1663, 1593, 1440, 1328, 1104, 986, 953, 745, 520 $cm^{-1}$; **HRMS** (ESI): *m/z* calcd. for $C_8H_{12}N_3O_2F_2^+$: 220.0892 $[M+H]^+$, found 220.0896.

**6-(Difluoro(phenyl)methyl)-2,4-dimethyl-1,2,4-triazine-3,5(2*H*,4*H*)-dione 14**

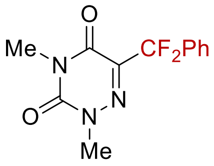

Product **14** was prepared according to the general procedure 1 using 2,4-dimethyl-1,2,4-triazine-3,5(2*H*,4*H*)-dione (42.3 mg, 0.3 mmol) and 2,2-difluoro-2-phenylacetic acid (3.0 equiv.). Purification by column chromatography (hexane/EtOAc 5:1) afforded the title compound as white solid (20.8 mg, 26%).

**$^{1}$H NMR** (400 MHz, $CDCl_3$) δ 7.62 (dd, *J* = 7.6, 2.2 Hz, 2H), 7.49 – 7.40 (m, 3H), 3.69 (s, 3H, Me), 3.30 (s, 3H, Me) ppm; **$^{13}$C NMR** (101 MHz, $CDCl_3$) δ 153.4 ($C_q$), 149.1 ($C_q$), 137.1 (t, *J* = 30.7 Hz, $C_q$), 134.4 (t, *J* = 26.5 Hz, $C_q$), 130.6 (CH), 128.5 (CH), 126.0 (t, *J* = 5.9 Hz, CH), 116.8 (t, *J* = 246.5 Hz, $C_q$), 40.3 (Me), 27.4 (Me) ppm; **$^{19}$F NMR** (377 MHz, $CDCl_3$) δ -98.0 (s) ppm; **mp** = 100–102 °C; **IR** (ATR): $\tilde{\nu}$ = 1727, 1668, 1590, 1452, 1261, 1041, 968, 743, 698, 667 $cm^{-1}$; **HRMS** (ESI): *m/z* calcd. for $C_{12}H_{11}N_3O_2F_2Na^+$: 290.0712 $[M+H]^+$, found 290.0713.

**2-(4-Chlorophenyl)-2-(2,6-dichloro-4-(6-(1,1-difluoroethyl)-4-methyl-3,5-dioxo-4,5-dihydro-1,2,4-triazin-2(3*H*)-yl)phenyl)acetonitrile 15**

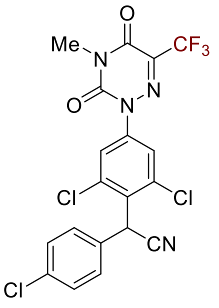


Product **15** was prepared from 2-(4-chlorophenyl)-2-(2,6-dichloro-4-(4-methyl-3,5-dioxo-4,5-dihydro-1,2,4-triazin-2(3*H*)-yl)phenyl)acetonitrile (0.3 mmol, 126.5 mg) according to the general procedure 1. Column chromatography (hexane/EtOAc 5:1) afforded the title compound as a colorless oil (72.9 mg, 50%).

**$^{1}$H NMR** (400 MHz, $CDCl_3$) δ 7.72 (s, 2H), 7.38 – 7.33 (m, 2H), 7.32 – 7.28 (m, 2H), 6.19 (s, 1H), 3.45 (s, 3H), 2.04 (t, *J* = 18.7 Hz, 3H). **$^{13}$C NMR** (101 MHz, $CDCl_3$) δ 152.5 ($C_q$), 147.7 ($C_q$), 140.9 ($C_q$), 138.9 (t, *J* = 30.0 Hz, $C_q$), 136.1 ($C_q$), 134.5 ($C_q$), 130.9 ($C_q$), 130.7 ($C_q$), 129.3 (CH), 128.4 (CH), 125.3 (CH), 120.7 ($C_q$), 118.3 ($C_q$), 116.2 ($C_q$), 115.9 ($C_q$), 37.1 ($CH_3$), 27.9 (CH), 22.6 (t, *J* = 25.8 Hz, $CH_3$).**$^{19}$F NMR** (377 MHz, $CDCl_3$) δ -93.1 (q, *J* = 18.8 Hz); **IR** (ATR): $\tilde{\nu}$ = 1737, 1680, 1592, 1493, 1387, 1319, 1120, 932, 773, 739 $cm^{-1}$; **HRMS** (ESI): *m/z* calcd. for $C_{20}H_{13}Cl_3F_2N_4O_2Na^+$: $[M+Na]^+$ 506.9964, found 506.9968.

**1,3-Dimethyl-5-(1,1,2,2-tetrafluoroethyl)pyrimidine-2,4(1*H*,3*H*)-dione 16**

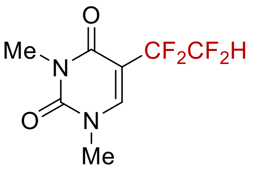


Product **16** was obtained from 1,3-dimethyluracil (42 mg, 0.3 mmol) and 3*H*-tetrafluoropropionic acid (4.0 equiv.) according to general procedure 2. Column chromatography (hexane/EtOAc, 4:3) afforded the title compound (58 mg, 81%) as a colorless oil.

**$^{1}$H NMR** (300 MHz, $CDCl_3$) δ 7.64 (t, *J* = 1.3 Hz, 1H, C6-H), 6.50 (tt, *J* = 53.8, 6.1 Hz, 1H, $CF_2CF_2H$), 3.48 (s, 3H, Me), 3.33 (s, 3H, Me) ppm; **$^{13}$C NMR** (75 MHz, $CDCl_3$) δ 160.4 (t, *J* = 4.4 Hz), 151.0, 144.2 (t, *J* = 9.5 Hz), 113.8 (tt, *J* = 250.2, 27.7 Hz, $CF_2$) 109.1 (tt, *J* = 250.4, 33.5 Hz, $CF_2H$), 104.3 (t, *J* = 24.3 Hz, <u>$C_q$</u>-$CF_2CF_2H$), 37.9, 28.1 ppm; **$^{19}$F NMR** (282 MHz, $CDCl_3$) δ -118.3 (td, *J* = 9.4, 5.7 Hz, 2F), -138.7 (dt, *J* = 53.8, 9.1 Hz, 2F) ppm; **IR** (ATR): ṽ = 1713, 1670, 1657, 1640, 1491, 1458, 1097, 1002, 798, 752 $cm^{-1}$; **HRMS** (ESI) *m/z* calcd for $C_8H_7F_4N_2O_2^-$: 239.0449 $[M-H]^-$, found 239.0445.

**2,6-Dimethoxy-3-(1,1,2,2-tetrafluoroethyl)pyridine 17**

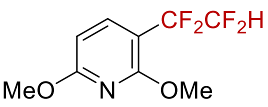


Product **17** was prepared from 2,6-dimethoxypyridine (42 mg, 0.3 mmol) and 3*H*-tetrafluoropropionic acid (4.0 equiv.) according to the General procedure 2 <u>using constant potential electrolysis at 1.5 V with reaction time of 30 h</u>. Column chromatography (pentane/EtOAc 9:1) afforded the title compound **17** as a colorless oil (51 mg, 72%).

**$^{1}$H NMR** (300 MHz, $CDCl_3$) δ 7.75 (d, *J* = 8.3 Hz, 1H), 6.39 (d, *J* = 8.3 Hz, 1H), 6.32 (tt, *J* = 53.9, 5.7 Hz, 1H, $CF_2H$), 4.00 (s, 3H, OMe), 3.95 (s, 3H, OMe); **$^{13}$C NMR** (75 MHz, $CDCl_3$) δ 165.3, 160.1 (t, *J* = 4.9 Hz), 139.8 (t, *J* = 7.1 Hz), 115.0 (tt, *J* = 249.0, 27.0 Hz, $CF_2$), 109.6 (tt, *J* = 250.4, 35.6 Hz, $CF_2H$), 104.6 (t, *J* = 25.3 Hz), 101.9, 54.0 (OMe), 53.9 (OMe); **$^{19}$F NMR** (282 MHz, $CDCl_3$) δ -116.0 (td, *J* = 9.4, 5.7 Hz), -137.3 (dt, *J* = 54.3, 9.7 Hz); **IR** (ATR): $\tilde{\nu}$ = 2956, 1607, 1589, 1391, 1328, 1240, 1095, 1013, 988, 812 $cm^{-1}$; **HRMS** (ESI): *m/z* calcd. for $C_9H_{10}F_4NO_2^+$: 240.0642 $[M+H]^+$, found 240.0636.

**2-(1,1-Difluoroethyl)quinoxaline 18**

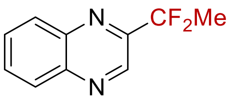


Product **18** was obtained from quinoxaline (39 mg, 0.3 mmol) and 2,2-difluoropropanoic acid (3.0 equiv.) according to the general procedure 1. Column chromatography (pentane/DCM 2:1 to 1:1) afforded the title compound (25 mg, 43%) as a pale-yellow oil.

**$^{1}$H NMR** (300 MHz, $CDCl_3$) δ 9.20 (s, 1H), 8.19 – 8.14 (m, 2H), 7.86 – 7.83 (m, 2H), 2.18 (t, *J* = 18.8 Hz, 3H, $CF_2$Me) ppm; **$^{13}$C NMR** (75 MHz, $CDCl_3$) δ 149.5 (t, *J* = 26.2 Hz, C2), 143.0, 141.7 (t, *J* = 3.7 Hz), 140.9, 131.2, 130.7, 129.8, 129.4, 121.0 (t, *J* = 236.0 Hz, $CF_2$), 22.7 (t, *J* = 26.4 Hz, Me) ppm; **$^{19}$F NMR** (282 MHz, $CDCl_3$) δ -89.6 (q, *J* = 18.7 Hz) ppm; **IR** (ATR): ṽ = 1495, 1387, 1324, 1233, 1188, 1129, 1088, 974, 922, 763 cm$^{-1}$; **HRMS** (ESI) *m/z* calcd for $C_{10}H_9F_2N_2^+$: 195.0728 [M+H]$^+$, found 195.0731.

**3-(1,1-Difluoroethyl)-2*H*-chromen-2-one 19**

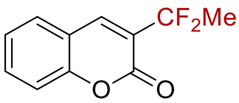


Product **19** was obtained from coumarin (44 mg, 0.3 mmol) and 2,2-difluoropropanoic acid (3.0 equiv.) according to the general procedure 1 using 14 mol% of the catalyst. Column chromatography (pentane/DCM 1:1) afforded the title compound (13 mg, 21%) as a colorless oil, which solidifies upon storage.

**$^{1}$H NMR** (300 MHz, $CDCl_3$) δ 8.03 (br s, 1H), 7.63 – 7.56 (m, 2H), 7.38 – 7.31 (m, 2H), 2.07 (t, *J* = 19.0 Hz, 3H, $CF_2$Me) ppm; **$^{13}$C NMR** (75 MHz, $CDCl_3$) δ 157.9 (br), 154.2, 140.3 (t, *J* = 8.3 Hz, $CF_2CH_3$), 133.0, 129.0, 124.9, 124.3 (t, *J* = 27.4 Hz, $CF_2CH_3$), 119.3 (t, *J* = 240.6 Hz, $CF_2$), 117.8, 116.7, 23.4 (t, *J* = 27.5 Hz, $MeCF_2$) ppm; **$^{19}$F NMR** (282 MHz, $CDCl_3$) δ -90.7 (q, *J* = 18.8 Hz) ppm; **mp** = 74–75°C; **IR** (ATR): ṽ = 1746, 1729, 1612, 1383, 1240, 1180, 1144, 926, 909, 756 cm$^{-1}$; **HRMS** (ESI) *m/z* calcd for $C_{11}H_9F_2O_2^+$: 211.0565 [M+H]$^+$, found 211.0566.

**6-Chloro-2-(1,1,2,2-tetrafluoroethyl)benzo[*d*]thiazole 20**

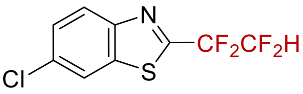


Product **20** was prepared from 5-chlorobenzo[d]thiazole (51 mg, 0.3 mmol) and 3*H*-tetrafluoropropionic acid (4.0 equiv.) according to the modified general procedure 2 using constant potential electrolysis and a reaction time of 30 h. Column chromatography (hexane) afforded the title compound as a colorless oil, which solidifies upon storage (22 mg, 27%).

**$^{1}$H NMR** (300 MHz, $CDCl_3$) δ 8.09 (d, *J* = 8.8 Hz, 1H), 7.99 (d, *J* = 2.1 Hz, 1H), 7.57 (dd, *J* = 8.9, 2.1 Hz, 1H), 6.40 (tt, *J* = 53.0, 4.7 Hz, 1H, $CF_2$H); **$^{13}$C NMR** (75 MHz, $CDCl_3$) δ 158.9 (t, *J* = 31.5 Hz, C2), 151.3, 136.3, 133.7, 128.4,125.7, 121.8, 112.0 (tt, *J* = 251.0, 27.8 Hz, $CF_2$), 109.5 (tt, *J* = 252.3, 34.7 Hz, $CF_2$H); **$^{19}$F NMR** (282 MHz, $CDCl_3$) δ -110.7 (dt, *J* = 7.8, 3.9 Hz), -136.3 (dt, *J* = 53.3, 7.8 Hz); **IR** (ATR): *ṽ* = 2927, 1517, 1311, 1275, 1242, 1223, 1111, 932, 863, 820, 804 cm$^{-1}$; **HRMS** (EI): *m/z* calcd. for $C_9H_4ClF_4NS^+$: 268.9674 [M]$^+$, found 268.9674.

***N*-(4-chloro-2-(1,1,2,2-tetrafluoroethyl)phenyl)acetamide 21 and *N*-(4-chloro-3-(1,1,2,2-tetrafluoroethyl)phenyl)acetamide 21'**

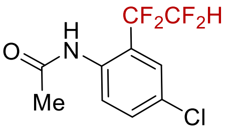


Product **21** was obtained from 4-chloroacetanilide (51 mg, 0.3 mmol) according to the modified general procedure 2 using constant potential electrolysis at 1.5 V, reaction time 30 h. Column chromatography (hexane/EtOAc 4:1 to 1:1) afforded the title compound **21** containing 10% of minor regioisomer (*N*-(4-chloro-3-(1,1,2,2-tetrafluoroethyl)phenyl)acetamide 21') as a yellow solid (42 mg, 56%, r. r. = 9:1).

**$^{1}$H NMR** (300 MHz, $CDCl_3$) δ 8.06 (d, *J* = 8.9 Hz, 1H, NH), 7.61-7.38 (m, 3H, Ar), 5.97 (tt, *J* = 53.9, 2.9 Hz, 1H, $CF_2$H), 2.17 (s, 3H, Ac); **$^{13}$C NMR** (75 MHz, $CDCl_3$) δ 168.6 (C=O), 137.6 (br, $C_q$), 134.7 (t, *J* = 2.5 Hz), 132.5, 130.6, 128.0 (t, *J* = 8.7 Hz), 126.8, 123.8, 116.0 (tt, *J* = 249.3, 29.2 Hz, $CF_2$), 110.3 (tt, *J* = 251.8, 41.8

Hz, $CF_2H$), 24.6 ($CH_3$); **$^{19}$F NMR** (282 MHz, $CDCl_3$) δ -109.9 – -111.0 (m), -133.8 (dt, *J* = 53.8, 5.0 Hz) (major isomer); -115.4 (td, *J* = 8.1, 5.0 Hz), -135.9 (dt, *J* = 53.3, 8.1 Hz) (minor isomer); **IR** (ATR): $\tilde{\nu}$ = 3260, 1668, 1516, 1407, 1286, 1247, 1106, 1010, 831, 814 $cm^{-1}$; **HRMS** (ESI): *m*/*z* calcd. for $C_{10}H_8ClF_4NONa^+$: 270.0303 $[M+Na]^+$, found 270.0308.

**1-(*tert*-Butyl)-4-iodo-2-(1,1,2,2-tetrafluoroethyl)benzene 22**

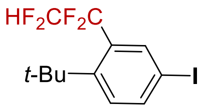


Product **22** was obtained from 1-iodo-4-(*tert*-butyl)benzene (78 mg, 0.3 mmol) according to the general procedure 1. Column chromatography (pentane) afforded the title compound as a colorless oil, which solidifies upon storage (50 mg, 46%).

**$^1$H NMR** (300 MHz, $CDCl_3$) δ 7.93 (dt, *J* = 8.3, 0.9 Hz, 1H), 7.59 (d, *J* = 2.4 Hz, 1H), 7.21 (ddt, *J* = 8.3, 2.5, 0.8 Hz, 1H), 6.35 (tt, *J* = 53.3, 5.0 Hz, 1H, $CF_2H$), 1.32 (s, 9H, *t*-Bu). **$^{13}$C NMR** (75 MHz, $CDCl_3$) δ 152.1, 142.0, 132.7 (t, *J* = 22.8 Hz), 127.2 – 126.1 (m), 130.21 (t, *J* = 1.4 Hz, CH), 114.7 (tt, *J* = 252.6, 26.7 Hz, $CF_2$), 109.3 (tt, *J* = 252.9, 35.4 Hz), 87.3 (t, *J* = 3.5 Hz), 34.9 ($Me_3\underline{C}$), 31.1 (Me). **$^{19}$F NMR** (282 MHz, $CDCl_3$) δ -113.5 (td, *J* = 8.1, 5.0 Hz), -135.6 (dt, *J* = 53.3, 8.1 Hz). **HRMS** (EI): *m*/*z* calcd. for $C_{12}H_{13}F_4I^+$: 395.9993 $[M]^+$, found 395.9996.

**8-(1,1-Difluoroethyl)-7-isopropyl-1,3-dimethyl-3,7-dihydro-1*H*-purine-2,6-dione 23**

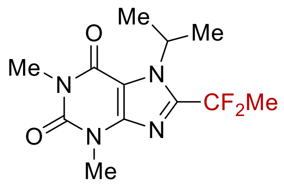


Product **23** was obtained from 7-isopropyl-1,3-dimethyl-3,7-dihydro-1*H*-purine-2,6-dione (67 mg, 0.3 mmol) according to the general procedure 1. Column chromatography (hexane/EtOAc, 3:1 to 1:1) afforded the title compound (29 mg, 34%) as a white solid.

**$^1$H NMR** (300 MHz, $CDCl_3$) δ 5.11 (hept, *J* = 7.0 Hz, 1H), 3.57 (s, 3H, Me), 3.44 (s, 3H, Me), 2.17 (t, *J* = 19.2 Hz, 3H, $CF_2\underline{Me}$), 1.65 (d, *J* = 6.9 Hz, 6H) ppm; **$^{13}$C NMR** (75 MHz, $CDCl_3$) δ 154.5, 151.4, 147.8, 144.6 (t, *J* = 31.3 Hz, $\underline{C_q}$-$CF_2$), 118.6 (t, *J* = 234.7 Hz, $CF_2$), 109.0, 51.3 (t, *J* = 4.6 Hz, CH), 29.8, 28.6, 23.4 (t, *J* = 25.1 Hz, $\underline{Me}CF_2$), 21.4 ppm; **$^{19}$F NMR** (282 MHz, $CDCl_3$) δ -85.5 (q, *J* = 19.4 Hz) ppm; **mp** = 146–147 °C; **IR** (ATR): ṽ = 1705, 1656, 1415, 1179, 1142, 1036, 977, 899, 633, 420 $cm^{-1}$; **HRMS** (ESI) *m*/*z* calcd for $C_{12}H_{17}N_4O_2F_2^+$: 287.1314 $[M+H]^+$, found 287.1320.

**7-Benzyl-8-(1,1-difluoroethyl)-1,3-dimethyl-3,7-dihydro-1*H*-purine-2,6-dione 24**

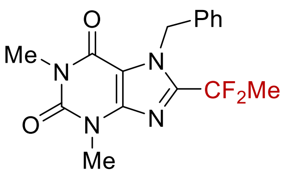


Product **24** was obtained from 7-benzyl-1,3-dimethyl-3,7-dihydro-1*H*-purine-2,6-dione (81 mg, 0.3 mmol) and 2,2-difluoropropanoic acid (3.0 equiv.) according to the general procedure 1. Purification by column chromatography (hexane/EtOAc 3:1) afforded the title compound as a white solid (65 mg, 65%).

**$^1$H NMR** (400 MHz, $CDCl_3$): δ 7.34 – 7.26 (m, 3H), 7.22 (dd, *J* = 8.0, 1.6 Hz, 2H), 5.76 (s, 2H, $NCH_2$), 3.58 (s, 3H, Me), 3.38 (s, 3H), 2.12 (t, *J* = 19.1 Hz, 3H, $CF_2$-$\underline{Me}$) ppm; **$^{13}$C NMR** (101 MHz, $CDCl_3$) δ 155.4 ($C_q$), 151.6 ($C_q$), 146.9 ($C_q$), 145.5 (t, *J* = 32.0 Hz, $\underline{C_q}$-$CF_2$), 136.2 ($C_q$), 128.7 (CH), 128.1 (CH), 127.1 (CH), 118.5 (t, *J* = 236.0 Hz, $CF_2$), 109.0 ($C_q$), 50.0 (t, *J* = 3.6 Hz, $CH_2$), 29.9 (Me), 28.3 (Me), 23.5 (t, *J* = 25.2 Hz, $CF_2\underline{Me}$) ppm; **$^{19}$F NMR** (377 MHz, $CDCl_3$) δ -85.4 (q, *J* = 19.1 Hz) ppm; **mp** = 124–125 °C; **IR** (ATR): ṽ = 1705, 1657, 1543, 1386, 1344, 1185, 1128, 922, 900, 726 $cm^{-1}$; **HRMS** (ESI): *m*/*z* calcd. for $C_{16}H_{17}N_4O_2F_2^+$: 335.1314 $[M+H]^+$, found 335.1314.

**1-Benzyl-8-(1,1-difluoroethyl)-3,7-dimethyl-3,7-dihydro-1*H*-purine-2,6-dione 25**

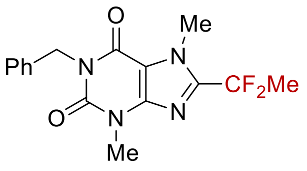


Product **25** was obtained from 1-benzyl-3,7-dimethyl-3,7-dihydro-1*H*-purine-2,6-dione (81 mg, 0.3 mmol) and 2,2-difluoropropanoic acid (3.0 equiv.) according to the general procedure 1 using 14 mol% of catalyst. Purification by column chromatography (hexane/EtOAc 10:1 to 5:1) afforded the title compound as a colorless oil (39.3 mg, 39%).

**$^{1}$H NMR** (400 MHz, $CDCl_3$) δ 7.47 – 7.41 (m, 2H), 7.29 – 7.17 (m, 3H), 5.16 (s, 2H), 4.11 (t, *J* = 1.5 Hz, 3H, Me), 3.51 (s, 3H, Me), 2.11 (t, *J* = 19.1 Hz, 3H, $CF_2$Me) ppm; **$^{13}$C NMR** (101 MHz, $CDCl_3$) δ 155.6 ($C_q$), 151.6 ($C_q$), 146.7 ($C_q$), 145.5 (t, *J* = 31.8 Hz, $C_q$-$CF_2$), 137.3 ($C_q$), 129.0 (CH), 128.5 (CH), 127.7 (CH), 118.4 (t, *J* = 234.7 Hz, $CF_2$), 109.5 ($C_q$), 44.7 ($CH_2$), 33.5 (t, *J* = 3.8 Hz, Me), 29.9 (Me), 23.1 (t, *J* = 24.9 Hz, $CF_2$Me) ppm; **$^{19}$F NMR** (377 MHz, $CDCl_3$) δ -87.2 (m) ppm; **IR** (ATR): ṽ = 1706, 1658, 1545, 1459, 1387, 1127, 921, 898, 749, 700 $cm^{-1}$; **HRMS** (ESI): *m/z* calcd. for $C_{16}H_{17}N_4O_2F_2^+$: 335.1314 [M+H]$^+$, found 335.1315.

**8-(1,1-Difluoroethyl)-1,3-dimethyl-7-(*p*-tolyl)-3,7-dihydro-1*H*-purine-2,6-dione 26**

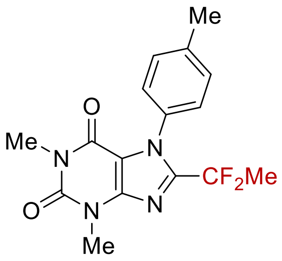


Product **26** was obtained from 1,3-dimethyl-7-(*p*-tolyl)-3,7-dihydro-1*H*-purine-2,6-dione (81 mg, 0.3 mmol) and 2,2-difluoropropanoic acid (3.0 equiv.) according to the general procedure 1. Column chromatography (hexane/EtOAc 3:1 to 1:1) afforded the title compound (48 mg, 48%) as a white solid.

**$^{1}$H NMR** (300 MHz, $CDCl_3$) δ 7.31–7.25 (m, 4H), 3.64 (s, 3H, Me), 3.33 (s, 3H, Me), 2.44 (s, 3H, Me), 2.06 (t, *J* = 18.8 Hz, 3H, $CF_2$Me) ppm; **$^{13}$C NMR** (75 MHz, $CDCl_3$) δ 154.5, 151.5, 146.6, 145.7 (t, *J* = 32.0 Hz, $C_q$–$CF_2$), 140.0, 132.1, 129.4, 126.9 (t, *J* = 1.7 Hz), 117.6 (t, *J* = 237.6 Hz, $CF_2$), 110.3, 29.8, 28.1, 23.5 (t, *J* = 25.4 Hz, Me$CF_2$), 21.3 ppm; **$^{19}$F NMR** (282 MHz, $CDCl_3$) δ -84.0 (q, *J* = 18.8 Hz) ppm; **mp** = 188–190°C; **IR** (ATR): ṽ = 1712, 1670, 1657, 1515, 1420, 1385, 1198, 1173, 1137, 745 $cm^{-1}$. **HRMS** (ESI) *m/z* calcd for $C_{16}H_{17}F_2N_4O_2^+$: 335.1314 [M+H]$^+$, found 335.1323.

**7-((1,3-Dioxolan-2-yl)methyl)-8-(1,1-difluoroethyl)-1,3-dimethyl-3,7-dihydro-1*H*-purine-2,6-dione 27**

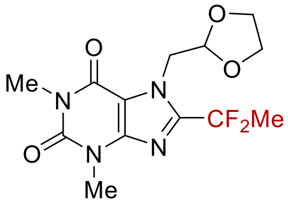


Product **27** was obtained from doxofylline (80 mg, 0.3 mmol) and 2,2-difluoropropanoic acid (3.0 equiv.) according to the general procedure 1. Column chromatography (hexane/EtOAc 1:1 to 1:2) afforded the title compound (51 mg, 52%) as a white solid.

**$^{1}$H NMR** (300 MHz, $CDCl_3$) δ 5.42 (t, *J* = 5.0 Hz, 1H), 4.64 (dt, *J* = 5.1, 0.9 Hz, 2H), 4.06–4.02 (m, 2H), 3.99-3.87 (m, 2H), 3.56 (s, 3H, Me), 3.40 (s, 3H, Me), 2.16 (t, *J* = 19.1 Hz, 3H, $CF_2$Me) ppm; **$^{13}$C NMR** (75 MHz, $CDCl_3$) δ 155.5, 151.5, 146.6, 145.3 (br), 118.3 (t, *J* = 236.1 Hz, $CF_2$), 109.2, 101.7 (t, *J* = 2.1 Hz), 65.1, 48.6, 29.7, 28.1, 23.5 (t, *J* = 25.3 Hz, Me$CF_2$) ppm; **$^{19}$F NMR** (282 MHz, $CDCl_3$) δ -85.6 (q, *J* = 19.1 Hz) ppm; **mp** = 109–112°C; **IR** (ATR): ṽ = 1705, 1657, 1544, 1386, 1190, 1123, 1035, 922, 763, 749 $cm^{-1}$; **HRMS** (ESI) *m/z* calcd for $C_{13}H_{17}N_4O_4F_2^+$ 331.1212 [M+H]$^+$, found 331.1215.

**Ethyl 2-(8-(1,1-difluoroethyl)-1,3-dimethyl-2,6-dioxo-1,2,3,6-tetrahydro-7*H*-purin-7-yl)acetate 28**

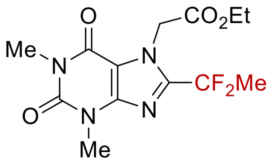


Product **28** was obtained from acefylline methyl ester (80 mg, 0.3 mmol) and 2,2-difluoropropanoic acid (3.0 equiv.) according to the general procedure 1. Column chromatography (hexane/EtOAc 2:1 to 1:2) afforded the title compound (60 mg, 60%) as a white solid.

**$^{1}$H NMR** (300 MHz, $CDCl_3$) δ 5.30 (t, *J* = 1.2 Hz, 2H, $CH_2$), 4.24 (q, *J* = 7.1 Hz, 2H), 3.56 (s, 3H, Me), 3.37 (s, 3H, Me), 2.14 (t, *J* = 19.3 Hz, 3H, $CF_2$Me), 1.28 (t, *J* = 7.1 Hz, 3H, Me) ppm; **$^{13}$C NMR** (75 MHz, $CDCl_3$) δ 166.9, 155.5, 151.4, 146.4, 145.3 (t, *J* = 32.0 Hz, $C_q$–$CF_2$), 118.4 (t, *J* = 235.1 Hz, $CF_2$), 109.0, 62.2, 47.3 (t, *J* = 3.7 Hz), 29.8, 28.0, 22.9 (t, *J* = 24.9 Hz, Me$CF_2$), 14.0 ppm; **$^{19}$F NMR** (282 MHz, $CDCl_3$) δ -86.3 (q, *J* =

19.2 Hz) ppm; **mp** = 157–159 °C; **IR** (ATR): ṽ = 1705, 1657, 1375, 1270, 1205, 1186, 1163, 1129, 902, 749 $cm^{-1}$. **HRMS** (ESI) *m/z* calcd for $C_{13}H_{17}F_2N_4O_4^+$: 331.1212 $[M+H]^+$, found 331.1220.

**8-(1,1-Difluoroethyl)-3,7-dimethyl-1-(5-oxohexyl)-3,7-dihydro-1*H*-purine-2,6-dione 29**

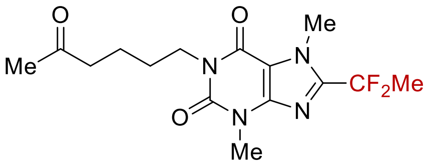


Product **29** was obtained from pentoxifylline (83.5 mg, 0.3 mmol) and 2,2-difluoropropanoic acid (3.0 equiv.) according to the general procedure 1. Column chromatography (hexane/EtOAc 1:1 to 1:2) afforded **29** (80 mg, 78%) as a colorless oil, which solidifies upon storage.

**$^1$H NMR** (300 MHz, $CDCl_3$) δ 4.11 (t, *J* = 1.5 Hz, 3H, Me), 4.00-3.95 (m, 2H), 3.51 (s, 3H, Me), 2.47 (t, *J* = 6.9 Hz, 2H), 2.12 (t, *J* = 19.1 Hz, 3H, $CF_2$Me), 2.11 (s, 3H, Me), 1.63-1.60 (m, 4H) ppm; **$^{13}$C NMR** (75 MHz, $CDCl_3$) δ 208.6 (C=O), 155.4, 151.2, 146.3, 145.2 (t, *J* = 31.8 Hz, $C_q$–$CF_2$), 118.2 (t, *J* = 234.7 Hz, $CF_2$), 109.2, 43.0, 40.8, 33.2 (t, *J* = 3.8 Hz), 29.9, 29.6, 27.3, 22.9 (t, *J* = 25.0 Hz, Me$CF_2$), 20.9 ppm; **$^{19}$F NMR** (282 MHz, $CDCl_3$) δ -87.2 (qd, *J* = 19.1, 1.7 Hz) ppm; **IR** (ATR): ṽ = 1704, 1665, 1657, 1545, 1387, 1175, 1128, 920, 765, 749 $cm^{-1}$; **HRMS** (ESI) *m/z* calcd for $C_{15}H_{21}N_4O_3F_2^+$: 343.1576 $[M+H]^+$, found 343.1578.

**4-(1,1,2,2-Tetrafluoroethyl)pyridazin-3(2*H*)-one 30**

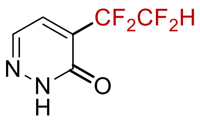


Product **30** was obtained from pyridazine-3(2*H*)-one (29 mg, 0.3 mmol) and 3*H*-tetrafluoropropanoic acid (3.0 equiv.) according to the general procedure 2. Column chromatography (DCM/EtOAc 7:3) afforded the title compound (44 mg, 75%) as a white solid.

**$^1$H NMR** (300 MHz, $CDCl_3$) δ 8.05 (d, *J* = 4.1 Hz, 1H), 7.76 (dt, *J* = 4.1, 1.3 Hz, 1H), 6.73 (tt, *J* = 53.4, 6.1 Hz, 1H, $CF_2CF_2H$) ppm; **$^{13}$C NMR** (75 MHz, $CDCl_3$) δ 160.3 (t, *J* = 4.5 Hz), 138.2, 132.9 (t, *J* = 7.8 Hz), 131.9 (t, *J* = 24.0 Hz, $C_q$-$CF_2$), 114.6 (tt, *J* = 249.9, 27.5 Hz), 110.6 (tt, *J* = 249.6, 32.2 Hz) ppm; **$^{19}$F NMR** (282 MHz, $CDCl_3$) δ -123.1 (q, *J* = 7.8 Hz), -140.1 (dt, *J* = 53.3, 8.4 Hz) ppm; **IR** (ATR): ṽ = 1670, 1609, 1563, 1273, 1233, 1098, 1011, 871, 806, 669 $cm^{-1}$; **mp** = 118–119°C; **HRMS** (ESI) *m/z* calcd for $C_6H_5F_4N_2O^+$: 197.0333 $[M+H]^+$, found 197.0331.

**6-(1,1,2,2-Tetrafluoroethyl)-1,2,4-triazine-3,5(2*H*,4*H*)-dione 31**

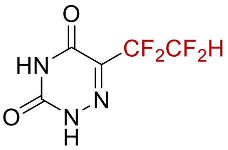


Product **31** was obtained from azauracil (34 mg, 0.3 mmol) and 3*H*-tetrafluoropropionic acid (4.0 equiv.) according to the general procedure 2. Column chromatography (DCM/EtOAc 7:3 to 2:3) afforded the title compound (61 mg, 95%) as a colorless oil.

**$^1$H NMR** (300 MHz, MeOH-$d_4$) δ 6.53 (tt, *J* = 52.6, 5.7 Hz, 1H, $CF_2CF_2H$) ppm; **$^{13}$C NMR** (75 MHz, MeOH-$d_4$) δ 155.3, 148.9, 134.5 (t, *J* = 25.3 Hz, $C_q$-$CF_2$), 113.0 (tt, *J* = 249.5, 27.0 Hz, $CF_2$), 110.2 (tt, *J* = 249.9, 31.7 Hz, $CF_2H$) ppm; **$^{19}$F NMR** (282 MHz, MeOH-$d_4$) δ -121.6 (td, *J* = 8.4, 5.5 Hz, 3F), -139.1 – -139.5 (m, 2F) ppm; **IR** (ATR): ṽ = 1690, 1603, 1428, 1270, 1231, 1103, 1013, 820, 755, 545 $cm^{-1}$; **HRMS** (ESI) *m/z* calcd for $C_5H_2F_4N_3O_2^-$: 212.0089 $[M-H]^-$, found 212.0098.

**5-(1,1,2,2-Tetrafluoroethyl)pyrimidine-2,4(1*H*,3*H*)-dione 32**

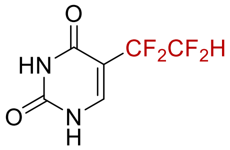


Product **32** was obtained from uracil (34 mg, 0.3 mmol) and 3*H*-tetrafluoropropionic acid (4.0 equiv.) according to the general procedure 2. Column chromatography (DCM/EtOAc 3:1 to 1:1) afforded the title compound (35 mg, 55%) as a white solid.

**$^1$H NMR** (300 MHz, MeCN-$d_3$) δ 9.26 (br s, 2H, NH), 7.73 (t, *J* = 1.3 Hz, 1H), 6.54 (tt, *J* = 53.4, 6.1 Hz, 1H, $CF_2H$) ppm; **$^{13}$C NMR** (75 MHz, MeCN-$d_3$) δ 162.1 (br), 150.8, 144.25 (t, *J* = 9.4 Hz), 115.2 (tt, *J* = 246.5, 27.4 Hz, $CF_2$), 110.3 (tt, *J* = 248.7, 33.7 Hz, $CF_2H$), 104.5 (t, *J* = 24.6 Hz) ppm; **$^{19}$F NMR** (282 MHz, MeCN-

d3) δ -117.8 – -119.0 (m, 2F), -139.7 (dt, *J* = 53.3, 8.9 Hz, 2F) ppm; **IR** (ATR): ṽ = 1727, 1688, 1224, 1118, 1098, 815, 783, 663, 551, 444 $cm^{-1}$; **mp** = 221–222 °C (dec.); **HRMS** (ESI) *m/z* calcd for $C_6H_3F_4N_2O_2^-$: 211.0136 [M-H]$^-$, found 211.0139.

**6-Methyl-5-(1,1,2,2-tetrafluoroethyl)pyrimidine-2,4(1*H*,3*H*)-dione 33**

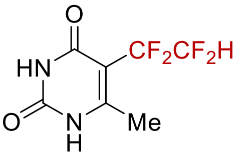


Product **33** was obtained from pseudothymine (38 mg, 0.3 mmol) and 3*H*-tetrafluoropropionic acid (4.0 equiv.) according to the general procedure 2. Column chromatography (hexane/EtOAc 1:1 to 1:2) afforded the title compound (46 mg, 68%) as a white solid.

**$^1$H NMR** (300 MHz, $CDCl_3$) δ 6.59 (tt, *J* = 53.9, 5.8 Hz, 1H, $CF_2CF_2H$), 2.34 (t, *J* = 4.0 Hz, 3H, Me) ppm; **$^{13}$C NMR** (75 MHz, $CDCl_3$) δ 164.0 (t, *J* = 4.8 Hz), 158.5, 151.9, 117.3 (tt, *J* = 250.5, 26.9 Hz, $CF_2H$), 114.5 – 107.7 (m, $CF_2$), 102.6 (t, *J* = 24.0 Hz), 18.3 – 18.0 (m, 6-Me) ppm; **$^{19}$F NMR** (282 MHz, $CDCl_3$) δ -111.0 – -113.3 (m, 2F), -140.17 (dt, *J* = 53.8, 8.4 Hz) ppm; **mp** = 140–141 °C; **HRMS** (ESI) *m/z* calcd for $C_7H_6F_4N_2O_2Na^+$: 249.0258 [M+Na]$^+$, found: 249.0260.

**3-(1,1,2,2-Tetrafluoroethyl)-1,5-dihydro-4*H*-pyrazolo[3,4-d]pyrimidin-4-one 34**

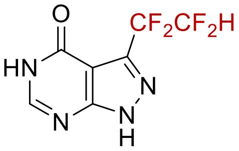


Product **34** was obtained from allopurinol (29 mg, 0.3 mmol) and 3*H*-tetrafluoropropanoic acid (4.0 equiv.) according to the general procedure 2. Column chromatography (DCM/MeOH, 19:1) afforded the title compound (44 mg, 35%) as a white solid.

**$^1$H NMR** (400 MHz, MeOH-d4): δ 8.06 (s, 1H), 6.81 (tt, *J* = 52.8, 5.6 Hz, 1H, $CF_2CF_2H$) ppm; **$^{13}$C NMR** (101 MHz, MeOH-d4) δ 158.5, 156.6, 149.9, 139.8 (t, *J* = 28.0 Hz, $C_q$-$CF_2$), 113.4 (tt, *J* = 248.0, 25.8 Hz, $CF_2$), 111.3 (tt, *J* = 249.0, 33.4 Hz, $CF_2H$), 104.5 ppm; **$^{19}$F NMR** (377 MHz, MeOH-d4) δ -117.3 (td, *J* = 9.2, 5.6 Hz), -140.1 (dt, *J* = 52.8, 9.1 Hz) ppm; **mp** = 176–180 °C; **IR** (ATR): ṽ = 1680, 1599, 1572, 1482, 1311, 1233, 1103, 983, 907, 815 $cm^{-1}$; **HRMS** (ESI): *m/z* calcd. for $C_7H_4F_4N_4ONa^+$: 259.0213 [M+Na]$^+$, found 259.0220.

**6-Chloro-8-(difluoro(phenyl)methyl)-4,5-dihydro-9*H*-purin-2-amine 35**

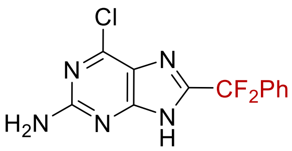


Product **35** was obtained from 2-amino-6-chloropurine (51 mg, 0.3 mmol) and α,α-difluorobenzeneacetic acid (2.0 equiv.) according to the general procedure 2. Column chromatography (DCM/EtOAc 8:2) afforded the title compound (37 mg, 40%) as a yellow solid.

**$^1$H NMR** (300 MHz, DMSO-d6) δ 7.65-7.52 (m, 5H, Ph), 6.98 (s, 2H, $NH_2$) ppm; **$^{13}$C NMR** (75 MHz, DMSO-d6) δ 160.5, 155.8, 155.2, 146.7 (t, *J* = 35.1 Hz), 134.0 (t, *J* = 26.3 Hz), 131.1 (br s, CH), 128.9 (CH), 125.6 (t, *J* = 5.6 Hz), 116.2 (t, *J* = 240.3 Hz, $CF_2$) ppm; **$^{19}$F NMR** (282 MHz, DMSO-d6) δ -90.0 (s, 2F) ppm; **IR** (ATR): ṽ = 3503, 3320, 3206, 2927, 1640, 1567, 1524, 1229, 1025, 993, 898, 691 $cm^{-1}$; mp = 158-162 °C (dec.) **HRMS** (ESI) *m/z* calcd for $C_{12}H_9F_2N_5Cl^+$: 296.0509 [M+H]$^+$, found 296.0517.

**(2*R*,3*S*,4*S*,5*S*)-2-(Acetoxymethyl)-5-(2-amino-8-(difluoro(phenyl)methyl)-6-oxo-1,4,5,6-tetrahydro-9*H*-purin-9-yl)tetrahydrofuran-3,4-diyl diacetate 36**

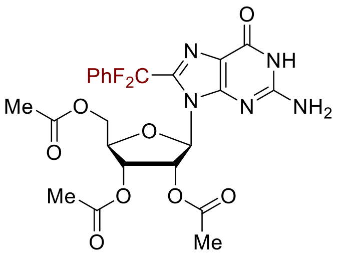


Product **36** was obtained from guanosine triacetate (122 mg, 0.3 mmol) and α,α-difluorobenzeneacetic acid (2.0 equiv.) according to the general procedure 2. Column chromatography (DCM/EtOAc 7:3 to EtOAc) afforded **36** (105 mg, 65%) as a beige sticky oil. After chromatography, residual DMSO has been removed by fast trituration with $H_2O$.

$^{1}$H NMR (300 MHz, DMSO-$d_6$) δ 11.02 (br s, 1H), 7.54 (s, 5H), 6.75 (br s, 1H), 6.14 (dd, *J* = 6.6, 4.5 Hz, 1H), 6.02 (d, *J* = 4.6 Hz, 1H), 5.65 (t, *J* = 6.1 Hz, 1H), 4.48-4.23 (m, 3H), 2.08 (s, 3H, Me), 2.00 (s, 3H, Me), 1.97 (s, 3H, Me) ppm; **$^{13}$C NMR** (75 MHz, DMSO-$d_6$) δ 170.7, 169.9, 169.6, 157.1, 154.8, 153.0, 139.9 (t, *J* = 34.5 Hz), 134.1 (t, *J* = 25.1 Hz), 131.5, 129.0, 126.4 (t, *J* = 5.4 Hz), 117.6 (t, *J* = 235.5 Hz, $CF_2$), 116.5, 88.0, 80.1, 71.6, 70.6, 63.4, 20.9 (Me), 20.7 (Me), 20.5 (Me) ppm; **$^{19}$F NMR** (282 MHz, DMSO-$d_6$) δ -84.1 (d, *J* = 272.3 Hz, 1F), -86.4 (d, *J* = 272.3 Hz, 1F) ppm; **IR** (ATR): ṽ = 3315, 3154, 2926, 1447, 1687, 1630, 1572, 1370, 1221, 1030, 701 $cm^{-1}$; **HRMS** (ESI) *m/z* calcd for $C_{23}H_{24}F_2N_5O_8^+$: 536.1587 $[M+H]^+$, found 536.1600.

**2-Amino-8-(difluoro(phenyl)methyl)-9-((3aR,4R,6R,6aR)-6-(hydroxymethyl)-2,2-dimethyltetrahydrofuro[3,4-*d*][1,3]dioxol-4-yl)-1,4,5,9-tetrahydro-6*H*-purin-6-one 37**

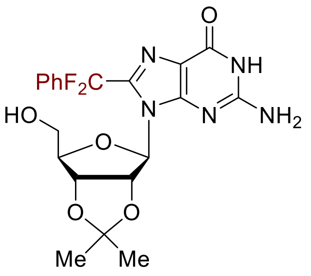


Product **37** was obtained from guanosine 3,4-acetonide (106 mg, 0.3 mmol) and α,α-difluorobenzeneacetic acid (2 equiv.) according to the general procedure 2. Column chromatography (DCM/EtOAc 1:1 to EtOAc/MeOH 8:2) afforded **37** (105 mg, 80%) as a beige solid. After chromatography, residual DMSO has been removed by fast trituration with $H_2O$.

**$^{1}$H NMR** (300 MHz, MeOH-$d_4$) δ 7.58-7.44 (m, 5H), 6.17 (d, *J* = 3.0 Hz, 1H), 5.43 (dd, *J* = 6.3, 2.9 Hz, 1H), 5.12 (dd, *J* = 6.3, 3.4 Hz, 1H), 4.22 (td, *J* = 5.0, 3.3 Hz, 1H), 3.89 – 3.69 (m, 2H). 1.50 (s, 3H, Me), 1.34 (s, 3H, Me) ppm; **$^{13}$C NMR** (75 MHz, MeOH-$d_4$) δ 159.5, 155.6, 153.9, 142.7 (t, *J* = 34.5 Hz), 135.5 (t, *J* = 25.3 Hz), 132.0, 129.6, 127.2 (t, *J* = 5.4 Hz), 118.3 (t, *J* = 238.4 Hz, $CF_2$), 117.5, 115.2, 92.1, 88.7, 84.6, 83.0, 63.7, 27.7 (Me), 25.7 (Me) ppm; **$^{19}$F NMR** (282 MHz, MeOH-$d_4$) δ -87.2 (s, 1F), -87.3 (s, 1F) ppm; **IR** (ATR): ṽ = 3505, 3319, 3209, 2928, 1648, 1568, 1499, 1236, 1027, 762, 693 $cm^{-1}$; **mp** = 190-191 °C (dec.); **HRMS** (ESI) *m/z* calcd for $C_{20}H_{21}F_2N_5O_5Na^+$: 472.1403 $[M+Na]^+$, found 472.1412.

**2-Amino-8-(difluoro(phenyl)methyl)-9-((2-hydroxyethoxy)methyl)-1,4,5,9-tetrahydro-6*H*-purin-6-one 38**

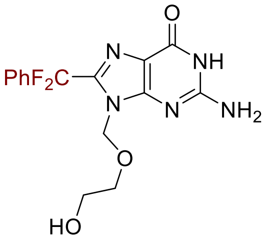


Product **38** was obtained from aciclovir (67 mg, 0.3 mmol) and α,α-difluorobenzeneacetic acid (2.0 equiv.) according to the general procedure 2. Column chromatography (DCM/EtOAc 1:1 to EtOAc/MeOH 9:1) afforded **38** (68 mg, 64%) as a yellowish solid. After chromatography, remaining DMSO has been removed by trituration with DCM.

**$^{1}$H NMR** (300 MHz, DMSO-$d_6$) δ 10.81 (br s, 1H), 7.60 – 7.50 (m, 5H), 6.76 (br s, 2H), 5.49 (br s, 2H), 4.66 (t, *J* = 5.3 Hz, 1H), 3.56 – 3.34 (m, 3H) ppm; **$^{13}$C NMR** (75 MHz, DMSO-$d_6$) δ 156.6, 154.7, 153.3, 140.1 (t, *J* = 34.6 Hz), 134.4 (t, *J* = 25.6 Hz), 130.7, 128.4, 126.0 (t, *J* = 5.6 Hz), 117.1 (d, *J* = 238.7 Hz, $CF_2$), 115.3, 72.2 ($CH_2$), 70.7 ($CH_2$), 59.9 ($CH_2$) ppm; **$^{19}$F NMR** (282 MHz, DMSO-$d_6$) δ -87.5 (s, 2F) ppm; **IR** (ATR): ṽ = 3347, 3149, 2921, 1690, 1652, 1605, 1567, 1063, 1022, 691 $cm^{-1}$; **mp** = 151–153 °C; **HRMS** (ESI) *m/z* calcd for $C_{15}H_{15}F_2N_5O_3^+$: 352.1216 $[M+H]^+$, found 352.1223.

**1-((2*R*,4*S*,5*R*)-4-Hydroxy-5-(hydroxymethyl)tetrahydrofuran-2-yl)-5-(1,1,2,2-tetrafluoroethyl)pyrimidine-2,4(1*H*,3*H*)-dione 39**

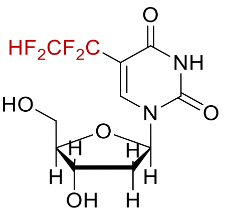


Product **39** was obtained from deoxyuridine (68 mg, 0.3 mmol) and 3*H*-tetrafluoropropionic acid (4.0 equiv.) according to general procedure 2. Column chromatography (DCM/EtOAc 1:1 to 1:2) afforded the title compound (74 mg, 75%) as a white solid.

**$^{1}$H NMR** (300 MHz, MeOH-d$_4$) δ 8.69 (t, *J* = 1.3 Hz, 1H), 6.52 (tt, *J* = 53.6, 6.1 Hz, 1H, $CF_2CF_2H$), 6.25 (t, *J* = 6.3 Hz, 1H), 4.42 (dt, *J* = 6.1, 3.9 Hz, 1H), 3.97 (q, *J* = 3.0 Hz, 1H), 3.89 – 3.69 (m, 2H), 2.41 – 2.21 (m, 2H) ppm; **$^{13}$C NMR** (75 MHz, MeOH-d$_4$) δ 162.4 (t, *J* = 4.2 Hz), 151.4, 143.8 (t, *J* = 9.4 Hz), 115.3 (tt, *J* = 247.9, 26.4 Hz, $CF_2$), 110.7 (tt, *J* = 249.2, 34.1 Hz, $CF_2H$), 105.9 (t, *J* = 24.3 Hz, $C_q$-$CF_2$), 89.3, 87.5, 71.9, 62.3, 42.1 ppm; **$^{19}$F NMR** (282 MHz, MeOH-d$_4$) δ -118.7 – -119.1 (m, 2F), -139.3 – -140.8 (m, 2F) ppm; **mp** = 129-130 °C; **IR** (ATR): ṽ = 3400 (br), 1680, 1480, 1299, 1272, 1093, 1015, 785, 594 cm$^{-1}$. **HRMS** (ESI) *m/z* calcd for $C_{11}H_{12}F_4N_2O_5Na^+$: 351.0575 [M+Na]$^+$, found 351.0578.

**1-((2*R*,4*S*,5*R*)-4-Hydroxy-5-(hydroxymethyl)tetrahydrofuran-2-yl)-5-(perfluoroethyl)pyrimidine-2,4(1*H*,3*H*)-dione 40**

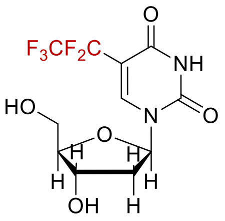


Product **40** was obtained from deoxyuridine (68 mg, 0.3 mmol) and perfluoropropionic acid (4.0 equiv.) according to general procedure 2, using $TBABF_4$ (0.3 mmol) as supporting electrolyte, with a reaction time of 36 h. Column chromatography (DCM/EtOAc 7:3 to 1:2) afforded the title compound (58 mg, 56%) as a colorless oil.

**$^{1}$H NMR** (300 MHz, MeCN-d$_3$) δ 9.35 (br s), 8.64 (t, *J* = 1.1 Hz, 1H), 6.11 (t, *J* = 6.0 Hz, 1H), 4.34 (dq, *J* = 6.3, 4.4 Hz, 1H), 3.91 (dt, *J* = 4.4, 2.8 Hz, 1H), 3.73 (qdd, *J* = 12.0, 4.4, 2.8 Hz, 2H), 3.42 (d, *J* = 4.4 Hz, 1H), 3.31 (t, *J* = 4.5 Hz, 1H), 2.37 – 2.16 (m, 2H) ppm; **$^{13}$C NMR** (75 MHz, MeCN-d$_3$) δ 159.8 (br), 150.5, 145.3 (t, *J* = 9.7 Hz), 122.5-109.9 (m, $CF_2CF_3$, signals overlap with solvent), 102.2 (t, *J* = 23.8 Hz), 88.7, 87.3, 70.8, 61.5, 41.9 ppm; **$^{19}$F NMR** (282 MHz, MeCN-d$_3$) δ -84.9 (t, *J* = 2.1 Hz, 3F, $CF_3$), -111.2 – -114.9 (m, 2F, $CF_2$) ppm; **IR** (ATR): ṽ = 1735, 1680, 1468, 1368, 1210, 1095, 998, 809, 592, 560 cm$^{-1}$; **HRMS** (ESI) *m/z* calcd for $C_{17}H_{19}F_4N_2O_9^+$: 471.1021 [M+H]$^+$, found 471.1013.

**1-((2*R*,3*R*,4*S*,5*R*)-3,4-Dihydroxy-5-(hydroxymethyl)tetrahydrofuran-2-yl)-5-(1,1,2,2-tetrafluoroethyl)pyrimidine-2,4(1*H*,3*H*)-dione 41**

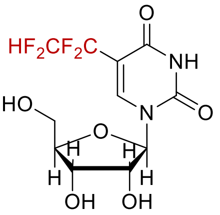


Product **41** was obtained from uridine (73 mg, 0.3 mmol) and 3*H*-tetrafluoropropionic acid (4.0 equiv.) according to the general procedure 2. Column chromatography (DCM/EtOAc 1:1 to 1:2) afforded the title compound (38 mg, 37%) as a colorless oil, which solidifies upon storage.

**$^{1}$H NMR** (300 MHz, MeOH-d$_4$) δ 8.80 (t, *J* = 1.3 Hz, 1H), 6.53 (tt, *J* = 53.6, 6.1 Hz, 1H, $CF_2CF_2H$), 5.91 (d, *J* = 3.3 Hz, 1H), 4.24 – 4.15 (m, 2H), 4.06 (dt, *J* = 4.8, 2.4 Hz, 1H), 3.91 (dd, *J* = 12.1, 2.5 Hz, 1H), 3.75 (dd, *J* = 12.1, 2.2 Hz, 1H) ppm; **$^{13}$C NMR** (75 MHz, $CD_3CN$) δ 162.4 (t, *J* = 4.2 Hz), 151.6, 144.0 (t, *J* = 9.4 Hz), 115.3 (tt, *J* = 247.7, 26.5 Hz, $CF_2$), 110.7 (tt, *J* = 248.6, 34.4 Hz, $CF_2H$), 106.0 (t, *J* = 24.3 Hz, $C_q$-$CF_2$), 91.4, 86.3, 76.4, 70.8, 61.4 ppm; **$^{19}$F NMR** (282 MHz, MeOH-d$_4$) δ -118.6 – -119.0 (m, 2F), -140.1 – -140.6 (m, 2F) ppm; **IR** (ATR): ṽ = 1686, 1474, 1275, 1098, 1058, 1007, 813, 655, 597, 561 cm$^{-1}$; **HRMS** (ESI) *m/z* calcd for $C_{11}H_{11}F_4N_2O_6^-$: 343.0559 [M-H]$^-$, found 343.0551.

**2-Amino-8-(difluoro(phenyl)methyl)-9-((2*R*,3*R*,4*S*,5*R*)-3,4-dihydroxy-5-(hydroxymethyl)tetrahydrofuran-2-yl)-1,4,5,9-tetrahydro-6*H*-purin-6-one 42**

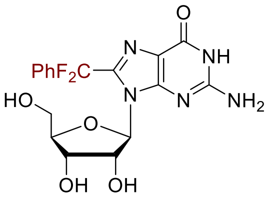

Product **42** was obtained from guanosine (85 mg, 0.3 mmol) and α,α-difluorobenzeneacetic acid (2 equiv.) according to the general procedure. Column chromatography (DCM/EtOAc 1:1 to EtOAc/MeOH 9:1) afforded the title compound (80 mg, 65%) as a yellowish solid.

**$^{1}$H NMR** (300 MHz, DMSO-d$_6$) δ 10.89 (br s, 1H), 7.56 (s, 5H), 6.60 (br s, 1H), 5.95 (d, *J* = 5.8 Hz, 1H), 5.07 (t, *J* = 5.7 Hz, 1H), 4.23 (dd, *J* = 5.7, 3.6 Hz, 1H), 3.87 (q, *J* = 5.2 Hz, 1H), 3.70 (dd, *J* = 11.8, 4.7 Hz, 1H), 3.55 (dd, *J* = 11.8, 5.7 Hz, 1H) ppm; **$^{13}$C NMR** (75 MHz, DMSO-d$_6$) δ 156.5, 154.0, 152.5, 140.5 (m), 133.1 (m), 130.8 (t, *J* = 253.7 Hz, CF$_2$), 130.5, 128.4, 126.2 (t, *J* = 4.6 Hz), 116.4, 89.7, 86.0, 70.9, 70.7, 62.2 ppm; **$^{19}$F NMR** (282 MHz, DMSO-d$_6$) δ -83.4 (d, *J* = 272.8 Hz, 1F), -85.4 (d, *J* = 272.8 Hz, 1F) ppm; **IR** (ATR): ṽ = 3455, 3333, 3217, 3170, 2935, 1694, 1630, 1590, 1078, 1015 cm$^{-1}$; **mp** = 153-156 °C (dec.); **HRMS** (ESI) *m*/*z* calcd for $C_{17}H_{17}F_2N_5O_2Na^+$: 432.1090 [M+Na]$^+$, found 432.1095.

**((2*R*,3*S*,4*R*,5*R*)-5-(2,4-Dioxo-5-(1,1,2,2-tetrafluoroethyl)-3,4-dihydropyrimidin-1(2*H*)-yl)-3,4-dihydroxytetrahydrofuran-2-yl) methyl dihydrogen phosphate 43**

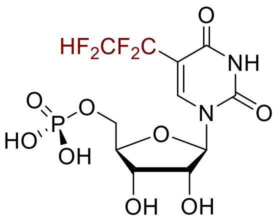


Product **43** was obtained from uridine monophosphoric acid (UMP) disodium salt (111 mg, 0.3 mmol) and 3*H*-tetrafluoropropionic acid (3.0 equiv.) according to the general procedure 2. Afterwards, the mixture was filtered and concentrated under reduced pressure. $^{19}$F NMR yield 76%.

**$^{19}$F NMR** (282 MHz, D$_2$O) δ -122.8 (td, *J* = 8.4, 5.5 Hz, 2F), -137.8 (dt, *J* = 52.8, 7.8 Hz, 2F) ppm; **$^{31}$P NMR** (122 MHz, D$_2$O) δ 4.85 ppm;

Purification of the residue was accomplished by treatment with excess of triethylamine without extraction, followed by preparative reverse-phase HPLC (MeCN/H$_2$O) to give product in a form of triethylammonium salt in 35% yield.

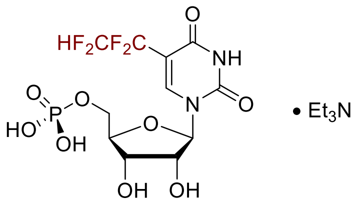


**$^{1}$H NMR** (300 MHz, D$_2$O + 1 drop DMSO) δ 6.47 (tt, *J* = 53.1, 5.5 Hz, 1H, CF$_2$H), 5.95 (d, *J* = 4.8 Hz, 1H), 4.42 (t, *J* = 4.8 Hz, 1H), 4.37 – 4.27 (m, 2H), 4.20 – 4.04 (m, 2H), 3.21 (q, *J* = 7.3 Hz, Et$_3$NH$^+$), 1.28 (t, *J* = 7.3 Hz, Et$_3$NH$^+$). **$^{13}$C NMR** (101 MHz, D$_2$O + 1 drop DMSO) δ 162.1, 151.0, 142.9 (t, *J* = 9.4 Hz), 116.2-111.8 (br m, CF$_2$), 109.2 (tt, *J* = 245.7, 33.0 Hz, CF$_2$H), 105.0 (t, *J* = 25.1 Hz), 89.6, 83.6 (d, *J* = 8.7 Hz), 74.0, 69.6, 64.1 (d, *J* = 4.7 Hz), 46.6 (Et$_3$NH$^+$), 8.2 (Et$_3$NH$^+$). **$^{19}$F NMR** (282 MHz, D$_2$O + 1 drop DMSO) δ -116.4 – -117.2 (m), -137.5 (dtd, *J* = 53.3, 8.4, 5.0 Hz); **HRMS** (ESI) *m*/*z* calcd for $C_{11}H_{12}F_4N_2O_9P^-$: 423.0222 [M-H]$^-$, found 423.0217.

## List of unsuccessful examples

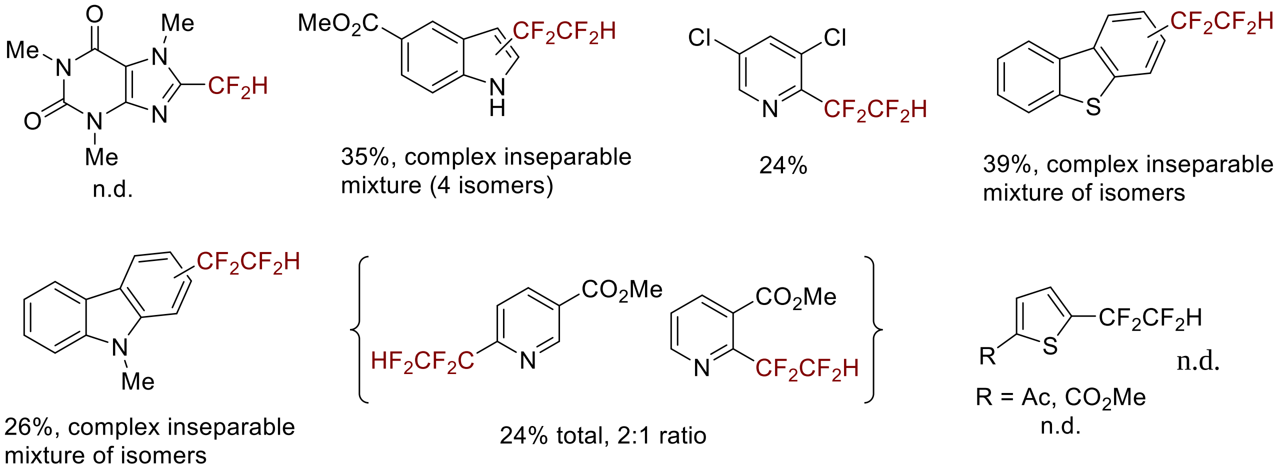


## Gram-scale experiment

A solid mixture of caffeine (1.94 g, 10 mmol), $LiClO_4$ (1.06 g, 10 mmol), and $Fe(ClO_4)_3{\cdot}10H_2O$ (1.07 g, 2 mmol, 20 mol%) was added to a 250 mL Schlenk tube for electrochemical reaction equipped with a large stirring bar, Pt cathode (50 × 25 × 0.25 mm) and graphite felt (GF) anode (50 × 25 × 7.5 mm). The tube was evacuated and backfilled with nitrogen three times. Then, a solution of 3*H*-tetrafluoropropanoic acid (4.60 g, 30 mmol, 3.0 equiv.) in 80 mL MeCN was added under positive pressure of nitrogen. The resulting mixture was intensively stirred (500 rpm) under 390 nm light and applied constant current electrolysis 160 mA for 27 hours. Then, it was exposed to air, poured into a 500 mL separatory funnel with 200 ml EtOAc, and washed with 75 mL of saturated $NaHCO_3$ solution to remove iron residues. Organic layer was separated, and aqueous layer was extracted twice with 50 mL EtOAc. Combined organic layers were dried over anhydrous $Na_2SO_4$, evaporated under reduced pressure and the crude product was purified by column chromatography on silica gel (hexane/EtOAc 3:1 to 2:1) to give product **7** (1.52 g, 52%) as a white solid.

## Kolbe dimerization of $PhCF_2CO_2H$

A 15 mL Schlenk tube (undivided electrochemical cell) charged with a solid mixture of $PhCF_2CO_2H$ (129 mg, 0.75 mmol), $LiClO_4$ (64 mg, 0.6 mmol), $Fe(ClO_4)_3{\cdot}10H_2O$ (32 mg, 0.06 mmol, 10 mol%) equipped with a stirring bar (ca. 9 × 2 × 2 mm), Pt cathode (25 × 10 × 0.25 mm) and a glassy carbon (GC) anode (25 × 10 × 1.5 mm) was evacuated and backfilled with nitrogen three times. Then DMSO/water mixture (6:1, 3 mL) was added under positive pressure of nitrogen, and the mixture was intensively stirred (1000 rpm) under 390 nm light and constant current electrolysis at 4 mA for 12 h. Then, the mixture was exposed to air, diluted with 30 mL EtOAc, and washed with 5 mL of 10% $Na_2CO_3$ solution to remove iron residues. The aqueous phase was extracted with EtOAc, combined organic layer was separated, dried by anhydrous $Na_2SO_4$, evaporated under reduced pressure and the crude product was purified by column chromatography on silica gel (hexane) to give **1,1,2,2-tetrafluoro-1,2-diphenylethane 3'** (53 mg, 56%) as a white solid.

**$^1$H NMR** (300 MHz, $CDCl_3$) δ 7.53 – 7.38 (m, 5H); **$^{13}$C NMR** (75 MHz, $CDCl_3$) δ 131.0, 128.2, 127.1 (dt, $J$ = 7.9, 3.8 Hz), 116.8 (tt, $J$ = 253.0, 36.4 Hz, $CF_2$) ; **$^{19}$F NMR** (282 MHz, $CDCl_3$) δ -111.8 (s); **IR** (ATR): $\tilde{\nu}$ =

2927, 1449, 1258, 1124, 1081, 1068, 877, 752, 697, 658 cm$^{-1}$; **HRMS** (EI): *m/z* calcd. for $C_{14}H_{10}F_4^+$: 254.0713 $[M]^+$, found 254.0715.

# Radical trap experiments

## I. Trapping experiments with TEMPO

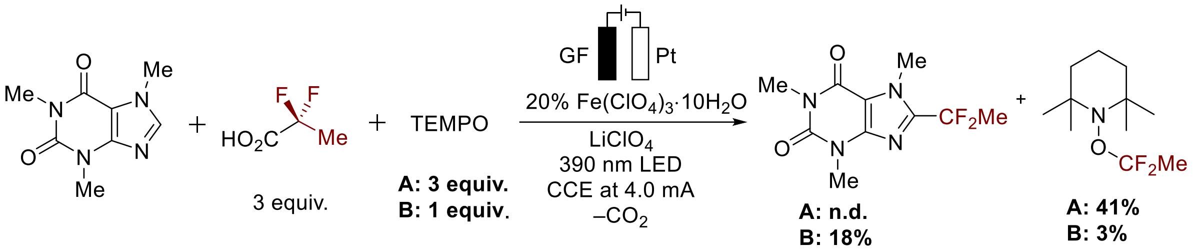


A 15 mL Schlenk tube (undivided electrochemical cell) charged with a solid mixture of caffeine (58.5 mg, 0.3 mmol), 2,2-difluoropropionic acid (3 equiv.), $LiClO_4$ (32 mg, 0.3 mmol), $Fe(ClO_4)_3 \cdot 10H_2O$ (32 mg, 0.06 mmol, 20 mol%) equipped with a stirring bar (ca. 9 × 2 × 2 mm), Pt cathode (25 × 10 × 0.25 mm) and a glassy carbon (GC) anode (25 × 10 × 1.5 mm) was evacuated and backfilled with nitrogen three times. Then, a solution of TEMPO (1.0 or 3.0 equiv. respectively) in MeCN (3 mL) was added under positive pressure of nitrogen, and the mixture was intensively stirred (1000 rpm) under 390 nm light and constant current electrolysis at 4 mA for 12 h. After the completion of the reaction, it was diluted with EtOAc, washed with 5 mL of 10% $Na_2CO_3$ solution to remove iron residues. The aqueous phase was extracted with EtOAc, combined organic layers were dried by anhydrous $Na_2SO_4$, evaporated and the residue was analyzed by $^{19}F$ NMR (using $PhCF_3$ as internal standard) and HRMS [yields determined by NMR are shown on the scheme]. TEMPO-$CF_2$Me adduct was detected by both NMR and HRMS.

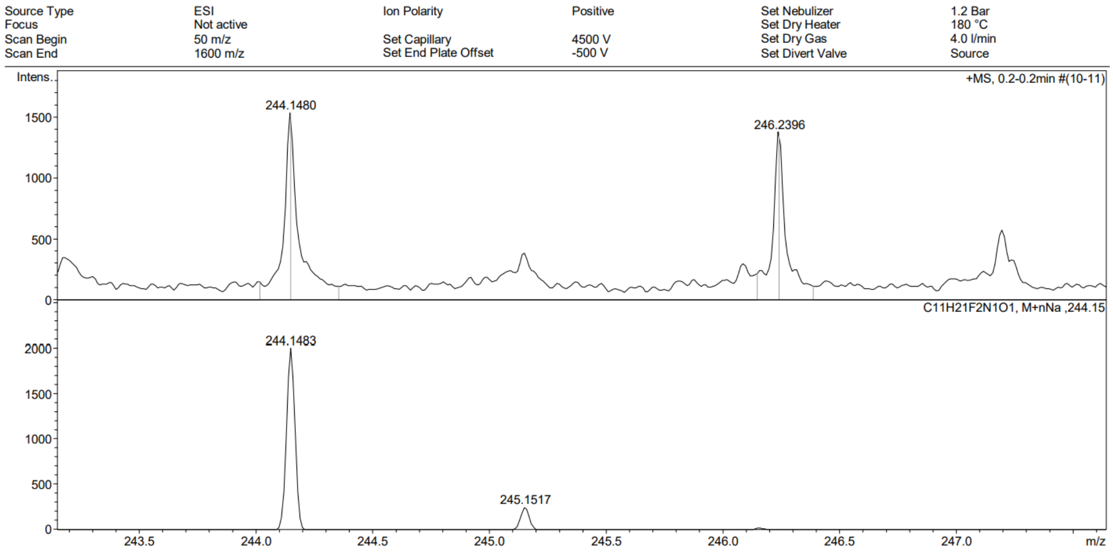


## II. Trapping experiment with DMPO

A 15 mL Schlenk tube (undivided electrochemical cell) charged with a solid mixture of caffeine (58.5 mg, 0.3 mmol), 2,2-difluoropropionic acid (3 equiv.), $LiClO_4$ (32 mg, 0.3 mmol), $Fe(ClO_4)_3 \cdot 10H_2O$ (32 mg, 0.06 mmol, 20 mol%) equipped with a stirring bar (ca. 9 × 2 × 2 mm), Pt cathode (25 × 10 × 0.25 mm) and a glassy carbon (GC) anode (25 × 10 × 1.5 mm) was evacuated and backfilled with nitrogen three times. Then, a solution of 5,5-dimethyl-1-pyrroline-*N*-oxide (1.0 or 3.0 equiv. respectively) in MeCN (3 mL) was added under positive pressure of nitrogen, and the mixture was intensively stirred (1000 rpm) under 390 nm light and constant current electrolysis at 4 mA for 12 h. After the completion of the reaction, it was diluted with EtOAc, washed with 5 mL of 10% $Na_2CO_3$ solution to remove iron residues. The aqueous phase was extracted with EtOAc, combined organic layers were dried by anhydrous $Na_2SO_4$, evaporated and the residue was analyzed by $^{19}$F NMR using $PhCF_3$ as internal standard. No formation of the fluoroalkylation product **3** was observed.

## On/off experiments

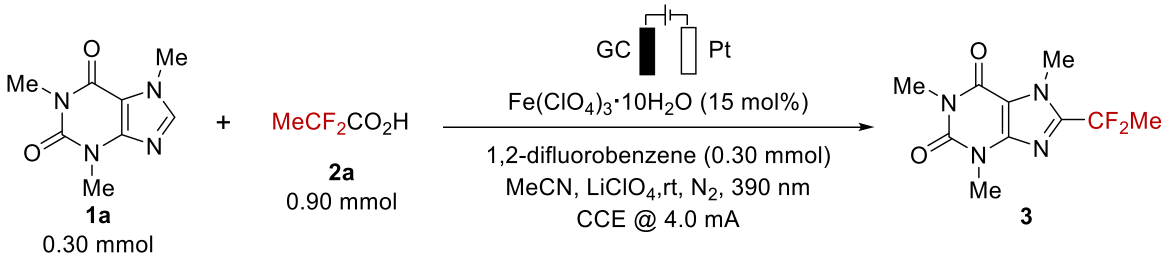


Into a 15 mL Schlenk tube (undivided electrochemical cell) charged with a solid mixture of caffeine **1a** (77.7 mg, 0.4 mmol, 1 eq.), $LiClO_4$ (32 mg, 0.3 mmol), $Fe(ClO_4)_3 \cdot 10H_2O$ (32 mg, 0.04 mmol, 15 mol.%), and 2,2-difluoropropanoic acid **2a** (99 mg, 0.9 mmol, 3 equiv.), equipped with a stirring bar (ca. 9 × 2 × 2 mm), platinum (Pt) cathode (25 × 10 × 0.25 mm) and a glassy carbon (GC) anode (25 × 10 × 1.5 mm), a solution of 1,4-difluorobenzene (30 μL) in MeCN (4 mL) was added under positive pressure of nitrogen. The reaction vessel was sealed with a rubber septum, and the resulting mixture was stirred under 390 nm irradiation and CCE at 4.0 mA. As indicated below, once per hour the current or light was turned on or off, respectively. After every interval, aliquots of 0.1 mL were taken via syringe under nitrogen, diluted with $CDCl_3$ (0.6 mL) and the resulting solution was analyzed by $^{19}$F NMR spectroscopy.

### A. Light On/Off

| Time (min) | On/Off | Yield (%) |
|---|---|---|
| 0 | Off | 0 |
| 60 | On | 28 |
| 120 | Off | 27 |
| 180 | On | 53 |

| | | |
|---|---|---|
| 240 | Off | 52 |
| 300 | On | 69 |
| 360 | Off | 74 |

**B. Electricity On/Off**

| Time (min) | On/Off | Yield (%) |
|---|---|---|
| 0 | Off | 0 |
| 60 | Off | 7 |
| 120 | On | 18 |
| 180 | Off | 19 |
| 240 | On | 32 |
| 300 | Off | 32 |
| 360 | On | 45 |

## Cyclic voltammetry, UV/Vis and spectroelectrochemistry measurements

Cyclic voltammetry

Cyclic voltammetry was carried out with a Metrohm Autolab PGSTAT204 potentiostat and Nova 2.1 software. A glassy carbon disk (diameter: 3 mm), a coiled platinum wire counter electrode and a saturated calomel (SCE) reference electrode were employed. The voltammograms were recorded at room temperature in MeCN (3.0 mL) with 0.1 M $LiClO_4$ as supporting electrolyte under $N_2$ atmosphere. The solution was degassed by nitrogen gas bubbling for 5 minutes before each measurement. The scan rate was set to 100 mV/s. Deviations from the general experimental conditions are indicated in the respective figures and descriptions.

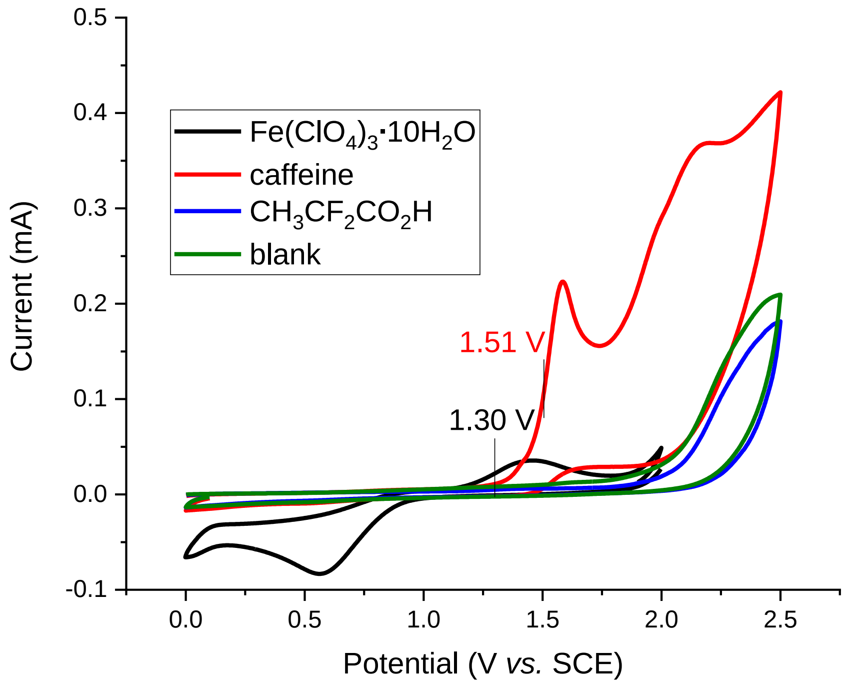


**Fig. S3**. Cyclic voltammogram of each reaction components. $Fe(ClO_4)_3{\cdot}10H_2O$ (10 mM), caffeine (10 mM), $CH_3CF_2CO_2H$ (30 mM). 0.1 M $LiClO_4$ in MeCN, 100 mV/s, $N_2$, room temperature.

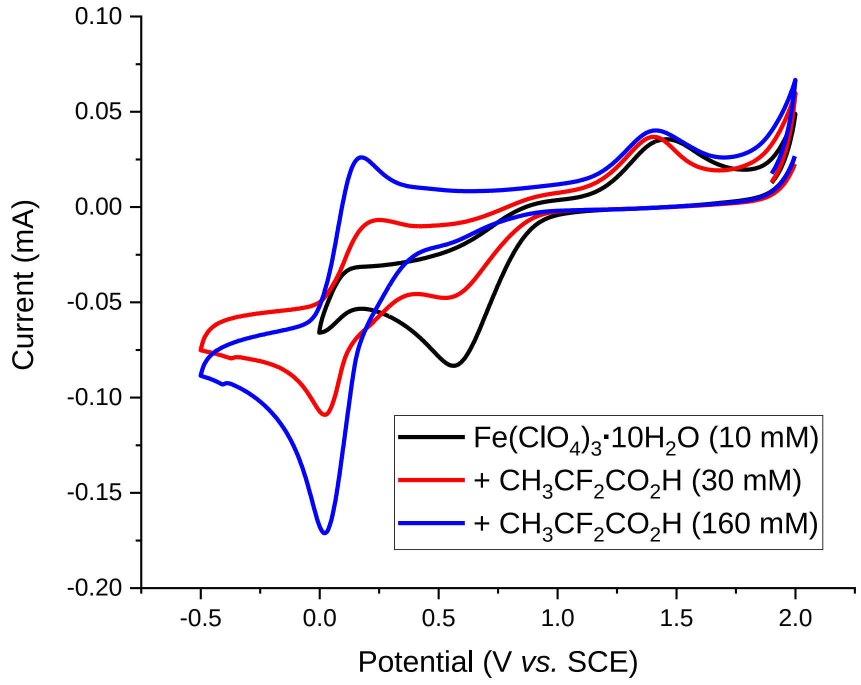


**Fig. S4**. Cyclic voltammogram of $Fe(ClO_4)_3{\cdot}10H_2O$ (10 mM) titrated with $CH_3CF_2CO_2H$ (0–160 mM). $LiClO_4$ (0.1 M) in MeCN, 100 mV/s, $N_2$, room temperature.

UV/Vis spectra of reaction components in MeCN

UV/Vis absorption spectroscopy was performed on a Jasco Spectrophotometer V-770. For determination of the absorption, a stock solution of $Fe(ClO_4)_3 \cdot 10H_2O$ in MeCN (0.2 mM) has been treated with varying equivalents of 2,2-difluorpropanoic acid or caffeine, respectively, as specified in the following. The sample solutions were filled in a quartz cuvette with a path length of 10 mm.

$\lambda_{max}$ ($Fe(ClO_4)_3$ in MeCN) = 360 nm, which slightly shifts to the lower wavelength region upon complexation (Fig. 5).

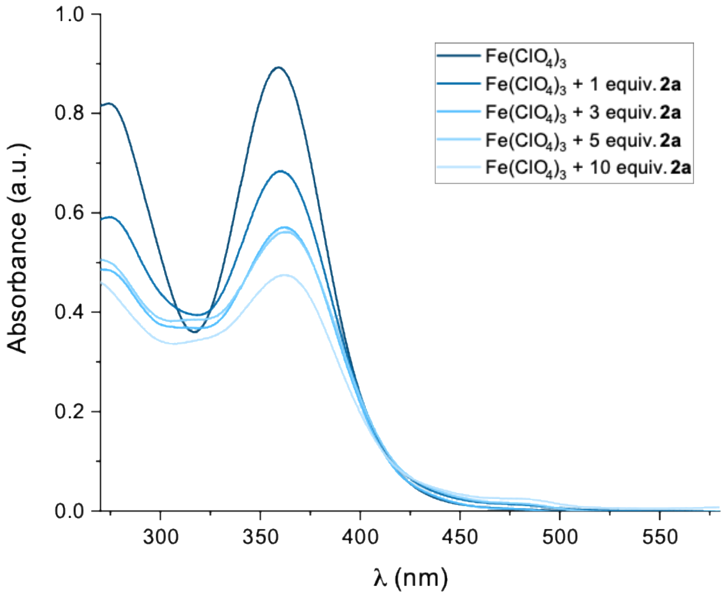


**Fig. S5**. UV/Vis absorption spectra of $Fe(ClO_4)_3 \cdot 10H_2O$ and varying amount of **2a** in MeCN.

*Comment: 2,2-difluorpropanoic acid* ***2a*** *showed no absorbance in the analyzed spectral window.*

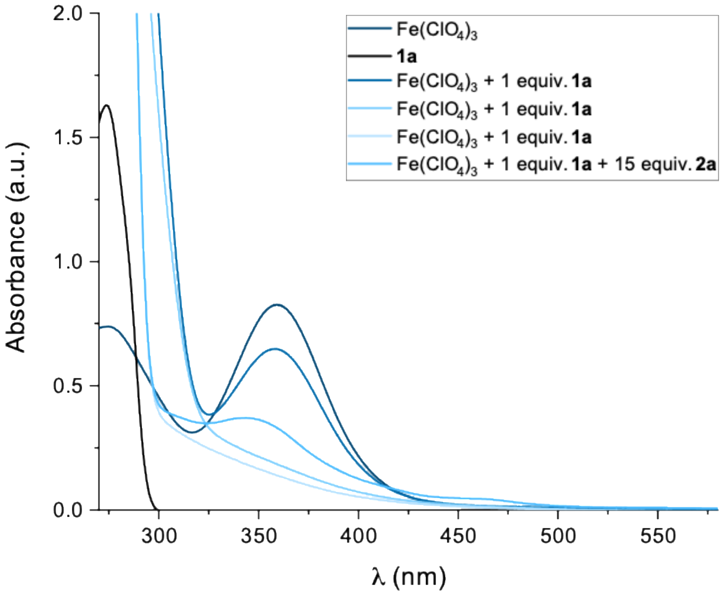


**Fig. S6**. UV/Vis absorption spectra of reagents and iron salt in MeCN.

## ICP-MS analysis of iron(III) perchlorate decahydrate

The iron(III) perchlorate hydrate sample material (10-15 mg) was ultra-sonicated in a mixture of 0.2 mL conc. HCl, and 0.6 mL conc. $HNO_3$; trace metal grade for 5 h at 50 °C. Then, the sample was diluted to 10 mL with water (LC-MS grade) and sonicated for 10 min at rt. The obtained sample was centrifuged for 20 min at 8000 rpm, decanted in a new vial, and measured on a Thermo Scientific iCAP RQ. For the analysis of the reaction mixture, a typical batch was evaporated to dryness and was used as the sample material (ca. 10 mg).

| **Metal** | **Co** | **Ni** | **Cu** | **Ru** | **Rh** | **Pd** |
|---|---|---|---|---|---|---|
| **Content** | 0.976 ppm | 11.94 ppm | 18.72 ppm | 0.223 ppm | 1 ppb | 48 ppb |

## Gas evolution study

Following the general procedure 1, caffeine **1a** (77.7 mg, 0.4 mmol, 1.0 equiv.) and 2,2-difluoropropanoic acid (132 mg, 1.2 mmol, 3.0 equiv.) were used. The change in volume of the headspace was monitored maintaining isobaric conditions using the GasMess-System (LIKAT Rostock and MesSen Nord GmbH).

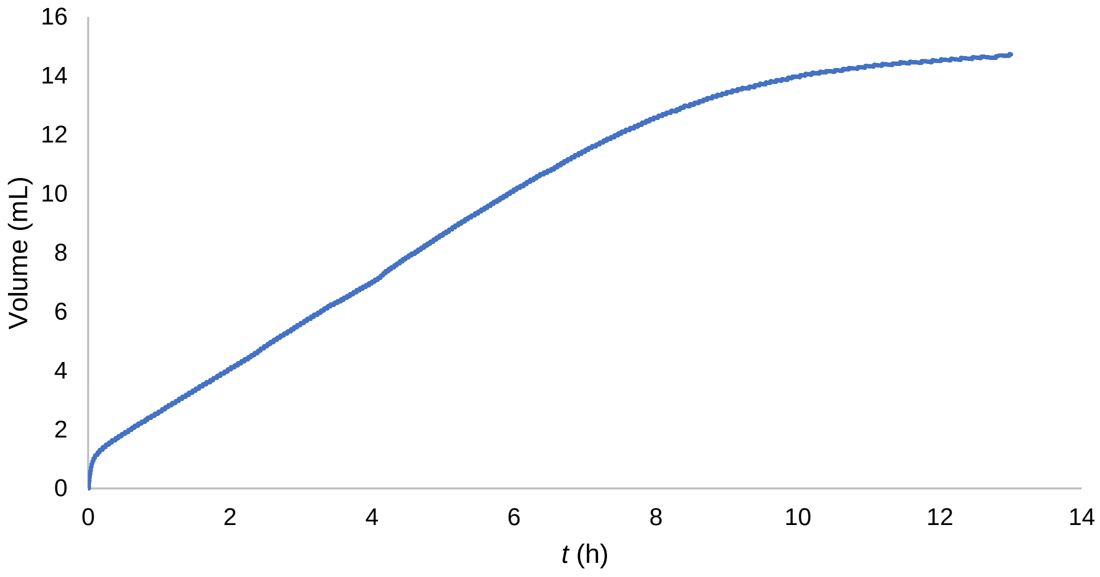


**Fig. S7**. Volume increase curve for the model reaction.

In a parallel experiment, after completion of the reaction, a sample of the headspace (1 mL) was carefully collected using a gas syringe. The sample was directly analyzed via gas chromatography. The peak observed at a retention time of 1.56 min was assigned to $H_2$, as confirmed by the comparison with pure hydrogen gas as reference sample (Fig. S8).

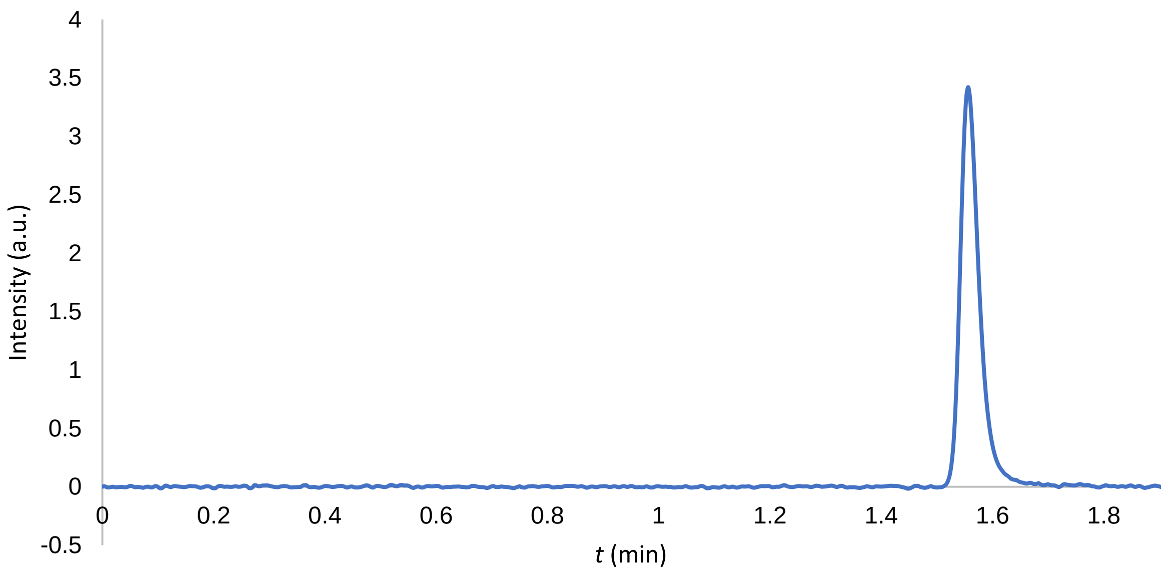


**Fig. S8**. Detection of hydrogen by GC headspace analysis.

# Kinetic analysis

## A. Variation of current

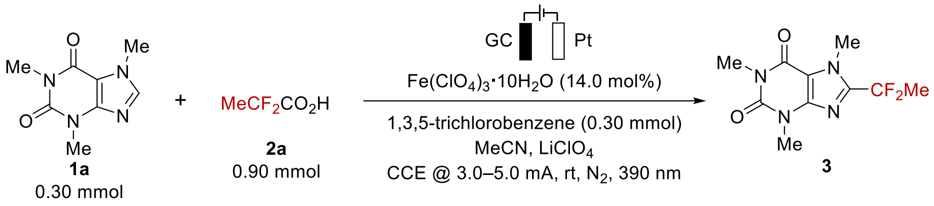


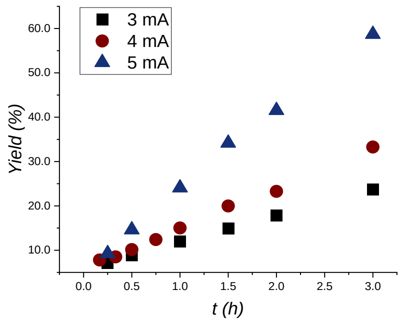


**Fig. S9**. Reaction profiles with various currents.

Into a 15 mL Schlenk tube (undivided electrochemical cell) charged with a solid mixture of caffeine **1a** (58.5 mg, 0.3 mmol), $LiClO_4$ (0.1 M), $Fe(ClO_4)_3 \cdot 10H_2O$ (22.4 mg, 0.04 mmol, 14 mol%), 2,2-difluoropropanoic acid **2a** (0.9 mmol), equipped with a stirring bar (ca. 12.5 × 7 × 7 mm), platinum (Pt) cathode (25 × 10 × 0.25 mm) and a glassy carbon (GC) anode (25 × 10 × 1.5 mm), a solution of 1,3,5-trichlorobenzene (0.9 mmol) in MeCN (6 mL) was added under positive pressure of nitrogen. The reaction mixture was stirred (500 rpm) under 390 nm irradiation and CCE at 3.0-5.0 mA. At specific intervals shown on the horizontal axis, a small aliquot of the reaction mixture was taken, filtered through a small silica gel pad and analyzed by GC. To obtain a reliable result, data were generated by using the same potentiostat, stirring plate, and GC. The position of the reactor and the orientation of the electrode placement were strictly controlled for exact reproducibility of reaction times. Yields were determined by GC analysis with calibration.

## B. Variation of caffeine (**1a**) concentration

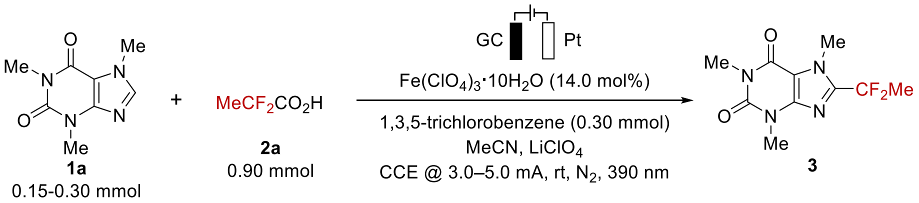

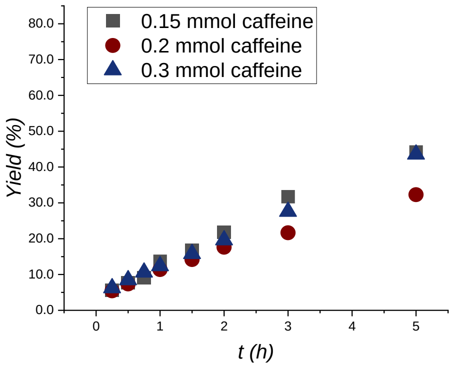


**Fig. S10**. Reaction profiles with various concentrations of caffeine **1a**.

A 15 mL Schlenk tube (undivided electrochemical cell) charged with a solid mixture of caffeine **1a** (0.15-0.3 mmol), $LiClO_4$ (0.1 M), $Fe(ClO_4)_3{\cdot}10H_2O$ (22.4 mg, 0.04 mmol, 14 mol%), 2,2-difluoropropanoic acid **2a** (0.9 mmol), equipped with a stirring bar (ca. 12.5 × 7 × 7 mm), platinum (Pt) cathode (25 × 10 × 0.25 mm) and a glassy carbon (GC) anode (25 × 10 × 1.5 mm) was evacuated and backfilled with nitrogen three times. Then a solution of 1,3,5-trichlorobenzene (0.9 mmol) in MeCN (6 mL) was added under positive pressure of nitrogen. The reaction mixture was stirred at 500 rpm under 390 nm irradiation and CCE at 3.0-5.0 mA. At specific intervals shown on the horizontal axis, a small aliquot of the reaction mixture was taken, filtered through a small silica gel pad and analyzed by GC. To obtain a reliable result, data were generated by using the same potentiostat, stirring plate, and GC. The position of the reactor and the orientation of the electrode placement were strictly controlled for exact reproducibility of reaction times. Yields were determined by GC analysis with calibration.

<u>C. Variation of carboxylic acid (**2a**) concentration</u>

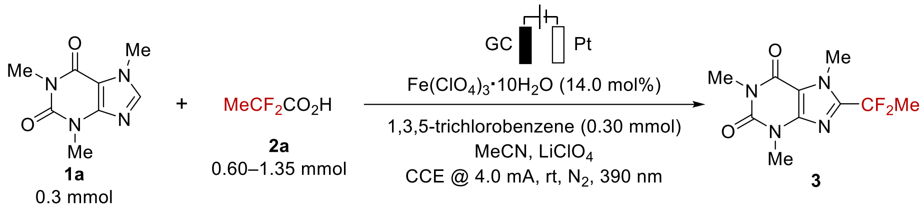


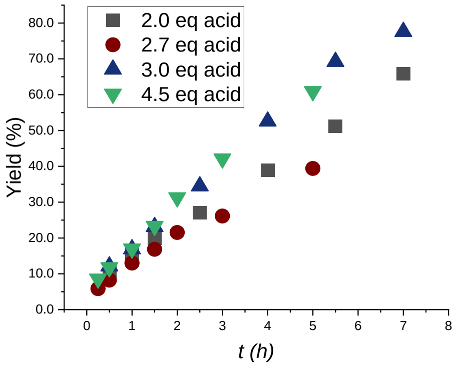


**Fig. S11**. Reaction profiles with various concentrations of acid **2a**.

Into a 15 mL Schlenk tube (undivided electrochemical cell) charged with a solid mixture of caffeine **1a** (58.5 mg, 0.3 mmol), $LiClO_4$ (0.1 M), $Fe(ClO_4)_3·10H_2O$ (22.4 mg, 0.04 mmol, 14 mol%), 2,2-difluoropropanoic acid **2a** (0.6-1.35 mmol), equipped with a stirring bar (ca. 12.5 × 7 × 7 mm), platinum (Pt) cathode (25 × 10 × 0.25 mm) and a glassy carbon (GC) anode (25 × 10 × 1.5 mm), a solution of 1,3,5-trichlorobenzene (0.9 mmol) in MeCN (6 mL) was added under positive pressure of nitrogen. The reaction mixture was stirred at 500 rpm under 390 nm irradiation and CCE at 3.0-5.0 mA. At specific intervals shown on the horizontal axis, a small aliquot of the reaction mixture was taken, filtered through a small silica gel pad and analyzed by GC. To obtain a reliable result, data were generated by using the same potentiostat, stirring plate, and GC. The position of the reactor and the orientation of the electrode placement were strictly controlled for exact reproducibility of reaction times. Yields were determined by GC analysis with calibration.

D. Variation of [Fe] catalyst concentration

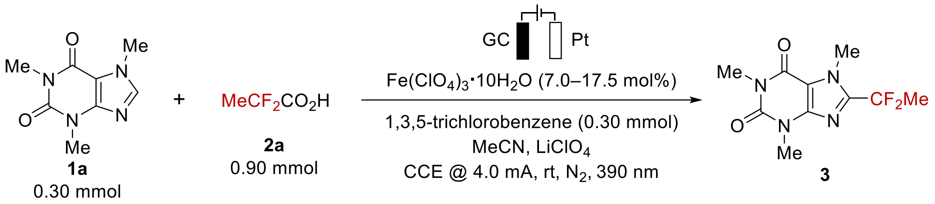


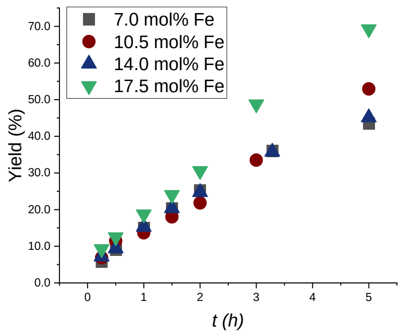


**Figure S12**. Reaction profiles with various catalyst loadings.

Into a 15 mL Schlenk tube (undivided electrochemical cell) charged with a solid mixture of caffeine **1a** (58.5 mg, 0.3 mmol), $LiClO_4$ (0.1 M), $Fe(ClO_4)_3·10H_2O$ (7.0-17.5 mol%), 2,2-difluoropropanoic acid **2a** (0.9 mmol), equipped with a stirring bar (ca. 12.5 × 7 × 7 mm), platinum (Pt) cathode (25 × 10 × 0.25 mm) and a glassy carbon (GC) anode (25 × 10 × 1.5 mm), a solution of 1,3,5-trichlorobenzene (0.9 mmol) in MeCN (6 mL) was added under positive pressure of nitrogen. The reaction mixture was stirred at 500 rpm under 390 nm irradiation and CCE at 3.0-5.0 mA. At specific intervals shown on the horizontal axis, a small aliquot of the reaction mixture was taken, filtered through a small silica gel pad and analyzed by GC. To obtain a reliable result, data were generated by using the same potentiostat, stirring plate, and GC. The position of the reactor and the orientation of the electrode placement were strictly controlled for exact reproducibility of reaction times. Yields were determined by GC analysis with calibration.

Although higher concentration of iron(III) salt gave higher initial rate, the rate was determined by the current after one turnover.

**Conclusions:** Zero order by all components (caffeine, acid, iron(III) salt). Anodic reoxidation of iron(II) to iron(III) is identified the rate-determining factor. This can explain the following observations: 1) Stirring rate influences the reaction rate. Heterogeneous electrode process is more sensitive to diffusion. 2) The reaction rate at the beginning of the reaction is the fastest.

## Computation studies

All calculation were performed using Gaussian 16, Revision A.03 package.[54] All structures were optimized at the ωB97X-D[55] level of theory with a def2-TZVPP basis set.[56] Analytical frequencies were carried out at the same level of theory in order to identify each stationary point as an intermediate (absence of imaginary frequencies). These also provided thermal and non-thermal corrections to the Gibbs free energy at 298.15 K. The electronic energy was then refined through ωB97X-D[57] single-point calculations on the optimized geometries in combination with a def2-QZVPP basis set.[56] Solvent effects were included implicitly in the single points calculation through the use of the SMD model.[57] In the latter, parameters for acetonitrile, octanol or water were used as implemented in Gaussian 16. Herein, the reported energies are based on gas-phase Gibbs free energies with a def2-TZVPP basis set for which the electronic energies were refined to ωB97X-D with a def2-QZVPP basis set and solvent effects. Nucleophilicity was determined according to [58] taking malononitrile radical as reference, whereas lipophilicity was obtained according to [59].

**Table S2**. Calculated HOMO ($E_{HOMO}$) and LUMO ($E_{LUMO}$) energies at the ωB97X-D+SMD(Acetonitrile)/def2-QZVPP//ωB97X-D/def2-TZVPP level of theory used in the nucleophilicity determination (all in Hartree).

| Structure | $E_{HOMO}$ | $E_{LUMO}$ |
|---|---|---|
| **$CF_3CF_2CF_2^•$** | -0.341770 | -0.027740 |
| **$CHF_2CF_2^•$** | -0.330560 | -0.014880 |
| **$CF_3CF_2^•$** | -0.345840 | -0.024140 |
| **$ClCF_2^•$** | -0.334210 | -0.021630 |
| **$EtCF_2^•$** | -0.298480 | 0.016620 |
| **$CF_3^•$** | -0.352600 | -0.016710 |
| **$CHF_2^•$** | -0.313560 | 0.012190 |
| **$MeCF_2^•$** | -0.299850 | 0.016700 |
| **$PhCF_2^•$** | -0.247610 | -0.011850 |
| **Malononitrile$^•$** | -0.359860 | -0.097270 |

**Table S3**. Calculated electronic energies ($E_{el}$) and Gibbs free energies (DG) at the ωB97X-D+SMD (Octanol or Water)/def2-QZVPP//ωB97X-D/def2-TZVPP level of theory used in the lipophilicity determination (all in Hartree).[a]

| Structure | $E_{el,O}$ | $DG_O$ | $E_{el,W}$ | $DG_W$ |
|---|---|---|---|---|
| **$CF_3CF_2CF_2^•$** | -813.316433 | -813.315094 | -813.310952 | -813.309613 |
| **$CHF_2CF_2^•$** | -476.211222 | -476.208919 | -476.207762 | -476.205459 |
| **$CF_3CF_2^•$** | -575.479616 | -575.486370 | -575.475315 | -575.482069 |
| **$ClCF_2^•$** | -697.992679 | -698.009941 | -697.988965 | -698.006227 |
| **$EtCF_2^•$** | -317.020901 | -316.974078 | -317.016515 | -316.969692 |
| **$CF_3^•$** | -337.634842 | -337.649236 | -337.632179 | -337.646573 |
| **$CHF_2^•$** | -238.366806 | -238.372552 | -238.364863 | -238.370609 |
| **$MeCF_2^•$** | -277.701504 | -277.681461 | -277.698180 | -277.678137 |
| **$PhCF_2^•$** | -469.454051 | -469.386622 | -469.448248 | -469.380819 |

[a] Subscripts O and W correspond to the octanol and water respectively.

**Cartesian coordinates of the optimized structures**

**$CF_3CF_2CF_2^•$**

Lowest frequency = 36.4373 $cm^{-1}$

Charge = 0, Multiplicity = 2

10

| | | | |
|---|---|---|---|
| C | -1.328363 | -2.227825 | 1.766850 |
| C | -0.574884 | -0.920855 | 1.583717 |
| C | -0.318526 | -0.486482 | 0.107329 |
| F | -2.579849 | -2.222664 | 1.371533 |
| F | -0.692848 | -3.309287 | 1.380760 |
| F | -1.278817 | 0.055277 | 2.175392 |
| F | 0.618917 | -1.037682 | 2.183378 |
| F | 0.342922 | 0.661525 | 0.078544 |
| F | -1.473269 | -0.332605 | -0.530623 |
| F | 0.397876 | -1.410506 | -0.522801 |

**$CHF_2CF_2^{\bullet}$**

Lowest frequency = 66.5645 $cm^{-1}$

Charge = 0, Multiplicity = 2

7

| | | | |
|---|---|---|---|
| C | -1.327824 | -2.222999 | 1.793627 |
| C | -0.577139 | -0.924703 | 1.577437 |
| F | -2.570691 | -2.204101 | 1.353568 |
| F | -0.685845 | -3.291873 | 1.364659 |
| H | -0.439084 | -0.695970 | 0.513702 |
| F | 0.633021 | -1.017207 | 2.158339 |
| F | -1.267440 | 0.079642 | 2.147602 |

**$CF_3CF_2^{\bullet}$**

Lowest frequency = 65.1603 $cm^{-1}$

Charge = 0, Multiplicity = 2

7

| | | | |
|---|---|---|---|
| C | -1.203485 | -2.167471 | 1.785856 |
| F | -2.514542 | -2.103742 | 1.750192 |
| F | -0.737139 | -3.155362 | 1.057183 |
| C | -0.501847 | -0.836953 | 1.562992 |
| F | -0.959920 | 0.061288 | 2.426181 |
| F | 0.805774 | -0.984609 | 1.736121 |
| F | -0.707850 | -0.369206 | 0.326434 |

**$ClCF_2^{\bullet}$**

Lowest frequency = 368.7214 cm$^{-1}$

Charge = 0, Multiplicity = 2

4

| | | | |
|---|---|---|---|
| C | -1.297058 | -2.173341 | 1.229050 |
| F | -2.551309 | -2.186404 | 1.622899 |
| F | -0.679740 | -3.266558 | 1.619298 |
| Cl | -0.453927 | -0.713826 | 1.611585 |

**$EtCF_2^{\bullet}$**

Lowest frequency = 92.5366 cm$^{-1}$

Charge = 0, Multiplicity = 2

10

| | | | |
|---|---|---|---|
| C | -1.308062 | -2.183531 | 1.753076 |
| C | -0.562836 | -0.901860 | 1.594022 |
| C | -0.331735 | -0.515086 | 0.128375 |
| H | 0.388334 | -1.008690 | 2.115462 |
| H | -1.130001 | -0.126475 | 2.108980 |
| H | 0.214968 | 0.425344 | 0.071852 |
| H | 0.248738 | -1.277972 | -0.388848 |
| H | -1.278565 | -0.390499 | -0.395500 |
| F | -2.562098 | -2.180981 | 1.299278 |
| F | -0.681613 | -3.273237 | 1.307342 |

**$CF_3^{\bullet}$**

Lowest frequency = 513.6813 cm$^{-1}$

Charge = 0, Multiplicity = 2

4

| | | | |
|---|---|---|---|
| C | -1.290034 | -2.161494 | 1.224842 |
| F | -2.543944 | -2.163658 | 1.617585 |
| F | -0.664042 | -3.249318 | 1.613801 |
| F | -0.663796 | -1.078478 | 1.626604 |

**$CHF_2^{\bullet}$**

Lowest frequency = 557.3242 $cm^{-1}$

Charge = 0, Multiplicity = 2

4

| | | | |
|---|---|---|---|
| C | -1.281102 | -2.146435 | 1.277813 |
| F | -2.564259 | -2.187110 | 1.590530 |
| F | -0.674452 | -3.278923 | 1.586665 |
| H | -0.764780 | -1.254113 | 1.627825 |

**$MeCF_2^{\bullet}$**

Lowest frequency = 194.1401 $cm^{-1}$

Charge = 0, Multiplicity = 2

7

| | | | |
|---|---|---|---|
| C | -1.312228 | -2.195222 | 1.789835 |
| C | -0.576798 | -0.923569 | 1.571827 |
| H | 0.407036 | -0.985657 | 2.031196 |
| H | -1.128642 | -0.099221 | 2.017695 |
| F | -2.572200 | -2.209778 | 1.358629 |
| F | -0.688181 | -3.296339 | 1.375465 |
| H | -0.455848 | -0.729930 | 0.500304 |

**$PhCF_2^{\bullet}$**

Lowest frequency = 117.7002 $cm^{-1}$

Charge = 0, Multiplicity = 2

14

| | | | |
|---|---|---|---|
| C | -1.282731 | -2.149983 | 1.525277 |
| F | -2.593460 | -2.249382 | 1.522960 |
| F | -0.709545 | -3.332925 | 1.528170 |
| C | -0.591553 | -0.948309 | 1.522224 |
| C | 0.826813 | -0.929245 | 1.523968 |
| C | -1.288244 | 0.287302 | 1.518054 |
| C | 1.498767 | 0.271361 | 1.521656 |
| H | 1.372963 | -1.861704 | 1.527069 |
| C | -0.588304 | 1.471810 | 1.515821 |
| H | -2.368871 | 0.290542 | 1.516607 |
| C | 0.805229 | 1.480076 | 1.517618 |
| H | 2.580784 | 0.272110 | 1.522976 |
| H | -1.131706 | 2.407474 | 1.512598 |
| H | 1.344224 | 2.417151 | 1.515843 |

**Malononitrile$^{\bullet}$**

Lowest frequency = 153.4059 $cm^{-1}$

Charge = 0, Multiplicity = 2

6

| | | | |
|---|---|---|---|
| C | 0.097915 | -0.208537 | 0.302171 |
| H | 0.684048 | 0.618973 | 0.677924 |
| C | -1.264000 | -0.023852 | 0.066014 |

| | | | |
|---|---|---|---|
| N | -2.390684 | 0.156802 | -0.119842 |
| C | 0.726045 | -1.431384 | 0.067002 |
| N | 1.272460 | -2.432960 | -0.119625 |

## X-ray crystallography

A colorless, plate-shaped crystal of **3** was mounted on a MiTeGen micromount with perfluoroether oil. Data were collected from a shock-cooled single crystal at 100.00 K on a Bruker D8 VENTURE dual wavelength Mo/Cu four-circle diffractometer with a microfocus sealed X-ray tube using a mirror optics as monochromator and a Bruker PHOTON III detector. The diffractometer was equipped with an Oxford Cryostream 800 low temperature device and used Mo$K_\alpha$ radiation ($\lambda$ = 0.71073 Å). All data were integrated with SAINT and a numerical absorption correction using SADABS was applied.[60,61] The structure was solved by dual methods using XT and refined by full-matrix least-squares methods against $F^2$ by XL using Olex2.[62-64] All non-hydrogen atoms were refined with anisotropic displacement parameters. All hydrogen atoms were refined isotropic on calculated positions using a riding model with their $U_{iso}$ values constrained to 1.5 times the $U_{eq}$ of their pivot atoms for terminal sp$^3$ carbon atoms and 1.2 times for all other carbon atoms. Crystallographic data for the structures reported here have been deposited with the Cambridge Crystallographic Data Centre.[65] CCDC 2342104 contains the supplementary crystallographic data for this paper. These data can be obtained free of charge from The Cambridge Crystallographic Data Centre via www.ccdc.cam.ac.uk/structures. This report and the CIF file were generated using FinalCif.

**Table S4.** Crystal data and structure refinement for **3.**

| | |
|---|---|
| CCDC number | 2342104 |
| Empirical formula | $C_{10}H_{12}F_2N_4O_2$ |
| Formula weight | 258.24 |
| Temperature [K] | 100.00 |
| Crystal system | triclinic |
| Space group (number) | $P\bar{1}$ (2) |
| *a* [Å] | 8.2296(10) |
| *b* [Å] | 8.3634(8) |
| *c* [Å] | 8.7249(11) |
| α [°] | 112.480(5) |
| β [°] | 99.395(5) |
| γ [°] | 95.538(5) |
| Volume [Å$^3$] | 539.06(11) |
| *Z* | 2 |
| $\rho_{calc}$ [gcm$^{-3}$] | 1.591 |

| | |
|---|---|
| $\mu$ [mm$^{-1}$] | 0.137 |
| $F$(000) | 268 |
| Crystal size [mm$^3$] | 1.001×0.442×0.076 |
| Crystal colour | colorless |
| Crystal shape | plate |
| Radiation | Mo$K_\alpha$ (λ=0.71073 Å) |
| 2θ range [°] | 5.10 to 70.11 (0.62 Å) |
| Index ranges | −13 ≤ h ≤ 12<br>−13 ≤ k ≤ 10<br>−14 ≤ l ≤ 14 |
| Reflections collected | 35432 |
| Independent reflections | 4458<br>$R_{int}$ = 0.0304<br>$R_{sigma}$ = 0.0164 |
| Completeness to<br>θ = 25.242° | 100.0 % |
| Data / Restraints / Parameters | 4458/0/167 |
| Absorption correction<br>$T_{min}/T_{max}$ (method) | 0.8386/1.0000<br>(numerical) |
| Goodness-of-fit on $F^2$ | 1.044 |
| Final $R$ indexes<br>[$I$≥2σ($I$)] | $R_1$ = 0.0350<br>w$R_2$ = 0.1011 |
| Final $R$ indexes<br>[all data] | $R_1$ = 0.0377<br>w$R_2$ = 0.1042 |
| Largest peak/hole [eÅ$^{-3}$] | 0.62/−0.29 |

**Table S5.** Atomic coordinates and $U_{eq}$ [Å$^2$] for **3.**

| Atom | *x* | *y* | *z* | $U_{eq}$ |
|---|---|---|---|---|
| F1 | 0.82878(6) | 0.56727(6) | 0.95094(5) | 0.01890(10) |
| F2 | 0.76966(6) | 0.74902(6) | 0.83489(6) | 0.01932(10) |
| O1 | 0.16321(6) | 0.21006(7) | 0.53036(7) | 0.01909(11) |
| O2 | 0.37111(7) | -0.17412(7) | 0.10484(7) | 0.01913(11) |
| N1 | 0.52503(7) | 0.41889(7) | 0.67158(7) | 0.01267(10) |
| N2 | 0.72790(7) | 0.32246(7) | 0.53241(7) | 0.01351(10) |
| N3 | 0.55561(7) | 0.06074(7) | 0.30503(7) | 0.01339(10) |
| N4 | 0.27287(7) | 0.01174(7) | 0.32661(7) | 0.01280(10) |
| C1 | 0.99106(9) | 0.59625(10) | 0.76011(9) | 0.01942(13) |
| H1A | 0.986117 | 0.623127 | 0.659701 | 0.029 |
| H1B | 1.026926 | 0.482457 | 0.737014 | 0.029 |
| H1C | 1.071115 | 0.688009 | 0.856786 | 0.029 |

| | | | | |
|---|---|---|---|---|
| C2 | 0.82128(8) | 0.58884(8) | 0.80116(8) | 0.01419(11) |
| C3 | 0.68888(8) | 0.44545(8) | 0.66597(8) | 0.01277(11) |
| C4 | 0.45308(7) | 0.26490(8) | 0.53151(7) | 0.01222(11) |
| C5 | 0.28539(8) | 0.16833(8) | 0.47020(8) | 0.01289(11) |
| C6 | 0.58054(8) | 0.21095(8) | 0.45070(8) | 0.01197(10) |
| C7 | 0.39873(8) | -0.04147(8) | 0.23715(8) | 0.01316(11) |
| C8 | 0.43681(9) | 0.52490(9) | 0.79643(8) | 0.01683(12) |
| H8A | 0.353245 | 0.572947 | 0.741136 | 0.025 |
| H8B | 0.517124 | 0.621531 | 0.888207 | 0.025 |
| H8C | 0.381008 | 0.451188 | 0.843520 | 0.025 |
| C9 | 0.10591(8) | -0.09711(9) | 0.25020(9) | 0.01672(12) |
| H9A | 0.042207 | -0.051626 | 0.175532 | 0.025 |
| H9B | 0.047236 | -0.093755 | 0.340220 | 0.025 |
| H9C | 0.116855 | -0.218590 | 0.183848 | 0.025 |
| C10 | 0.69213(9) | 0.00891(9) | 0.21831(9) | 0.01818(12) |
| H10A | 0.693305 | -0.116396 | 0.188542 | 0.027 |
| H10B | 0.798932 | 0.077714 | 0.293682 | 0.027 |
| H10C | 0.675544 | 0.030707 | 0.114651 | 0.027 |

$U_{eq}$ is defined as 1/3 of the trace of the orthogonalized $U_{ij}$ tensor.

**Table S6.** Anisotropic displacement parameters [Å$^2$] for **3.**
The anisotropic displacement factor exponent takes the form:
$-2\pi^2[\, h^2(a^*)^2U_{11} + k^2(b^*)^2U_{22} + \ldots + 2hka^*b^*U_{12}\,]$

| Atom | $U_{11}$ | $U_{22}$ | $U_{33}$ | $U_{23}$ | $U_{13}$ | $U_{12}$ |
|---|---|---|---|---|---|---|
| F1 | 0.0209(2) | 0.0212(2) | 0.01258(17) | 0.00765(15) | −0.00066(14) | −0.00173(15) |
| F2 | 0.0238(2) | 0.01206(17) | 0.0192(2) | 0.00422(15) | 0.00260(16) | 0.00237(15) |
| O1 | 0.0137(2) | 0.0211(2) | 0.0197(2) | 0.00398(19) | 0.00721(17) | 0.00240(17) |
| O2 | 0.0200(2) | 0.0150(2) | 0.0153(2) | −0.00079(17) | 0.00381(17) | −0.00006(17) |
| N1 | 0.0130(2) | 0.0123(2) | 0.0103(2) | 0.00224(17) | 0.00244(16) | 0.00154(16) |
| N2 | 0.0122(2) | 0.0131(2) | 0.0130(2) | 0.00338(17) | 0.00212(17) | 0.00084(17) |
| N3 | 0.0122(2) | 0.0127(2) | 0.0122(2) | 0.00161(17) | 0.00361(17) | 0.00128(17) |
| N4 | 0.0114(2) | 0.0127(2) | 0.0121(2) | 0.00320(17) | 0.00210(16) | 0.00042(16) |
| C1 | 0.0147(3) | 0.0202(3) | 0.0187(3) | 0.0045(2) | 0.0027(2) | −0.0021(2) |
| C2 | 0.0158(2) | 0.0126(2) | 0.0119(2) | 0.00393(19) | 0.00086(19) | 0.00021(19) |
| C3 | 0.0126(2) | 0.0125(2) | 0.0115(2) | 0.00395(19) | 0.00126(18) | 0.00083(18) |
| C4 | 0.0116(2) | 0.0125(2) | 0.0106(2) | 0.00294(19) | 0.00225(18) | 0.00128(18) |
| C5 | 0.0126(2) | 0.0135(2) | 0.0116(2) | 0.00422(19) | 0.00283(18) | 0.00153(18) |
| C6 | 0.0121(2) | 0.0114(2) | 0.0108(2) | 0.00300(18) | 0.00229(18) | 0.00135(18) |
| C7 | 0.0134(2) | 0.0126(2) | 0.0122(2) | 0.0039(2) | 0.00264(19) | 0.00154(19) |
| C8 | 0.0186(3) | 0.0169(3) | 0.0130(2) | 0.0024(2) | 0.0064(2) | 0.0043(2) |
| C9 | 0.0125(2) | 0.0168(3) | 0.0171(3) | 0.0047(2) | 0.0011(2) | −0.0015(2) |
| C10 | 0.0161(3) | 0.0178(3) | 0.0184(3) | 0.0030(2) | 0.0082(2) | 0.0035(2) |
| | | | | | | |
| | | | | | | |

**Table S7.** Bond lengths and angles for **3.**

| Atom–Atom | Length [Å] |
|---|---|
| F1–C2 | 1.3785(8) |
| F2–C2 | 1.3824(8) |
| O1–C5 | 1.2282(8) |
| O2–C7 | 1.2253(8) |
| N1–C3 | 1.3572(8) |

| | |
|---|---|
| N1–C4 | 1.3841(8) |
| N1–C8 | 1.4641(8) |
| N2–C3 | 1.3373(8) |
| N2–C6 | 1.3563(8) |
| N3–C6 | 1.3710(8) |
| N3–C7 | 1.3778(8) |
| N3–C10 | 1.4641(8) |
| N4–C5 | 1.4044(8) |
| N4–C7 | 1.4026(8) |
| N4–C9 | 1.4685(8) |
| C1–H1A | 0.9800 |
| C1–H1B | 0.9800 |
| C1–H1C | 0.9800 |
| C1–C2 | 1.4993(10) |
| C2–C3 | 1.5044(9) |
| C4–C5 | 1.4340(9) |
| C4–C6 | 1.3750(8) |
| C8–H8A | 0.9800 |
| C8–H8B | 0.9800 |
| C8–H8C | 0.9800 |
| C9–H9A | 0.9800 |
| C9–H9B | 0.9800 |
| C9–H9C | 0.9800 |
| C10–H10A | 0.9800 |
| C10–H10B | 0.9800 |
| C10–H10C | 0.9800 |
| | |
| **Atom–Atom–Atom** | **Angle [°]** |
| C3–N1–C4 | 105.45(5) |
| C3–N1–C8 | 129.23(5) |
| C4–N1–C8 | 125.32(5) |
| C3–N2–C6 | 103.39(5) |
| C6–N3–C7 | 119.51(5) |
| C6–N3–C10 | 121.10(5) |
| C7–N3–C10 | 119.37(5) |
| C5–N4–C9 | 116.62(5) |
| C7–N4–C5 | 126.62(5) |
| C7–N4–C9 | 116.32(5) |
| H1A–C1–H1B | 109.5 |
| H1A–C1–H1C | 109.5 |
| H1B–C1–H1C | 109.5 |
| C2–C1–H1A | 109.5 |
| C2–C1–H1B | 109.5 |
| C2–C1–H1C | 109.5 |
| F1–C2–F2 | 103.97(5) |
| F1–C2–C1 | 110.36(5) |
| F1–C2–C3 | 108.03(5) |
| F2–C2–C1 | 110.02(5) |
| F2–C2–C3 | 109.36(5) |
| C1–C2–C3 | 114.55(5) |
| N1–C3–C2 | 124.96(5) |
| N2–C3–N1 | 113.62(5) |
| N2–C3–C2 | 121.24(6) |

| | |
|---|---|
| N1–C4–C5 | 131.90(5) |
| C6–C4–N1 | 105.52(5) |
| C6–C4–C5 | 122.57(6) |
| O1–C5–N4 | 121.51(6) |
| O1–C5–C4 | 126.88(6) |
| N4–C5–C4 | 111.61(5) |
| N2–C6–N3 | 125.88(6) |
| N2–C6–C4 | 112.01(5) |
| N3–C6–C4 | 122.11(6) |
| O2–C7–N3 | 121.20(6) |
| O2–C7–N4 | 121.55(6) |
| N3–C7–N4 | 117.25(5) |
| N1–C8–H8A | 109.5 |
| N1–C8–H8B | 109.5 |
| N1–C8–H8C | 109.5 |
| H8A–C8–H8B | 109.5 |
| H8A–C8–H8C | 109.5 |
| H8B–C8–H8C | 109.5 |
| N4–C9–H9A | 109.5 |
| N4–C9–H9B | 109.5 |
| N4–C9–H9C | 109.5 |
| H9A–C9–H9B | 109.5 |
| H9A–C9–H9C | 109.5 |
| H9B–C9–H9C | 109.5 |
| N3–C10–H10A | 109.5 |
| N3–C10–H10B | 109.5 |
| N3–C10–H10C | 109.5 |
| H10A–C10–H10B | 109.5 |
| H10A–C10–H10C | 109.5 |
| H10B–C10–H10C | 109.5 |

**Table S8.** Torsion angles for **3.**

| **Atom–Atom–Atom–Atom** | **Torsion Angle [°]** |
|---|---|
| F1–C2–C3–N1 | 59.87(8) |
| F1–C2–C3–N2 | −114.98(6) |
| F2–C2–C3–N1 | −52.69(8) |
| F2–C2–C3–N2 | 132.46(6) |
| N1–C4–C5–O1 | 3.41(12) |
| N1–C4–C5–N4 | −176.38(6) |
| N1–C4–C6–N2 | 0.02(7) |
| N1–C4–C6–N3 | 179.88(6) |
| C1–C2–C3–N1 | −176.70(6) |
| C1–C2–C3–N2 | 8.45(9) |
| C3–N1–C4–C5 | 179.09(7) |
| C3–N1–C4–C6 | −0.52(7) |
| C3–N2–C6–N3 | −179.36(6) |
| C3–N2–C6–C4 | 0.49(7) |
| C4–N1–C3–N2 | 0.89(7) |
| C4–N1–C3–C2 | −174.31(6) |
| C5–N4–C7–O2 | −173.90(6) |

| | |
|---|---|
| C5–N4–C7–N3 | 6.72(9) |
| C5–C4–C6–N2 | −179.63(6) |
| C5–C4–C6–N3 | 0.22(10) |
| C6–N2–C3–N1 | −0.85(7) |
| C6–N2–C3–C2 | 174.54(6) |
| C6–N3–C7–O2 | 178.15(6) |
| C6–N3–C7–N4 | −2.46(9) |
| C6–C4–C5–O1 | −177.04(6) |
| C6–C4–C5–N4 | 3.17(9) |
| C7–N3–C6–N2 | 179.16(6) |
| C7–N3–C6–C4 | −0.67(9) |
| C7–N4–C5–O1 | 173.39(6) |
| C7–N4–C5–C4 | −6.81(9) |
| C8–N1–C3–N2 | −179.59(6) |
| C8–N1–C3–C2 | 5.22(10) |
| C8–N1–C4–C5 | −0.46(11) |
| C8–N1–C4–C6 | 179.93(6) |
| C9–N4–C5–O1 | 1.32(9) |
| C9–N4–C5–C4 | −178.88(5) |
| C9–N4–C7–O2 | −1.80(9) |
| C9–N4–C7–N3 | 178.82(5) |
| C10–N3–C6–N2 | −2.11(10) |
| C10–N3–C6–C4 | 178.05(6) |
| C10–N3–C7–O2 | −0.60(10) |
| C10–N3–C7–N4 | 178.79(5) |

**Table S9.** Crystal data and structure refinement for **34**.

| | |
|---|---|
| CCDC number | 2355458 |
| Empirical formula | $C_7H_4F_4N_4O$ |
| Formula weight | 236.14 |
| Temperature [K] | 100.00 |
| Crystal system | monoclinic |
| Space group (number) | $C2/c$ (15) |
| $a$ [Å] | 18.844(2) |
| $b$ [Å] | 13.6660(15) |
| $c$ [Å] | 14.2041(19) |
| α [°] | 90 |
| β [°] | 107.258(6) |
| γ [°] | 90 |
| Volume [Å$^3$] | 3493.2(7) |
| $Z$ | 16 |
| $\rho_{calc}$ [gcm$^{-3}$] | 1.796 |
| $\mu$ [mm$^{-1}$] | 0.183 |
| $F$(000) | 1888 |
| Crystal size [mm$^3$] | 0.713×0.228×0.062 |
| Crystal color | colorless |
| Crystal shape | plate |
| Radiation | Mo$K_\alpha$ (λ=0.71073 Å) |
| 2θ range [°] | 3.74 to 57.52 (0.74 Å) |
| Index ranges | $-25 \leq h \leq 25$<br>$-18 \leq k \leq 18$<br>$-19 \leq l \leq 19$ |
| Reflections collected | 41043 |
| Independent reflections | 4539<br>$R_{int}$ = 0.0251<br>$R_{sigma}$ = 0.0133 |
| Completeness to θ = 25.242° | 100.0 % |
| Data / Restraints / Parameters | 4539/316/404 |

| | |
|---|---|
| Absorption correction $T_{min}/T_{max}$ (method) | 0.9177/1.0000 (numerical) |
| Goodness-of-fit on $F^2$ | 1.022 |
| Final $R$ indexes [$I \geq 2\sigma(I)$] | $R_1$ = 0.0486<br>w$R_2$ = 0.1409 |
| Final $R$ indexes [all data] | $R_1$ = 0.0567<br>w$R_2$ = 0.1505 |
| Largest peak/hole [eÅ$^{-3}$] | 0.49/−1.05 |

Refinement details for **34**

The second molecule in asymmetric unit exhibits disorder of the tetrafluoroethyl group. The moiety was split in three disorder positions and heavily restrained to keep a chemically reasonable geometry. However, the residual density map in that area indicates further disorder, which could not be modelled successfully.

**Table S10.** Atomic coordinates and $U_{eq}$ [Å$^2$] for **34.**

| **Atom** | ***x*** | ***y*** | ***z*** | $U_{eq}$ |
|---|---|---|---|---|
| F1 | 0.56054(6) | 0.60877(8) | 0.32815(7) | 0.0281(2) |
| F2 | 0.53865(7) | 0.58532(9) | 0.46734(9) | 0.0342(3) |
| F3 | 0.60703(9) | 0.75099(9) | 0.46838(11) | 0.0491(4) |
| F4 | 0.69377(7) | 0.68553(9) | 0.41567(10) | 0.0391(3) |
| O1 | 0.46190(6) | 0.39943(9) | 0.33509(9) | 0.0246(3) |
| N1 | 0.52077(7) | 0.25333(11) | 0.37295(11) | 0.0232(3) |
| H1 | 0.477554 | 0.223538 | 0.351491 | 0.028 |
| N2 | 0.64977(7) | 0.22858(10) | 0.44085(11) | 0.0220(3) |
| N3 | 0.71570(7) | 0.38124(10) | 0.47890(11) | 0.0229(3) |
| H3 | 0.760345 | 0.355671 | 0.502320 | 0.027 |
| N4 | 0.70155(8) | 0.47860(11) | 0.47460(11) | 0.0239(3) |
| C1 | 0.52062(8) | 0.35557(12) | 0.37031(11) | 0.0201(3) |
| C2 | 0.59437(8) | 0.39428(12) | 0.41177(11) | 0.0195(3) |
| C3 | 0.65308(8) | 0.32819(12) | 0.44294(11) | 0.0197(3) |
| C4 | 0.62869(9) | 0.48699(12) | 0.43385(11) | 0.0205(3) |
| C5 | 0.59423(9) | 0.58606(13) | 0.42386(12) | 0.0232(3) |
| C7 | 0.58207(9) | 0.19620(13) | 0.40589(12) | 0.0231(3) |
| H7 | 0.575027 | 0.127311 | 0.403232 | 0.028 |
| C8 | 0.64742(12) | 0.66919(13) | 0.47046(14) | 0.0315(4) |
| H8 | 0.676246 | 0.652382 | 0.539778 | 0.038 |
| F1A | 0.1169(3) | 0.4356(4) | 0.1115(4) | 0.0265(11) |
| F1B | 0.1254(3) | 0.4555(4) | 0.0812(4) | 0.0316(12) |
| F1C | 0.1191(3) | 0.4817(4) | 0.0457(4) | 0.0320(11) |
| F2A | 0.0295(5) | 0.5285(8) | 0.1647(7) | 0.0356(18) |
| F2B | 0.0334(4) | 0.5271(7) | 0.1333(6) | 0.041(2) |
| F2C | 0.0358(4) | 0.5372(7) | 0.1016(8) | 0.051(2) |
| F3A | 0.0562(4) | 0.3482(5) | 0.2276(5) | 0.0420(15) |
| F3B | 0.0506(3) | 0.3506(4) | 0.1817(5) | 0.0478(15) |
| F3C | 0.0554(2) | 0.3657(3) | 0.1000(4) | 0.0380(10) |
| F4A | 0.1027(6) | 0.4599(7) | 0.3428(5) | 0.0496(16) |
| F4B | 0.0910(5) | 0.4561(5) | 0.3065(5) | 0.0399(13) |
| F4C | 0.0941(6) | 0.4502(6) | 0.2744(6) | 0.0485(17) |
| O1A | 0.28309(7) | 0.42141(9) | 0.26926(13) | 0.0380(4) |
| N1' | 0.35892(7) | 0.55395(10) | 0.31728(11) | 0.0205(3) |
| H1' | 0.3947(15) | 0.5148(19) | 0.3263(19) | 0.036(6) |
| N2' | 0.31852(7) | 0.71888(10) | 0.30851(10) | 0.0203(3) |
| N3' | 0.18592(7) | 0.73123(10) | 0.23525(11) | 0.0209(3) |
| H3' | 0.182917 | 0.795398 | 0.237558 | 0.025 |
| N4' | 0.12702(8) | 0.67095(11) | 0.19828(13) | 0.0285(3) |
| C1' | 0.28990(9) | 0.51047(12) | 0.27801(14) | 0.0247(3) |
| C2' | 0.23159(9) | 0.58158(12) | 0.25170(14) | 0.0237(3) |
| C3' | 0.24956(8) | 0.68006(11) | 0.26793(11) | 0.0185(3) |
| C4' | 0.15401(10) | 0.58087(13) | 0.20757(17) | 0.0339(4) |
| C5A | 0.1005(4) | 0.4973(5) | 0.1953(6) | 0.0213(11) |

| | | | | |
|---|---|---|---|---|
| C5B | 0.1049(4) | 0.4917(5) | 0.1677(5) | 0.0226(11) |
| C5C | 0.1069(4) | 0.5052(6) | 0.1363(6) | 0.0340(11) |
| C6A | 0.1113(6) | 0.4177(8) | 0.2691(8) | 0.0288(13) |
| H6A | 0.162069 | 0.388599 | 0.283886 | 0.035 |
| C6B | 0.1067(4) | 0.4137(5) | 0.2350(5) | 0.0241(12) |
| H6B | 0.156282 | 0.380744 | 0.256554 | 0.029 |
| C6C | 0.1006(5) | 0.4188(6) | 0.1906(6) | 0.0439(14) |
| H6C | 0.150842 | 0.387672 | 0.207741 | 0.053 |
| C7' | 0.37002(8) | 0.65231(12) | 0.33031(12) | 0.0206(3) |
| H7' | 0.419790 | 0.674002 | 0.357744 | 0.025 |

$U_{eq}$ is defined as 1/3 of the trace of the orthogonalized $U_{ij}$ tensor.

**Table S11.** Anisotropic displacement parameters [Å$^2$] for **34**.
The anisotropic displacement factor exponent takes the form:
$-2\pi^2[\, h^2(a^*)^2U_{11} + k^2(b^*)^2U_{22} + \ldots + 2hka^*b^*U_{12}\,]$

| **Atom** | $U_{11}$ | $U_{22}$ | $U_{33}$ | $U_{23}$ | $U_{13}$ | $U_{12}$ |
|---|---|---|---|---|---|---|
| F1 | 0.0268(5) | 0.0311(5) | 0.0229(5) | 0.0048(4) | 0.0022(4) | 0.0083(4) |
| F2 | 0.0347(6) | 0.0356(6) | 0.0396(6) | 0.0048(5) | 0.0222(5) | 0.0101(5) |
| F3 | 0.0600(9) | 0.0253(6) | 0.0585(8) | −0.0050(6) | 0.0121(7) | 0.0099(6) |
| F4 | 0.0379(6) | 0.0332(6) | 0.0449(7) | 0.0044(5) | 0.0102(5) | −0.0090(5) |
| O1 | 0.0147(5) | 0.0309(6) | 0.0255(6) | 0.0005(5) | 0.0016(4) | 0.0060(4) |
| N1 | 0.0123(6) | 0.0253(7) | 0.0286(7) | −0.0008(5) | 0.0006(5) | 0.0005(5) |
| N2 | 0.0139(6) | 0.0225(7) | 0.0269(7) | 0.0014(5) | 0.0018(5) | 0.0008(5) |
| N3 | 0.0136(6) | 0.0218(7) | 0.0295(7) | 0.0026(5) | 0.0007(5) | 0.0009(5) |
| N4 | 0.0177(6) | 0.0222(7) | 0.0293(7) | 0.0021(5) | 0.0031(5) | 0.0011(5) |
| C1 | 0.0152(6) | 0.0251(8) | 0.0187(7) | 0.0007(6) | 0.0032(5) | 0.0027(6) |
| C2 | 0.0151(7) | 0.0235(7) | 0.0186(7) | 0.0013(6) | 0.0030(5) | 0.0019(5) |
| C3 | 0.0130(6) | 0.0229(7) | 0.0207(7) | 0.0021(6) | 0.0012(5) | 0.0012(5) |
| C4 | 0.0171(7) | 0.0231(7) | 0.0201(7) | 0.0024(6) | 0.0039(5) | 0.0021(5) |
| C5 | 0.0222(7) | 0.0266(8) | 0.0214(7) | 0.0020(6) | 0.0075(6) | 0.0048(6) |
| C7 | 0.0155(7) | 0.0234(7) | 0.0278(8) | 0.0004(6) | 0.0023(6) | 0.0012(6) |
| C8 | 0.0393(10) | 0.0222(8) | 0.0306(9) | −0.0004(7) | 0.0065(7) | 0.0021(7) |
| F1A | 0.023(2) | 0.012(2) | 0.034(3) | −0.0115(19) | −0.0086(18) | −0.0023(16) |
| F1B | 0.0381(19) | 0.014(3) | 0.041(3) | −0.006(2) | 0.009(2) | −0.0048(18) |
| F1C | 0.048(2) | 0.012(2) | 0.034(2) | −0.0008(16) | 0.008(2) | −0.0092(18) |
| F2A | 0.0090(15) | 0.031(2) | 0.063(5) | 0.014(3) | 0.005(2) | −0.0012(14) |
| F2B | 0.0108(16) | 0.031(2) | 0.073(7) | 0.013(4) | −0.003(3) | −0.0028(15) |
| F2C | 0.0223(19) | 0.035(3) | 0.082(5) | −0.017(3) | −0.006(2) | −0.001(2) |
| F3A | 0.025(2) | 0.0256(19) | 0.071(4) | −0.001(3) | 0.008(3) | −0.0060(15) |
| F3B | 0.0178(16) | 0.0224(16) | 0.099(4) | −0.018(3) | 0.010(3) | −0.0061(12) |
| F3C | 0.0196(15) | 0.0184(15) | 0.064(3) | −0.0093(16) | −0.0062(16) | −0.0034(12) |
| F4A | 0.070(4) | 0.038(3) | 0.052(3) | −0.003(3) | 0.036(3) | −0.005(3) |
| F4B | 0.058(3) | 0.0245(19) | 0.043(3) | −0.009(3) | 0.024(3) | −0.0085(17) |
| F4C | 0.078(4) | 0.024(3) | 0.048(3) | 0.001(2) | 0.027(3) | −0.010(3) |
| O1A | 0.0195(6) | 0.0151(6) | 0.0748(11) | −0.0025(6) | 0.0069(6) | 0.0000(4) |
| N1' | 0.0133(6) | 0.0172(6) | 0.0297(7) | 0.0023(5) | 0.0044(5) | 0.0018(5) |
| N2' | 0.0165(6) | 0.0170(6) | 0.0263(7) | −0.0008(5) | 0.0046(5) | −0.0019(5) |
| N3' | 0.0158(6) | 0.0159(6) | 0.0293(7) | 0.0019(5) | 0.0041(5) | 0.0010(5) |
| N4' | 0.0149(6) | 0.0192(6) | 0.0465(9) | 0.0003(6) | 0.0016(6) | 0.0002(5) |
| C1' | 0.0155(7) | 0.0163(7) | 0.0407(9) | 0.0010(6) | 0.0056(6) | −0.0003(5) |
| C2' | 0.0147(7) | 0.0154(7) | 0.0380(9) | 0.0003(6) | 0.0033(6) | −0.0004(5) |
| C3' | 0.0156(7) | 0.0164(7) | 0.0229(7) | 0.0011(5) | 0.0047(5) | 0.0002(5) |

| | | | | | | |
|---|---|---|---|---|---|---|
| C4' | 0.0154(7) | 0.0179(7) | 0.0609(12) | −0.0018(7) | 0.0001(7) | −0.0008(5) |
| C5A | 0.0094(15) | 0.0169(16) | 0.035(3) | −0.0003(16) | 0.0029(19) | −0.0010(12) |
| C5B | 0.0126(15) | 0.0175(15) | 0.035(3) | 0.0014(16) | 0.0027(19) | −0.0015(11) |
| C5C | 0.0218(18) | 0.034(2) | 0.042(2) | −0.0057(17) | 0.0025(18) | −0.0022(16) |
| C6A | 0.022(2) | 0.027(2) | 0.037(3) | 0.004(2) | 0.008(2) | −0.0025(17) |
| C6B | 0.0226(18) | 0.0210(16) | 0.028(3) | −0.004(2) | 0.007(2) | −0.0032(13) |
| C6C | 0.040(3) | 0.041(2) | 0.045(3) | −0.004(2) | 0.004(2) | −0.006(2) |
| C7' | 0.0161(7) | 0.0192(7) | 0.0253(7) | −0.0002(6) | 0.0046(6) | −0.0013(5) |

**Table S12.** Bond lengths and angles for **34.**

| **Atom–Atom** | **Length [Å]** |
|---|---|
| F1–C5 | 1.3549(19) |
| F2–C5 | 1.3646(19) |
| F3–C8 | 1.348(2) |
| F4–C8 | 1.350(2) |
| O1–C1 | 1.2269(19) |
| N1–H1 | 0.8800 |
| N1–C1 | 1.398(2) |
| N1–C7 | 1.357(2) |
| N2–C3 | 1.363(2) |
| N2–C7 | 1.302(2) |
| N3–H3 | 0.8800 |
| N3–N4 | 1.3548(19) |
| N3–C3 | 1.350(2) |
| N4–C4 | 1.327(2) |
| C1–C2 | 1.440(2) |
| C2–C3 | 1.395(2) |
| C2–C4 | 1.415(2) |
| C4–C5 | 1.490(2) |
| C5–C8 | 1.530(3) |
| C7–H7 | 0.9500 |
| C8–H8 | 1.0000 |
| F1A–C5A | 1.563(8) |
| F1B–C5B | 1.478(7) |
| F1C–C5C | 1.411(8) |
| F2A–C5A | 1.347(9) |
| F2B–C5B | 1.378(8) |
| F2C–C5C | 1.357(8) |
| F3A–C6A | 1.401(10) |
| F3B–C6B | 1.399(6) |
| F3C–C6C | 1.503(8) |
| F4A–C6A | 1.246(10) |
| F4B–C6B | 1.277(7) |
| F4C–C6C | 1.304(9) |
| O1A–C1' | 1.226(2) |
| N1'–H1' | 0.84(3) |
| N1'–C1' | 1.387(2) |
| N1'–C7' | 1.364(2) |
| N2'–C3' | 1.3631(19) |
| N2'–C7' | 1.299(2) |
| N3'–H3' | 0.8800 |
| N3'–N4' | 1.3566(19) |
| N3'–C3' | 1.3462(19) |
| N4'–C4' | 1.324(2) |
| C1'–C2' | 1.431(2) |
| C2'–C3' | 1.390(2) |
| C2'–C4' | 1.410(2) |
| C4'–C5A | 1.499(7) |
| C4'–C5B | 1.534(6) |
| C4'–C5C | 1.532(7) |
| C5A–C6A | 1.482(10) |
| C5B–C6B | 1.425(8) |
| C5C–C6C | 1.434(11) |
| C6A–H6A | 1.0000 |
| C6B–H6B | 1.0000 |
| C6C–H6C | 1.0000 |
| C7'–H7' | 0.9500 |
| | |
| **Atom–Atom–Atom** | **Angle [°]** |
| C1–N1–H1 | 117.3 |
| C7–N1–H1 | 117.3 |
| C7–N1–C1 | 125.37(14) |
| C7–N2–C3 | 112.41(14) |
| N4–N3–H3 | 124.1 |
| C3–N3–H3 | 124.1 |
| C3–N3–N4 | 111.85(13) |
| C4–N4–N3 | 105.60(13) |
| O1–C1–N1 | 119.58(15) |
| O1–C1–C2 | 129.17(16) |
| N1–C1–C2 | 111.25(13) |
| C3–C2–C1 | 118.06(15) |
| C3–C2–C4 | 104.02(13) |
| C4–C2–C1 | 137.91(15) |
| N2–C3–C2 | 127.82(14) |
| N3–C3–N2 | 125.04(14) |
| N3–C3–C2 | 107.12(14) |
| N4–C4–C2 | 111.41(14) |
| N4–C4–C5 | 119.03(15) |
| C2–C4–C5 | 129.41(14) |
| F1–C5–F2 | 105.17(13) |
| F1–C5–C4 | 111.40(13) |
| F1–C5–C8 | 108.42(14) |

| | |
|---|---|
| F2–C5–C4 | 108.75(14) |
| F2–C5–C8 | 107.59(14) |
| C4–C5–C8 | 114.99(14) |
| N1–C7–H7 | 117.5 |
| N2–C7–N1 | 125.01(16) |
| N2–C7–H7 | 117.5 |
| F3–C8–F4 | 107.93(16) |
| F3–C8–C5 | 108.26(16) |
| F3–C8–H8 | 110.5 |
| F4–C8–C5 | 109.06(15) |
| F4–C8–H8 | 110.5 |
| C5–C8–H8 | 110.5 |
| C1'–N1'–H1' | 113.7(18) |
| C7'–N1'–H1' | 121.6(18) |
| C7'–N1'–C1' | 124.34(14) |
| C7'–N2'–C3' | 112.29(13) |
| N4'–N3'–H3' | 124.4 |
| C3'–N3'–H3' | 124.4 |
| C3'–N3'–N4' | 111.15(13) |
| C4'–N4'–N3' | 106.27(13) |
| O1A–C1'–N1' | 121.55(15) |
| O1A–C1'–C2' | 126.76(15) |
| N1'–C1'–C2' | 111.69(14) |
| C3'–C2'–C1' | 118.93(14) |
| C3'–C2'–C4' | 104.47(14) |
| C4'–C2'–C1' | 136.57(15) |
| N2'–C3'–C2' | 126.99(14) |
| N3'–C3'–N2' | 125.70(14) |
| N3'–C3'–C2' | 107.31(14) |
| N4'–C4'–C2' | 110.80(15) |
| N4'–C4'–C5A | 118.1(3) |
| N4'–C4'–C5B | 122.1(3) |
| N4'–C4'–C5C | 115.6(3) |
| C2'–C4'–C5A | 129.1(3) |
| C2'–C4'–C5B | 127.0(3) |
| C2'–C4'–C5C | 128.3(3) |
| F2A–C5A–F1A | 109.1(6) |
| F2A–C5A–C4' | 111.4(7) |

| | |
|---|---|
| F2A–C5A–C6A | 112.0(7) |
| C4'–C5A–F1A | 102.7(5) |
| C6A–C5A–F1A | 97.3(6) |
| C6A–C5A–C4' | 122.0(7) |
| F1B–C5B–C4' | 107.1(4) |
| F2B–C5B–F1B | 107.0(6) |
| F2B–C5B–C4' | 105.7(6) |
| F2B–C5B–C6B | 109.1(6) |
| C6B–C5B–F1B | 110.4(5) |
| C6B–C5B–C4' | 117.1(5) |
| F1C–C5C–C4' | 122.5(6) |
| F1C–C5C–C6C | 111.4(6) |
| F2C–C5C–F1C | 98.8(7) |
| F2C–C5C–C4' | 109.8(7) |
| F2C–C5C–C6C | 103.3(7) |
| C6C–C5C–C4' | 108.9(6) |
| F3A–C6A–C5A | 105.7(7) |
| F3A–C6A–H6A | 111.2 |
| F4A–C6A–F3A | 113.7(8) |
| F4A–C6A–C5A | 103.3(8) |
| F4A–C6A–H6A | 111.2 |
| C5A–C6A–H6A | 111.2 |
| F3B–C6B–C5B | 103.5(5) |
| F3B–C6B–H6B | 112.1 |
| F4B–C6B–F3B | 113.2(6) |
| F4B–C6B–C5B | 103.2(5) |
| F4B–C6B–H6B | 112.1 |
| C5B–C6B–H6B | 112.1 |
| F3C–C6C–H6C | 104.9 |
| F4C–C6C–F3C | 139.2(8) |
| F4C–C6C–C5C | 105.4(7) |
| F4C–C6C–H6C | 104.9 |
| C5C–C6C–F3C | 93.1(6) |
| C5C–C6C–H6C | 104.9 |
| N1'–C7'–H7' | 117.1 |
| N2'–C7'–N1' | 125.73(14) |
| N2'–C7'–H7' | 117.1 |

**Table S13.** Torsion angles for **34**

| **Atom–Atom–Atom–Atom** | **Torsion Angle [°]** |
|---|---|
| F1–C5–C8–F3 | 63.33(19) |
| F1–C5–C8–F4 | −53.86(18) |
| F2–C5–C8–F3 | −49.93(19) |
| F2–C5–C8–F4 | −167.12(14) |
| O1–C1–C2–C3 | −176.91(16) |
| O1–C1–C2–C4 | 4.2(3) |
| N1–C1–C2–C3 | 2.8(2) |
| N1–C1–C2–C4 | −176.05(18) |
| N3–N4–C4–C2 | 0.47(19) |
| N3–N4–C4–C5 | 176.47(14) |
| N4–N3–C3–N2 | −177.68(15) |
| N4–N3–C3–C2 | 0.91(19) |
| N4–C4–C5–F1 | 119.14(16) |
| N4–C4–C5–F2 | −125.39(16) |
| N4–C4–C5–C8 | −4.7(2) |
| C1–N1–C7–N2 | 0.7(3) |
| C1–C2–C3–N2 | −1.2(3) |
| C1–C2–C3–N3 | −179.76(14) |
| C1–C2–C4–N4 | 179.00(18) |
| C1–C2–C4–C5 | 3.5(3) |
| C2–C4–C5–F1 | −65.7(2) |
| C2–C4–C5–F2 | 49.8(2) |
| C2–C4–C5–C8 | 170.48(16) |
| C3–N2–C7–N1 | 1.2(2) |
| C3–N3–N4–C4 | −0.86(19) |
| C3–C2–C4–N4 | 0.06(18) |
| C3–C2–C4–C5 | −175.42(16) |
| C4–C2–C3–N2 | 177.97(16) |
| C4–C2–C3–N3 | −0.57(17) |
| C4–C5–C8–F3 | −171.25(15) |
| C4–C5–C8–F4 | 71.56(19) |
| C7–N1–C1–O1 | 177.00(16) |
| C7–N1–C1–C2 | −2.7(2) |
| C7–N2–C3–N3 | 177.40(16) |
| C7–N2–C3–C2 | −0.9(2) |
| F1A–C5A–C6A–F3A | −63.2(8) |
| F1A–C5A–C6A–F4A | 177.1(7) |
| F1B–C5B–C6B–F3B | −64.3(6) |
| F1B–C5B–C6B–F4B | 177.5(6) |
| F1C–C5C–C6C–F3C | −40.5(7) |
| F1C–C5C–C6C–F4C | 176.3(7) |
| F2A–C5A–C6A–F3A | 50.9(10) |
| F2A–C5A–C6A–F4A | −68.9(9) |
| F2B–C5B–C6B–F3B | 52.9(7) |
| F2B–C5B–C6B–F4B | −65.3(8) |
| F2C–C5C–C6C–F3C | 64.7(7) |
| F2C–C5C–C6C–F4C | −78.6(9) |

| | |
|---|---|
| O1A–C1'–C2'–C3' | 179.3(2) |
| O1A–C1'–C2'–C4' | −3.0(4) |
| N1'–C1'–C2'–C3' | −0.3(2) |
| N1'–C1'–C2'–C4' | 177.5(2) |
| N3'–N4'–C4'–C2' | 0.3(2) |
| N3'–N4'–C4'–C5A | 165.7(4) |
| N3'–N4'–C4'–C5B | −176.0(3) |
| N3'–N4'–C4'–C5C | −156.1(4) |
| N4'–N3'–C3'–N2' | 179.47(16) |
| N4'–N3'–C3'–C2' | −0.03(19) |
| N4'–C4'–C5A–F1A | 123.8(4) |
| N4'–C4'–C5A–F2A | 7.1(7) |
| N4'–C4'–C5A–C6A | −129.1(7) |
| N4'–C4'–C5B–F1B | 108.6(4) |
| N4'–C4'–C5B–F2B | −5.2(6) |
| N4'–C4'–C5B–C6B | −126.8(5) |
| N4'–C4'–C5C–F1C | 93.8(7) |
| N4'–C4'–C5C–F2C | −21.1(8) |
| N4'–C4'–C5C–C6C | −133.6(5) |
| C1'–N1'–C7'–N2' | 0.2(3) |
| C1'–C2'–C3'–N2' | −0.9(3) |
| C1'–C2'–C3'–N3' | 178.62(16) |
| C1'–C2'–C4'–N4' | −178.3(2) |
| C1'–C2'–C4'–C5A | 18.3(6) |
| C1'–C2'–C4'–C5B | −2.2(5) |
| C1'–C2'–C4'–C5C | −25.7(6) |
| C2'–C4'–C5A–F1A | −73.9(6) |
| C2'–C4'–C5A–F2A | 169.4(5) |
| C2'–C4'–C5A–C6A | 33.2(9) |
| C2'–C4'–C5B–F1B | −67.0(5) |
| C2'–C4'–C5B–F2B | 179.1(4) |
| C2'–C4'–C5B–C6B | 57.5(7) |
| C2'–C4'–C5C–F1C | −57.7(8) |
| C2'–C4'–C5C–F2C | −172.7(5) |
| C2'–C4'–C5C–C6C | 74.9(7) |
| C3'–N2'–C7'–N1' | −1.2(2) |
| C3'–N3'–N4'–C4' | −0.2(2) |
| C3'–C2'–C4'–N4' | −0.4(2) |
| C3'–C2'–C4'–C5A | −163.7(4) |
| C3'–C2'–C4'–C5B | 175.7(4) |
| C3'–C2'–C4'–C5C | 152.3(4) |
| C4'–C2'–C3'–N2' | −179.27(17) |
| C4'–C2'–C3'–N3' | 0.2(2) |
| C4'–C5A–C6A–F3A | −173.2(7) |
| C4'–C5A–C6A–F4A | 67.1(9) |
| C4'–C5B–C6B–F3B | 172.8(5) |
| C4'–C5B–C6B–F4B | 54.6(7) |
| C4'–C5C–C6C–F3C | −178.6(5) |
| C4'–C5C–C6C–F4C | 38.1(9) |

| | |
|---|---|
| C7'–N1'–C1'–O1A | −178.95(18) |
| C7'–N1'–C1'–C2' | 0.6(2) |
| C7'–N2'–C3'–N3' | −177.81(15) |
| C7'–N2'–C3'–C2' | 1.6(2) |

## EPR detection of free fluoroalkyl radical

Measurements were done on a Bruker EPR spectrometer with the following parameters: modulation amplitude: 2 G; modulation frequency**:** 100 kHz; microwave frequency: 9,394551·$10^9$ (127K); 9,392129 ·$10^9$ (141K); 9,389916·$10^9$ (146K).

Radical species are not stable over a longer period of time and need to be freshly prepared and frozen afterwards.

**Experiment**: Into a 15 mL Schlenk tube (undivided electrochemical cell) charged with a solid mixture of $LiClO_4$ (32 mg, 0.3 mmol), $Fe(ClO_4)_3$·$10H_2O$ (32 mg, 0.04 mmol), and 2,2-difluoropropanoic acid **2a** (99 mg, 0.9 mmol), equipped with a stirring bar (ca. 9 × 2 × 2 mm), platinum (Pt) cathode (25 × 10 × 0.25 mm) and a glassy carbon (GC) anode (25 × 10 × 1.5 mm), MeCN (3 mL) was added under positive pressure of nitrogen. The reaction vessel was sealed with a rubber septum, and the resulting mixture was stirred under 390 nm irradiation and CCE at 4.0 mA for 1 hour. Then the reaction was stopped, diluted with 3 mL of degassed toluene, frozen by liquid nitrogen and analyzed by EPR spectroscopy at T = 127 K to 146 K. Spectrum exhibiting resonance at 3000-3600 G with a g-value of 2.0032±0.0010 corroborate the literature data for $CF_2Me$ radical.[66]

## References


40. Y. Qiu, A. Scheremetjew, L. H. Finger, L. Ackermann, *Chem. Eur. J.* **2020**, *26*, 3241–3246.

54. Gaussian 16, Revision A.03, M. J. Frisch, G. W. Trucks, H. B. Schlegel, G. E. Scuseria, M. A. Robb, J. R. Cheeseman, G. Scalmani, V. Barone, G. A. Petersson, H. Nakatsuji, X. Li, M. Caricato, A. V. Marenich, J. Bloino, B. G. Janesko, R. Gomperts, B. Mennucci, H. P. Hratchian, J. V. Ortiz, A. F. Izmaylov, J. L. Sonnenberg, D. Williams-Young, F. Ding, F. Lipparini, F. Egidi, J. Goings, B. Peng, A. Petrone, T. Henderson, D. Ranasinghe, V. G. Zakrzewski, J. Gao, N. Rega, G. Zheng, W. Liang, M. Hada, M. Ehara, K. Toyota, R. Fukuda, J. Hasegawa, M. Ishida, T. Nakajima, Y. Honda, O. Kitao, H. Nakai, T. Vreven, K. Throssell, J. A. Montgomery, Jr., J. E. Peralta, F. Ogliaro, M. J. Bearpark, J. J. Heyd, E. N. Brothers, K. N. Kudin, V. N. Staroverov, T. A. Keith, R. Kobayashi, J. Normand, K. Raghavachari, A. P. Rendell, J. C. Burant, S. S. Iyengar, J. Tomasi, M. Cossi, J. M. Millam, M. Klene, C. Adamo, R. Cammi, J. W. Ochterski, R. L. Martin, K. Morokuma, O. Farkas, J. B. Foresman, D. J. Fox, Gaussian, Inc., Wallingford CT, **2016**.
55. a) F. Weigend, *Phys. Chem. Chem. Phys.* **2006**, *8*, 1057–1065; b) F. Weigend, R. Ahlrichs, *Phys. Chem. Chem. Phys.* **2005**, *7,* 3297–3305.
56. J.-D. Chai, M. Head-Gordon, *Phys. Chem. Chem. Phys.* **2008**, *10*, 6615–6620.
57. A.V. Marenich, C. J. Cramer, D. G. Truhlar, *J. Phys. Chem. B* **2009**, *113*, 6378–6396.
58. L. R. Domingo, P. Pérez, *Org. Biomol. Chem.* **2013**, *11*, 4350–4358.
59. R. F. Ribeiro, A.V. Marenich, C. J. Cramer, D. G. Truhlar, *Phys. Chem. Chem. Phys.* **2011**, *13*, 10908–10922.
60. Bruker, *SAINT*, *V8.40B*, Bruker AXS Inc., Madison, Wisconsin, USA, **2015**.
61. L. Krause, R. Herbst-Irmer, G. Sheldrick, D. Stalke, *J. Appl. Crystallogr.* **2015**, *48*, 3–10.
62. G. Sheldrick, *Acta Crystallogr. Section A* **2015**, *71*, 3–8.
63. G. Sheldrick, *Acta Crystallogr. Section C* **2015**, *71*, 3–8.
64. O. Dolomanov, L. Bourhis, R. Gildea, J. Howard, H. Puschmann, *J. Appl. Cryst* **2009**, *42*, 339–341.
65. C. R. Groom, I. J. Bruno, M. P. Lightfoot, S. C. Ward, *Acta Cryst. B72*, **2016**, 171–179.
66. K. S. Chen, P. J. Krusic, P. Meakin, J. K. Kochi, *J. Phys. Chem.* **1974**, *78*, 2014–2030.

# NMR spectra

## 8-(1,1-Difluoroethyl)-1,3,7-trimethyl-3,7-dihydro-1*H*-purine-2,6-dione 3

$^1$H NMR (300 MHz, $CDCl_3$)

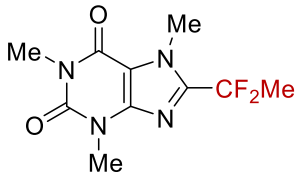


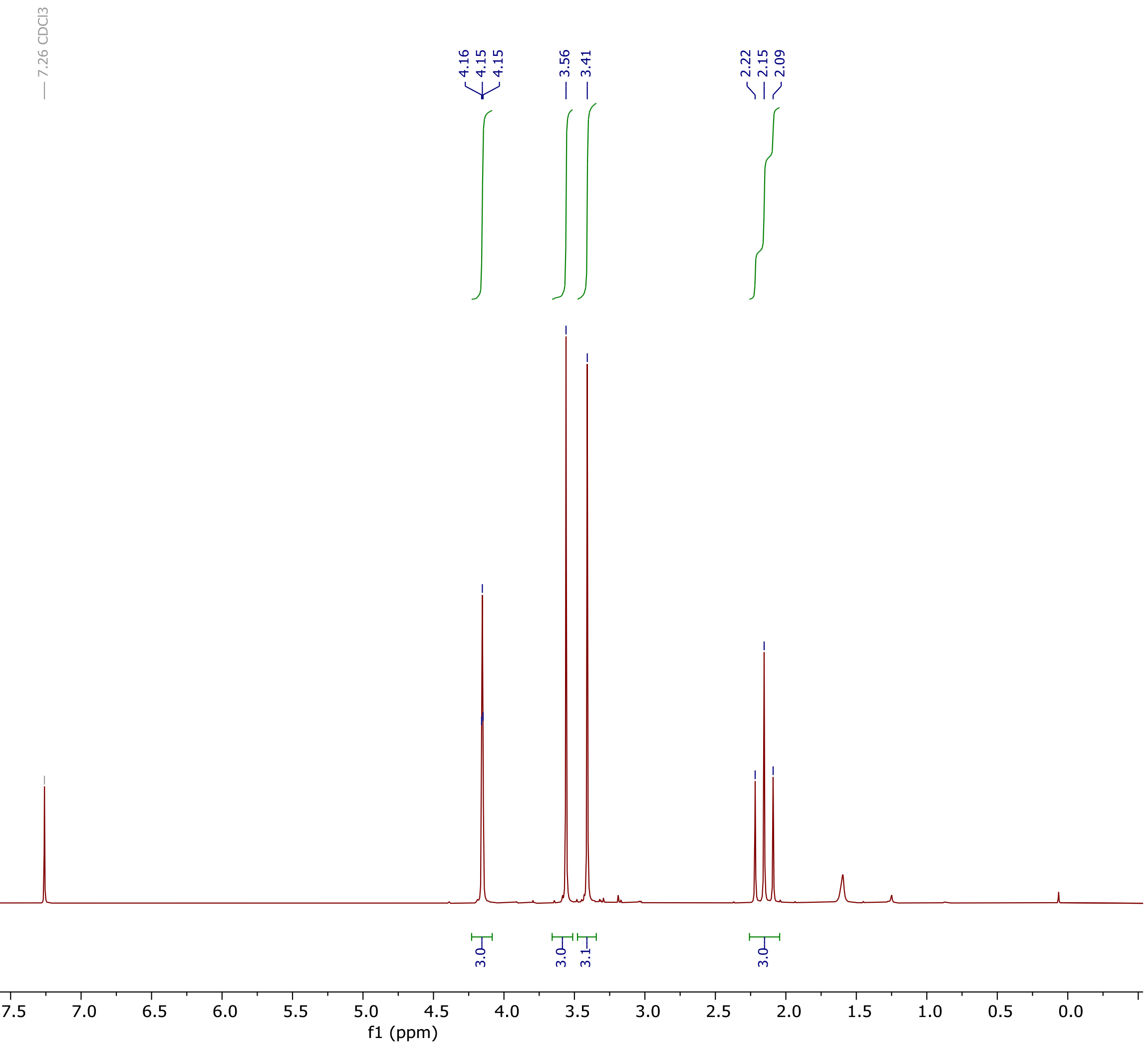

$^{13}C$ NMR (75 MHz, $CDCl_3$)

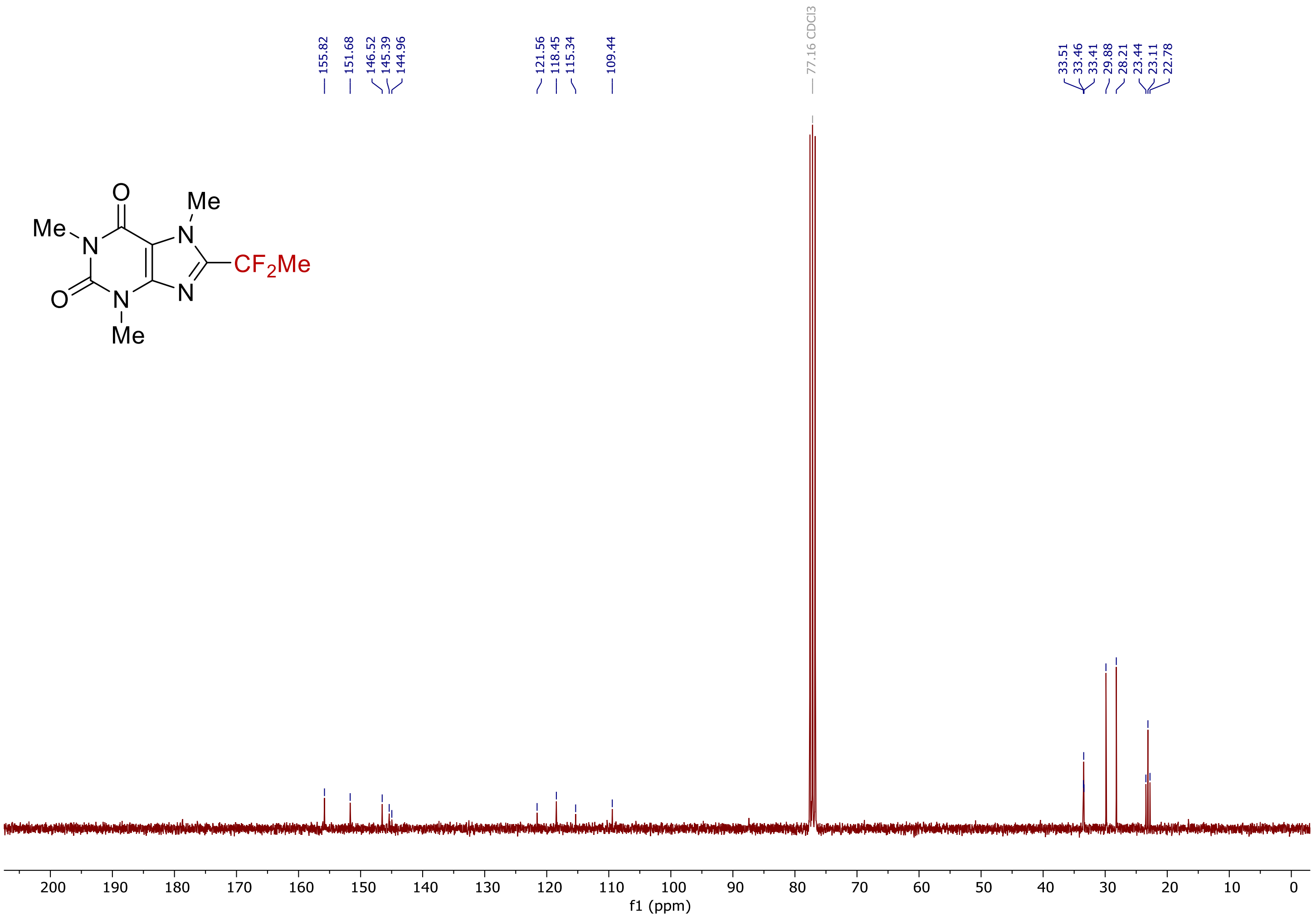

$^{19}F$ NMR (282 MHz, $CDCl_3$)

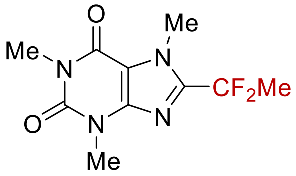


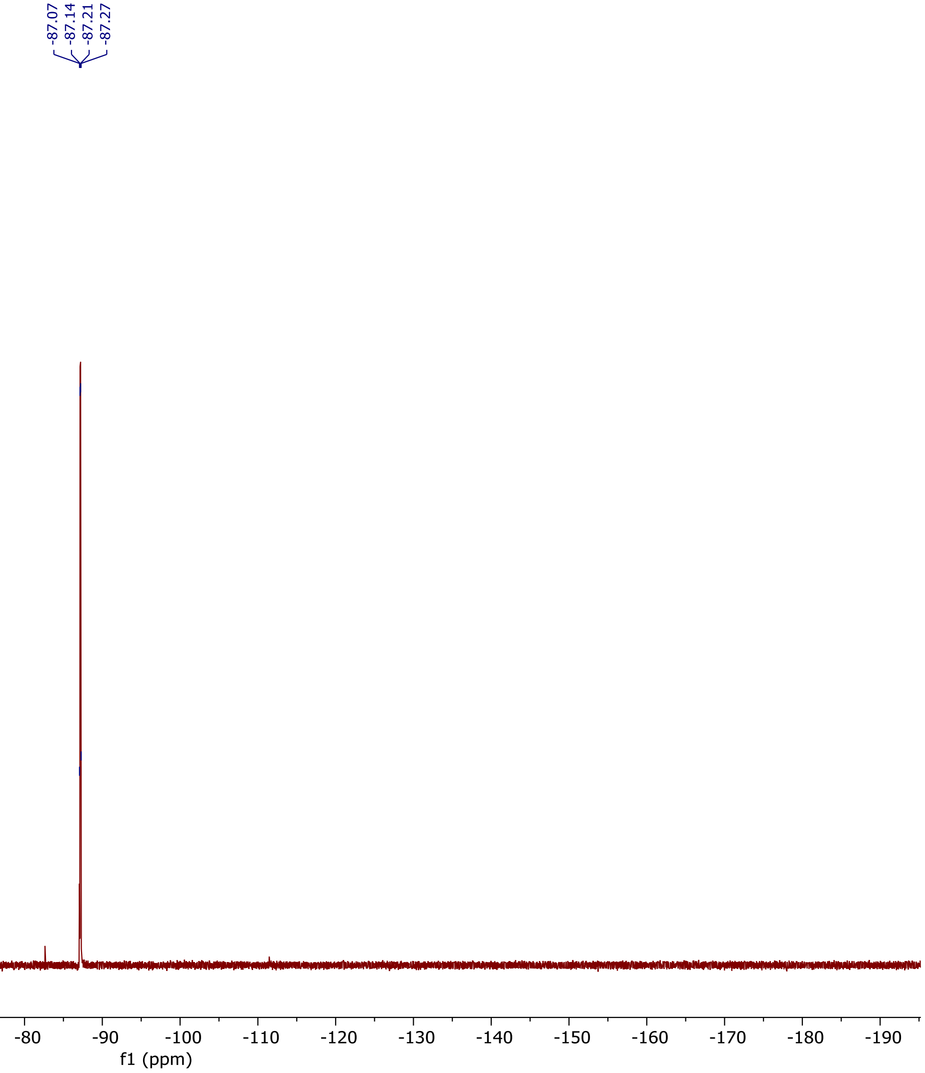

**1,3,7-Trimethyl-8-(perfluoroethyl)-3,7-dihydro-1*H*-purine-2,6-dione 4**

$^1$H NMR (300 MHz, $CDCl_3$)

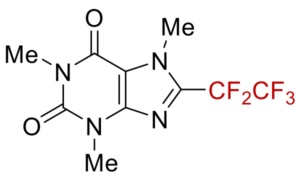

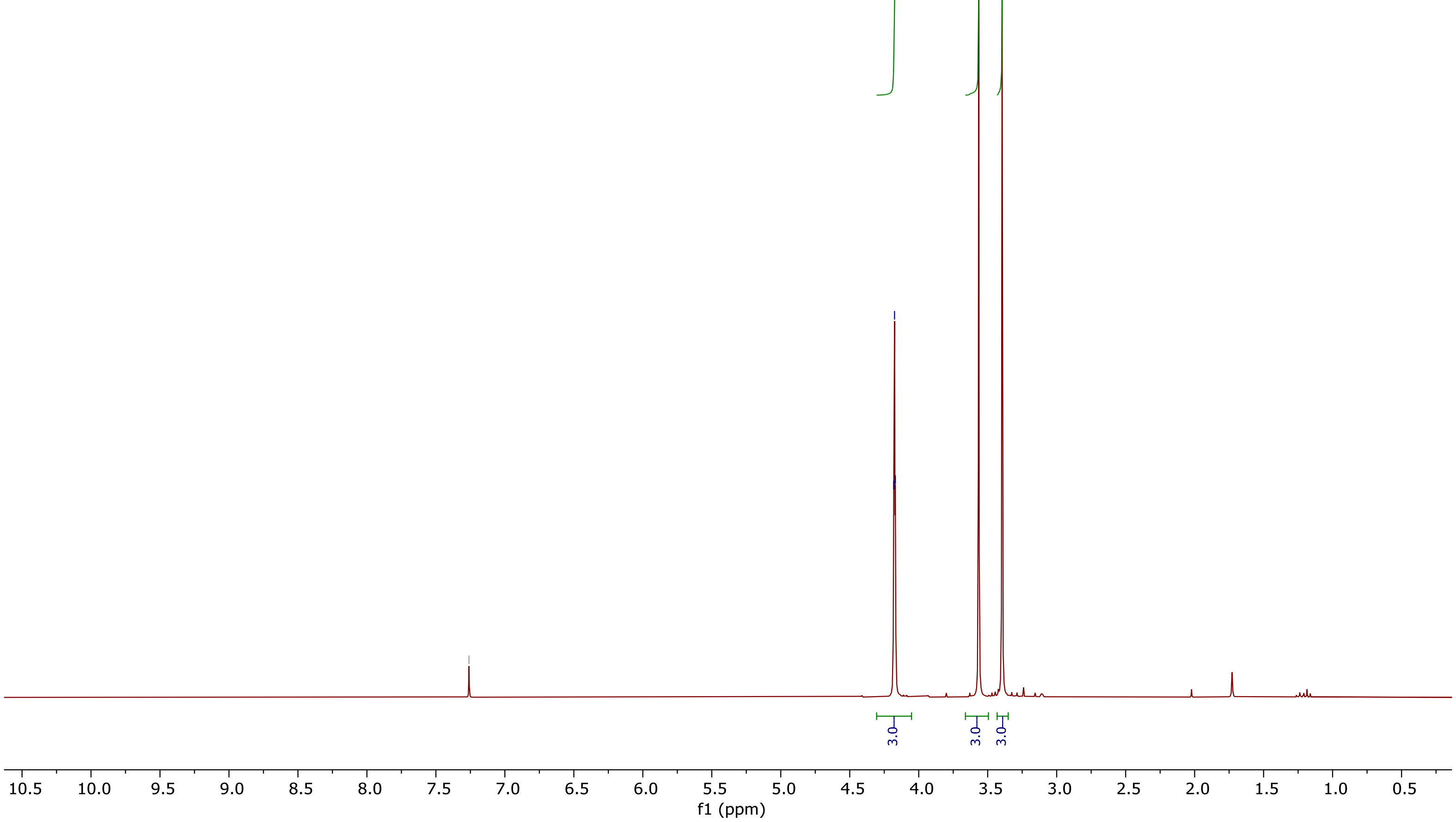

$^{13}C$ NMR (75 MHz, $CDCl_3$)

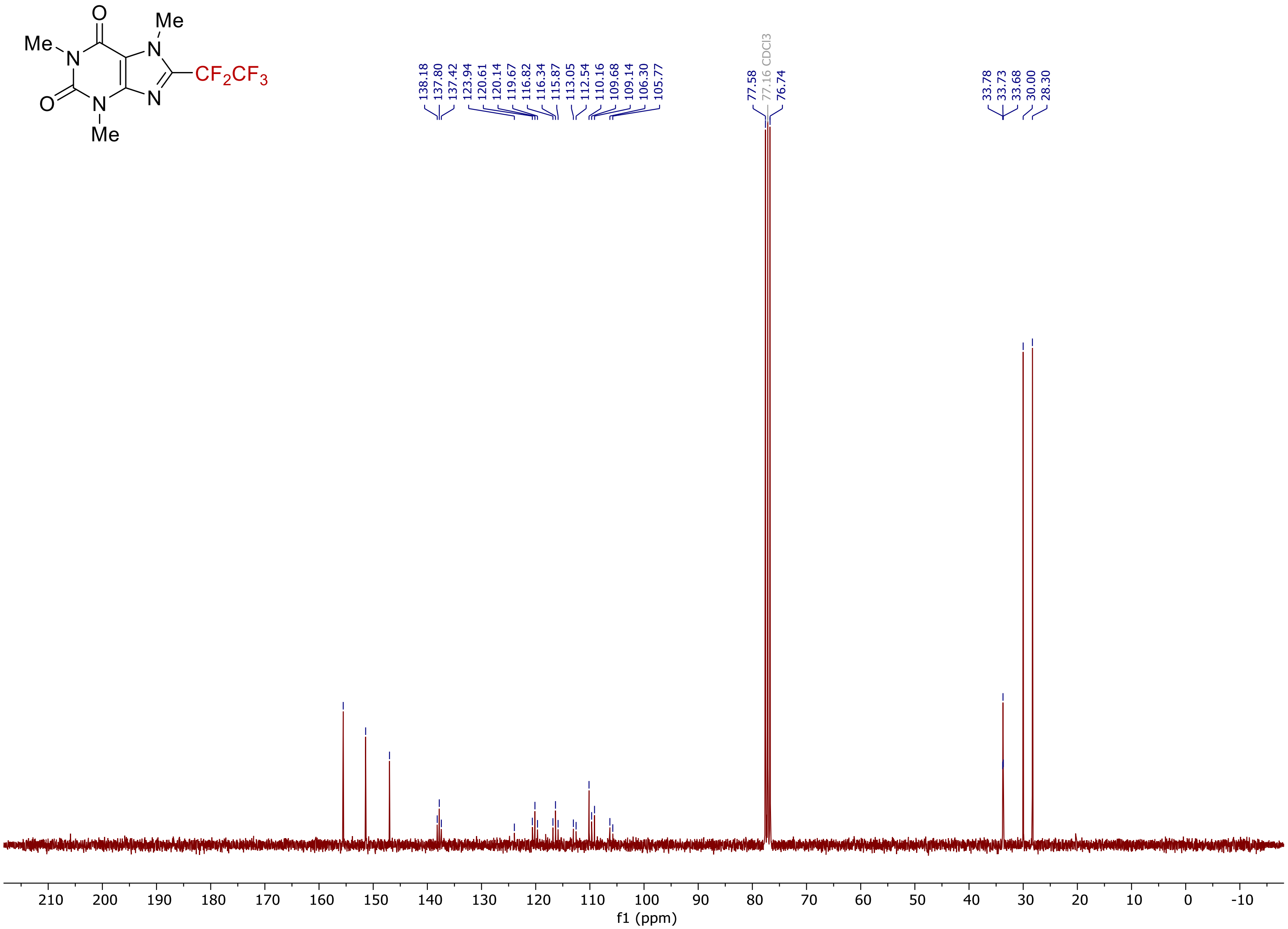

$^{19}F$ NMR (282 MHz, $CDCl_3$)

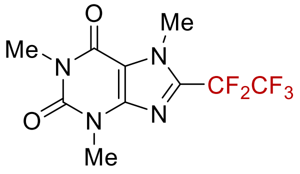


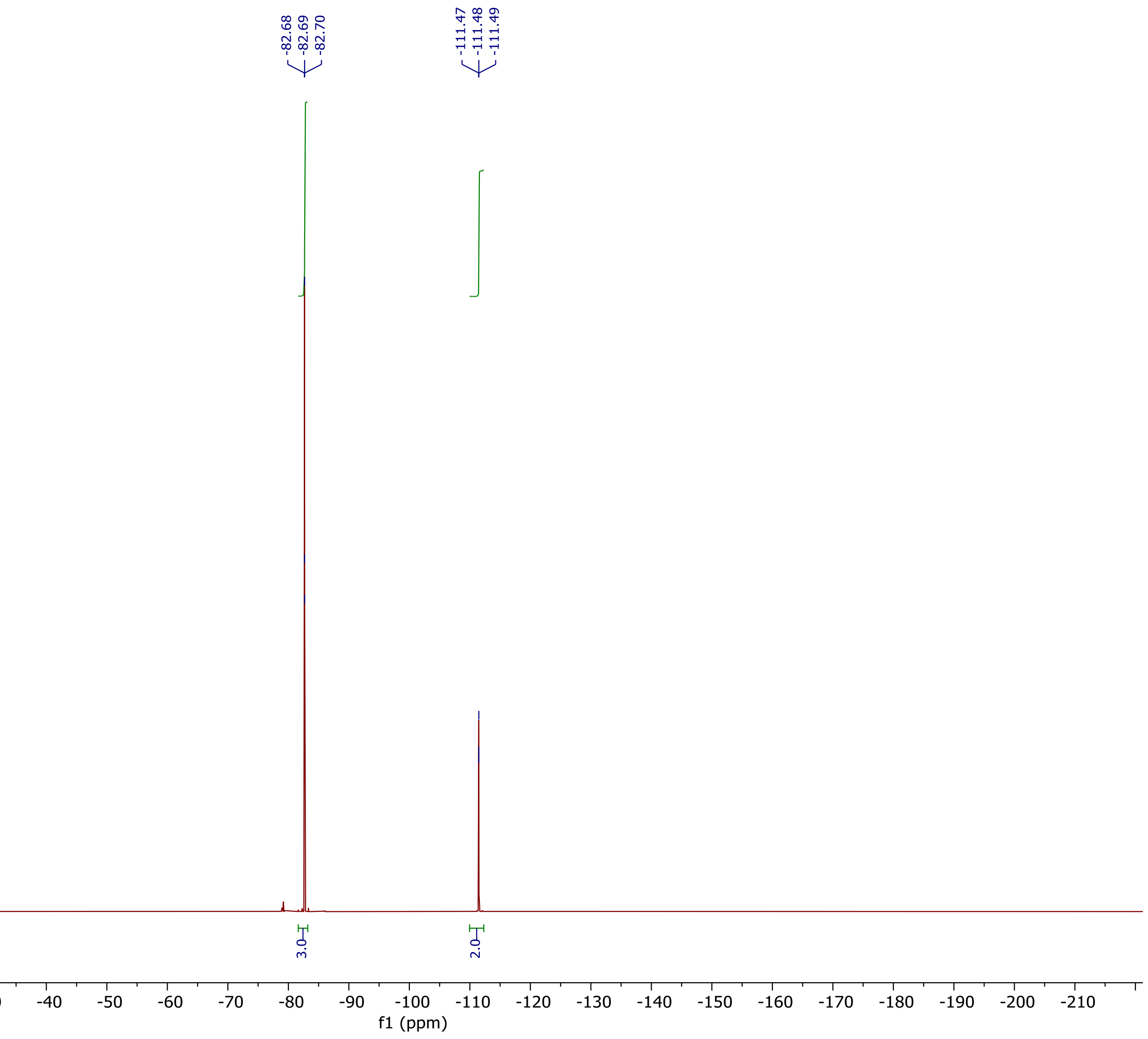

**1,3,7-trimethyl-8-(perfluoropropyl)-3,7-dihydro-1*H*-purine-2,6-dione 5**

$^{1}$H NMR (300 MHz, $CDCl_3$)

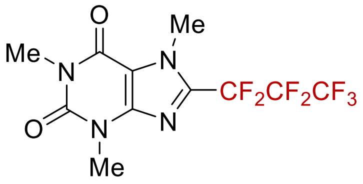


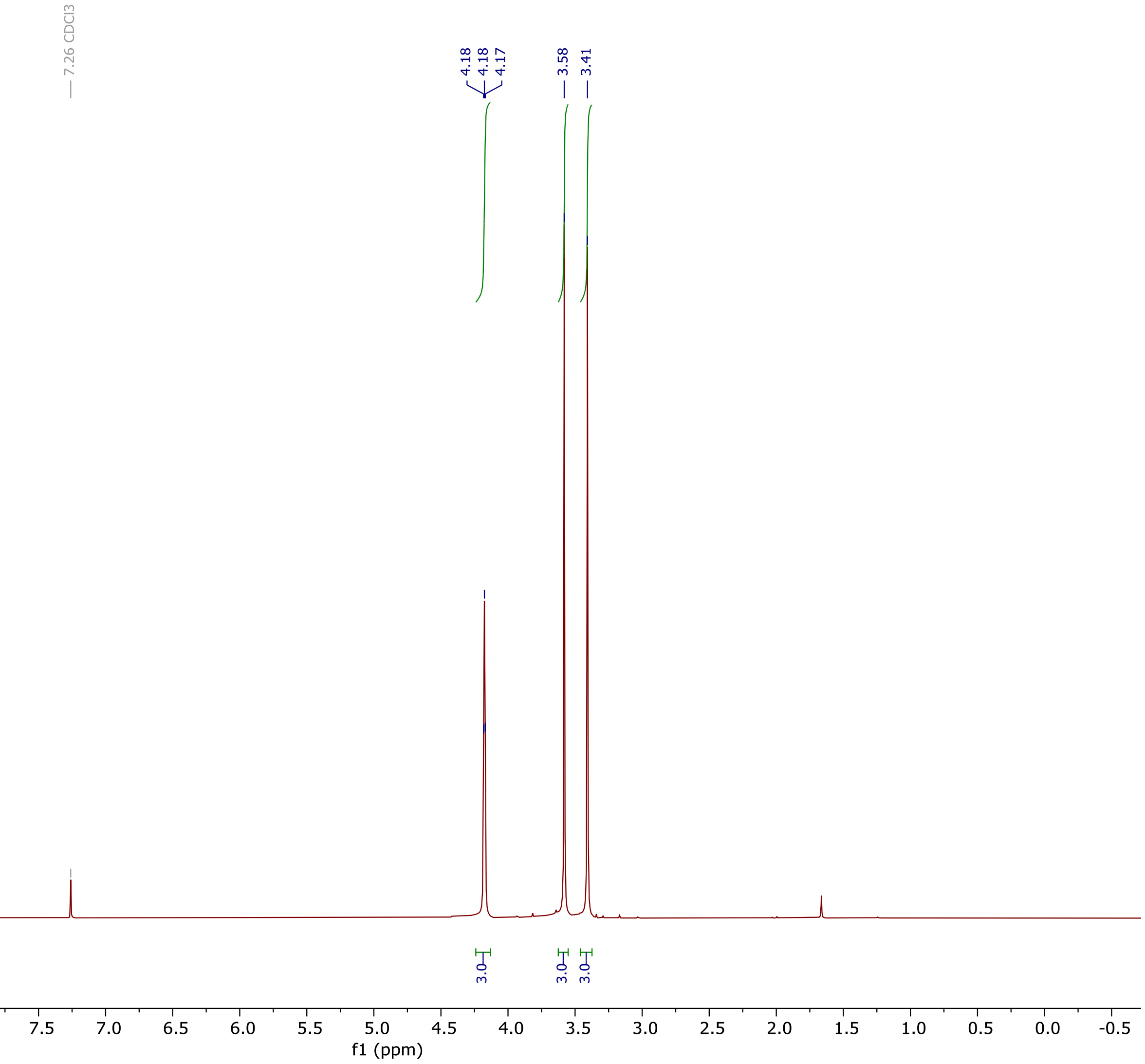

$^{13}C$ NMR (75 MHz, $CDCl_3$)

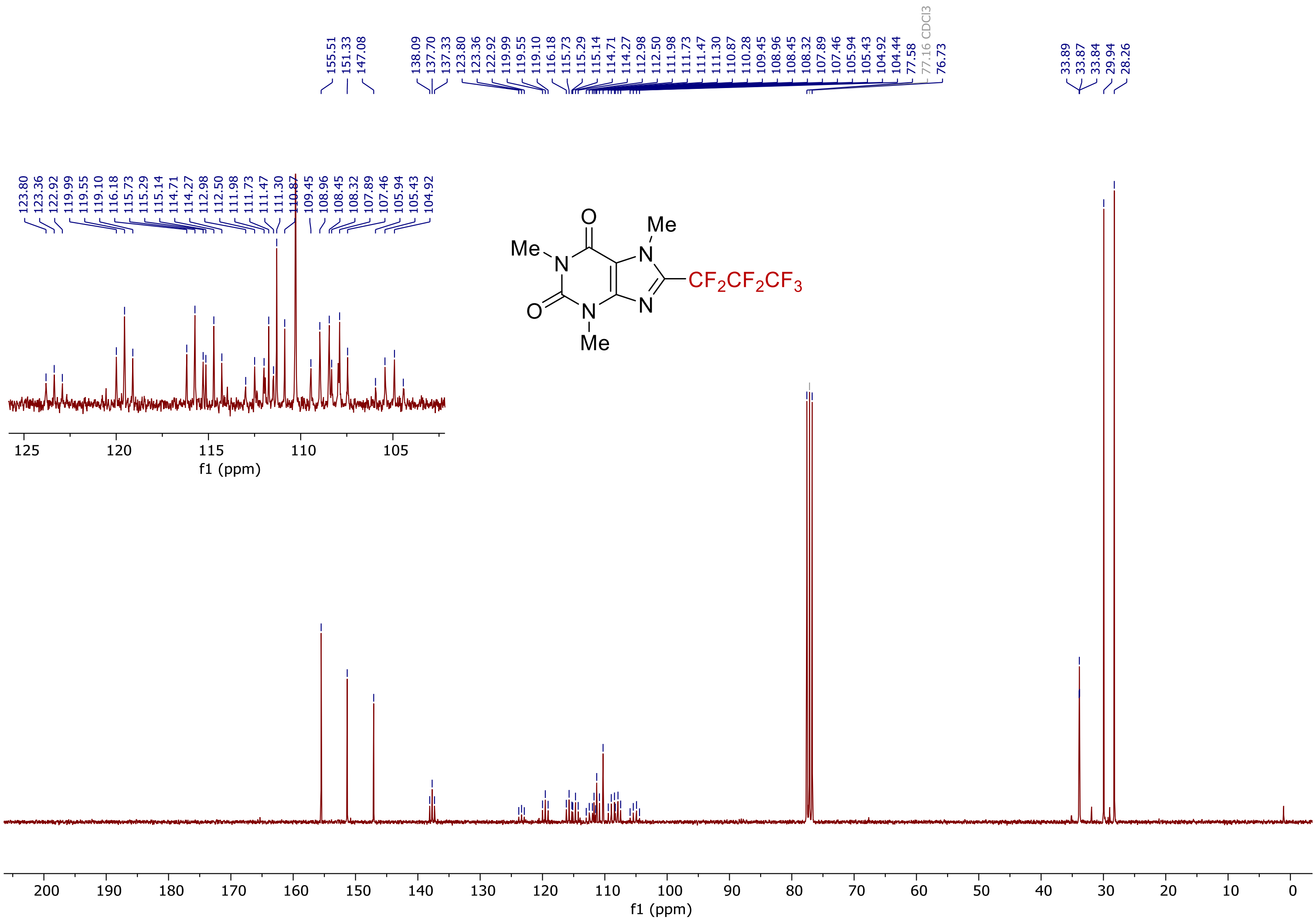

$^{19}$F NMR (282 MHz, $CDCl_3$)

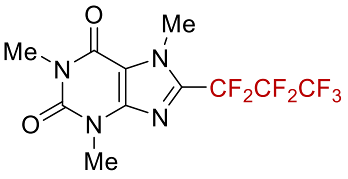


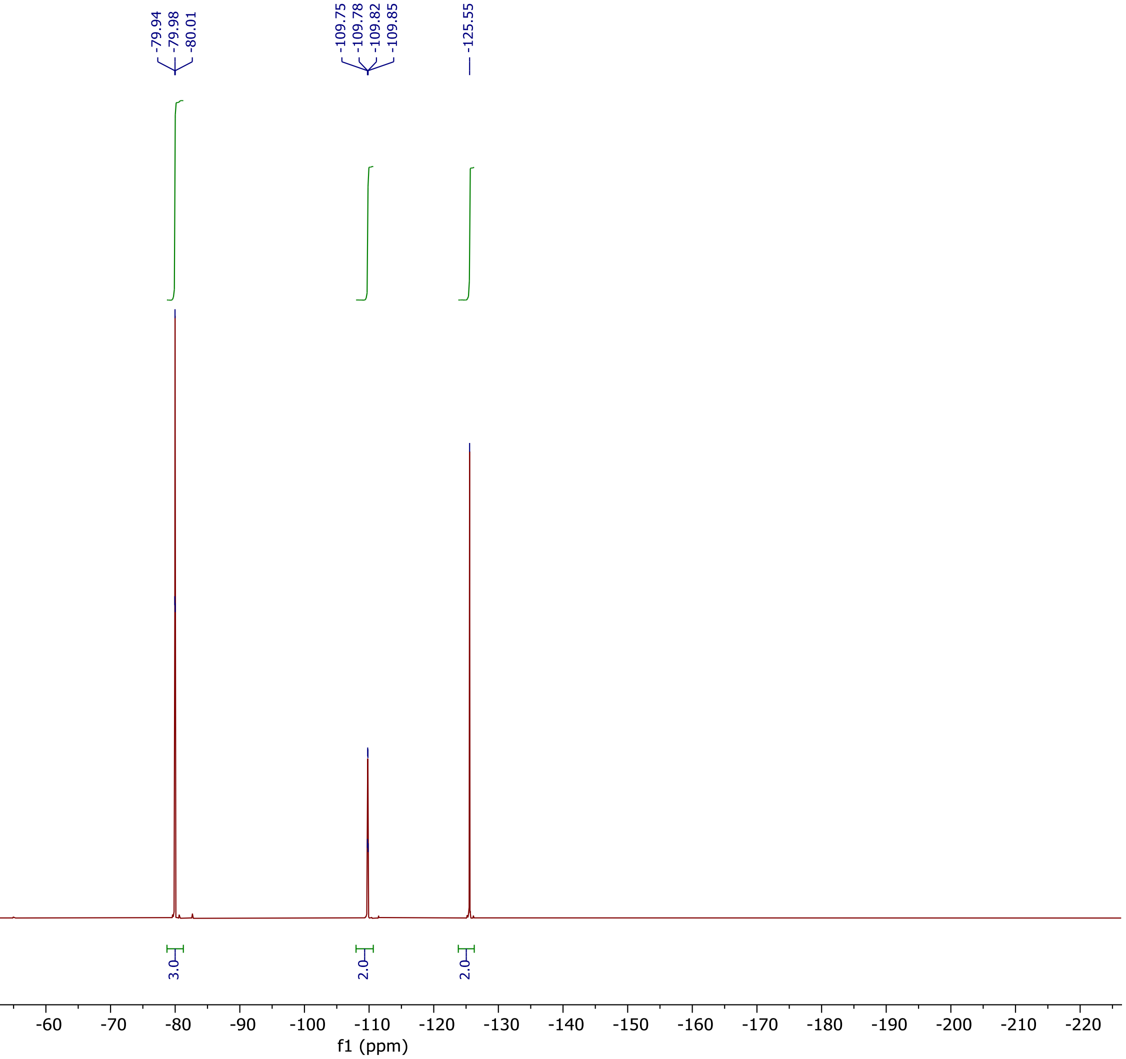

**1,3,7-Trimethyl-8-(1,1,2,2-tetrafluoroethyl)-3,7-dihydro-1*H*-purine-2,6-dione 6**

$^{1}$H NMR (300 MHz, $CDCl_3$)

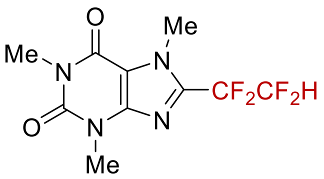


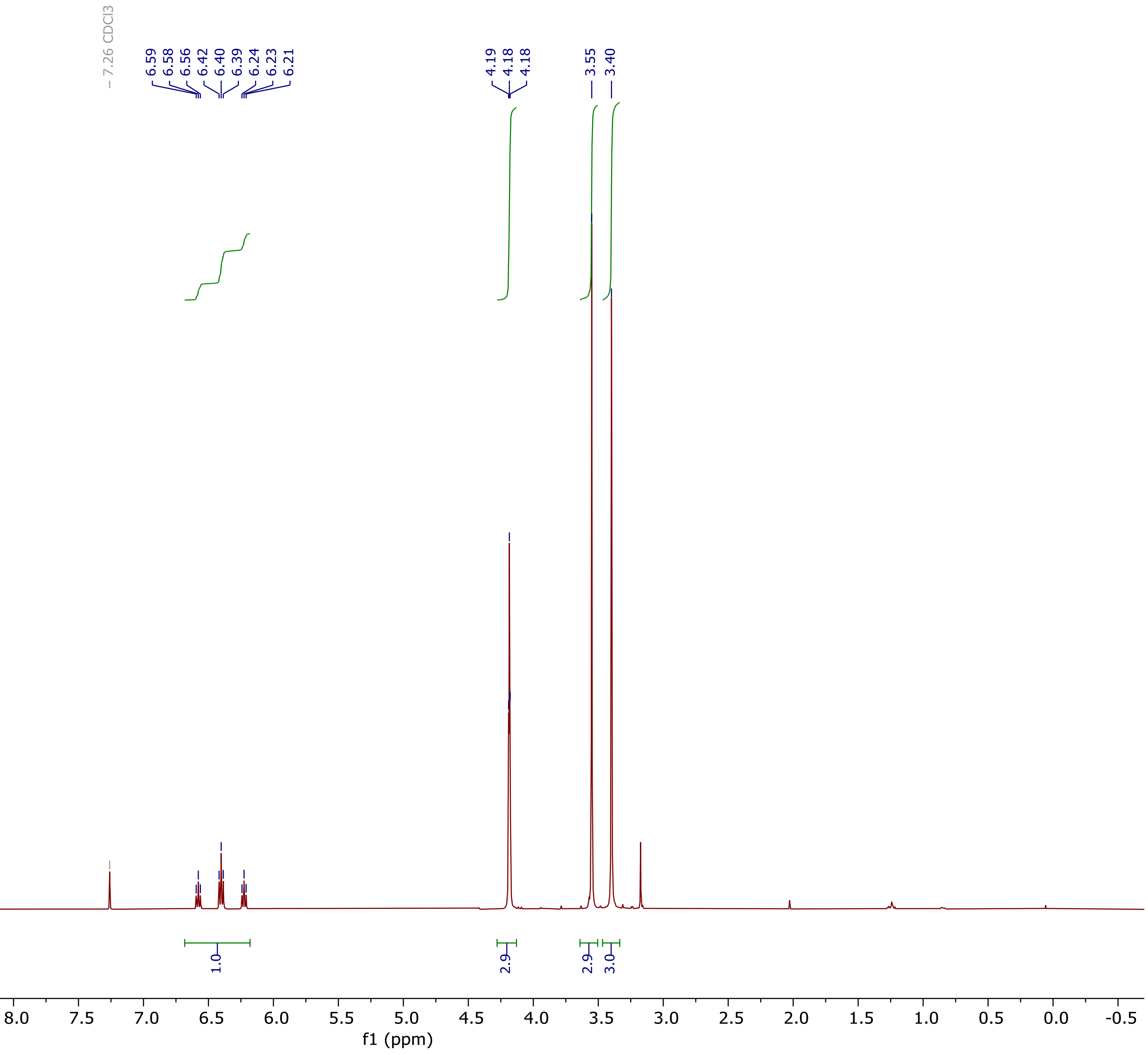

$^{13}C$ NMR (75 MHz, $CDCl_3$)

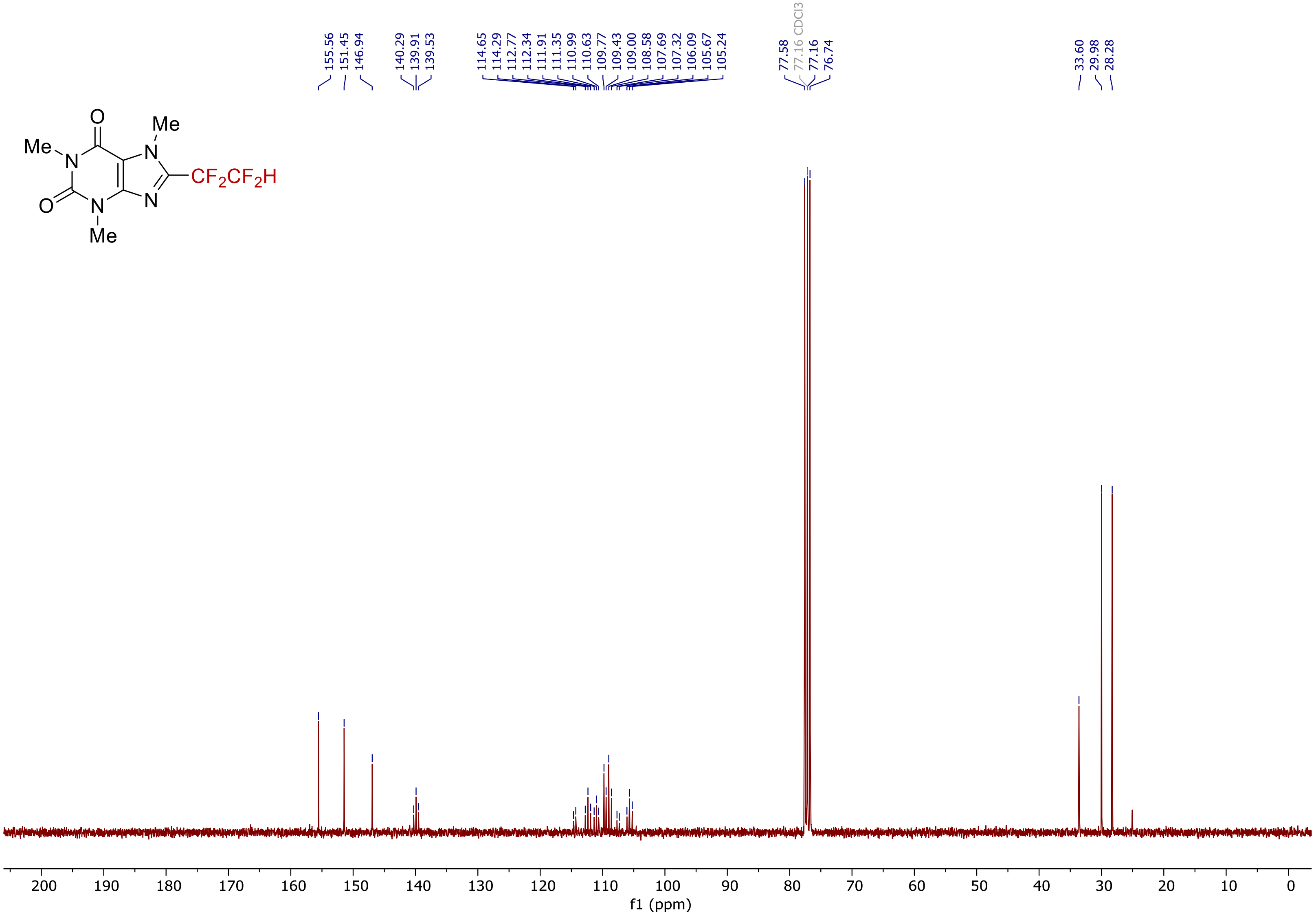

$^{19}$F NMR (282 MHz, $CDCl_3$)

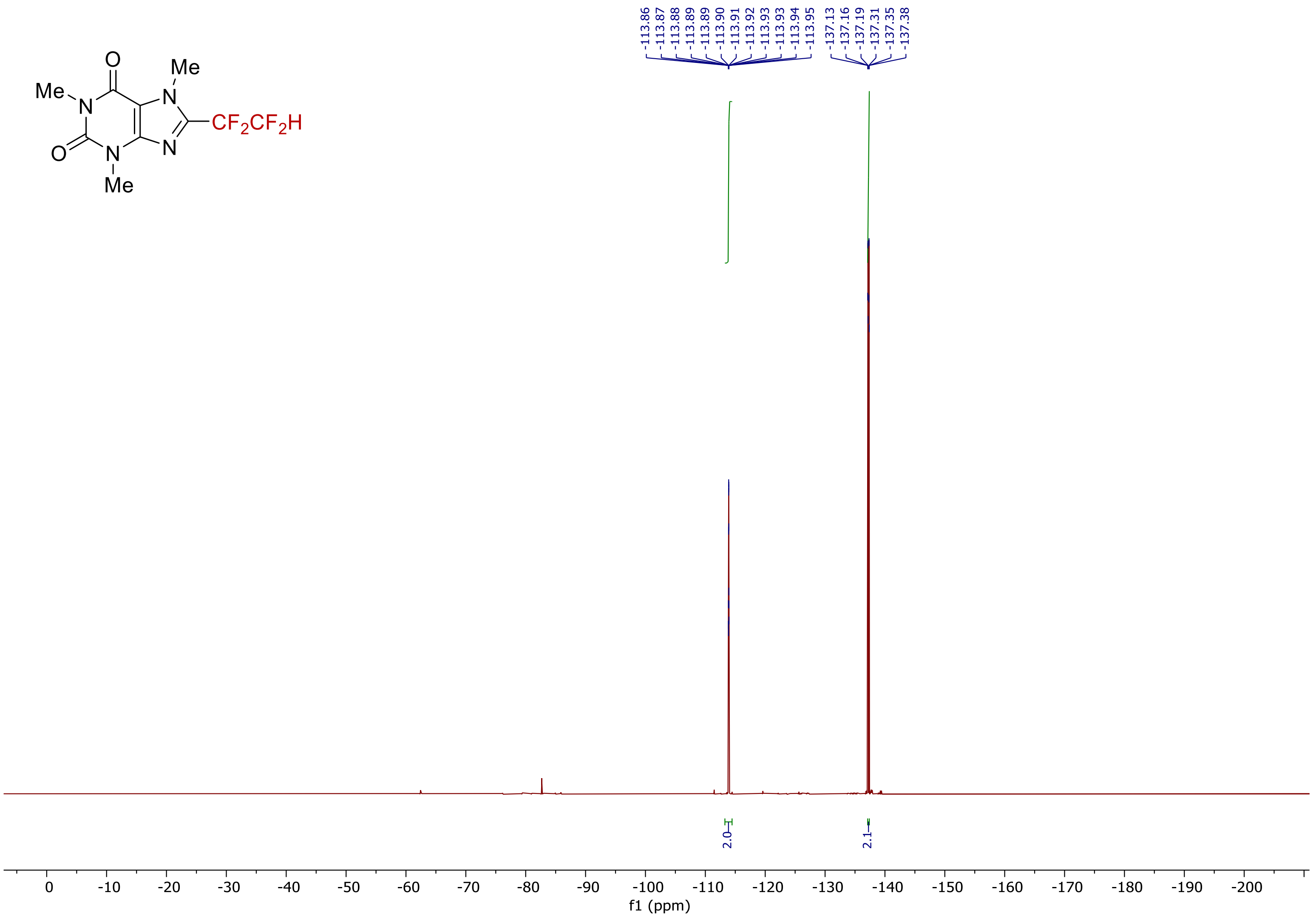

**1,3,7-Trimethyl-8-(trifluoromethyl)-3,7-dihydro-1H-purine-2,6-dione 7**

$^{1}$H NMR (300 MHz, $CDCl_3$)

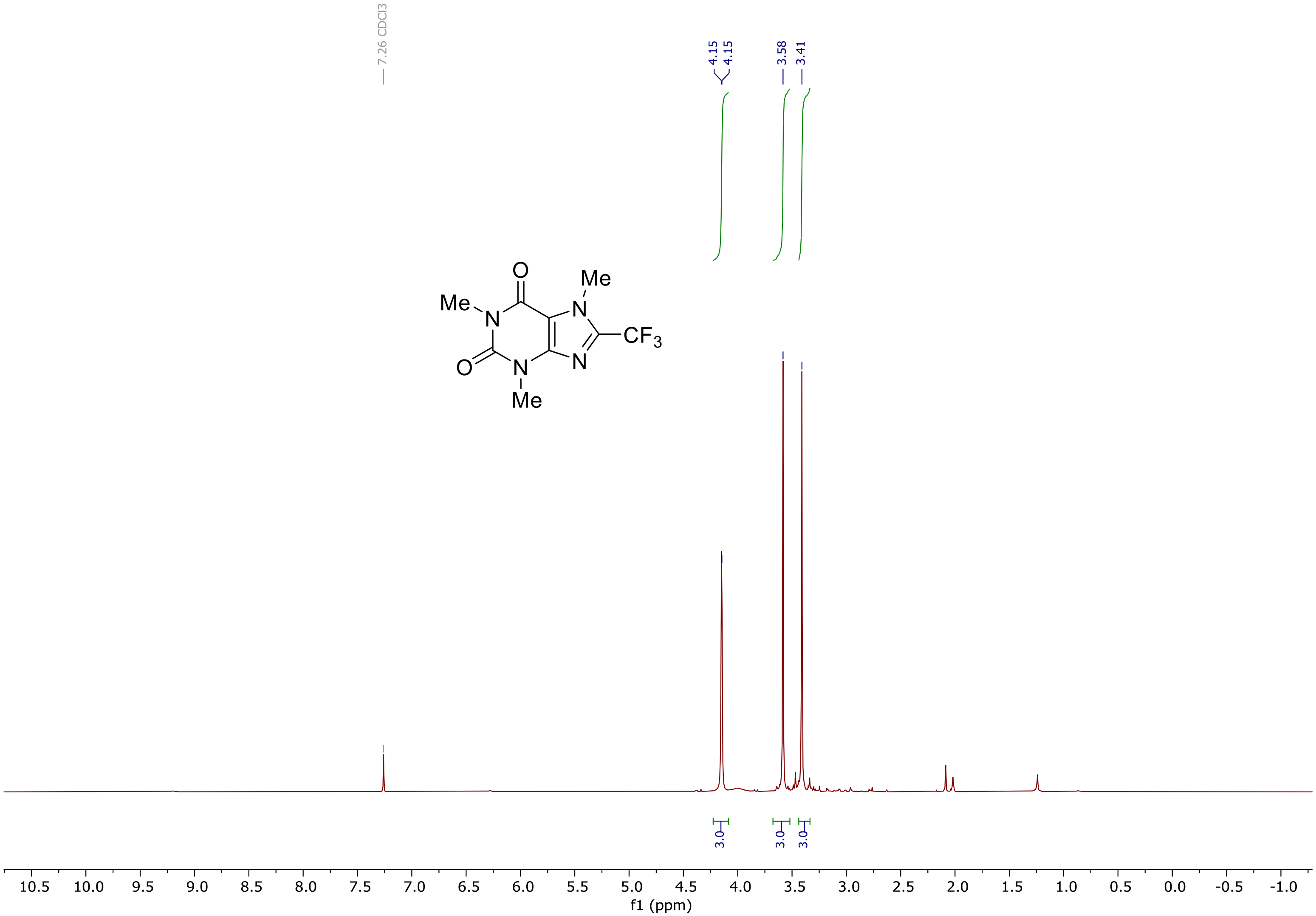

**8-(1,1-Difluoropropyl)-1,3,7-trimethyl-3,7-dihydro-1*H*-purine-2,6-dione 8**

$^1$H NMR (300 MHz, $CDCl_3$)

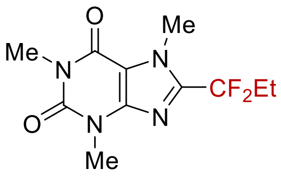


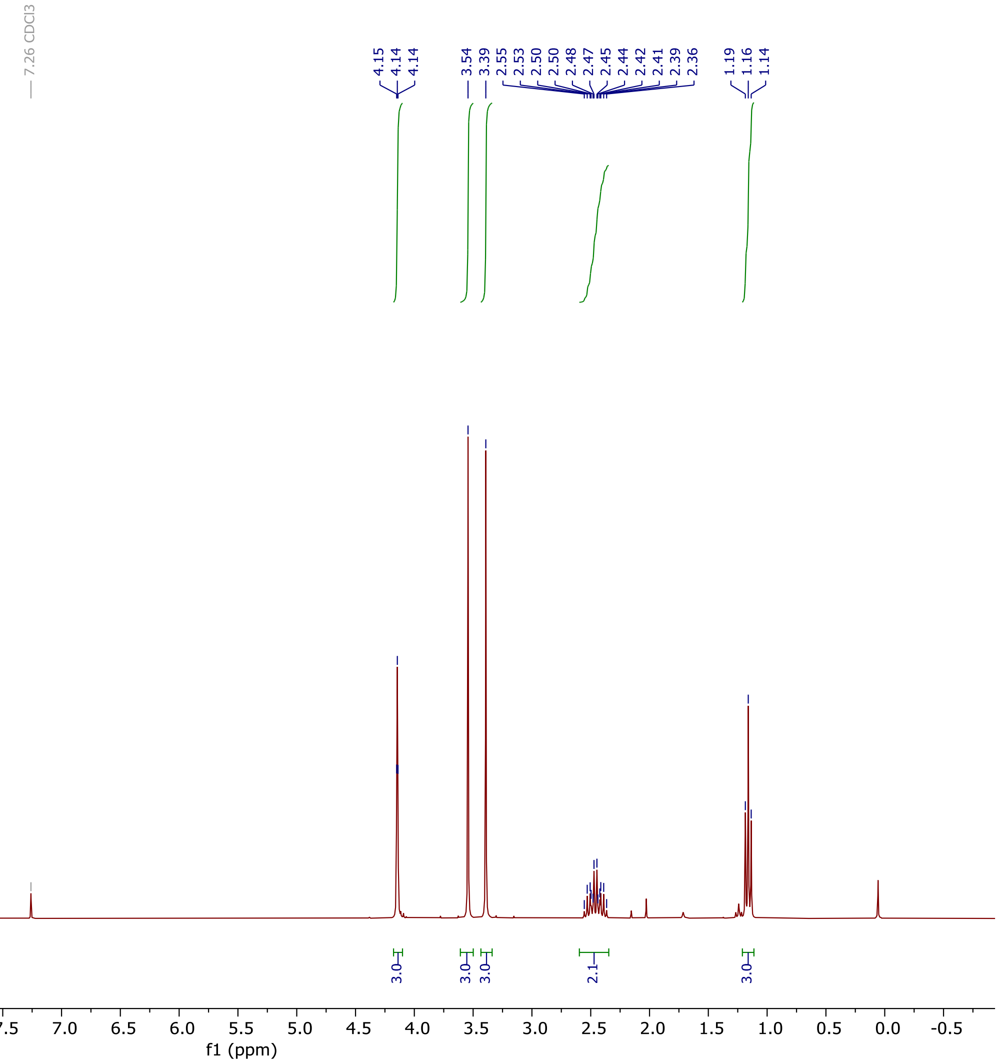

$^{13}C$ NMR (75 MHz, $CDCl_3$)

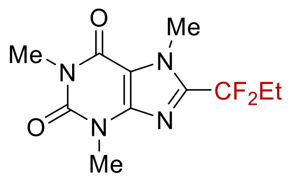


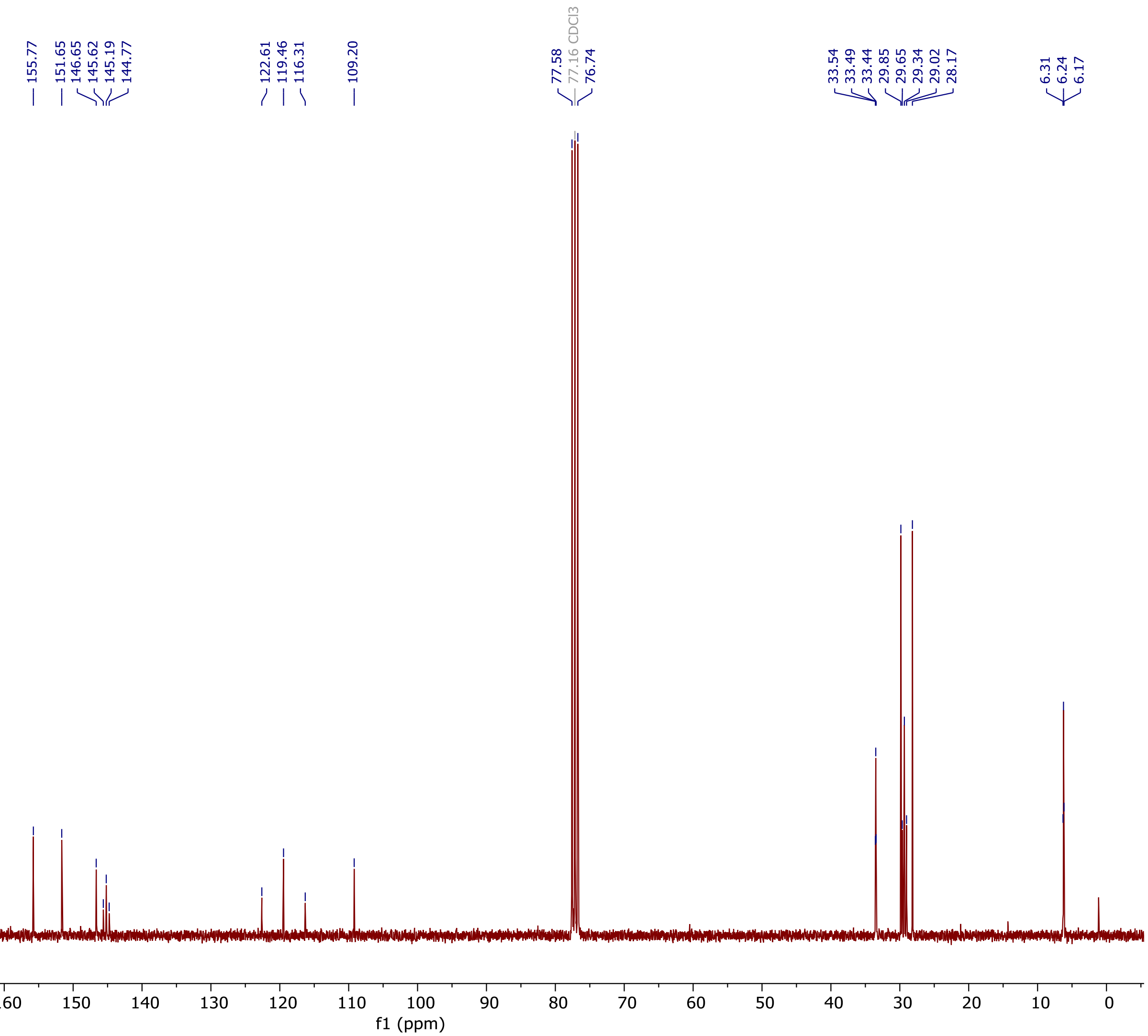

$^{19}F$ NMR (282 MHz, $CDCl_3$)

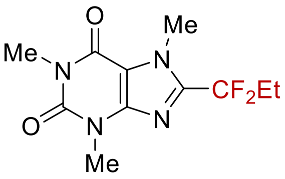

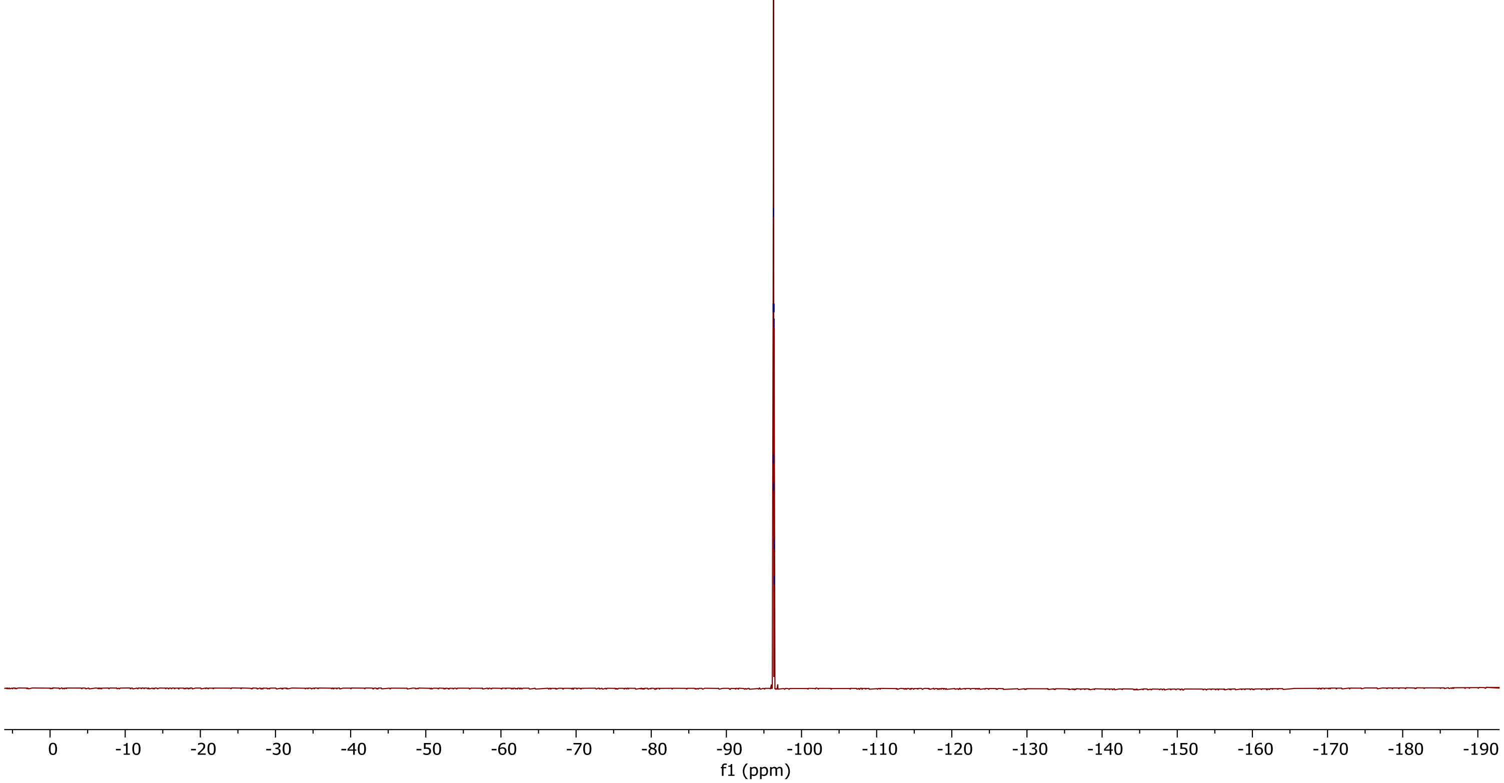

**8-(Difluoro(phenyl)methyl)-1,3,7-trimethyl-3,7-dihydro-1*H*-purine-2,6-dione 9**

$^{1}$H NMR (300 MHz, $CDCl_3$)

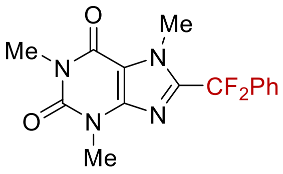


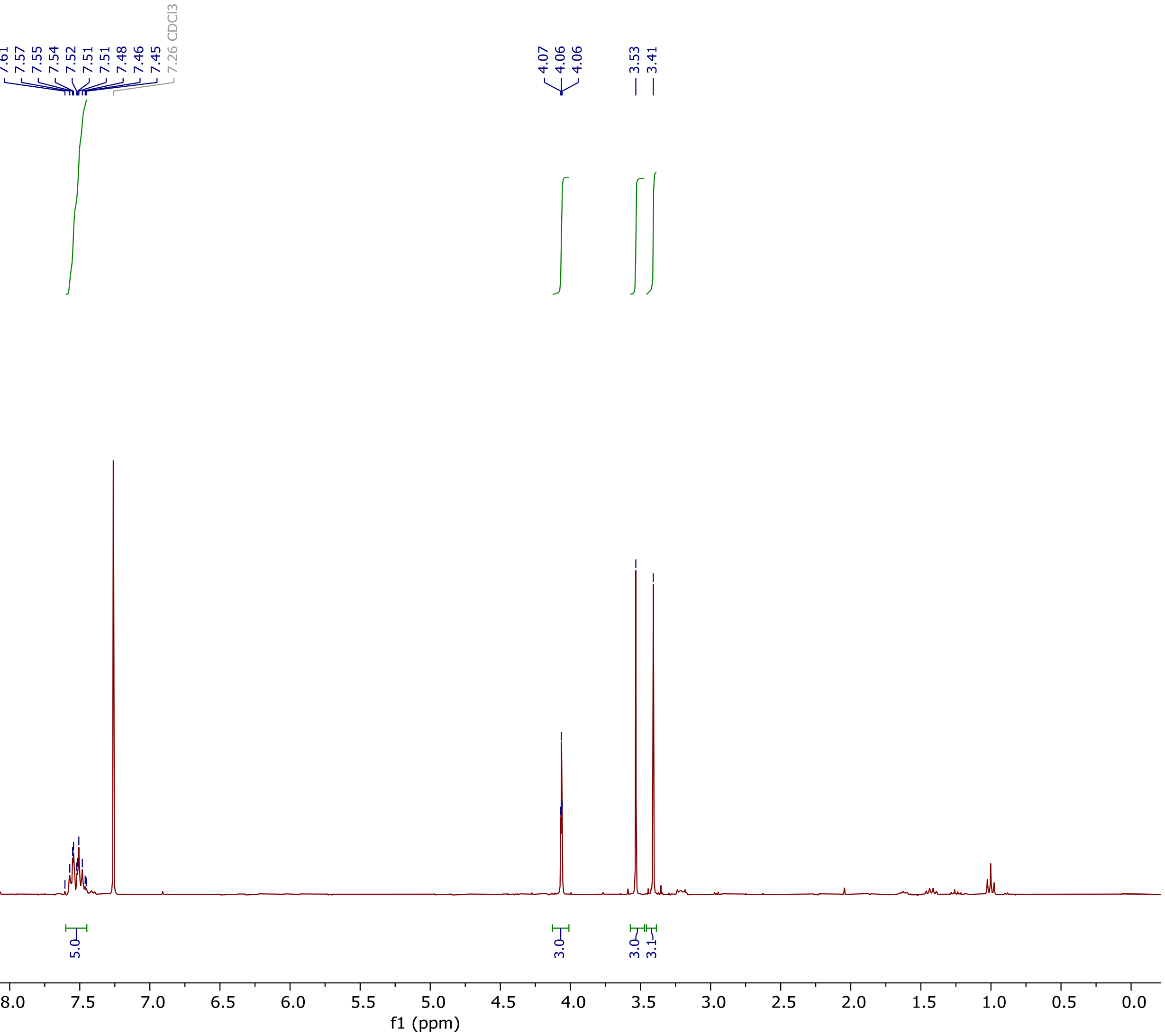

$^{13}C$ NMR (75 MHz, $CDCl_3$)

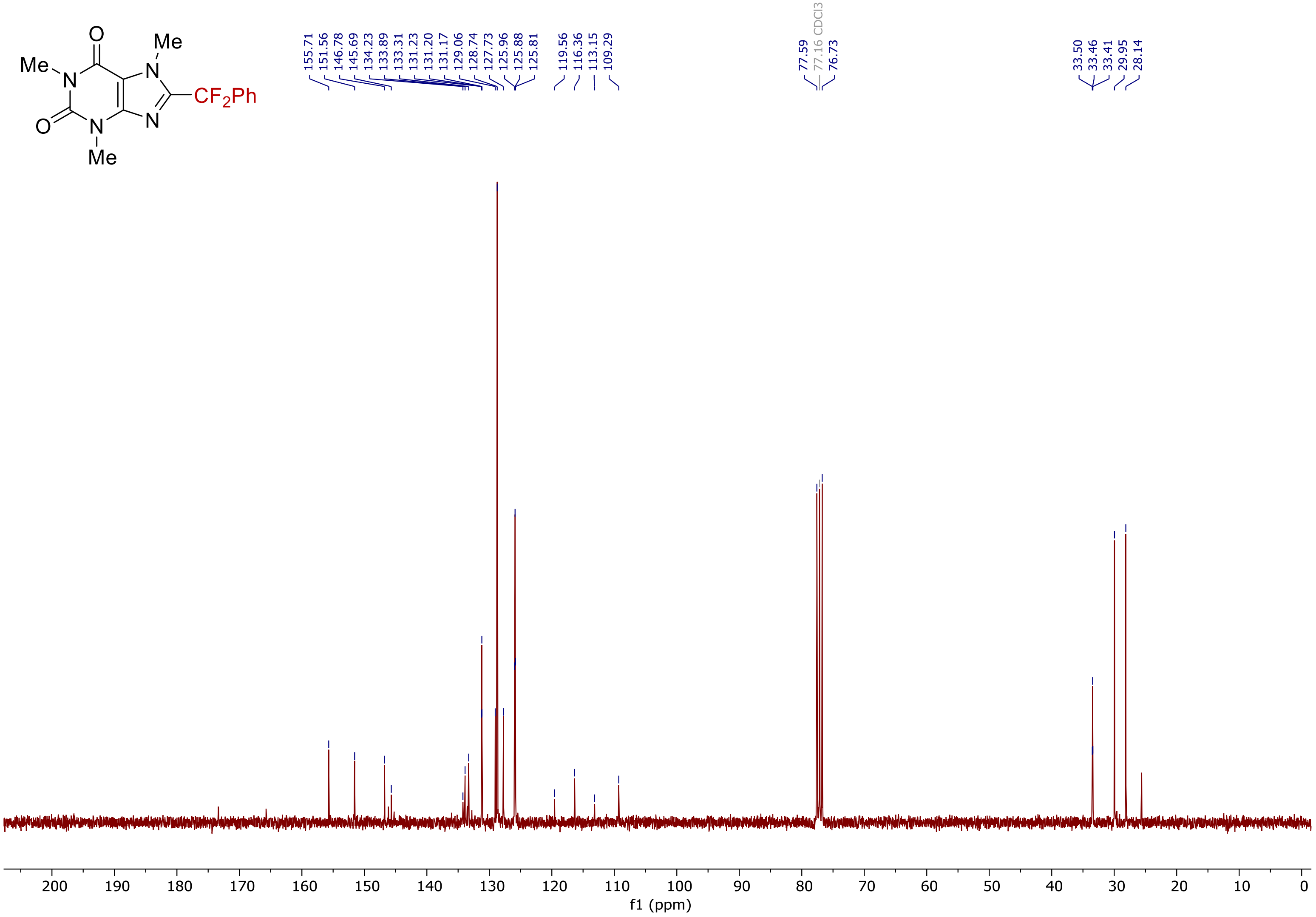

$^{19}$F NMR (282 MHz, $CDCl_3$)

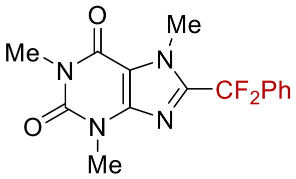


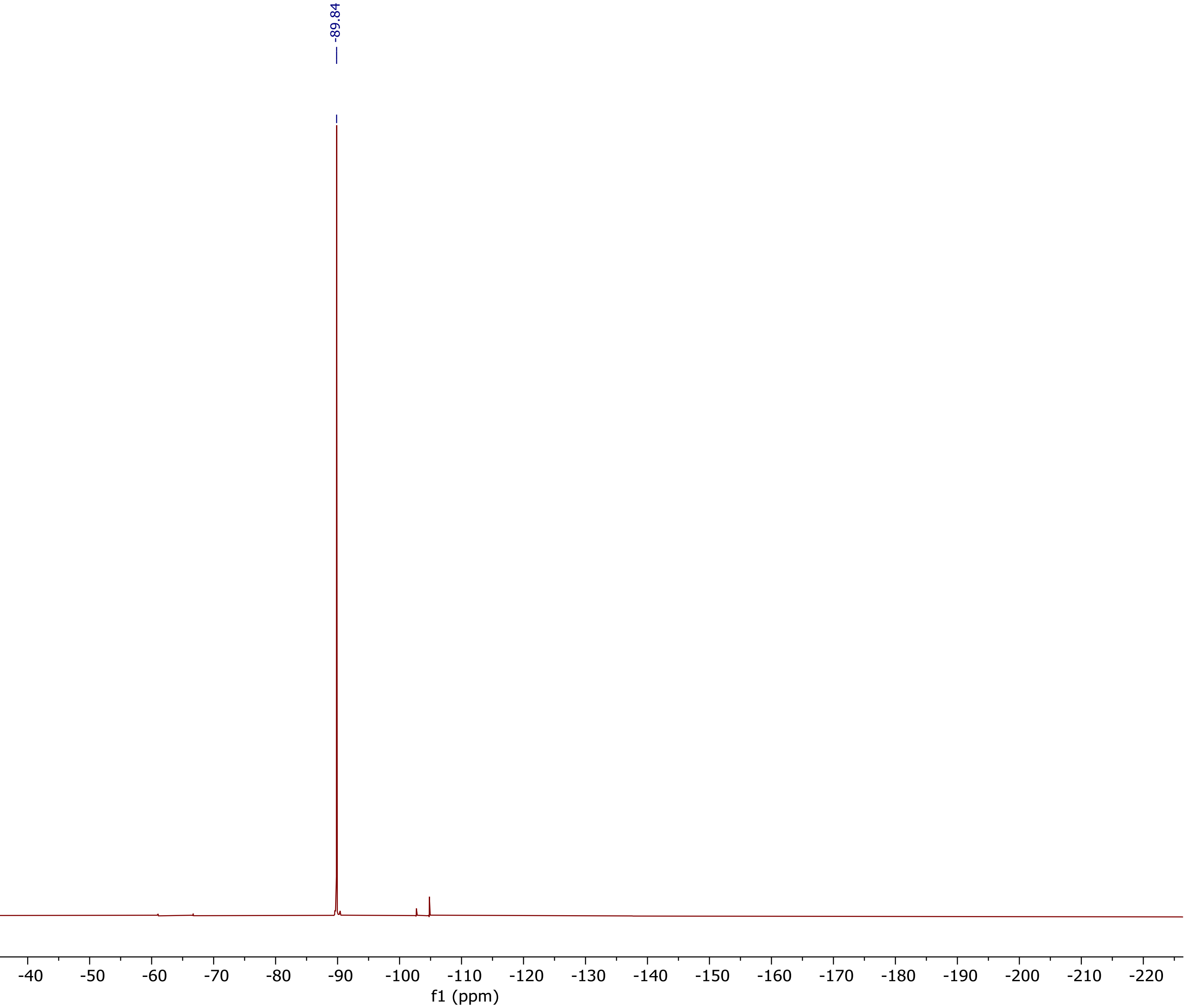

**2,4-Dimethyl-6-(perfluorohexyl)-1,2,4-triazine-3,5(2*H*,4*H*)-dione 10**

$^1$H NMR (600 MHz, $CDCl_3$)

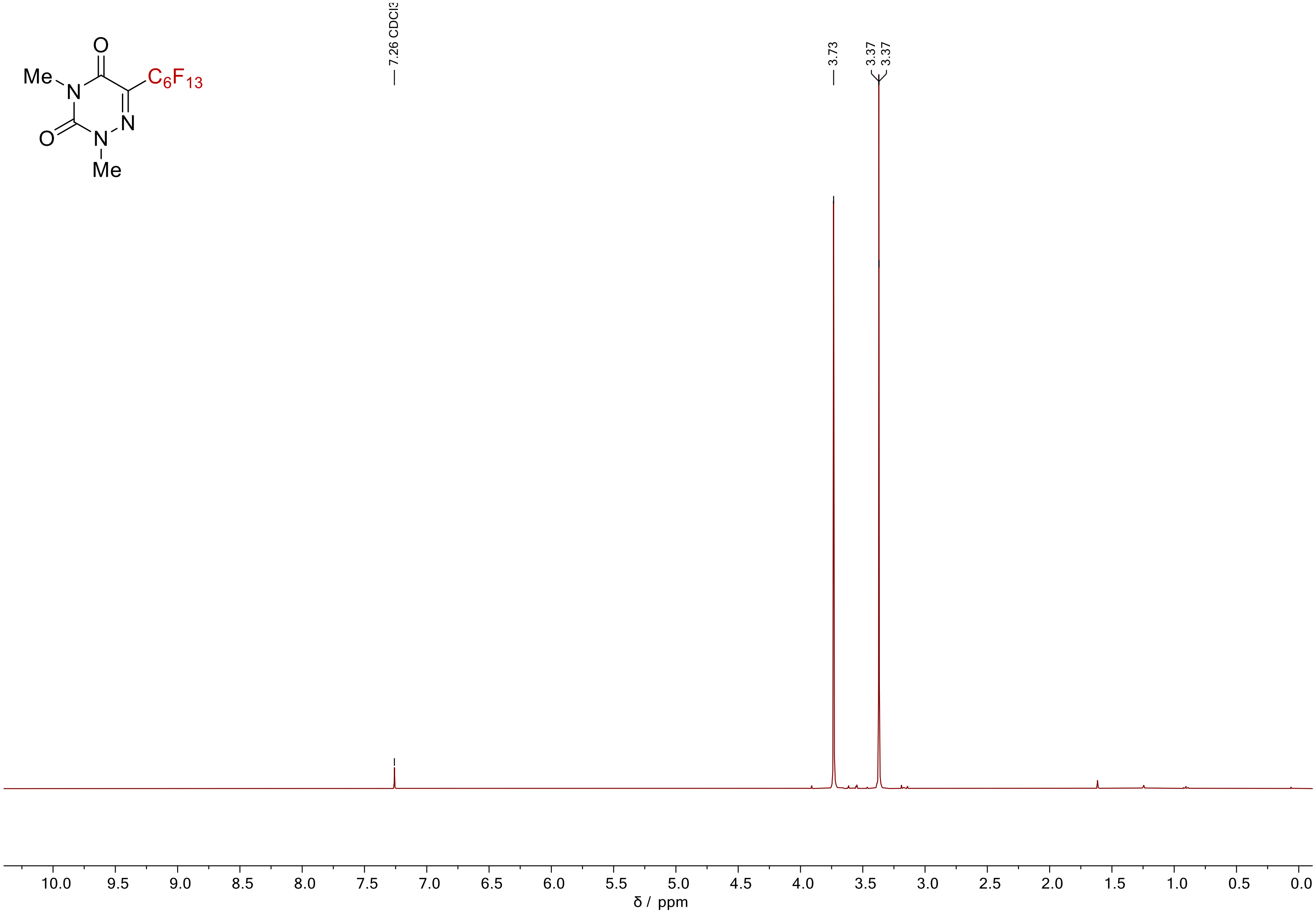

$^{13}C$ NMR (151 MHz, $CDCl_3$)

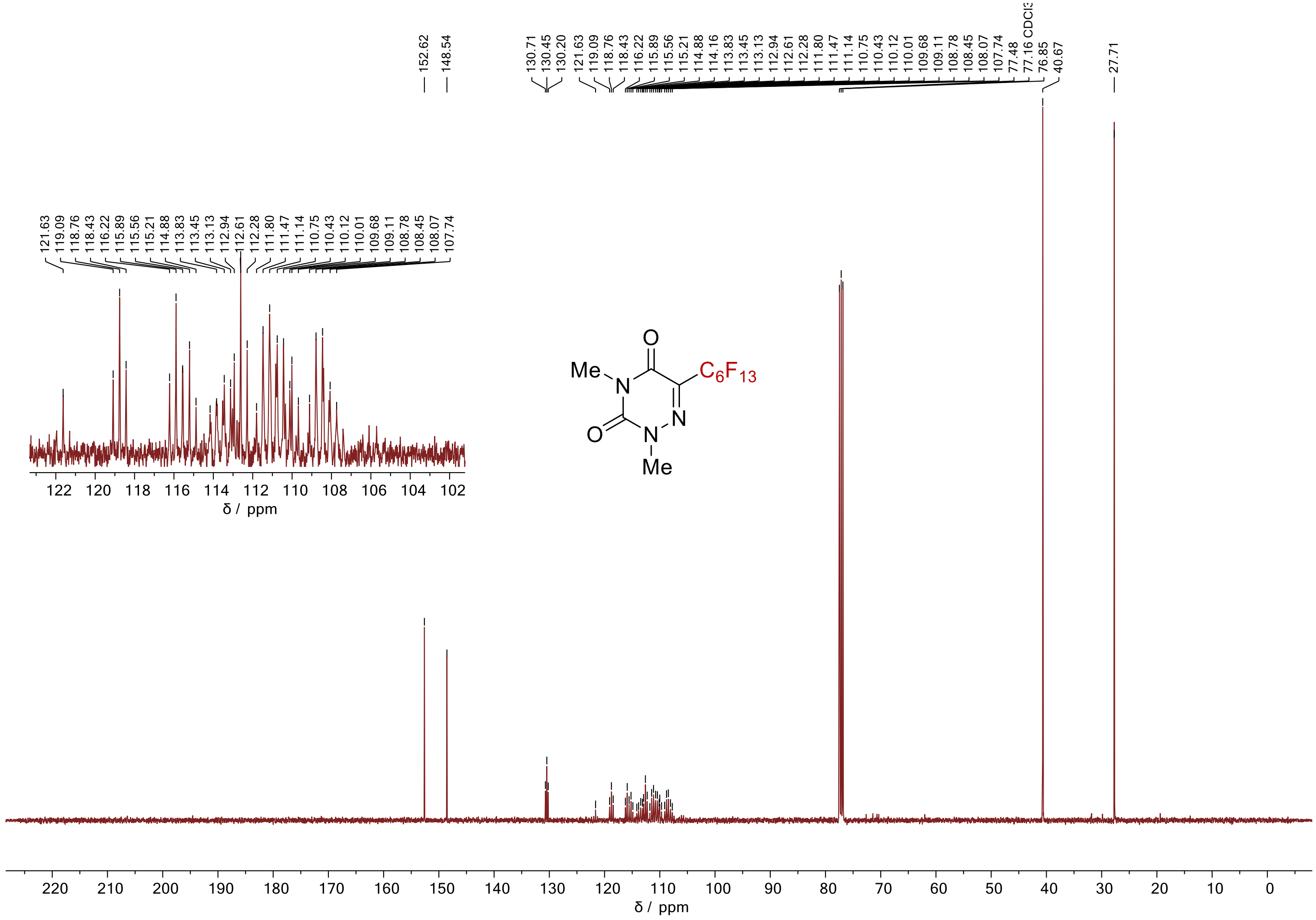

$^{19}$F NMR (565 MHz, $CDCl_3$)

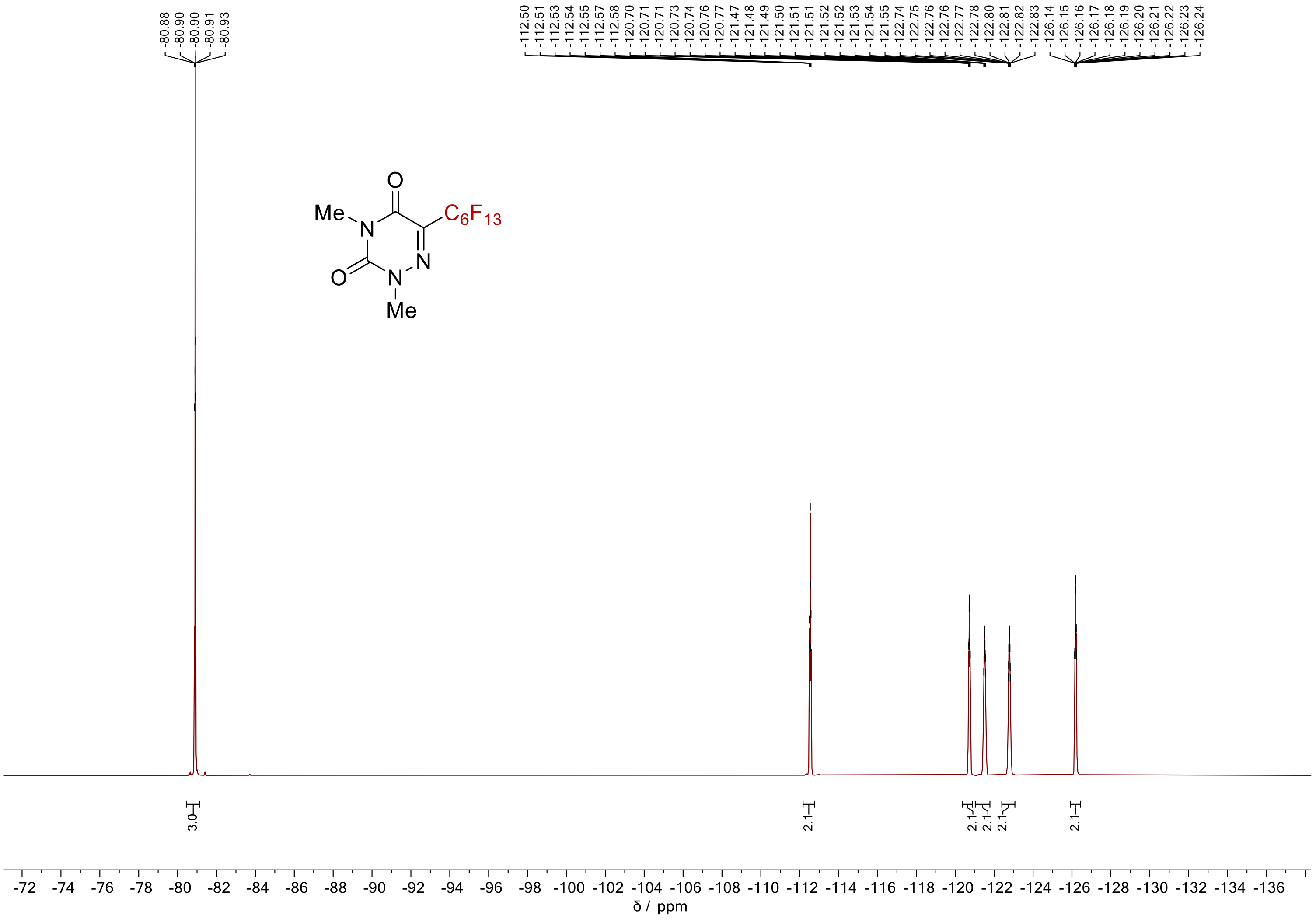

**2,4-Dimethyl-6-(1,1,2,2-tetrafluoroethyl)-1,2,4-triazine-3,5(2*H*,4*H*)-dione 11**

$^{1}H$ NMR (400 MHz, $CDCl_3$)

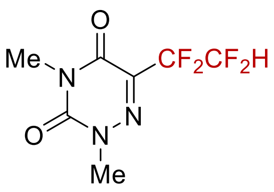


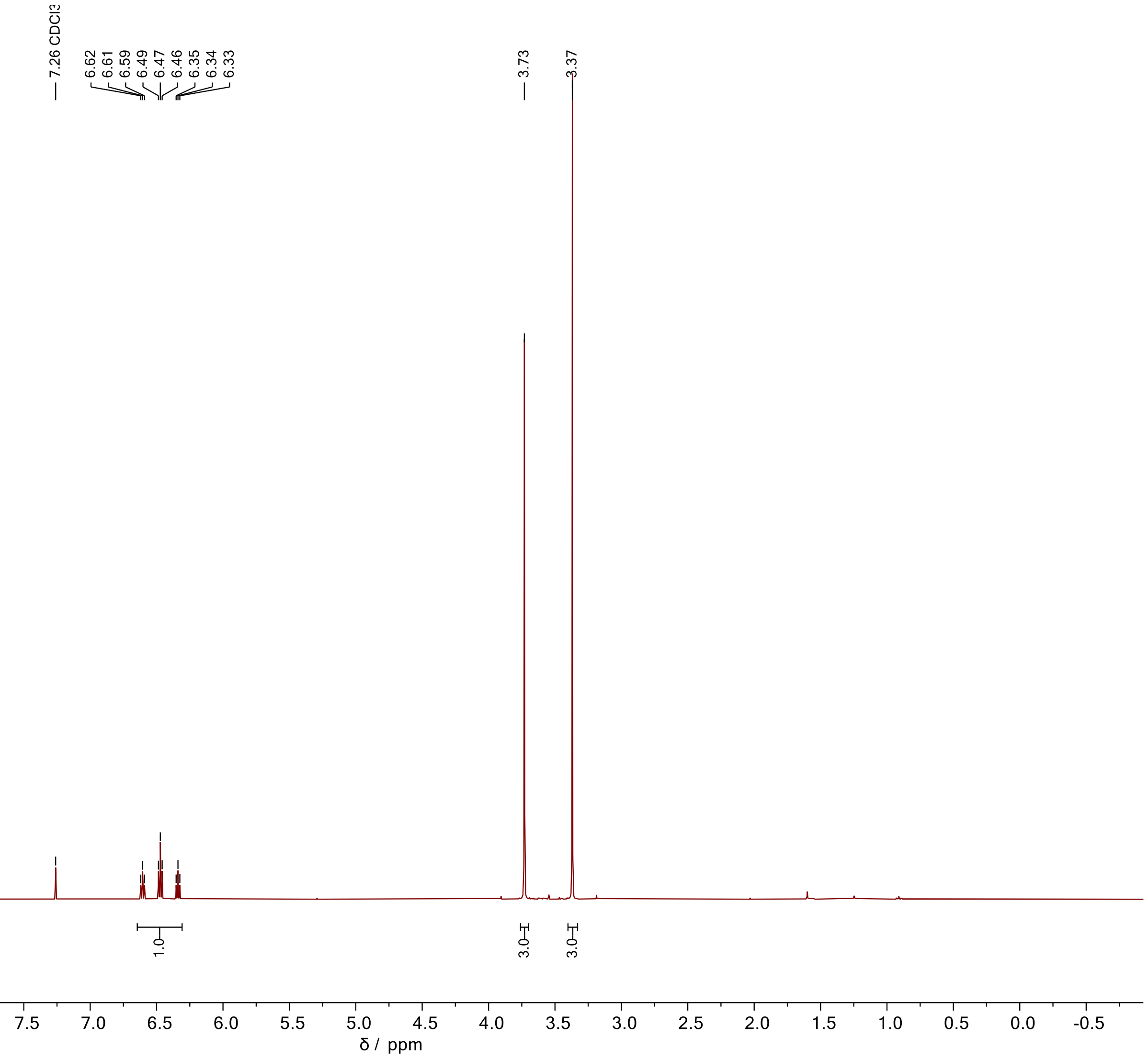

$^{13}C$ NMR (101 MHz, $CDCl_3$)

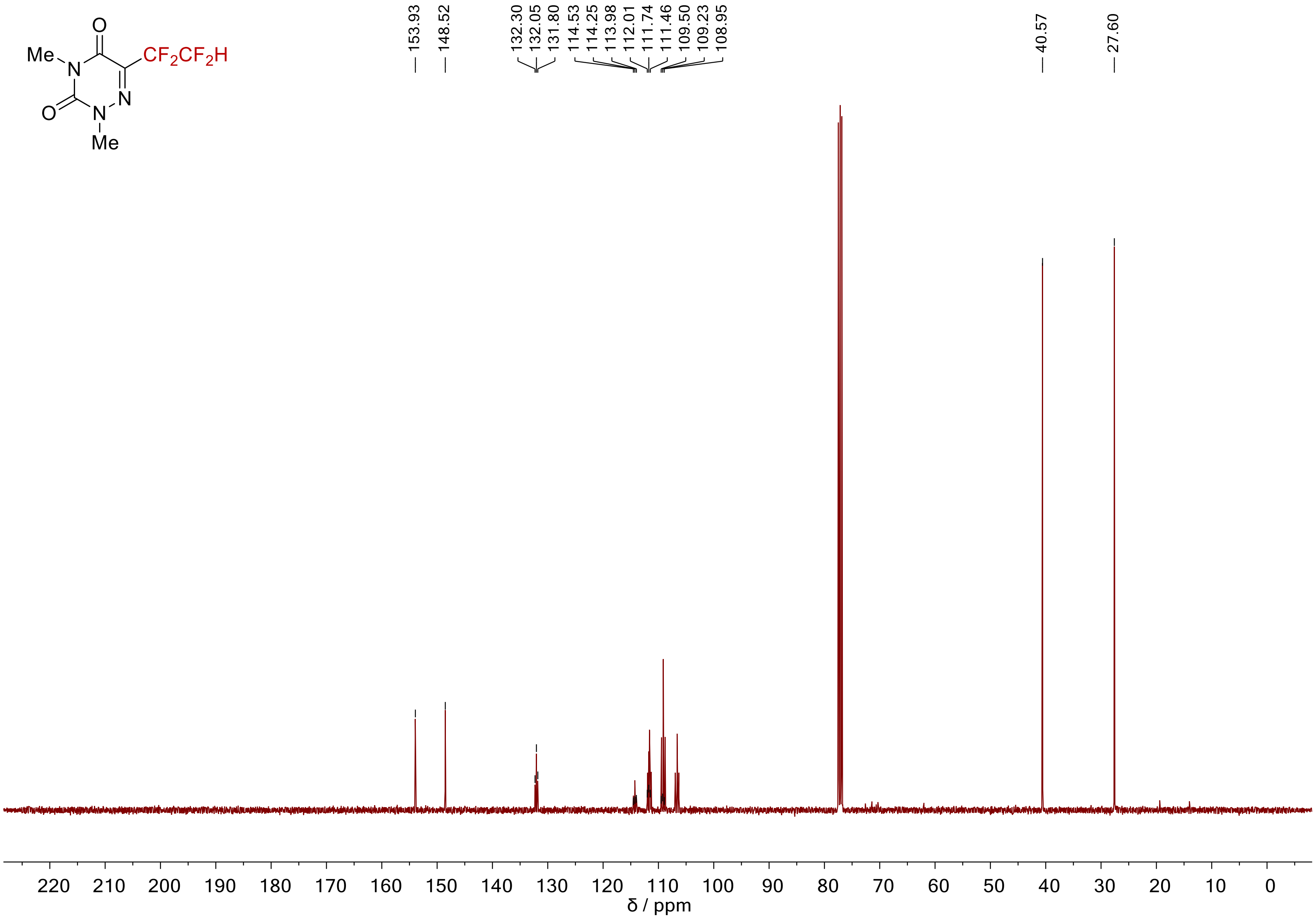

$^{19}$F NMR (377 MHz, $CDCl_3$)

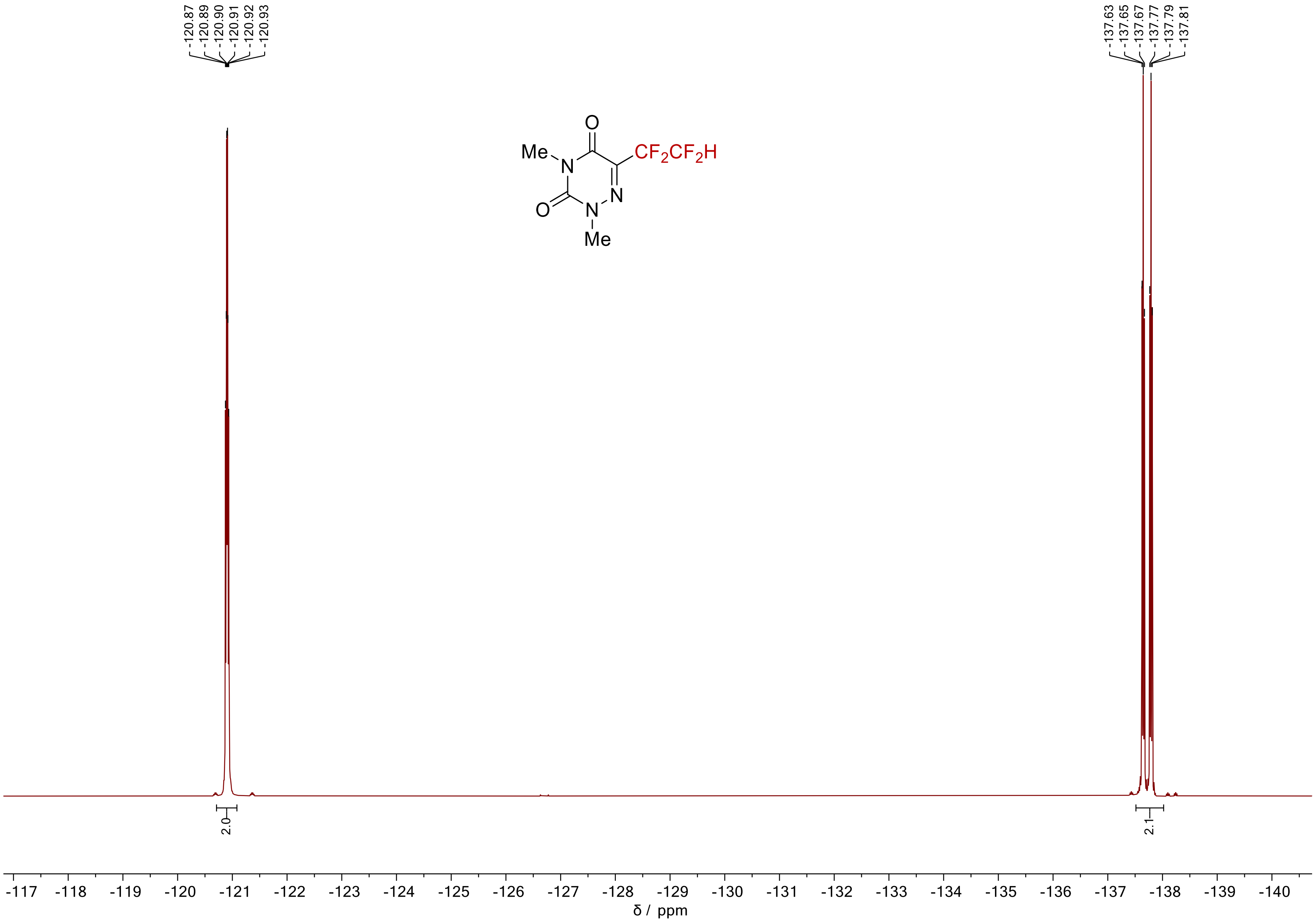

**6-(1,1-Difluoroethyl)-2,4-dimethyl-1,2,4-triazine-3,5(2*H*,4*H*)-dione 12**

$^{1}H$ NMR (400 MHz, $CDCl_3$)

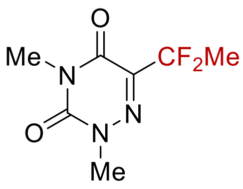


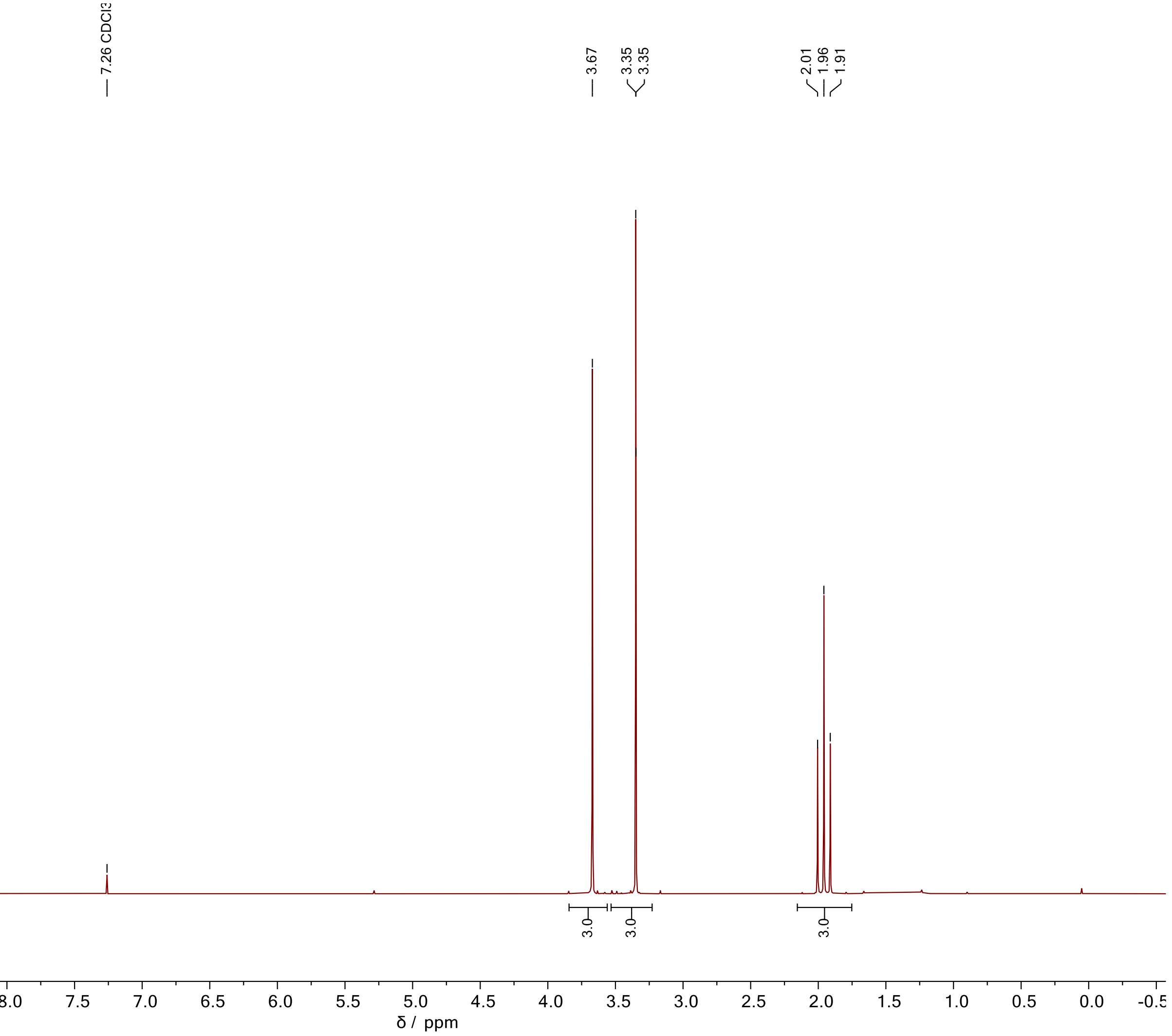

$^{13}C$ NMR (101 MHz, $CDCl_3$)

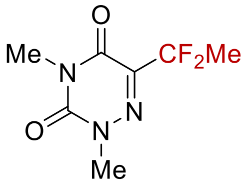

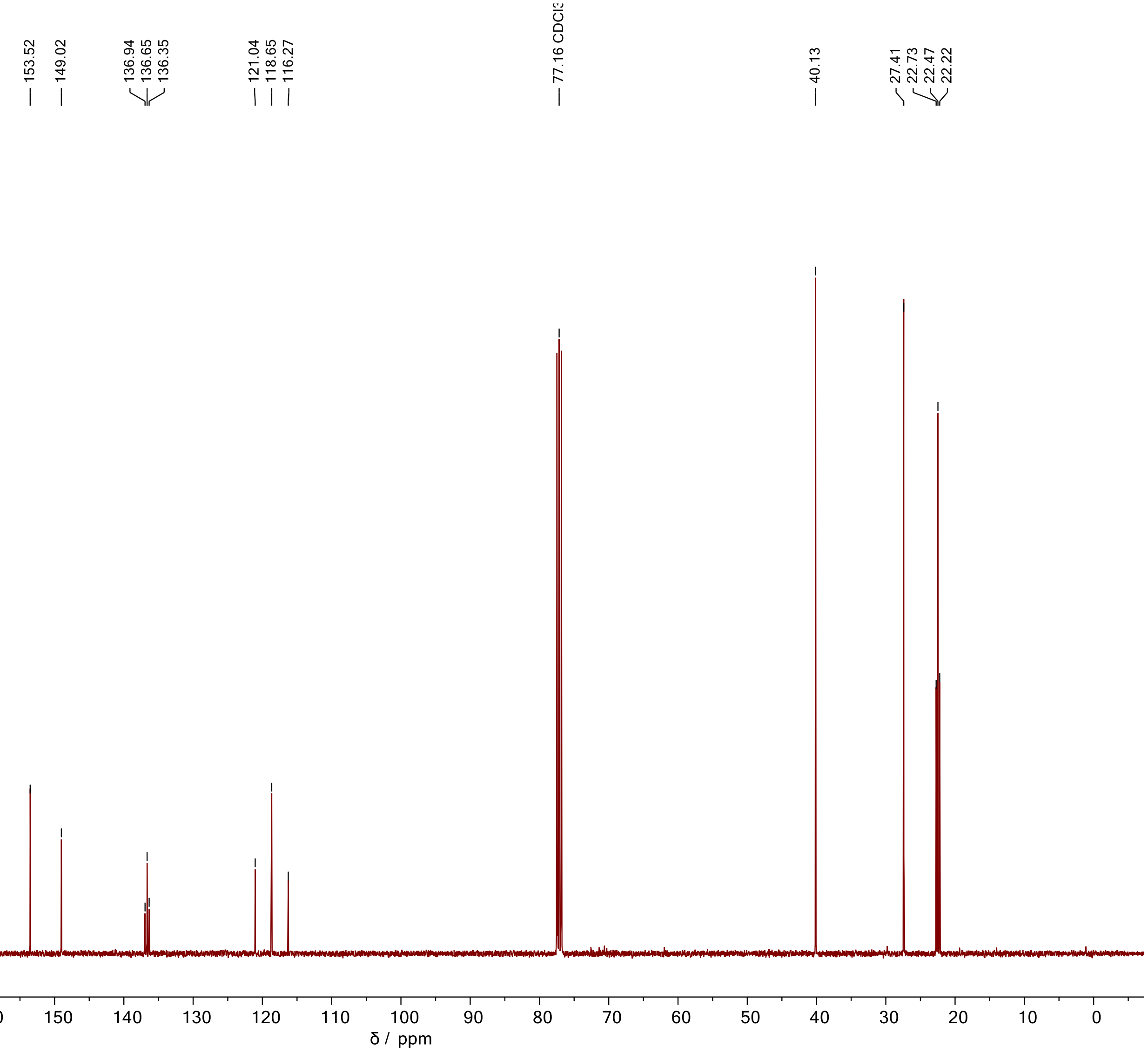

$^{19}F$ NMR (377 MHz, $CDCl_3$)

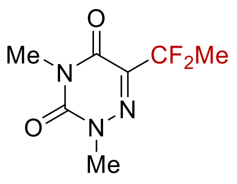


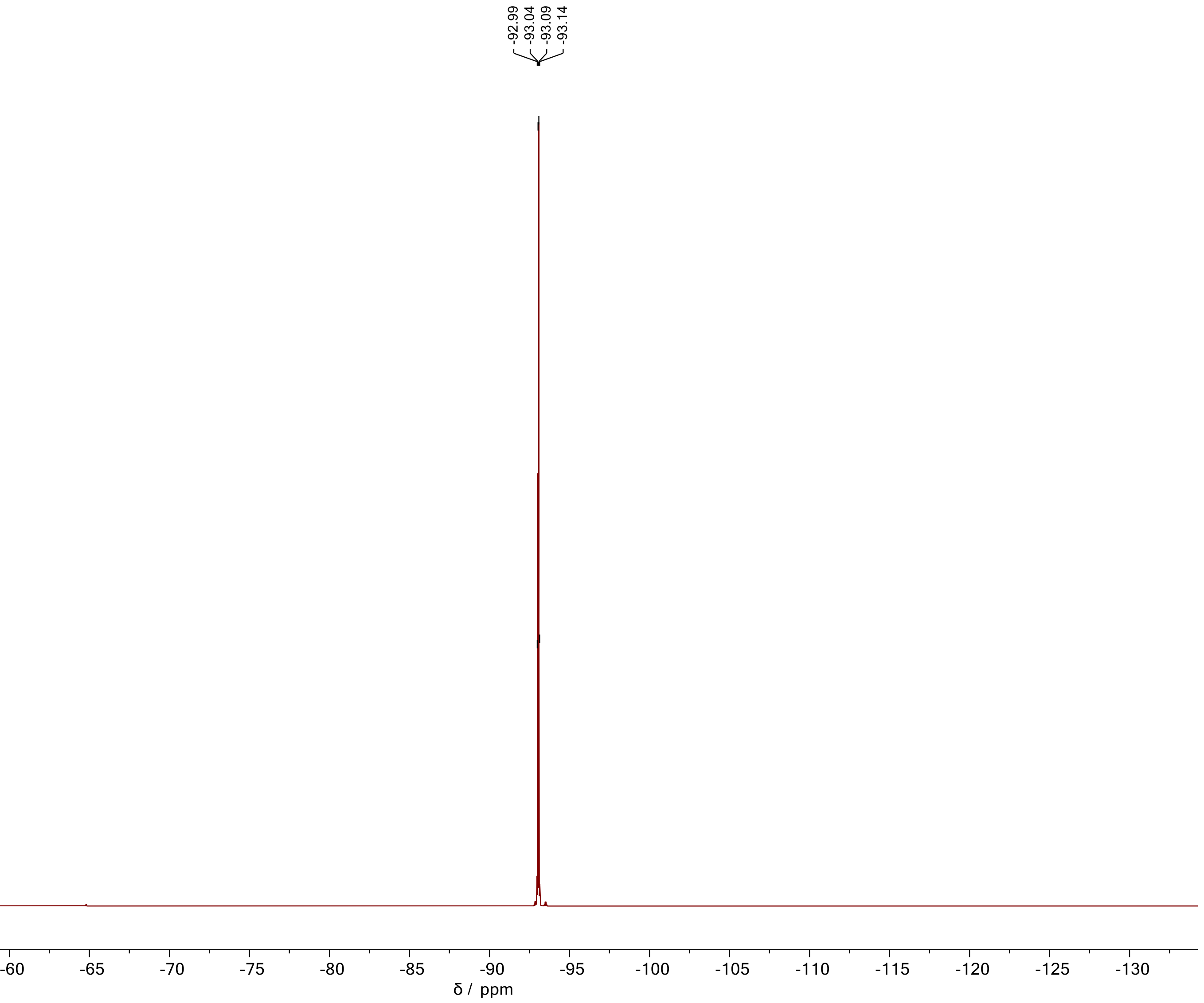

**6-(1,1-Difluoropropyl)-2,4-dimethyl-1,2,4-triazine-3,5(2*H*,4*H*)-dione 13**

$^{1}$H NMR (400 MHz, $CDCl_3$)

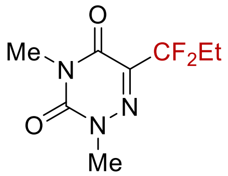

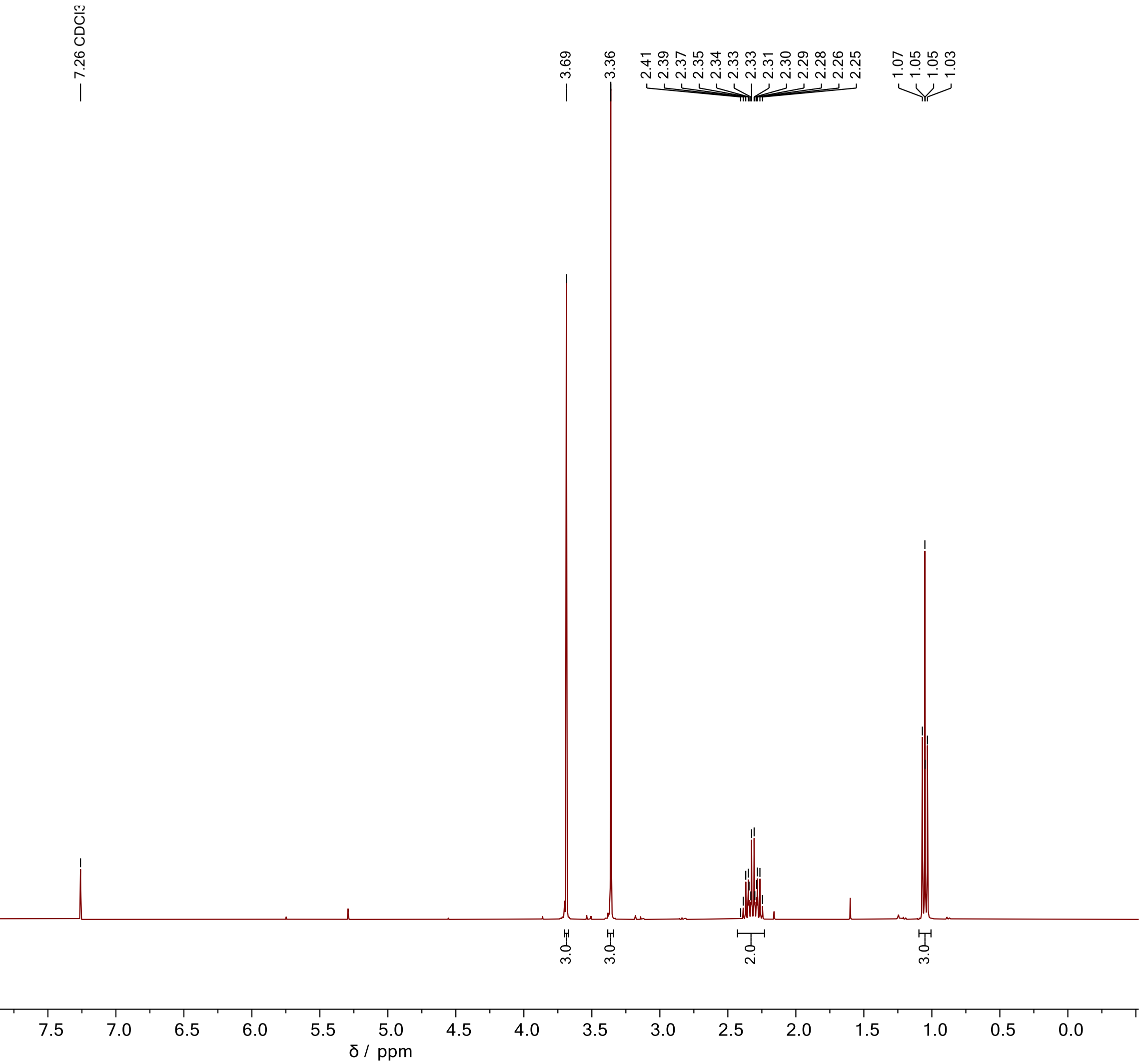

$^{13}C$ NMR (101 MHz, $CDCl_3$)

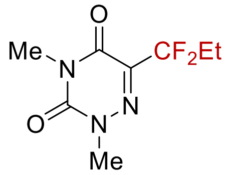


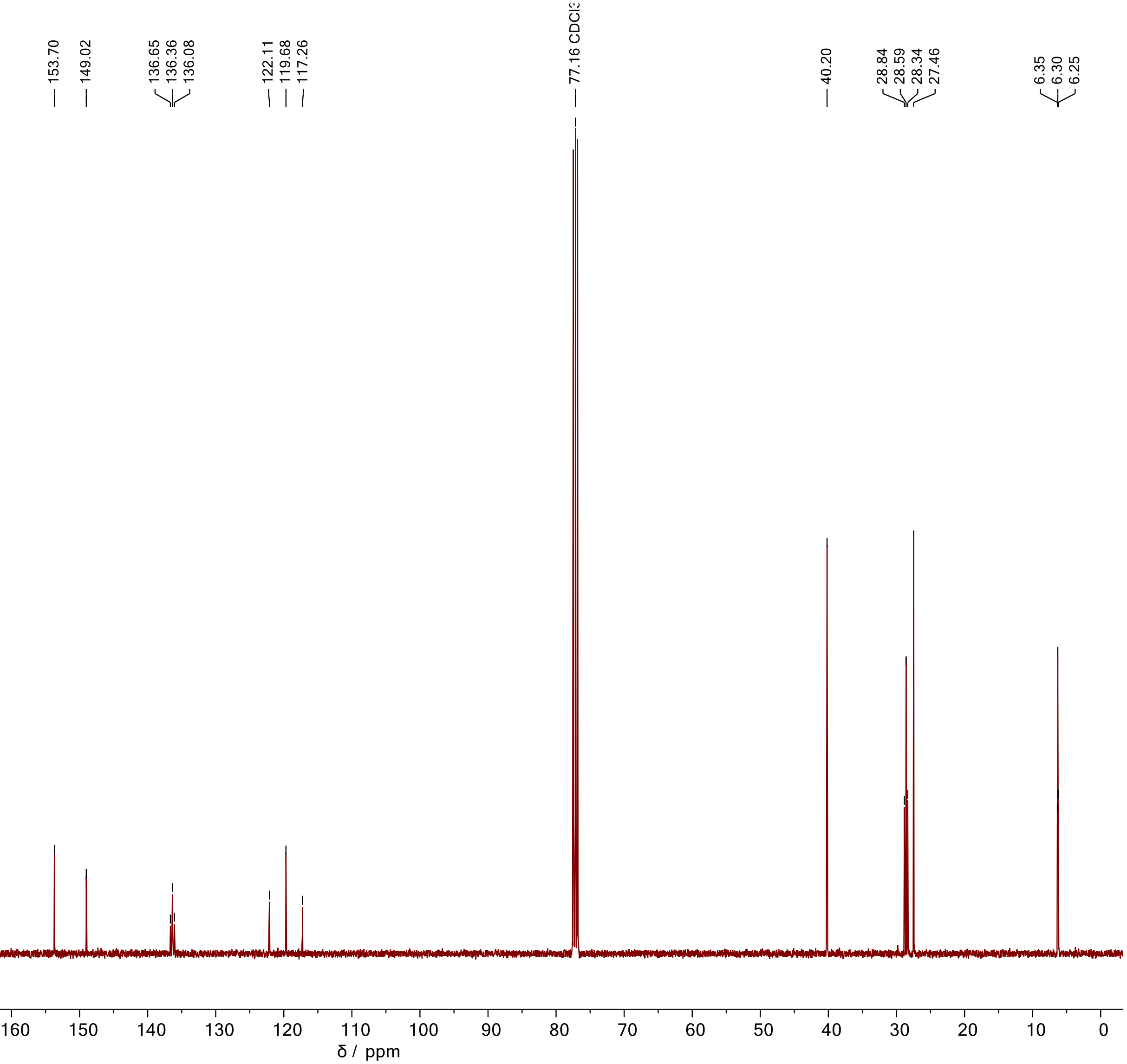

$^{19}$F NMR (377 MHz, $CDCl_3$)

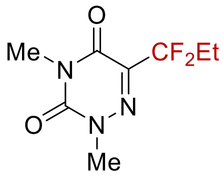

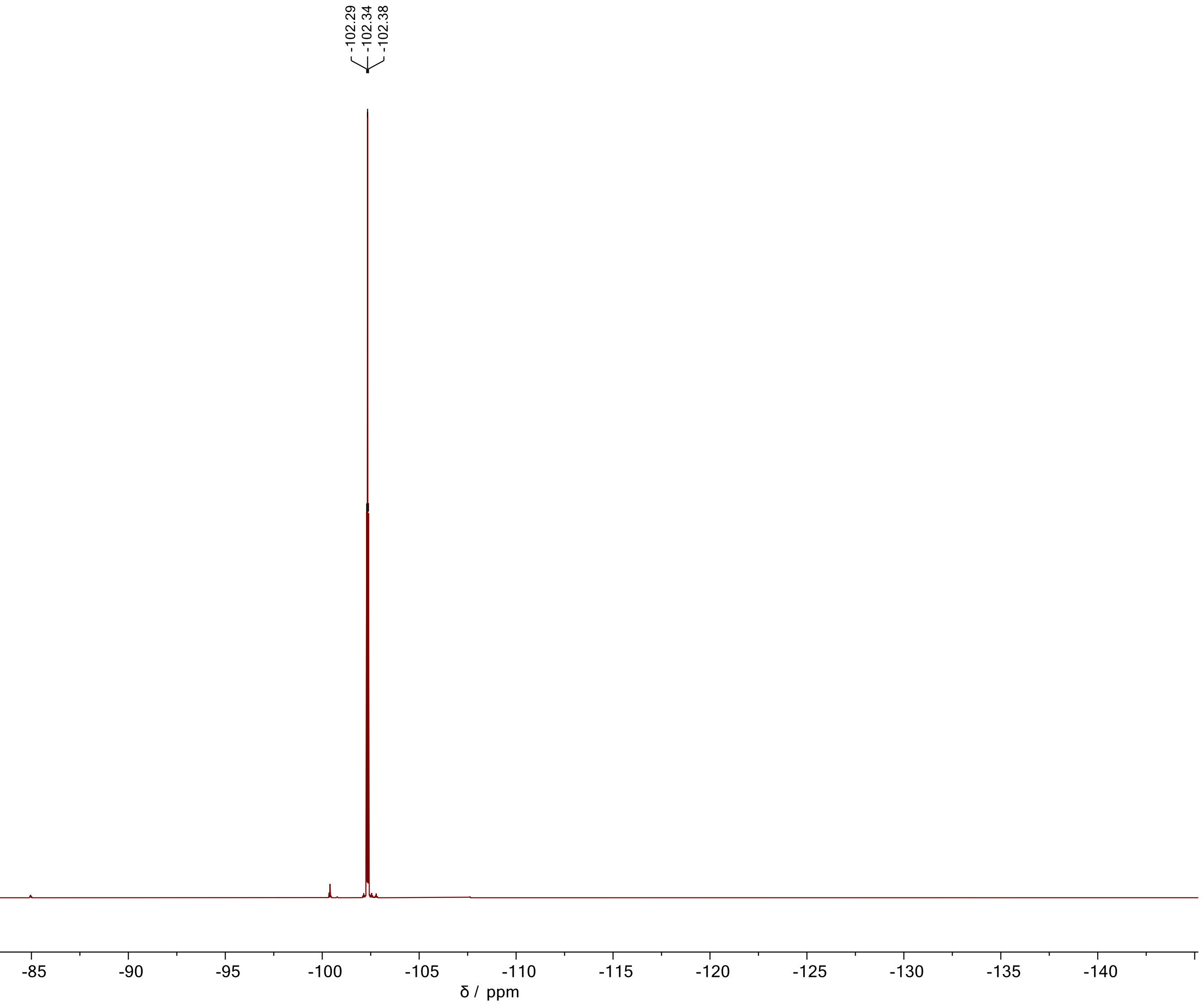

**6-(Difluoro(phenyl)methyl)-2,4-dimethyl-1,2,4-triazine-3,5(2$H$,4$H$)-dione 14**

$^{1}$H NMR (400 MHz, $CDCl_3$)

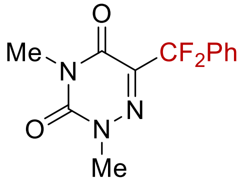

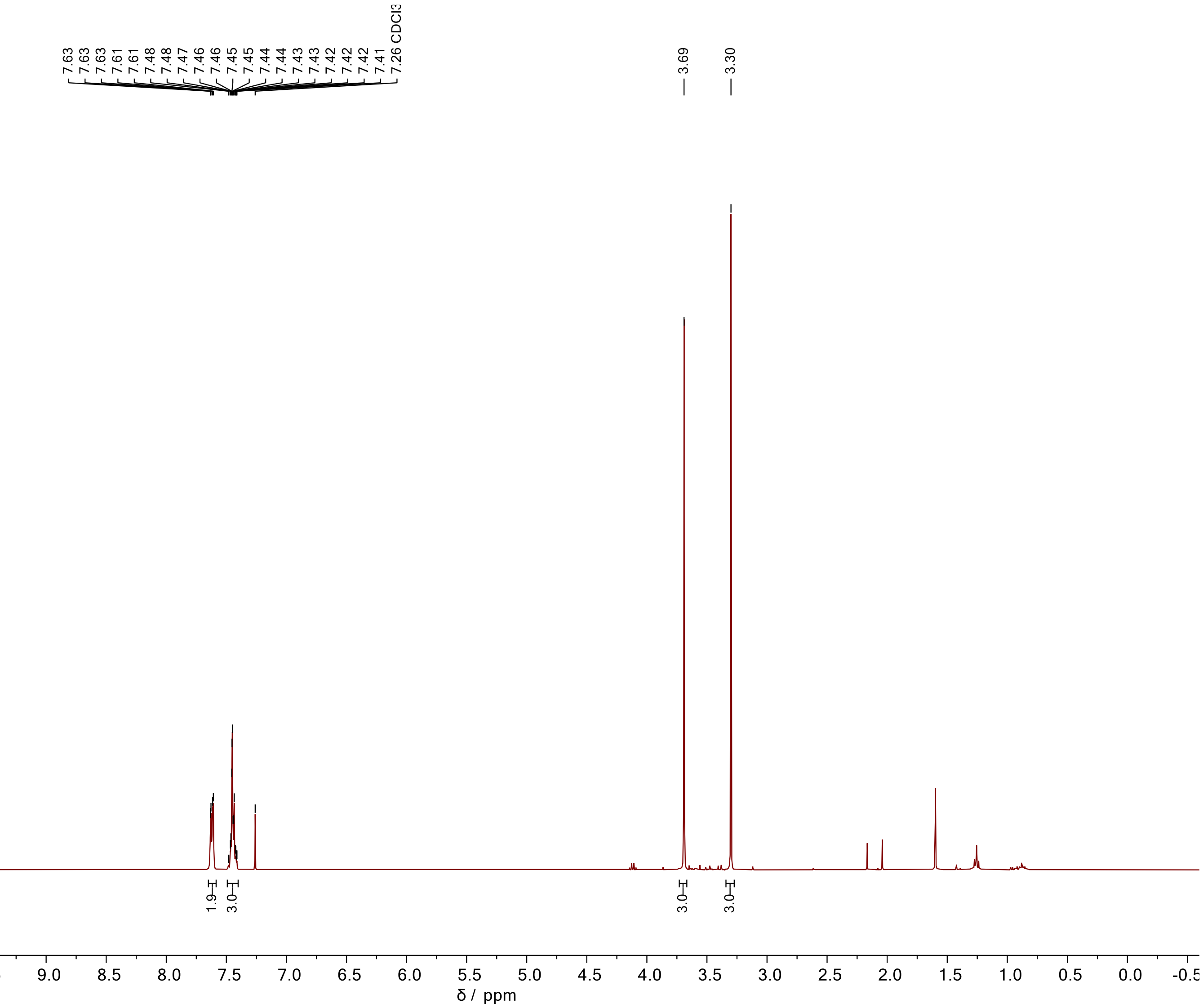

$^{13}C$ NMR (101 MHz, $CDCl_3$)

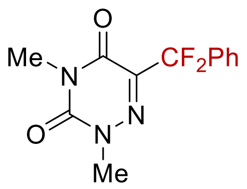


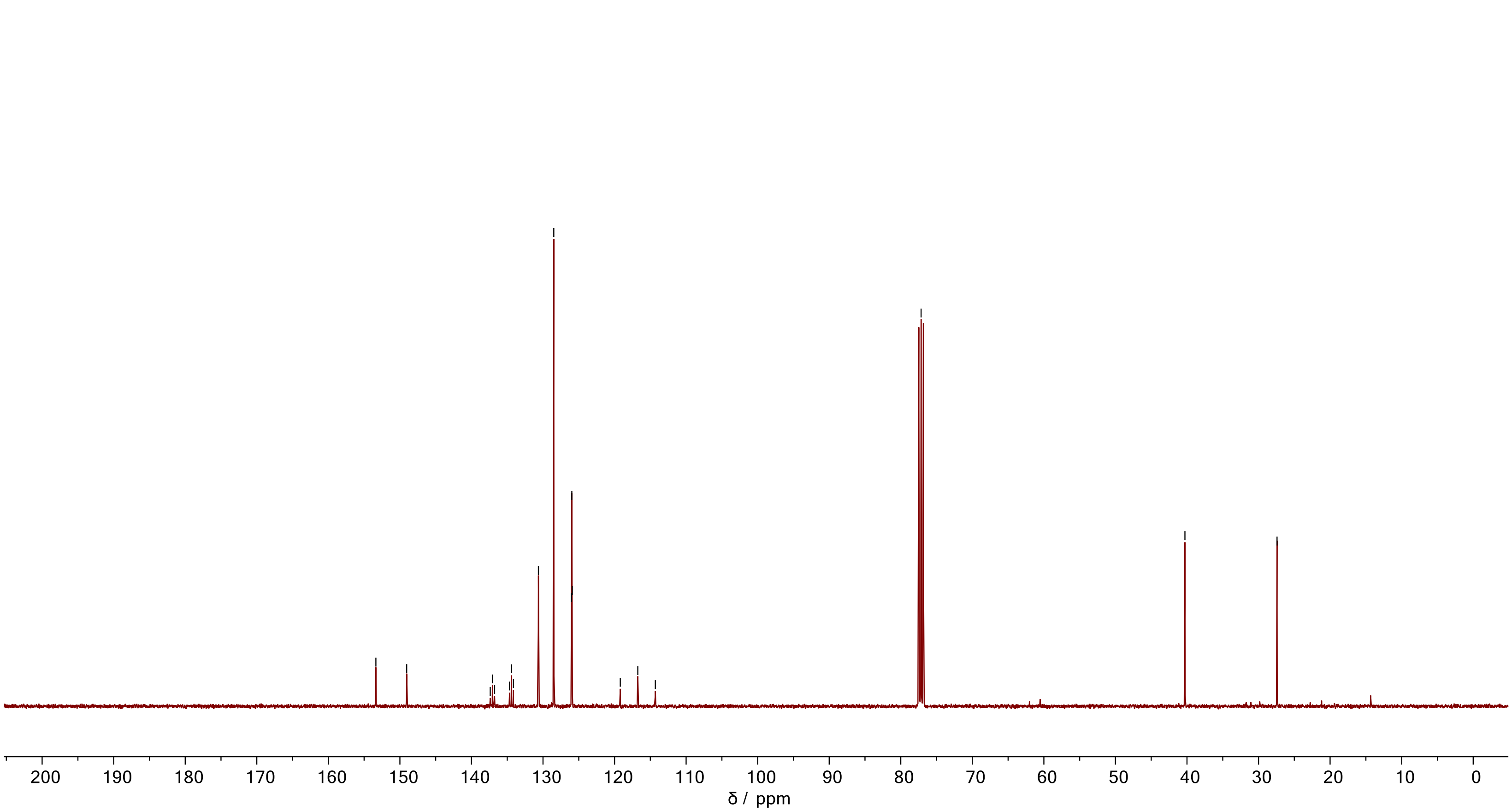

$^{19}$F NMR (377 MHz, $CDCl_3$)

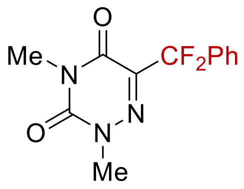


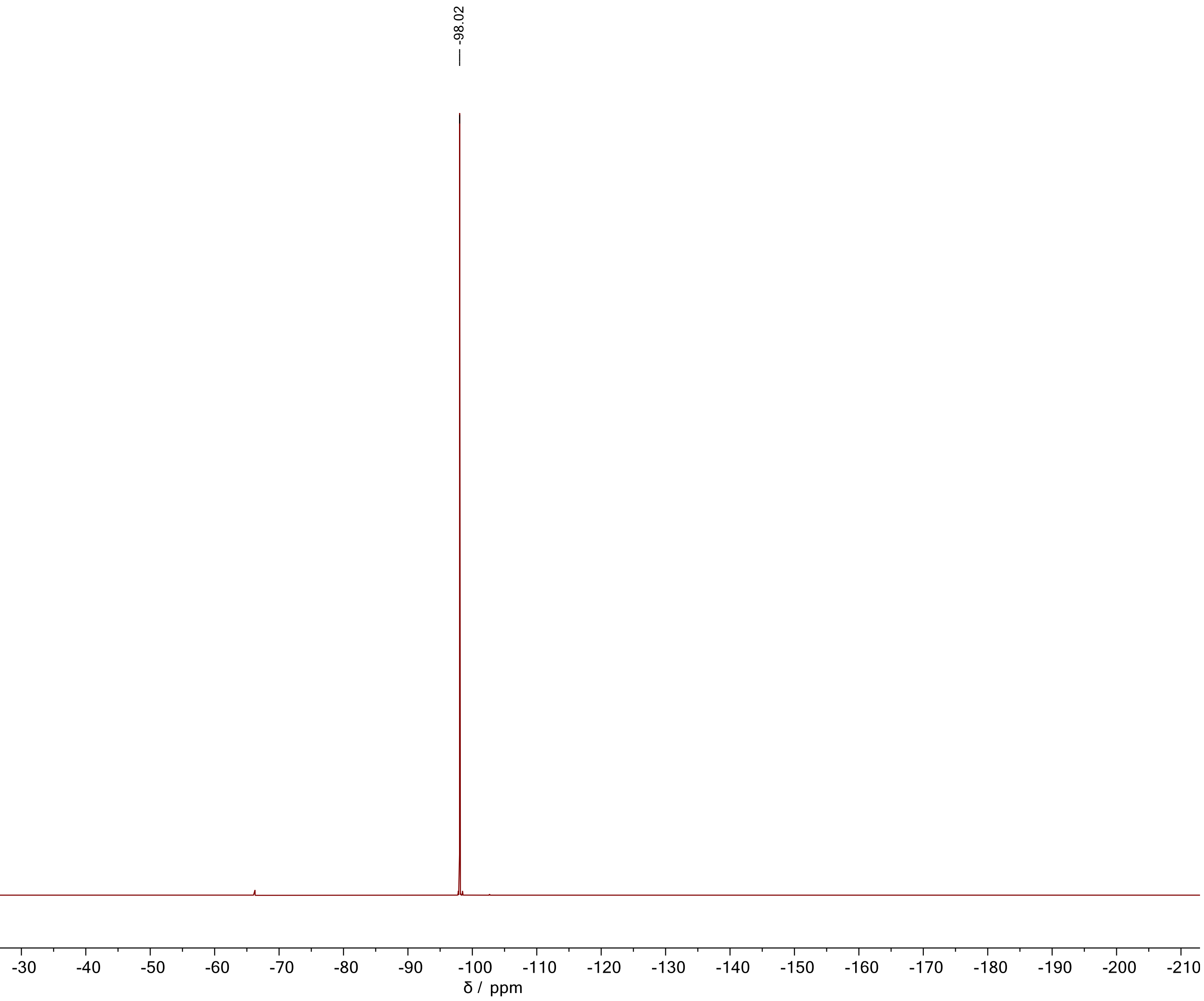

**2-(4-Chlorophenyl)-2-(2,6-dichloro-4-(6-(1,1-difluoroethyl)-4-methyl-3,5-dioxo-4,5-dihydro-1,2,4-triazin-2(3*H*)-yl)phenyl)acetonitrile 15**

$^{1}$H NMR (400 MHz, $CDCl_3$)

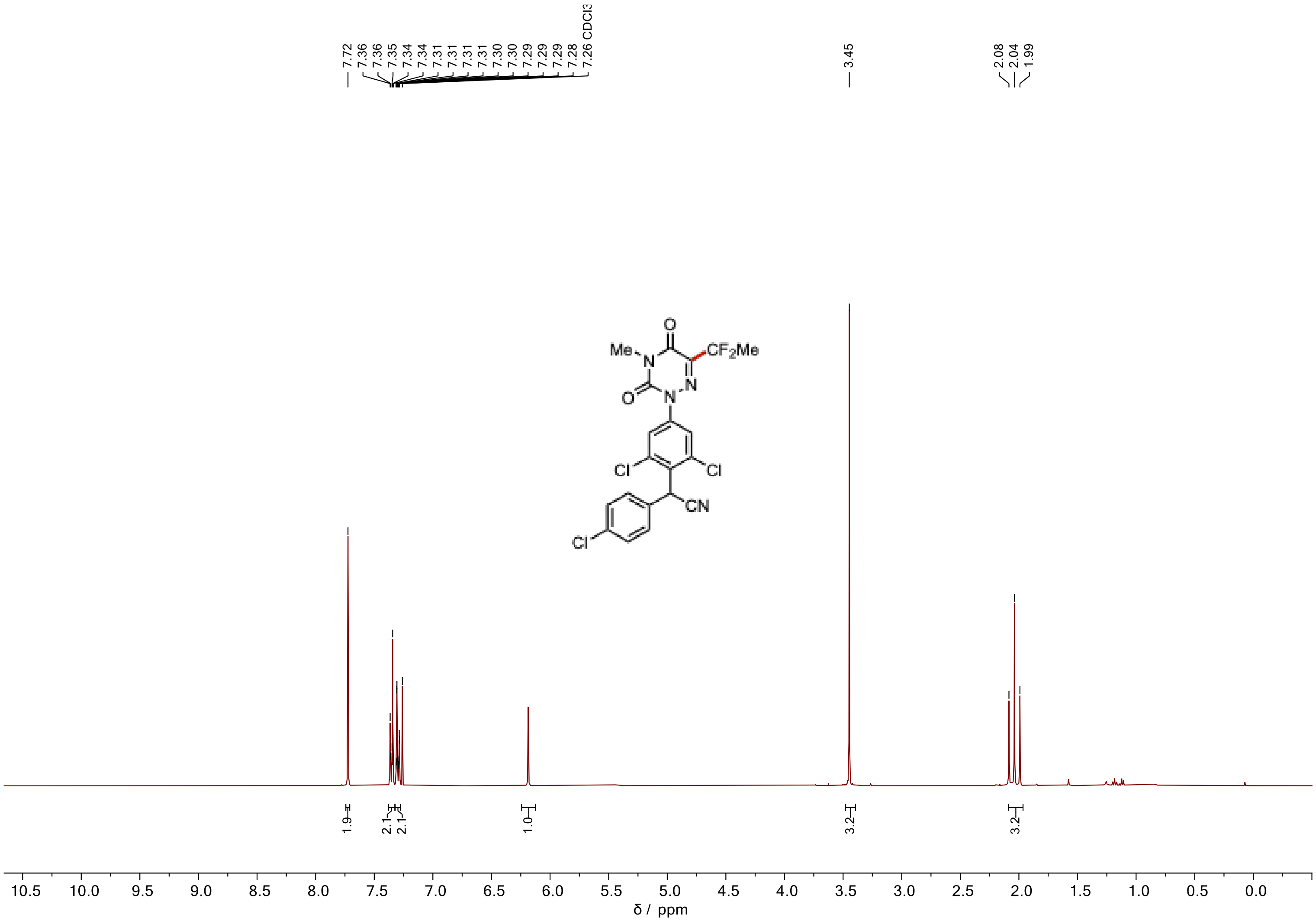

<sup></sup>

$^{13}$C NMR (101 MHz, $CDCl_3$)

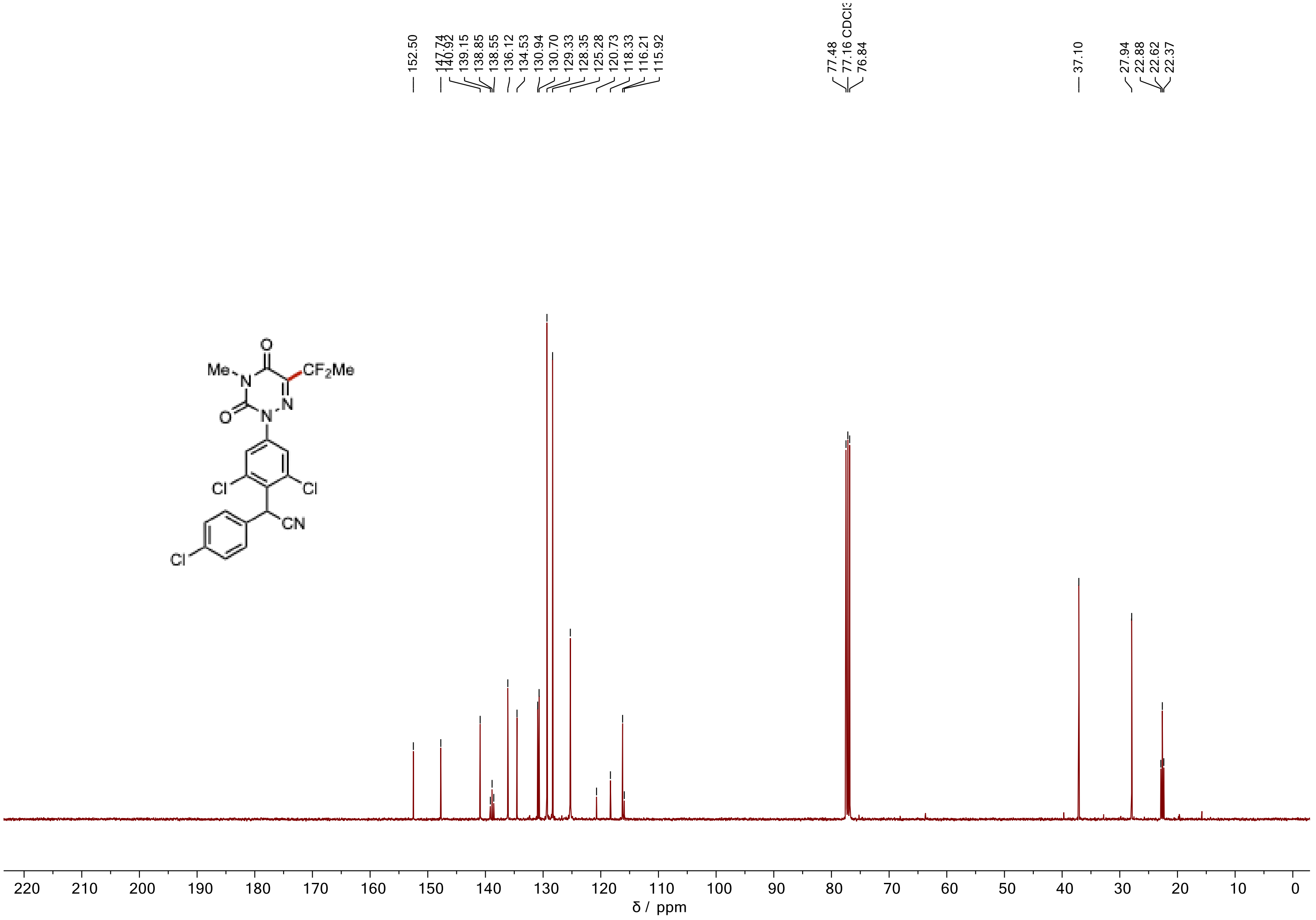

$^{19}F$ NMR (377 MHz, $CDCl_3$)

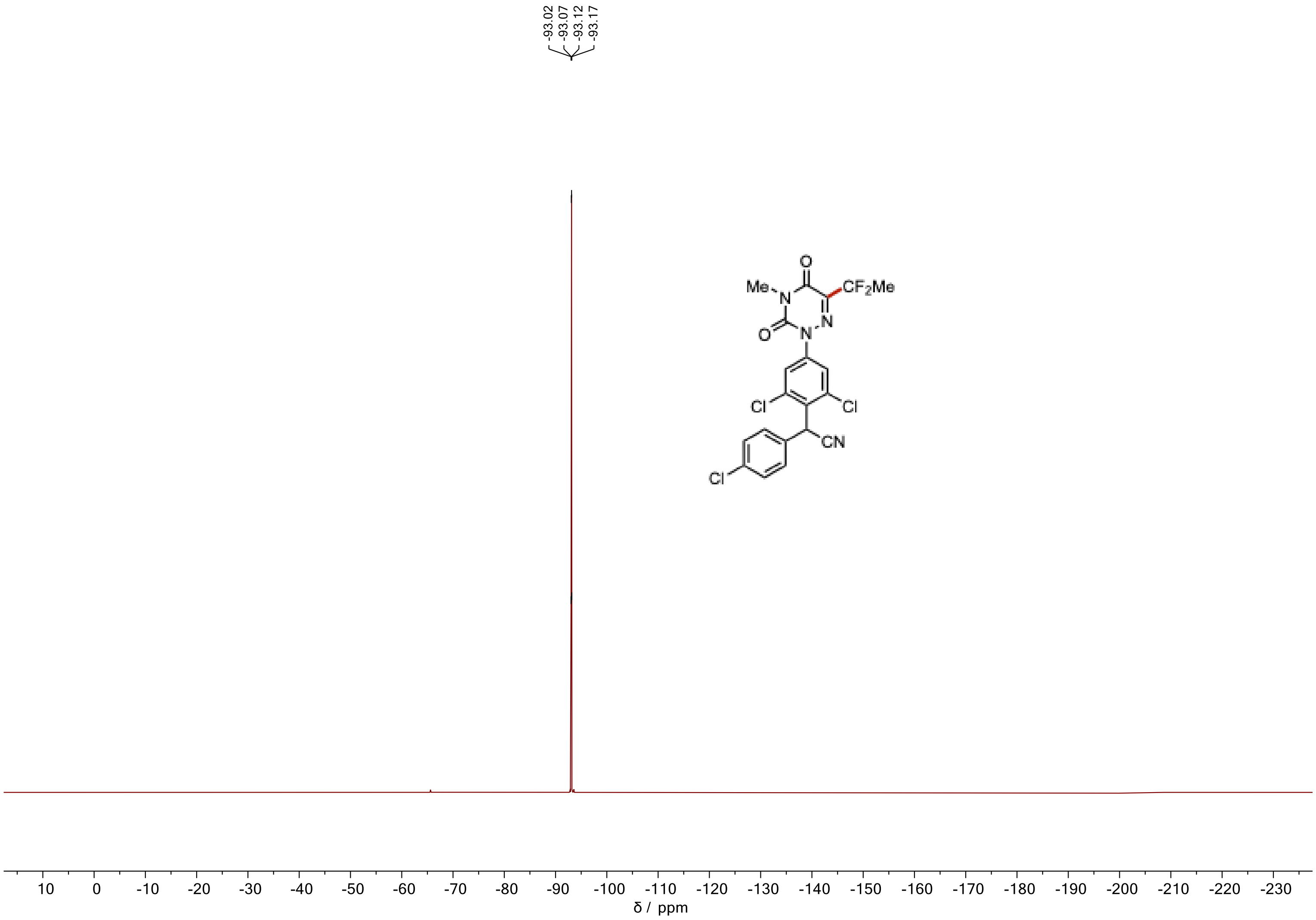

**1,3-Dimethyl-5-(1,1,2,2-tetrafluoroethyl)pyrimidine-2,4(1*H*,3*H*)-dione 16**

$^1$H NMR (300 MHz, $CDCl_3$)

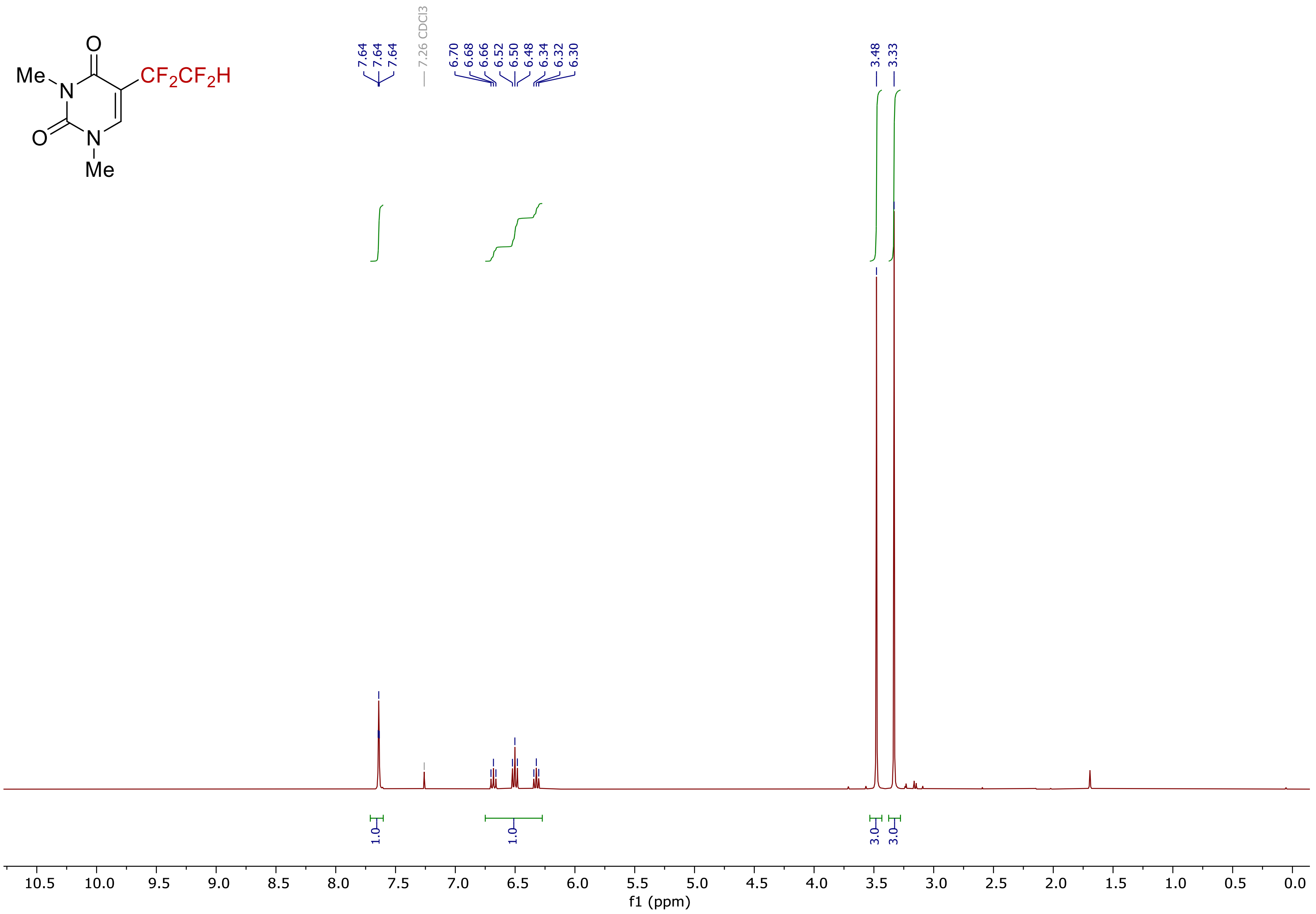

$^{13}C$ NMR (75 MHz, $CDCl_3$)

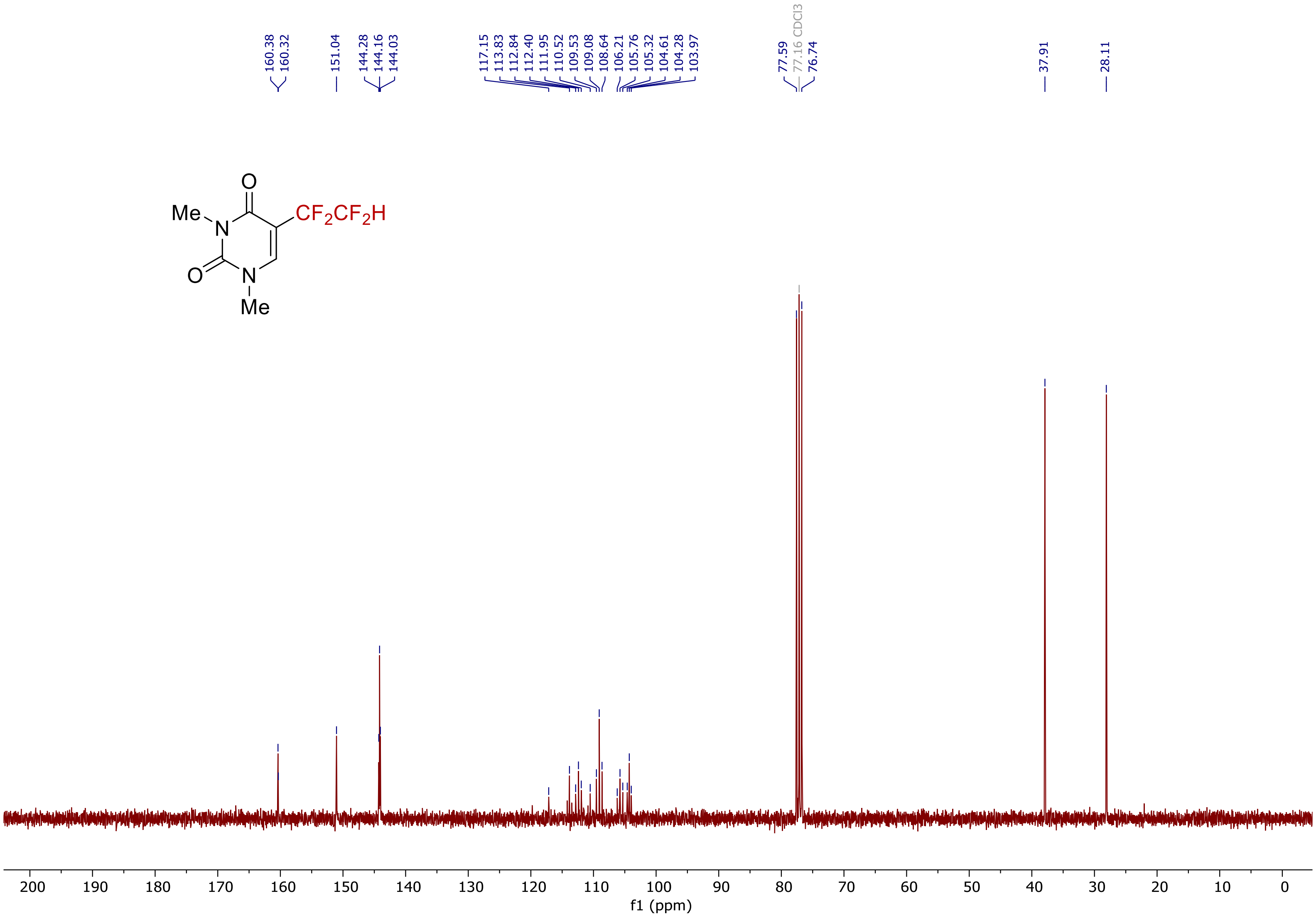

$^{19}F$ NMR (282 MHz, $CDCl_3$)

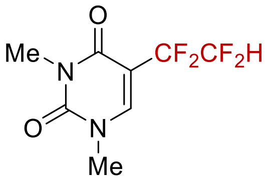

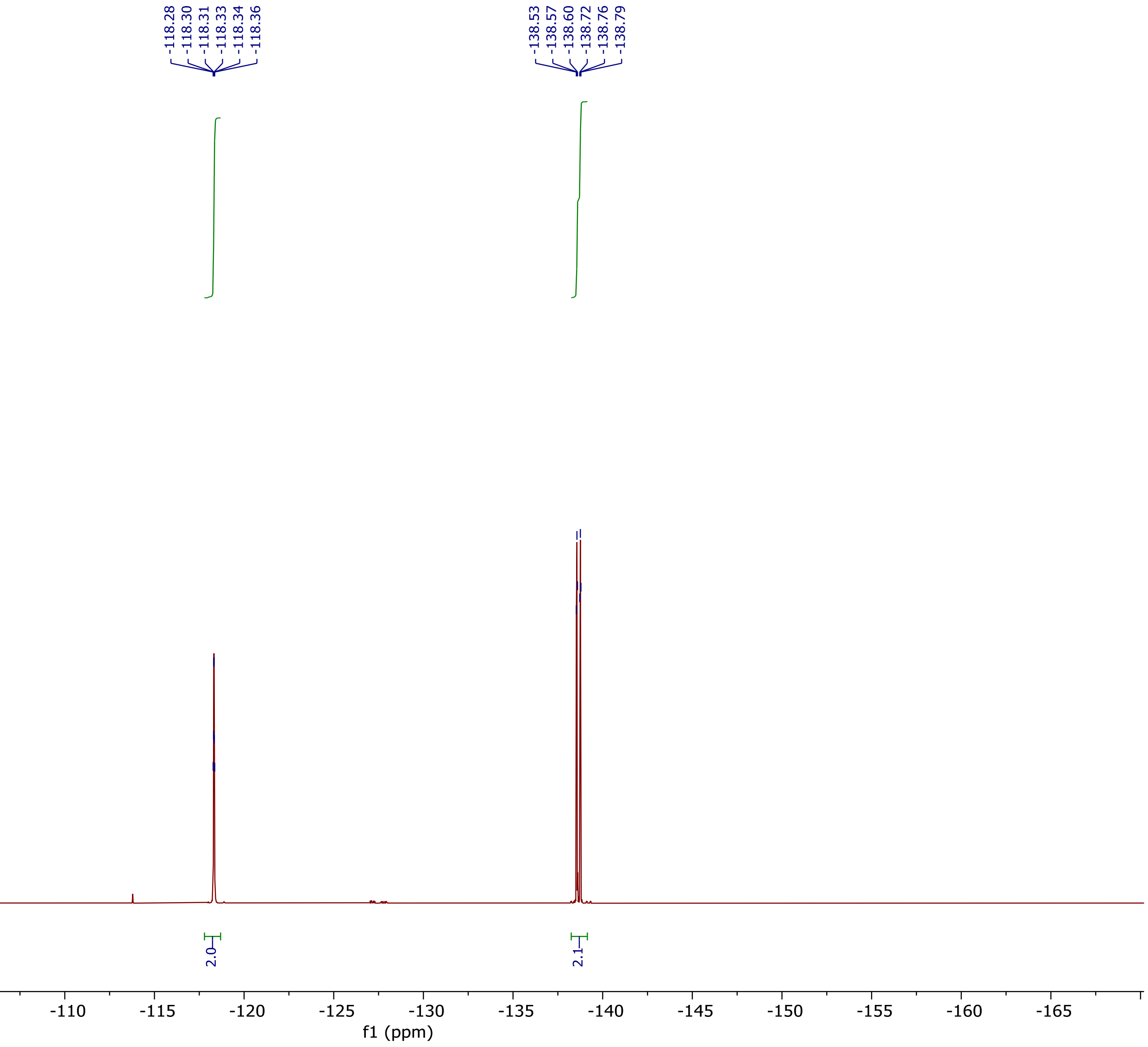

**2,6-Dimethoxy-3-(1,1,2,2-tetrafluoroethyl)pyridine 17**

$^1$H NMR (300 MHz, $CDCl_3$)

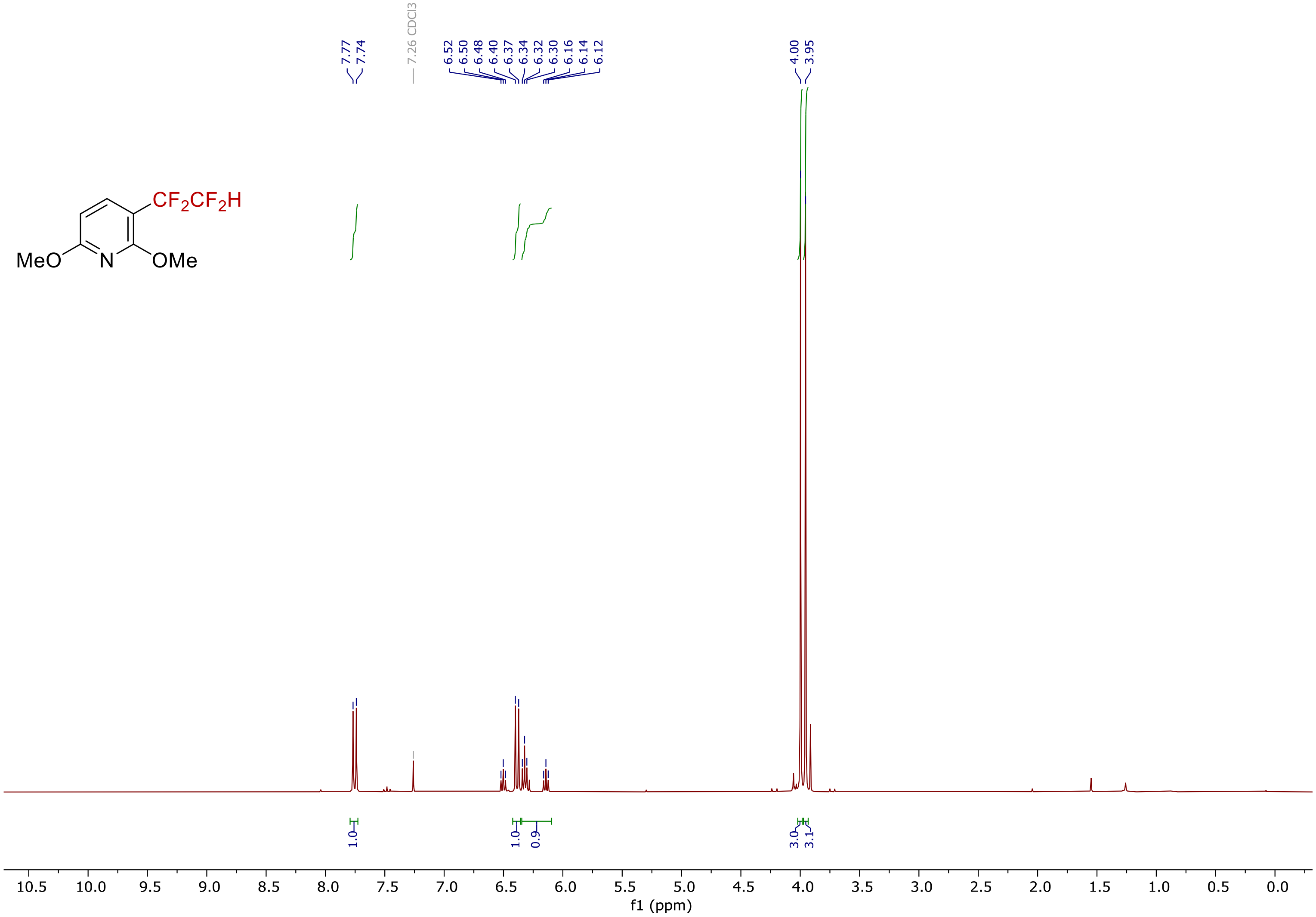

$^{13}C$ NMR (75 MHz, $CDCl_3$)

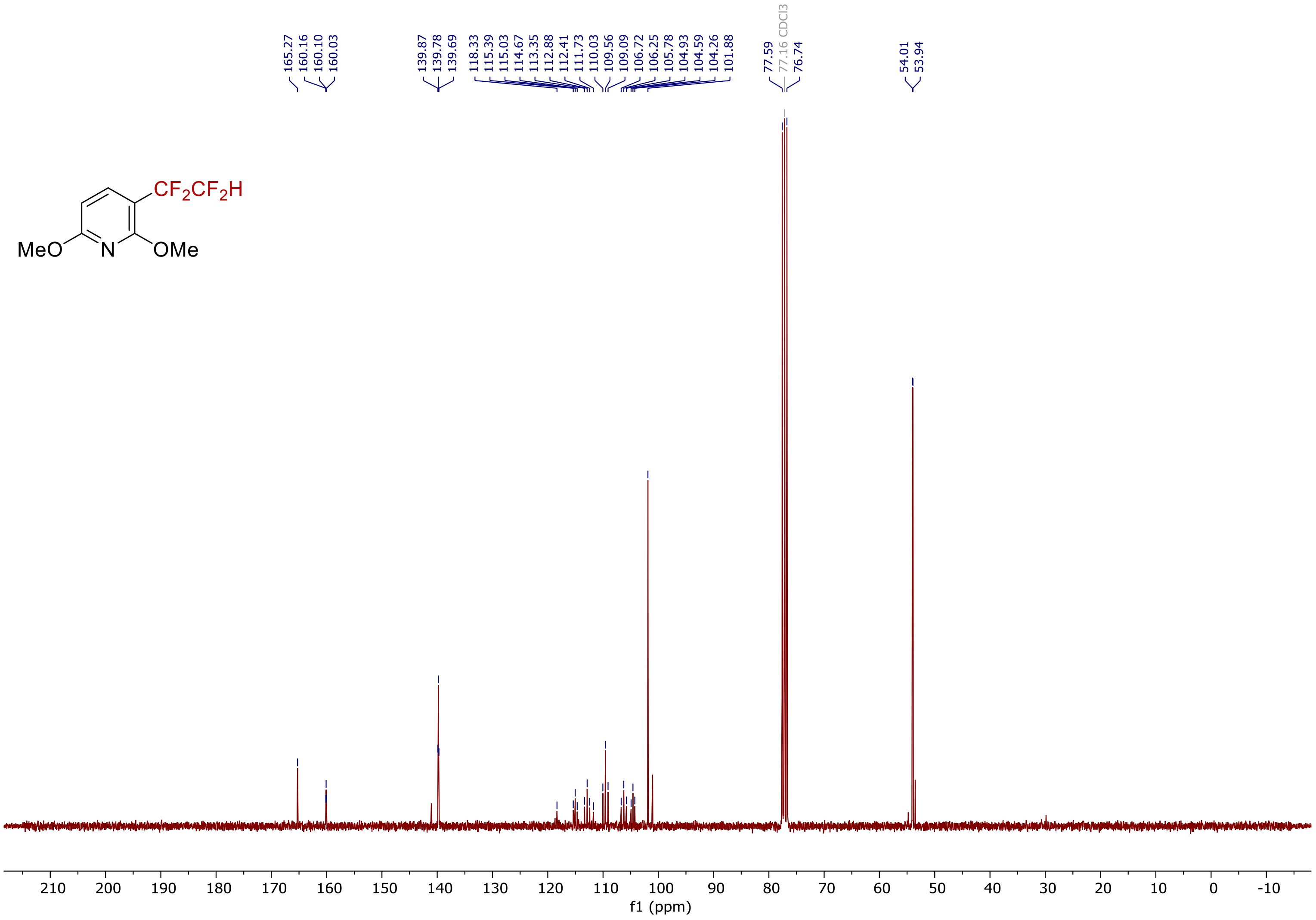

$^{19}F$ NMR (282 MHz, $CDCl_3$)

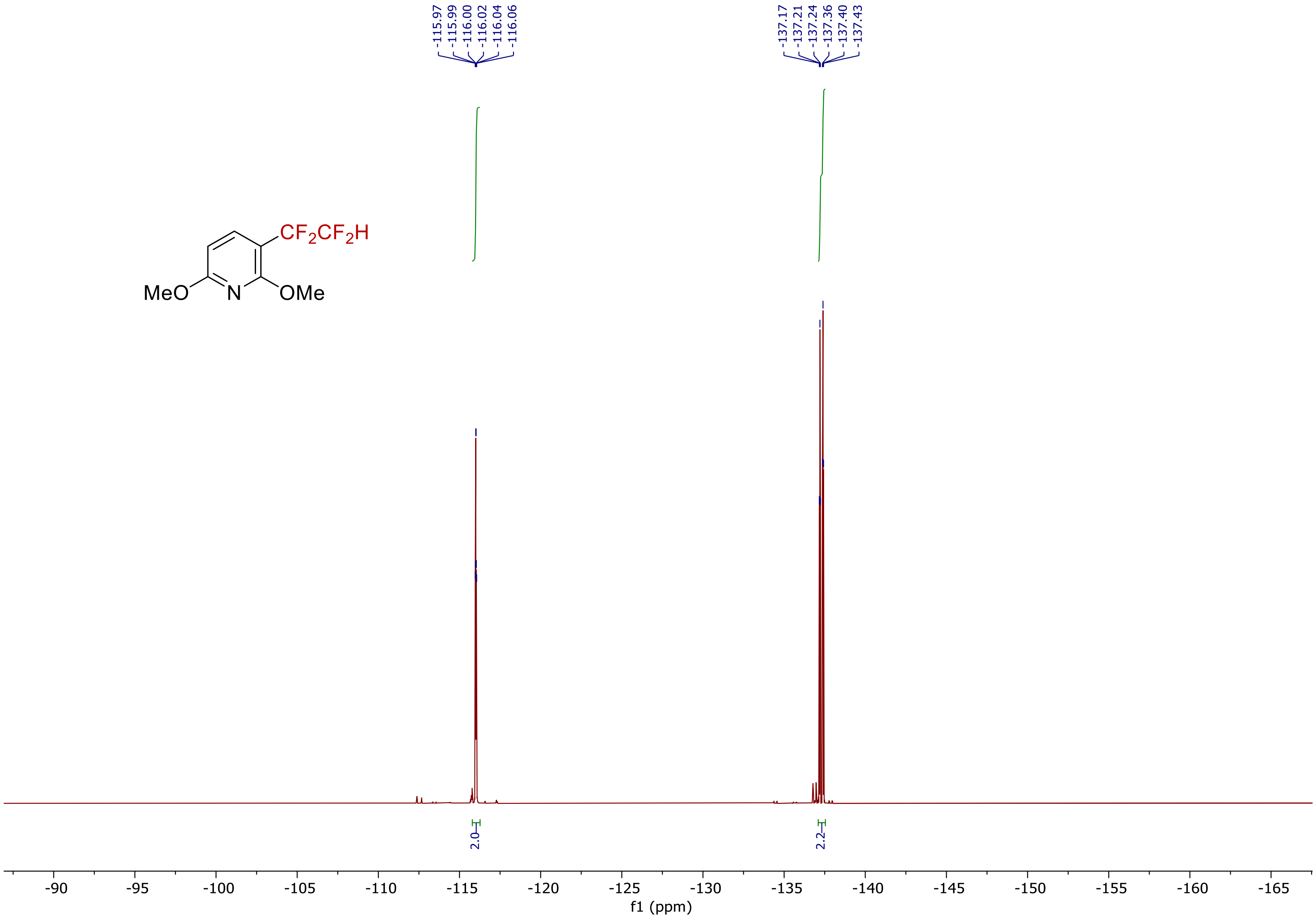

**2-(1,1-Difluoroethyl)quinoxaline 18**

$^1$H NMR (300 MHz, $CDCl_3$)

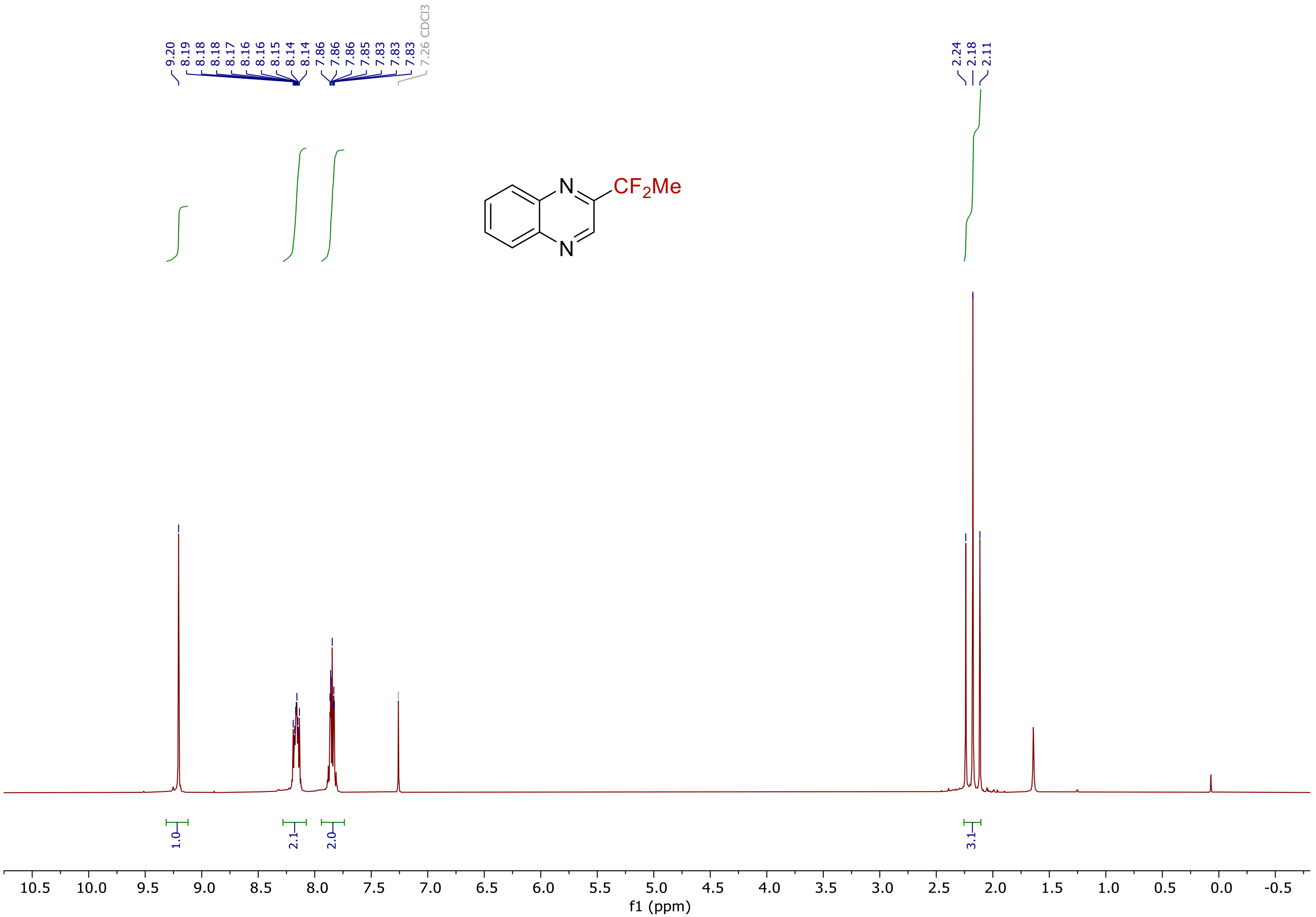

$^{13}C$ NMR (75 MHz, $CDCl_3$)

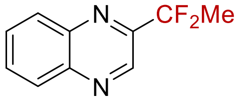


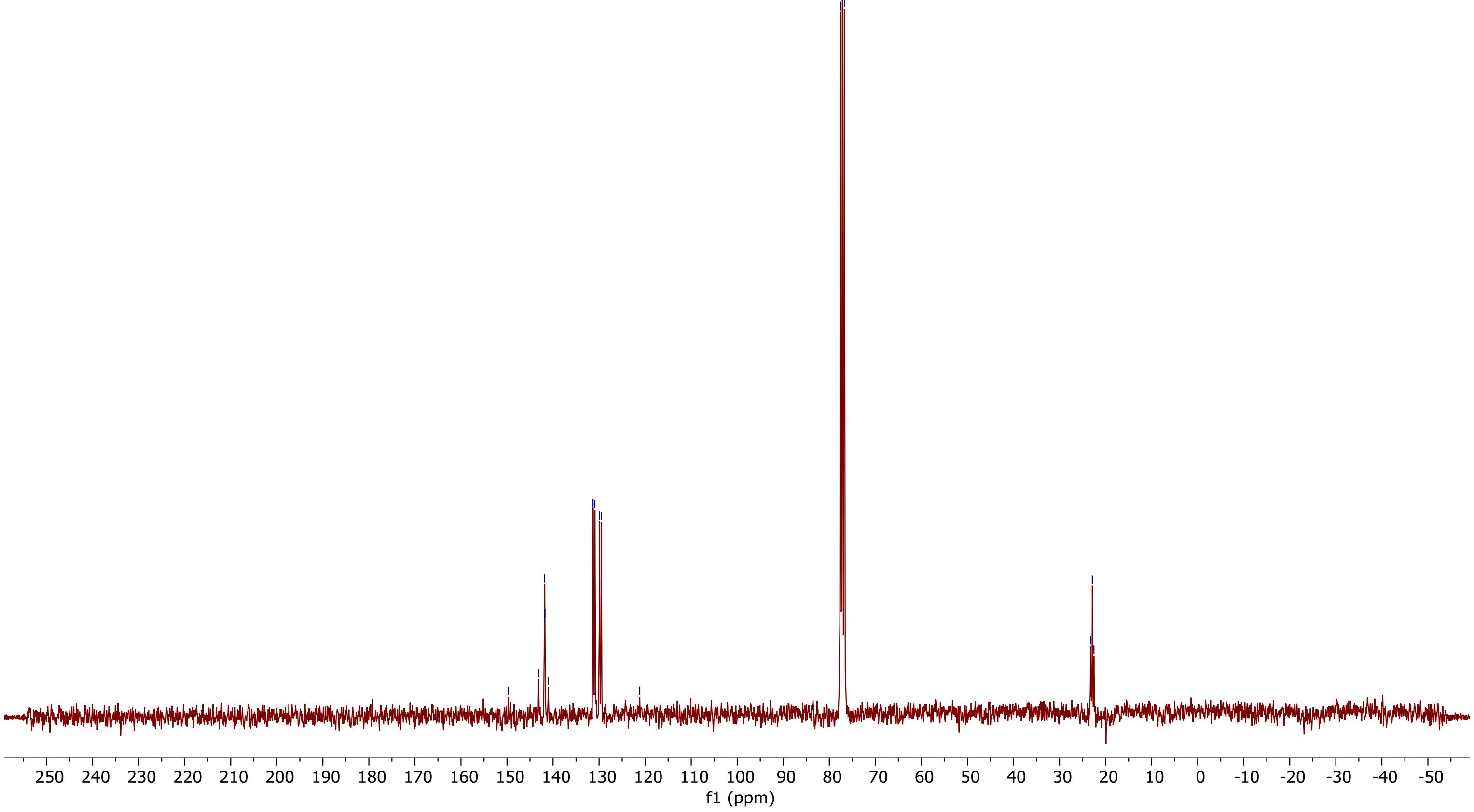

$^{19}F$ NMR (282 MHz, $CDCl_3$)

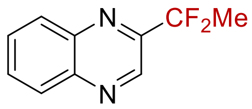


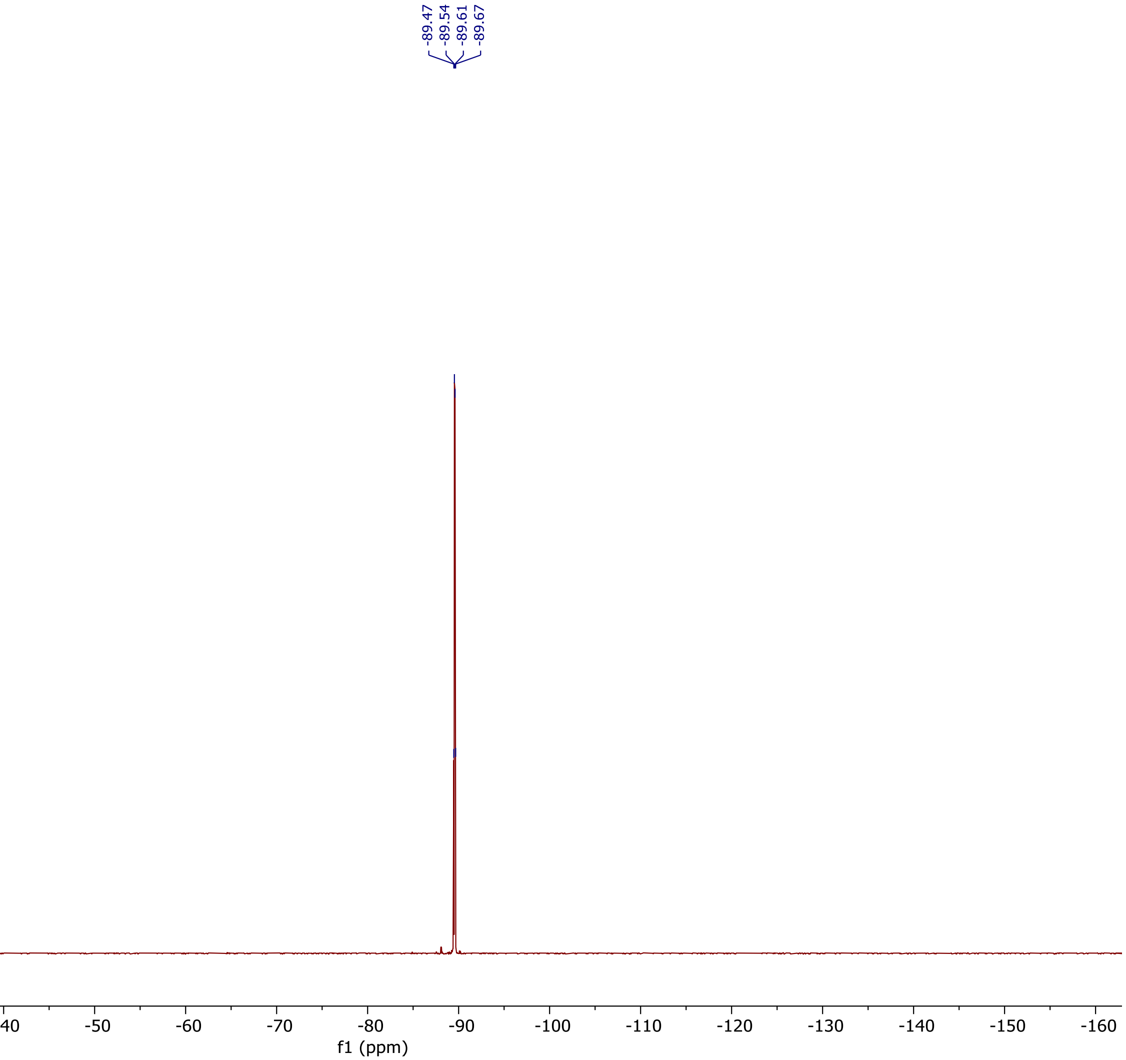

**3-(1,1-Difluoroethyl)-2*H*-chromen-2-one 19**

$^1$H NMR (300 MHz, $CDCl_3$)

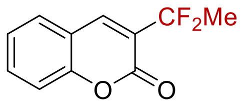

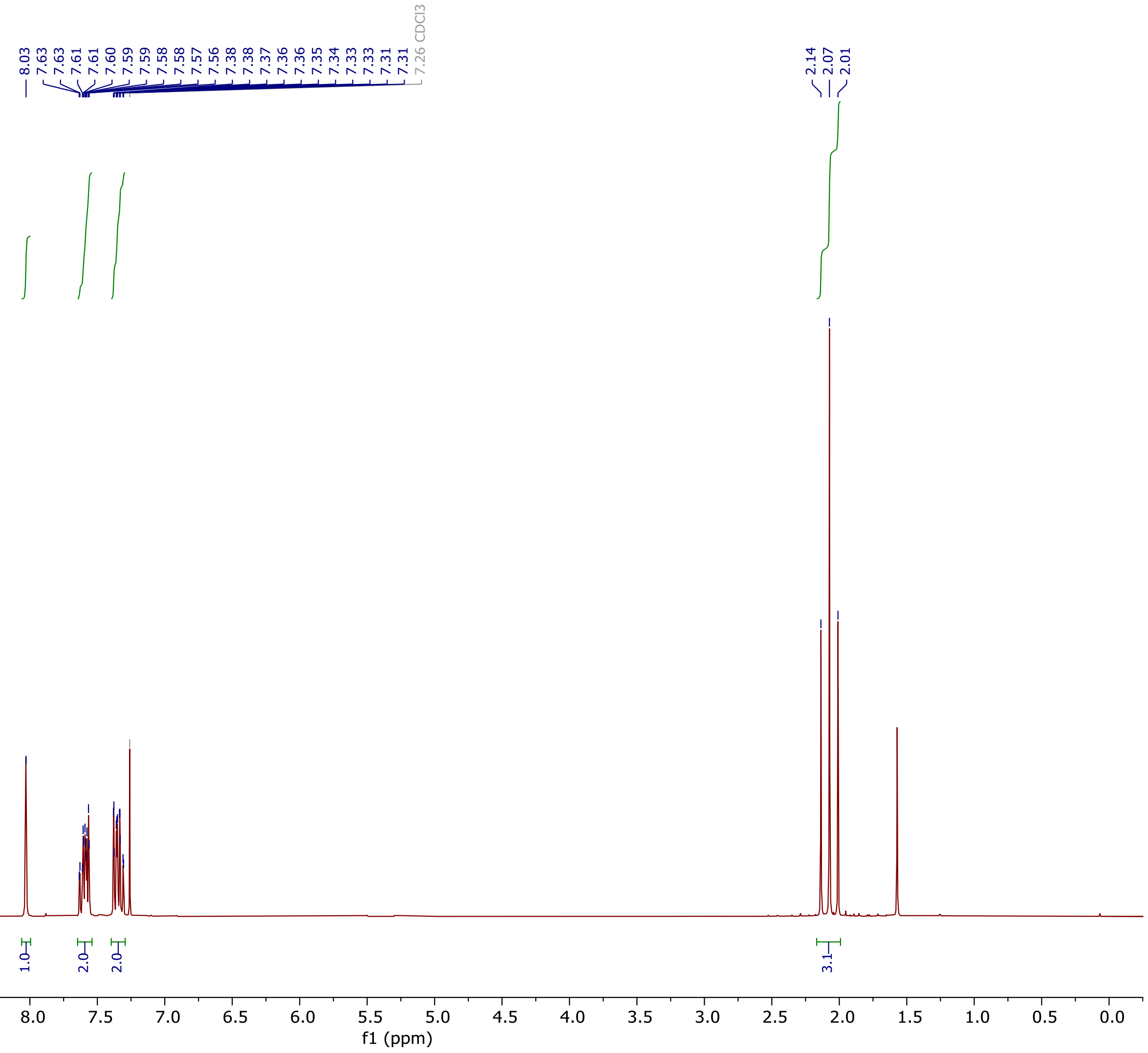

$^{13}C$ NMR (75 MHz, $CDCl_3$)

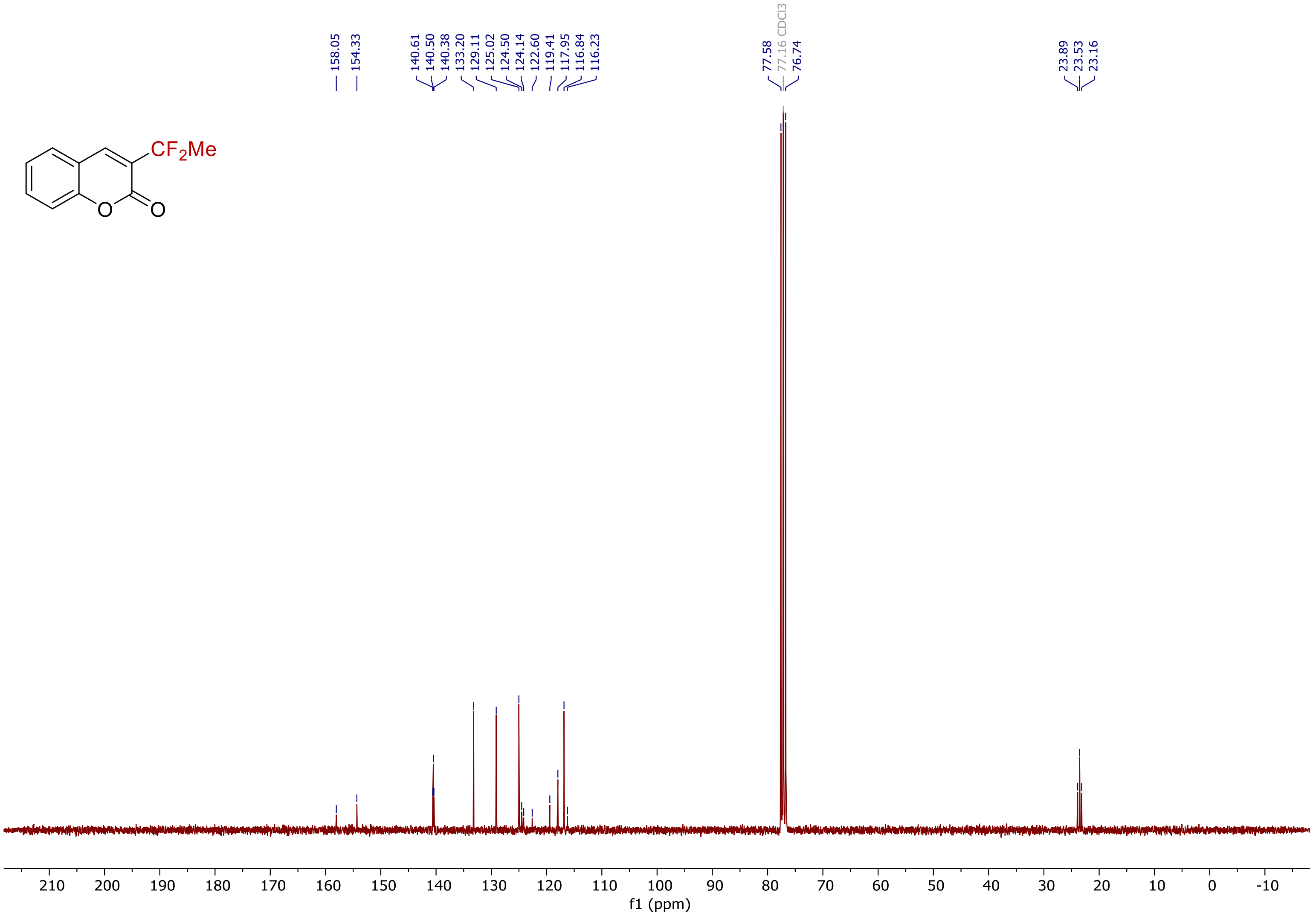

$^{19}F$ NMR (282 MHz, $CDCl_3$)

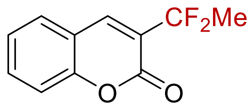


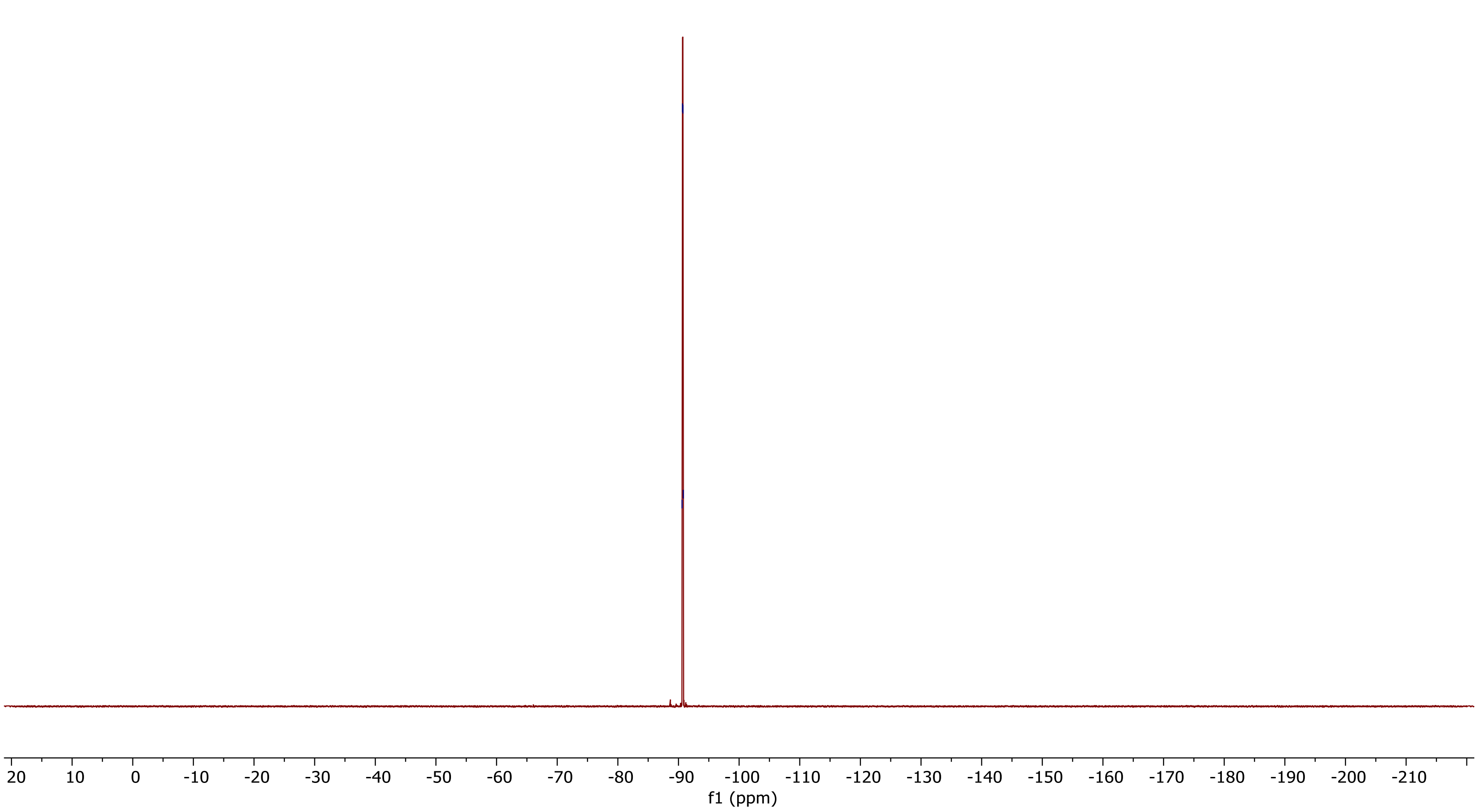

**5-Chloro-2-(1,1,2,2-tetrafluoroethyl)benzo[d]thiazole 20**

$^{1}$H NMR (300 MHz, $CDCl_3$)

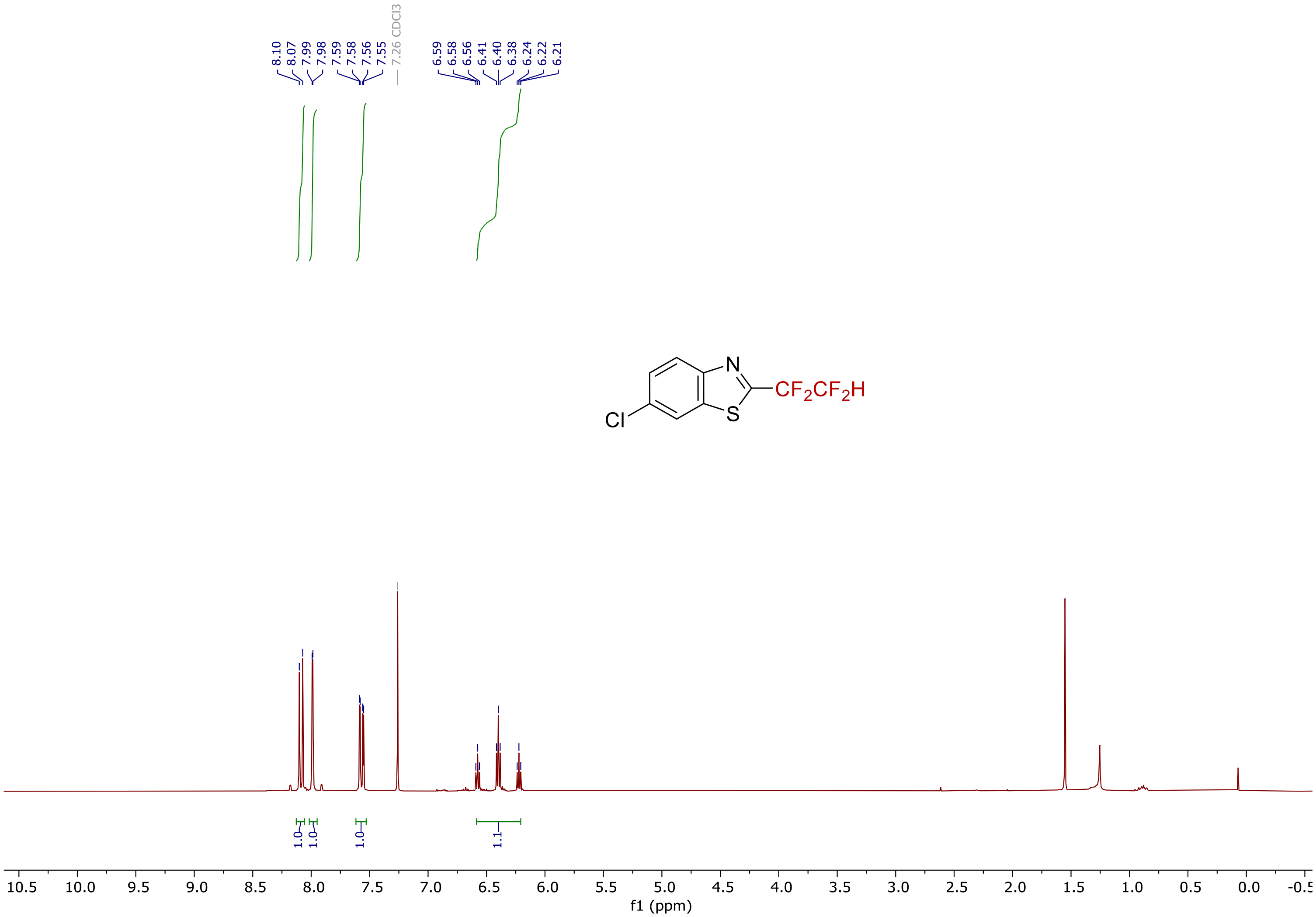

$^{13}C$ NMR (75 MHz, $CDCl_3$)

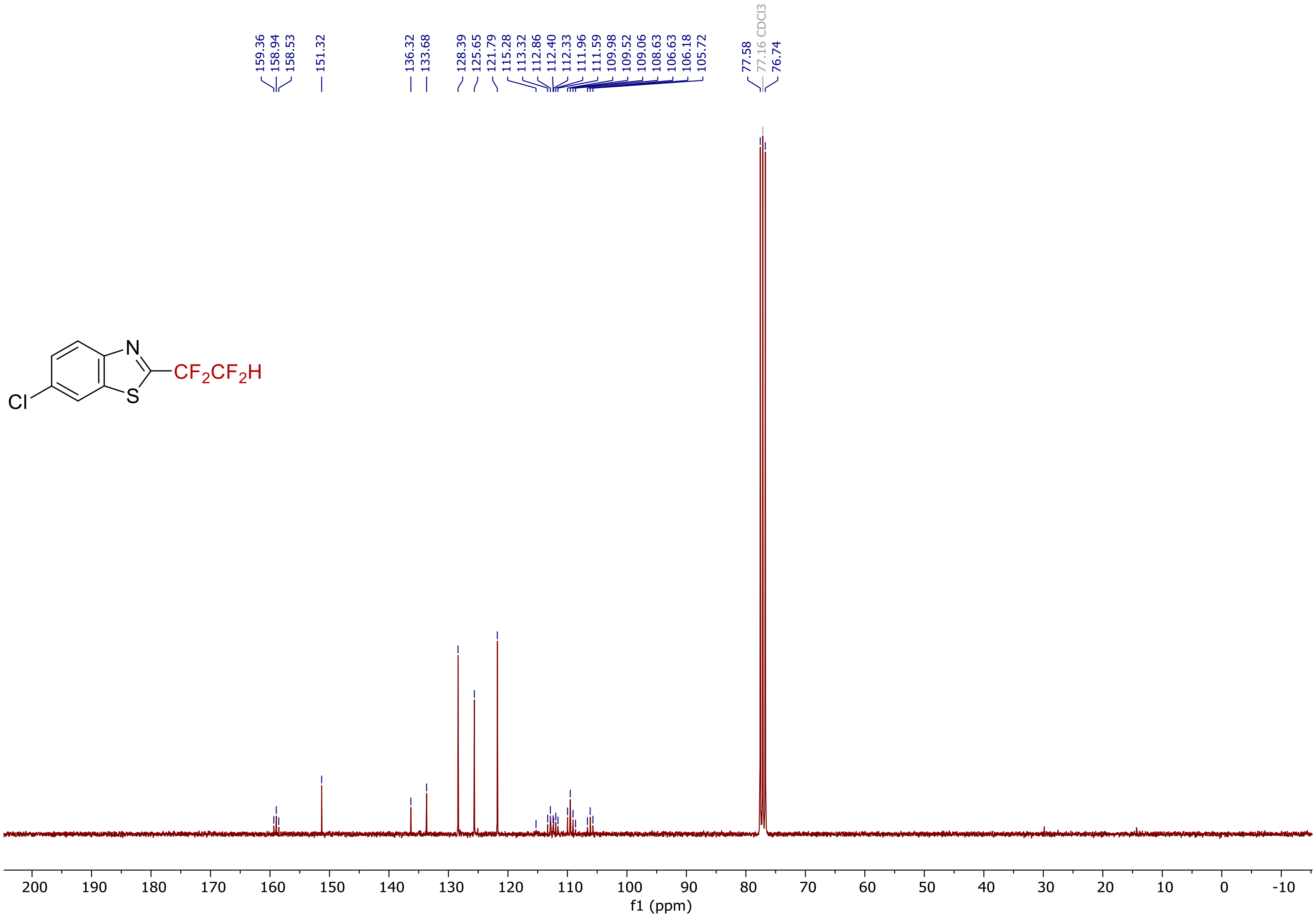

$^{19}F$ NMR (282 MHz, $CDCl_3$)

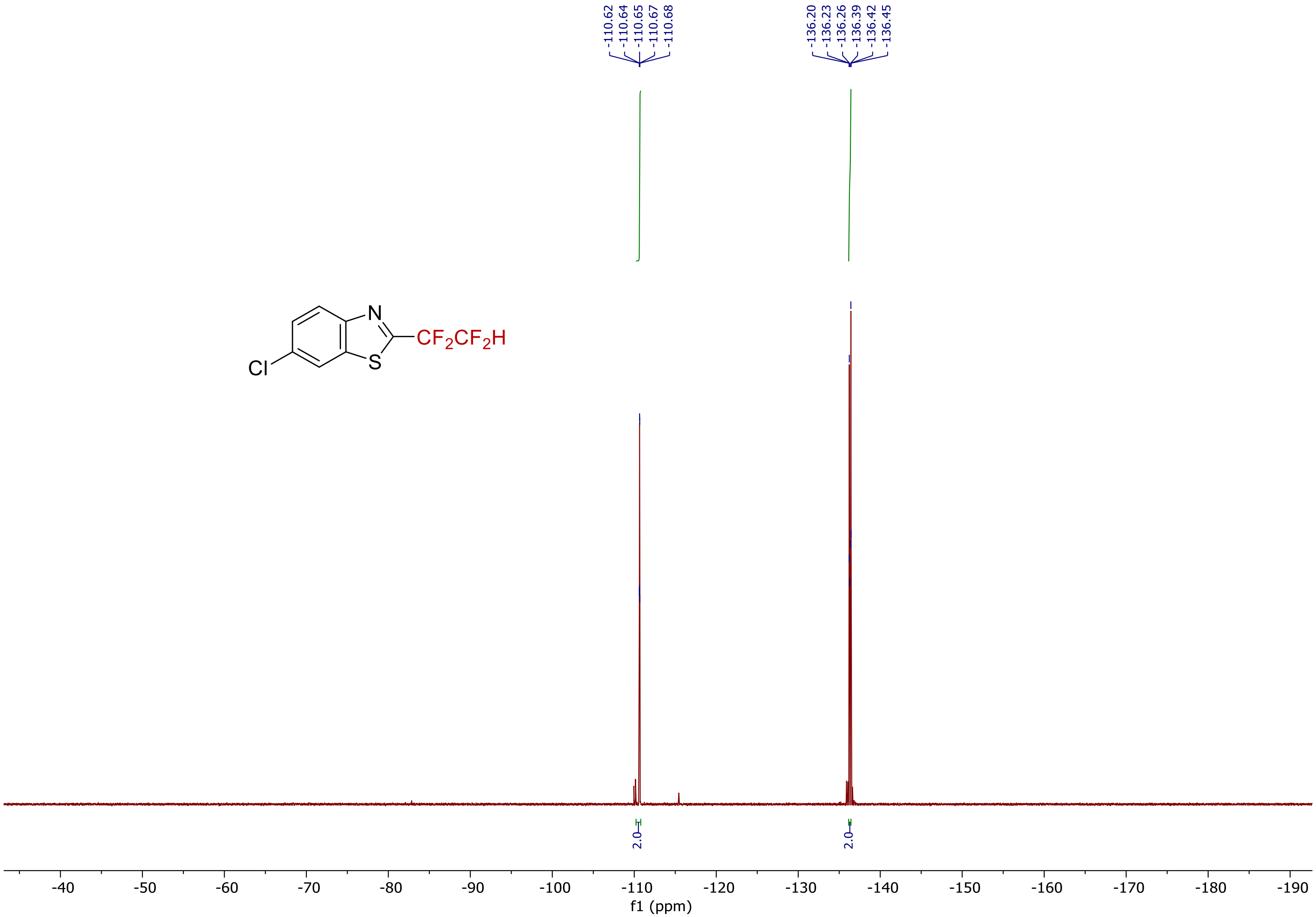

**_N_-(4-chloro-2-(1,1,2,2-tetrafluoroethyl)phenyl)acetamide 21 and _N_-(4-chloro-3-(1,1,2,2-tetrafluoroethyl)phenyl)acetamide 21’**

$^{1}$H NMR (300 MHz, $CDCl_3$)

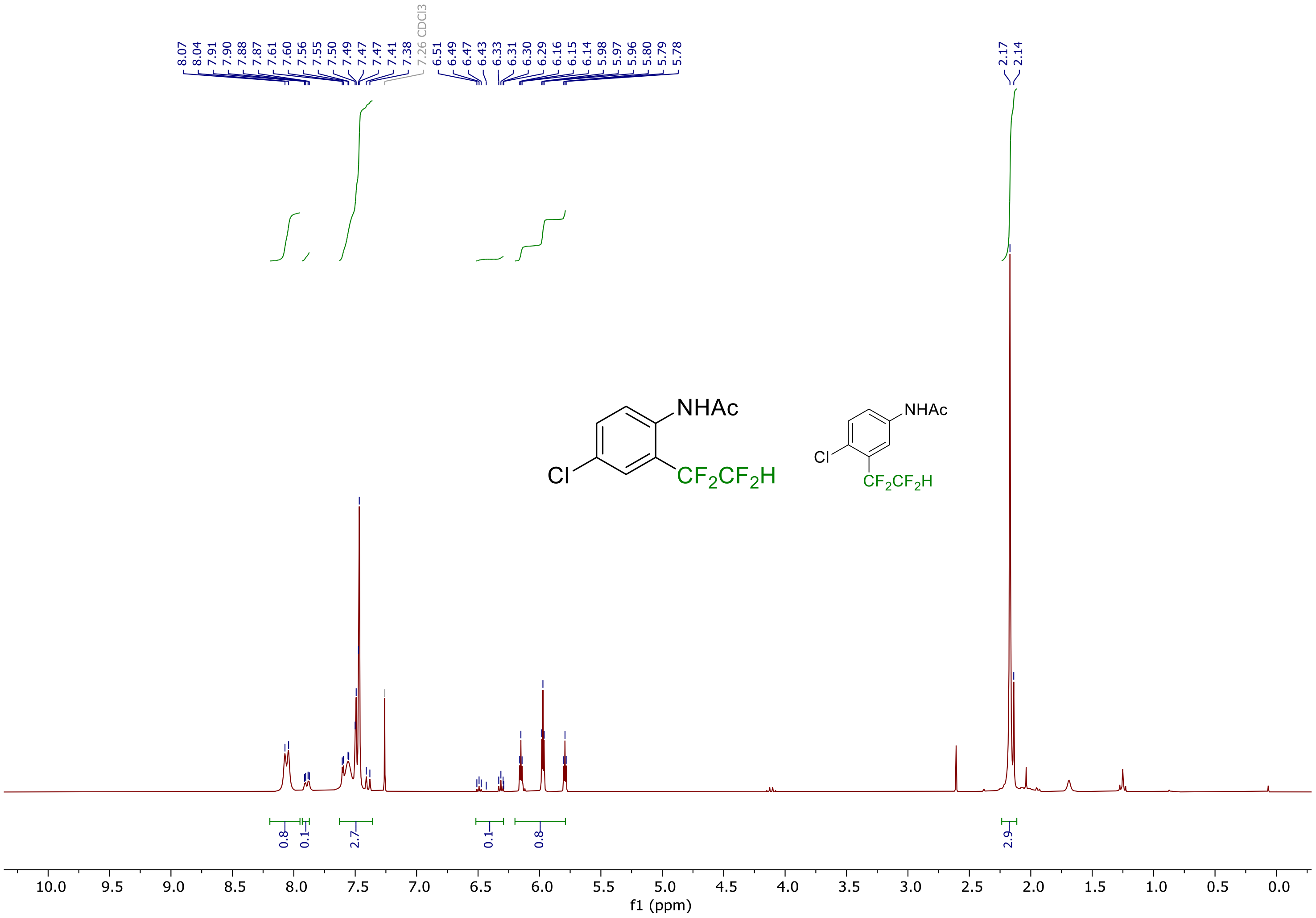

$^{13}C$ NMR (75 MHz, $CDCl_3$)

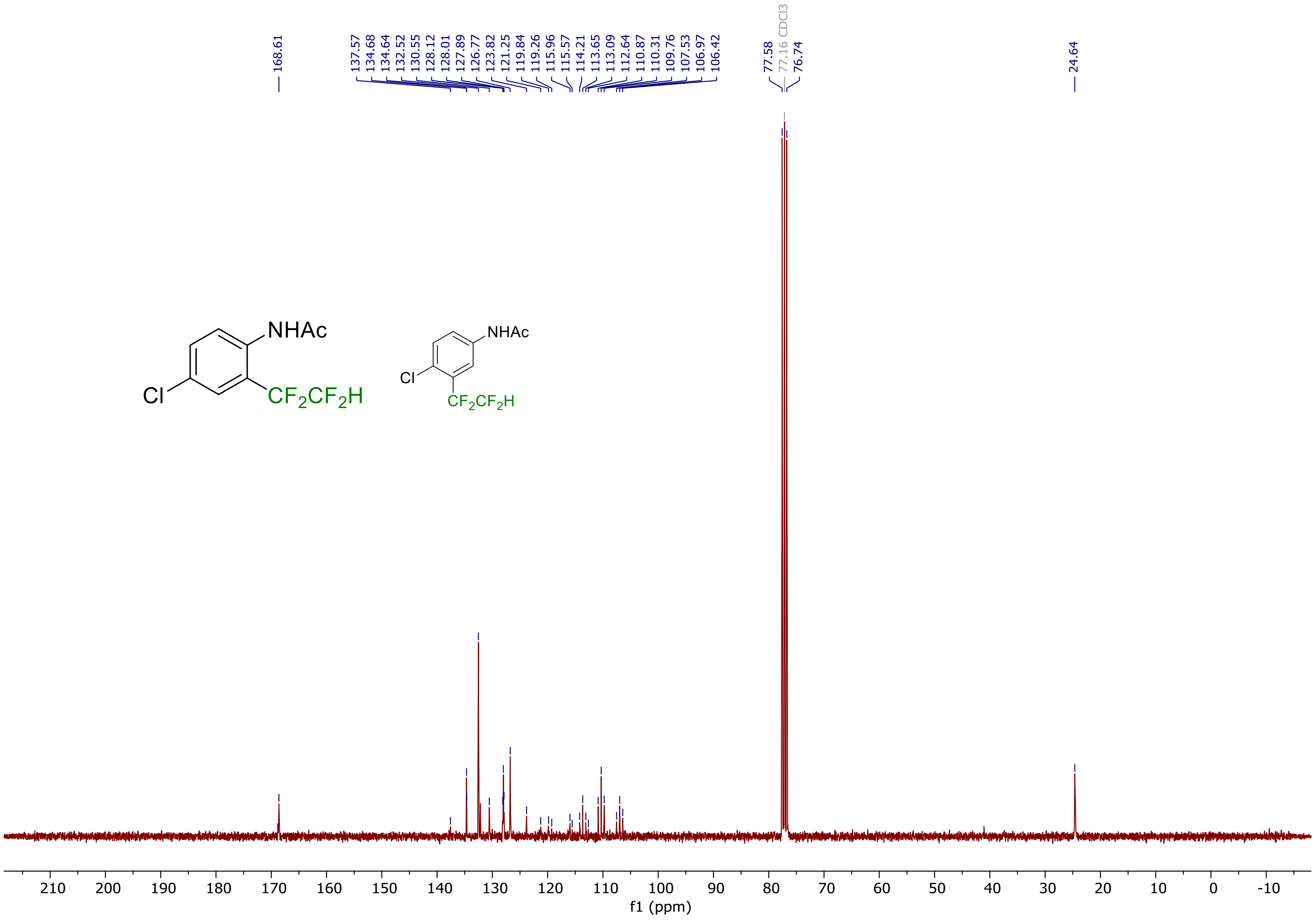

$^{19}F$ NMR (282 MHz, $CDCl_3$)

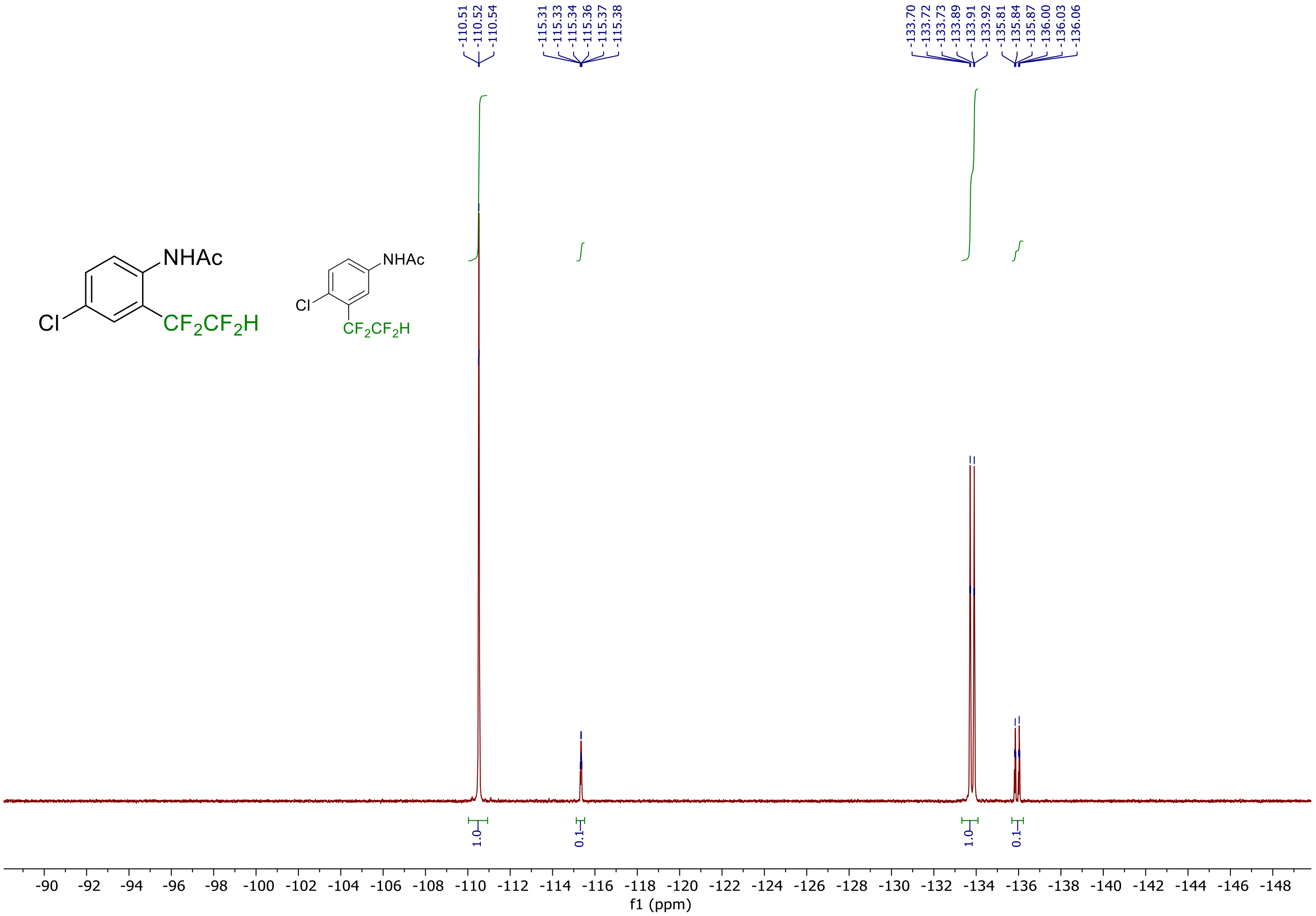

**1-(Tert-butyl)-4-iodo-2-(1,1,2,2-tetrafluoroethyl)benzene 22**

$^{1}$H NMR (300 MHz, $CDCl_3$)

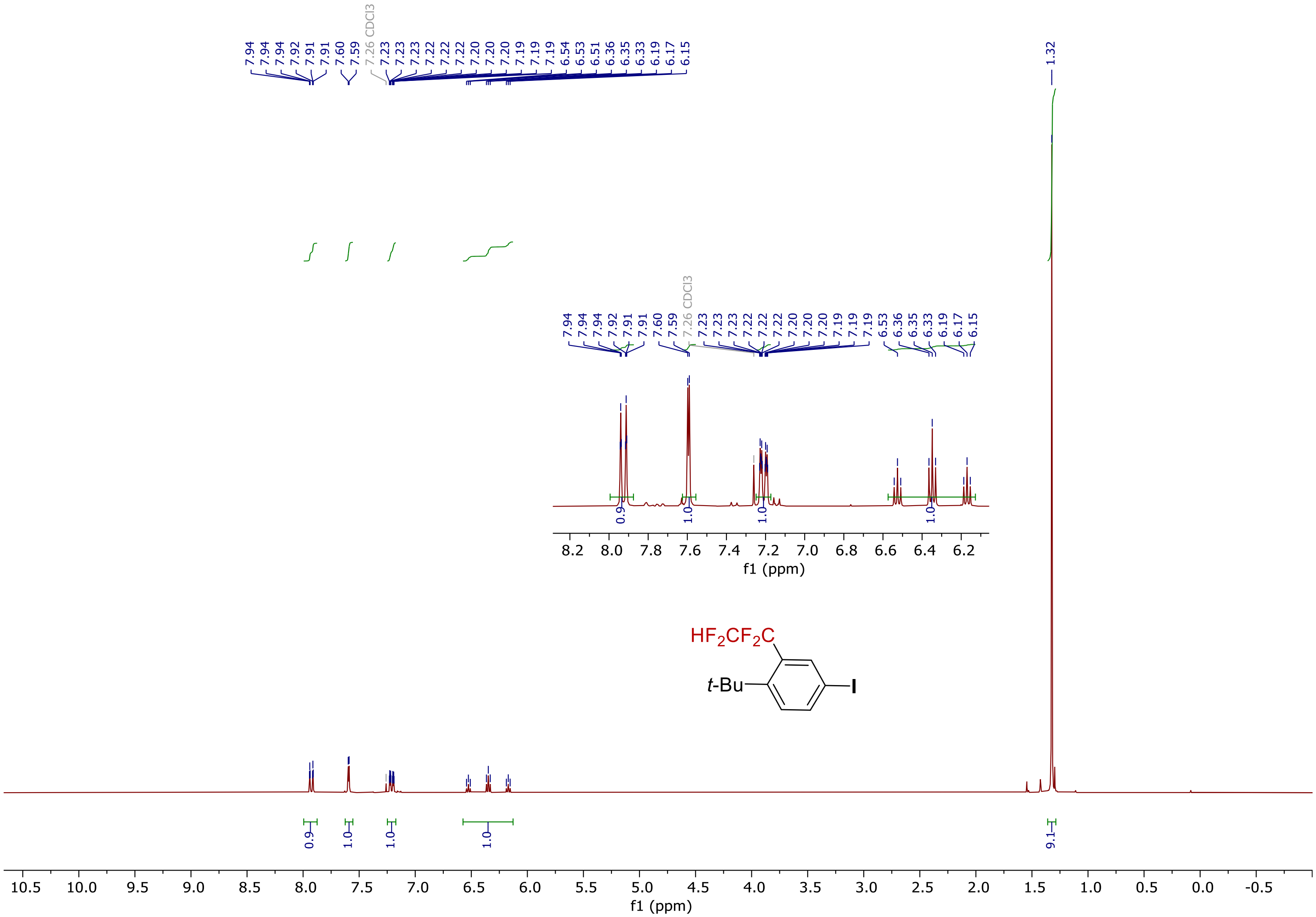

$^{13}C$ NMR (75 MHz, $CDCl_3$)

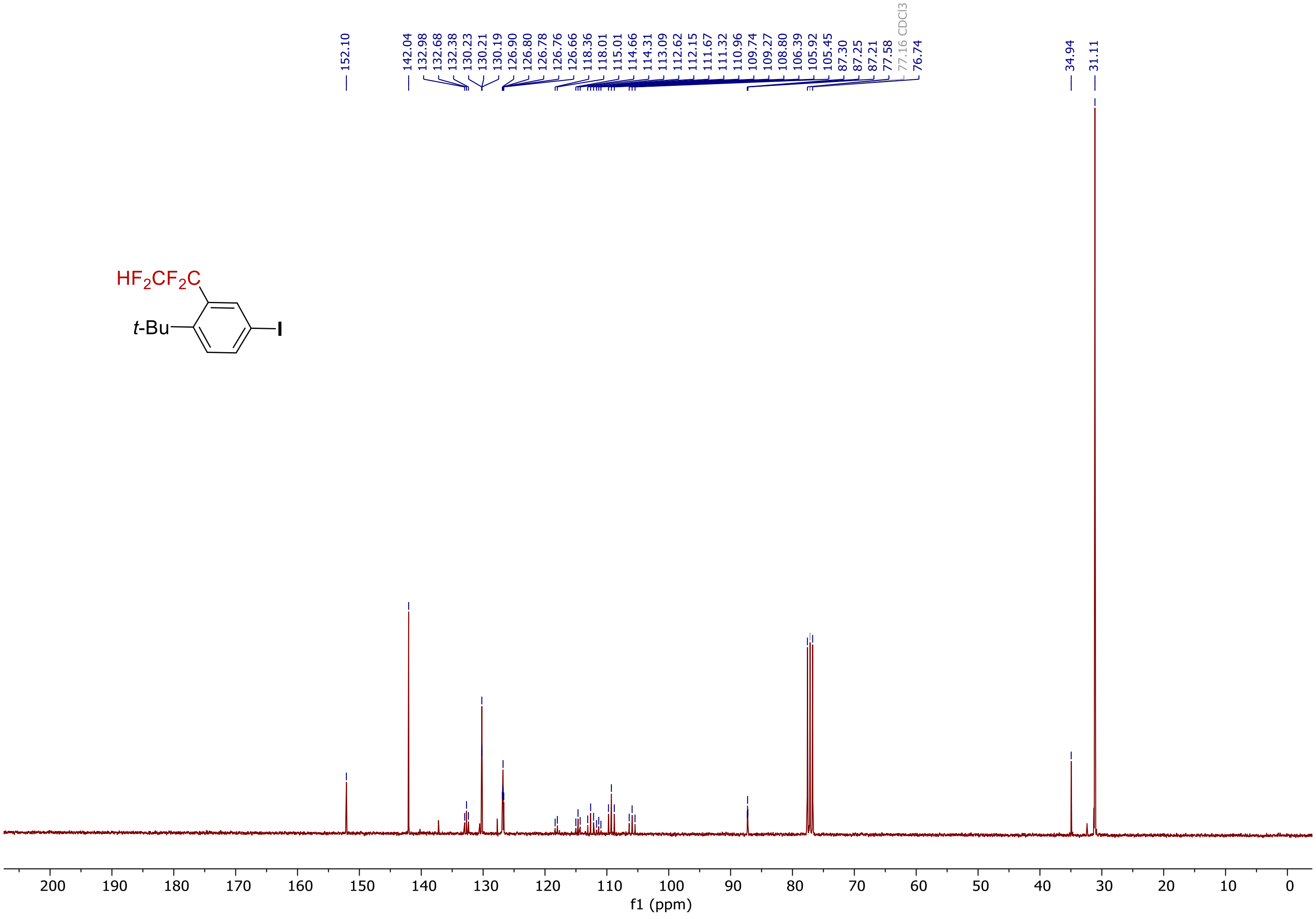

$^{19}F$ NMR (282 MHz, $CDCl_3$)

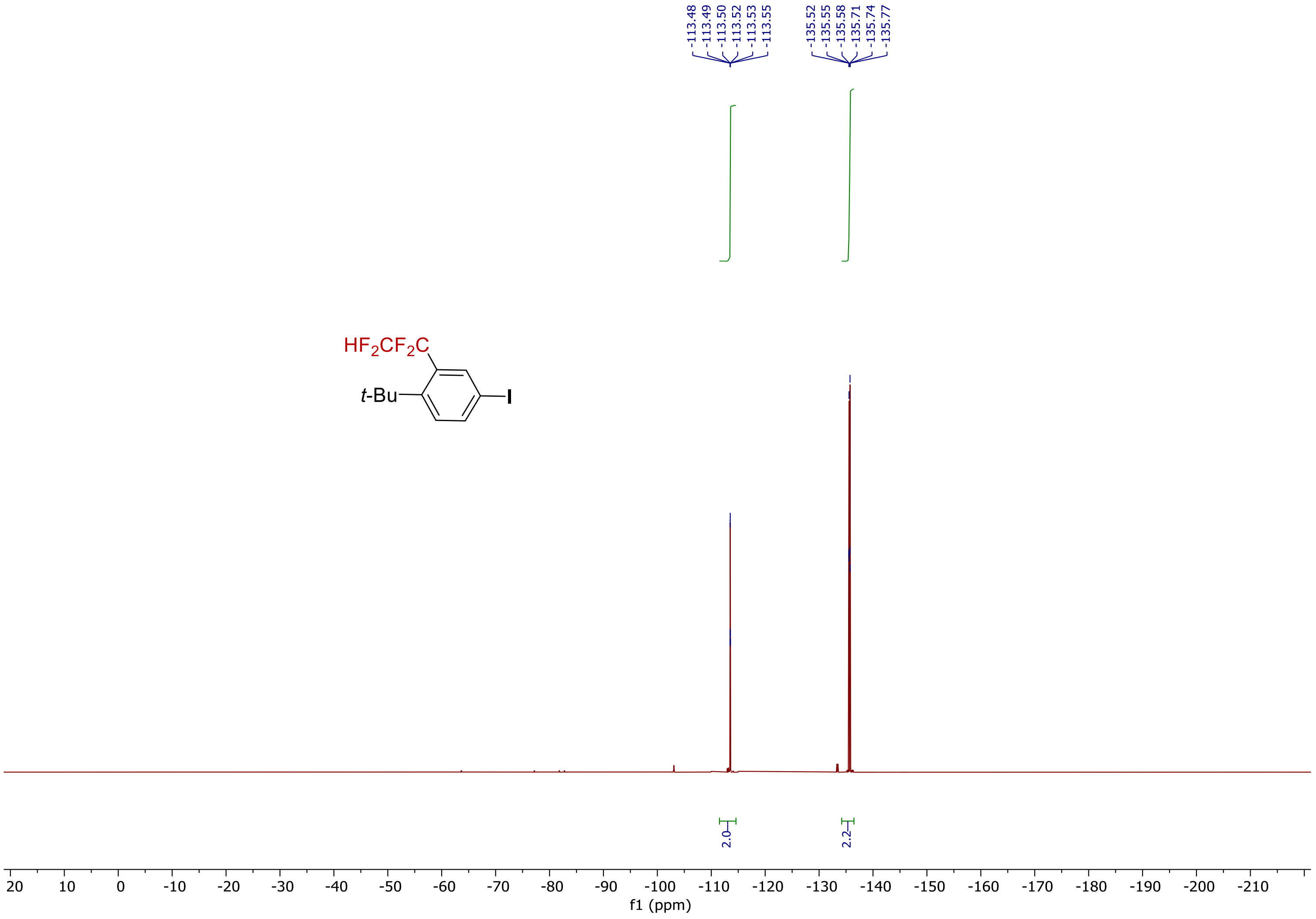

**8-(1,1-Difluoroethyl)-7-isopropyl-1,3-dimethyl-3,7-dihydro-1*H*-purine-2,6-dione 23**

$^{1}$H NMR (300 MHz, $CDCl_3$)

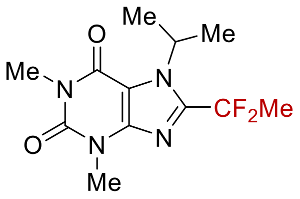


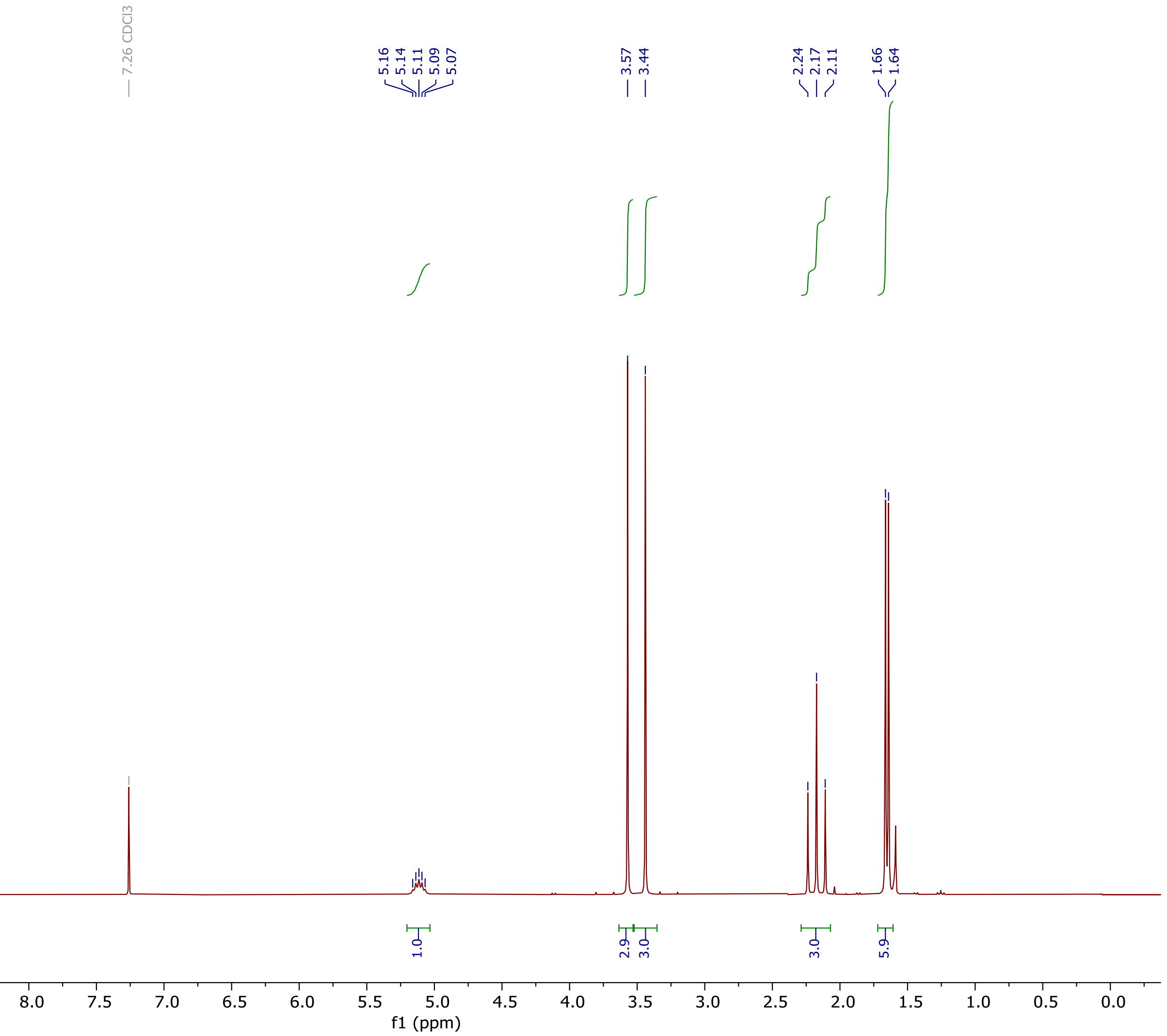

$^{13}C$ NMR (75 MHz, $CDCl_3$)

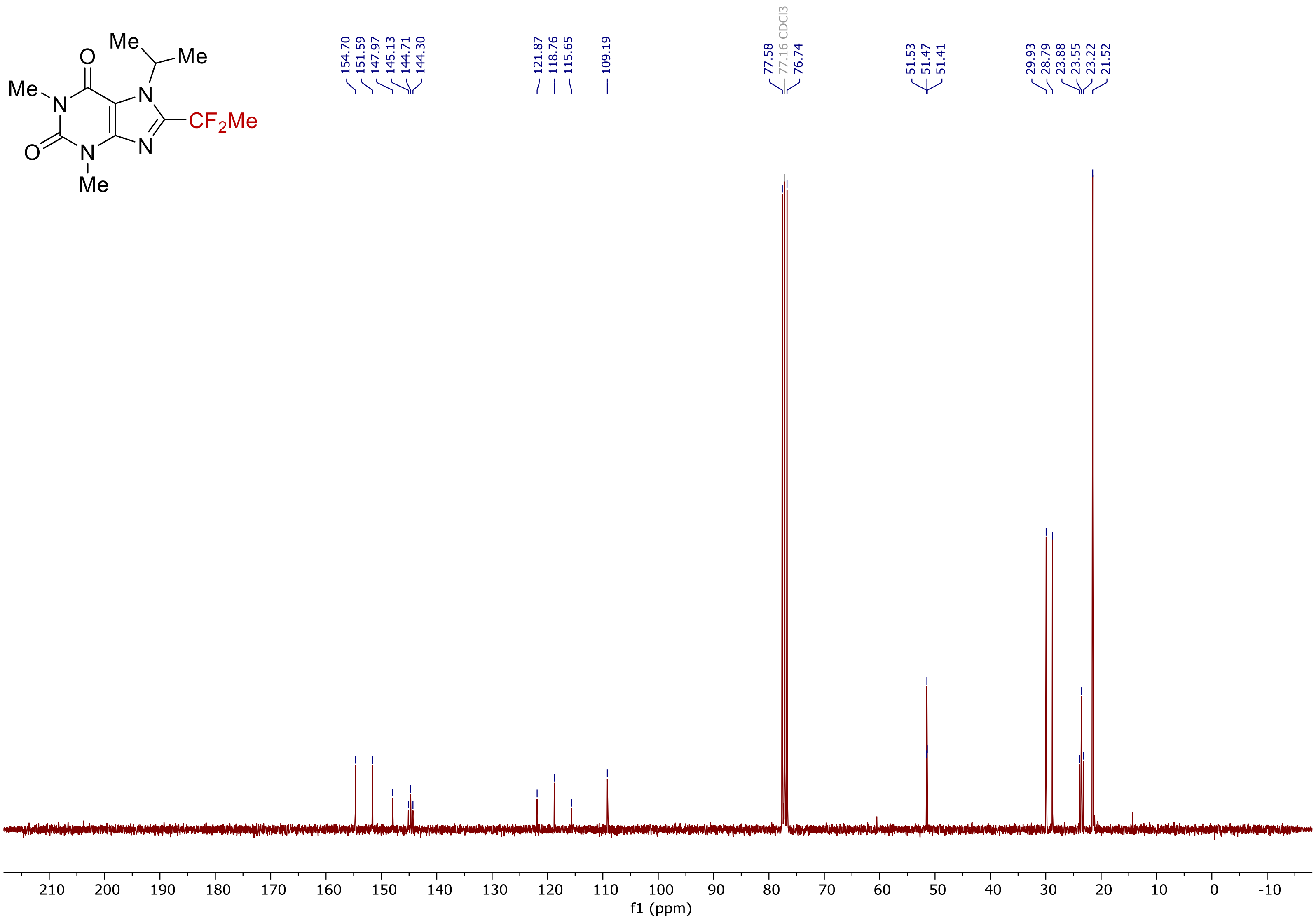

$^{19}F$ NMR (282 MHz, $CDCl_3$)

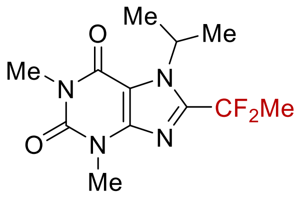


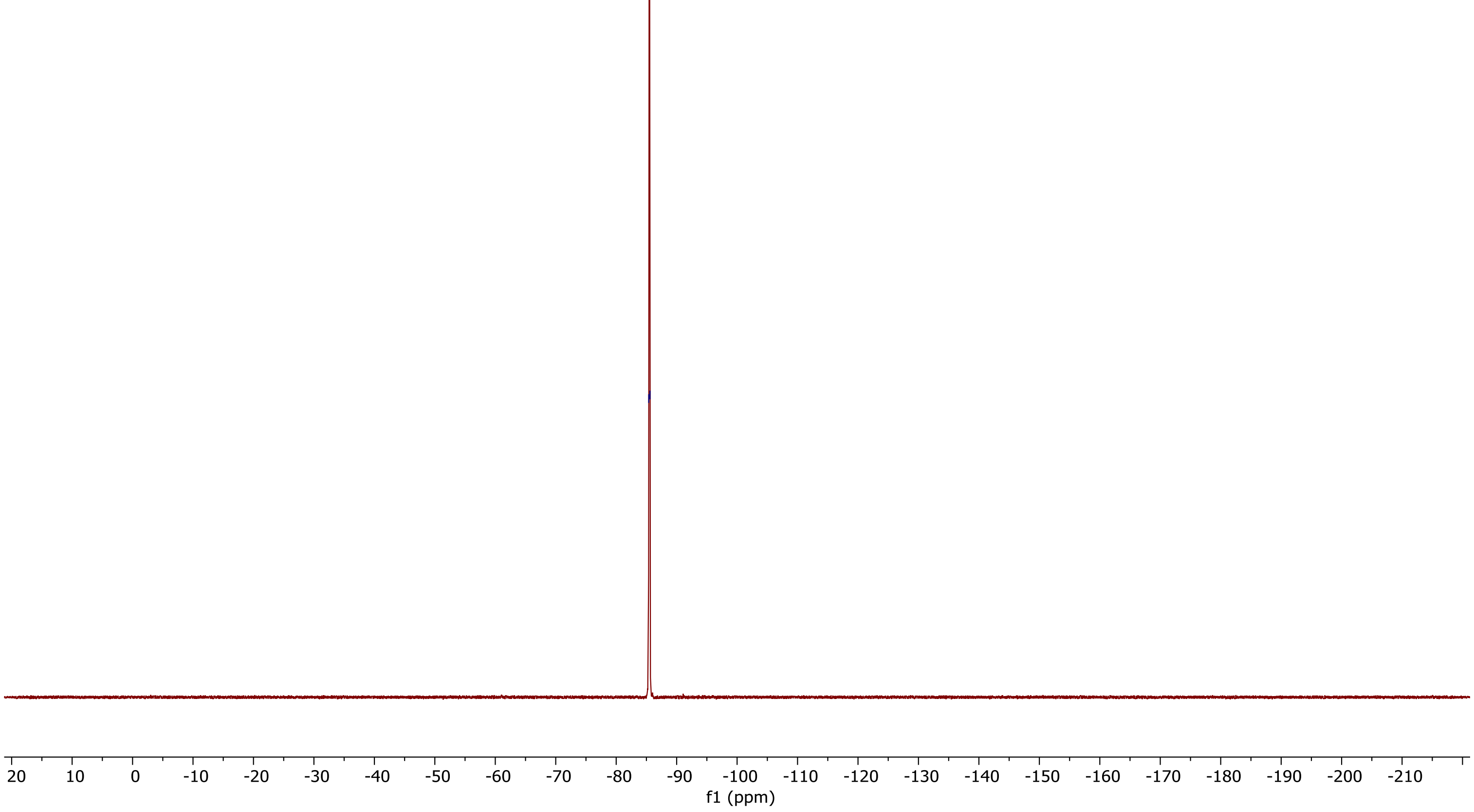

**7-Benzyl-8-(1,1-difluoroethyl)-1,3-dimethyl-3,7-dihydro-1*H*-purine-2,6-dione 24**

$^1$H NMR (400 MHz, $CDCl_3$)

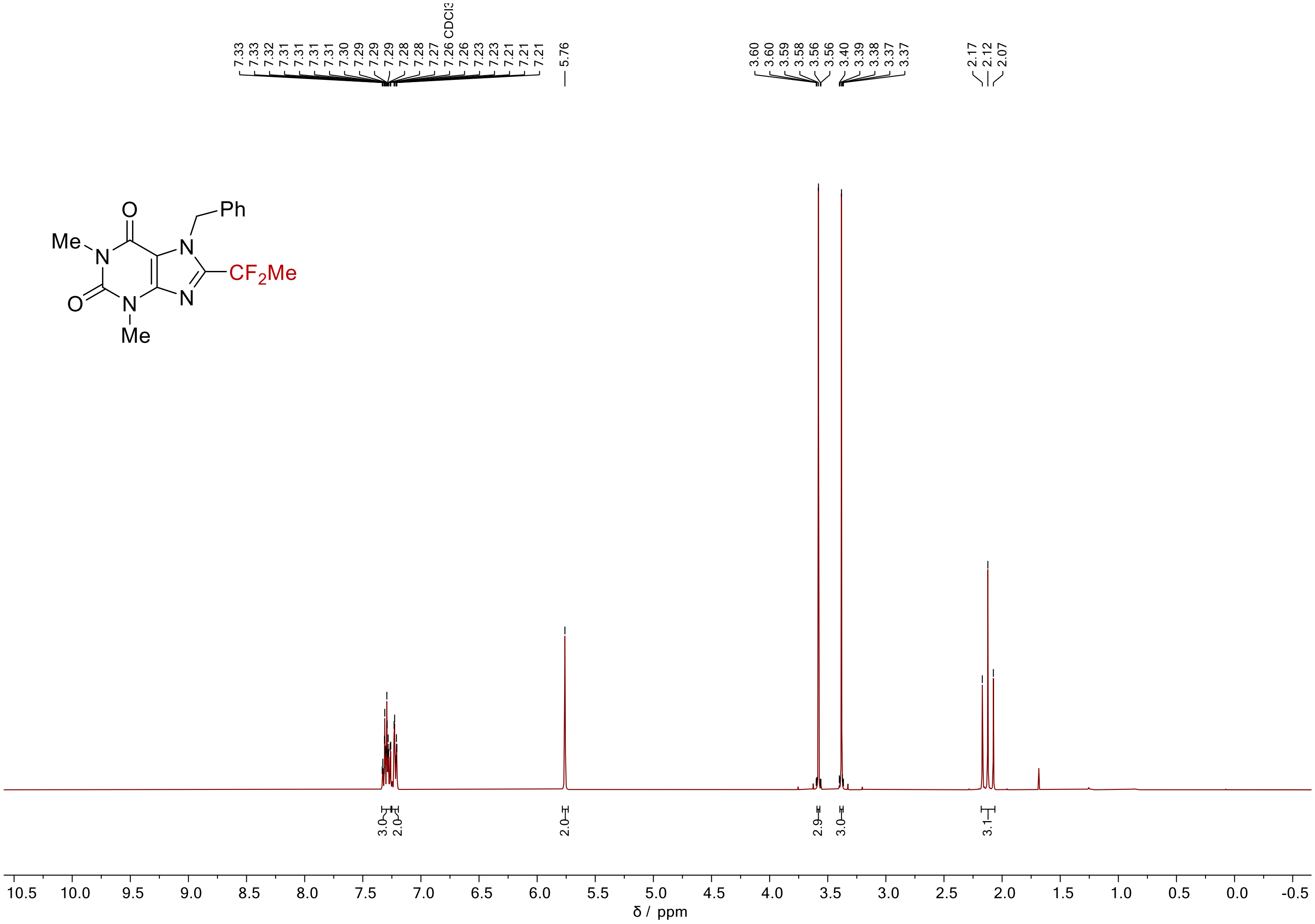

$^{13}C$ NMR (101 MHz, $CDCl_3$)

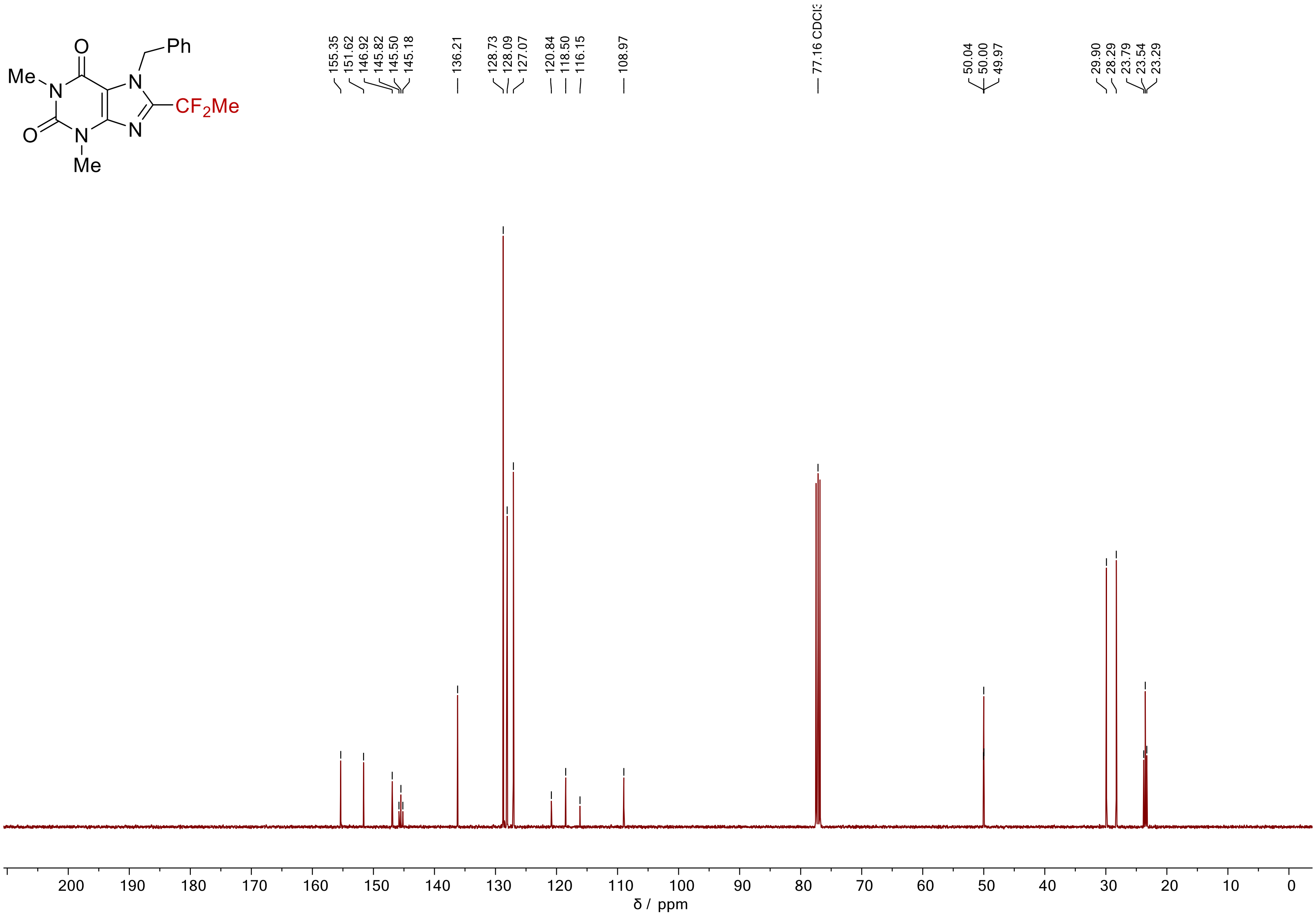

$^{19}F$ NMR (377 MHz, $CDCl_3$)

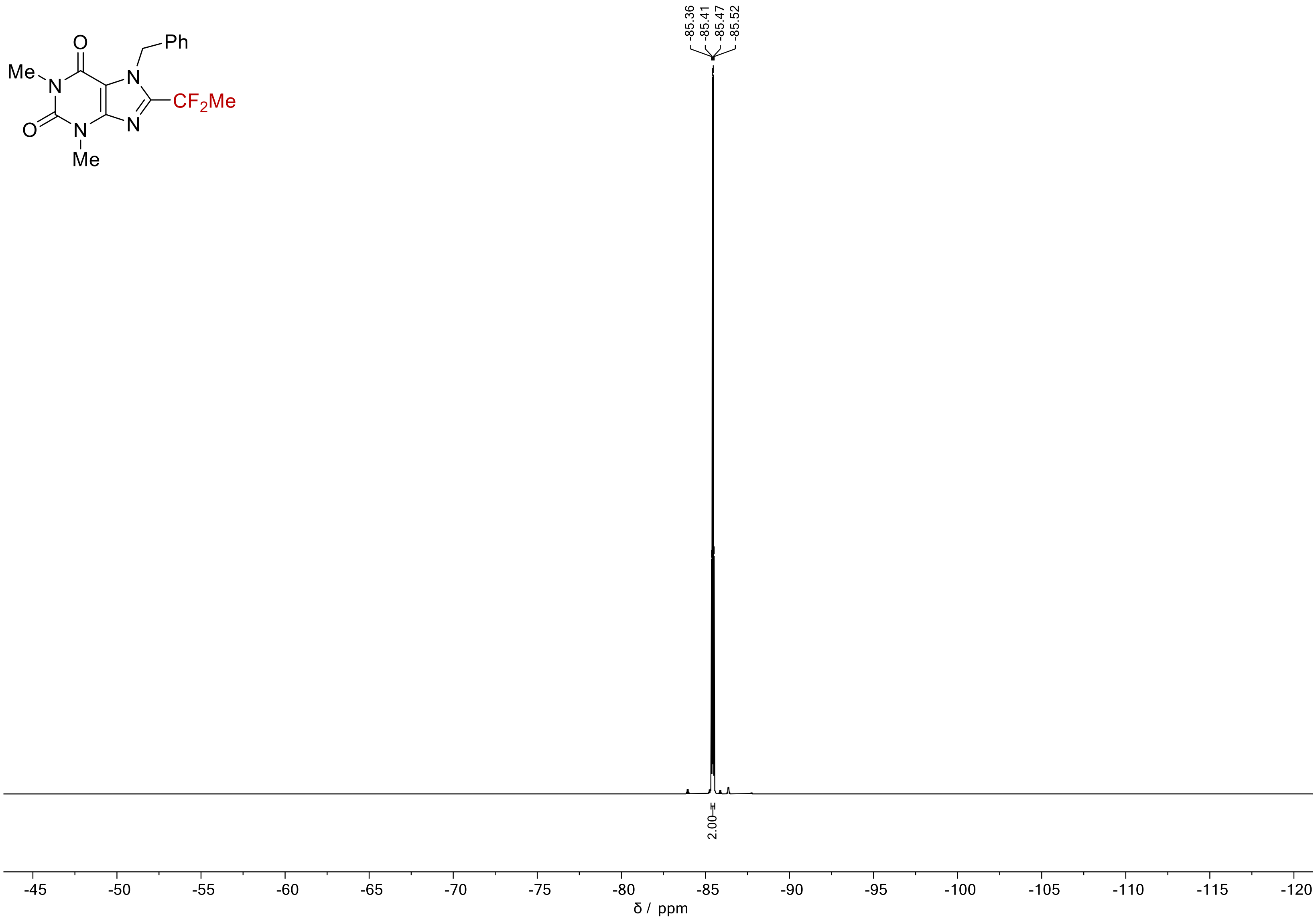

**1-Benzyl-8-(1,1-difluoroethyl)-3,7-dimethyl-3,7-dihydro-1*H*-purine-2,6-dione 25**

$^{1}$H NMR (400 MHz, $CDCl_3$)

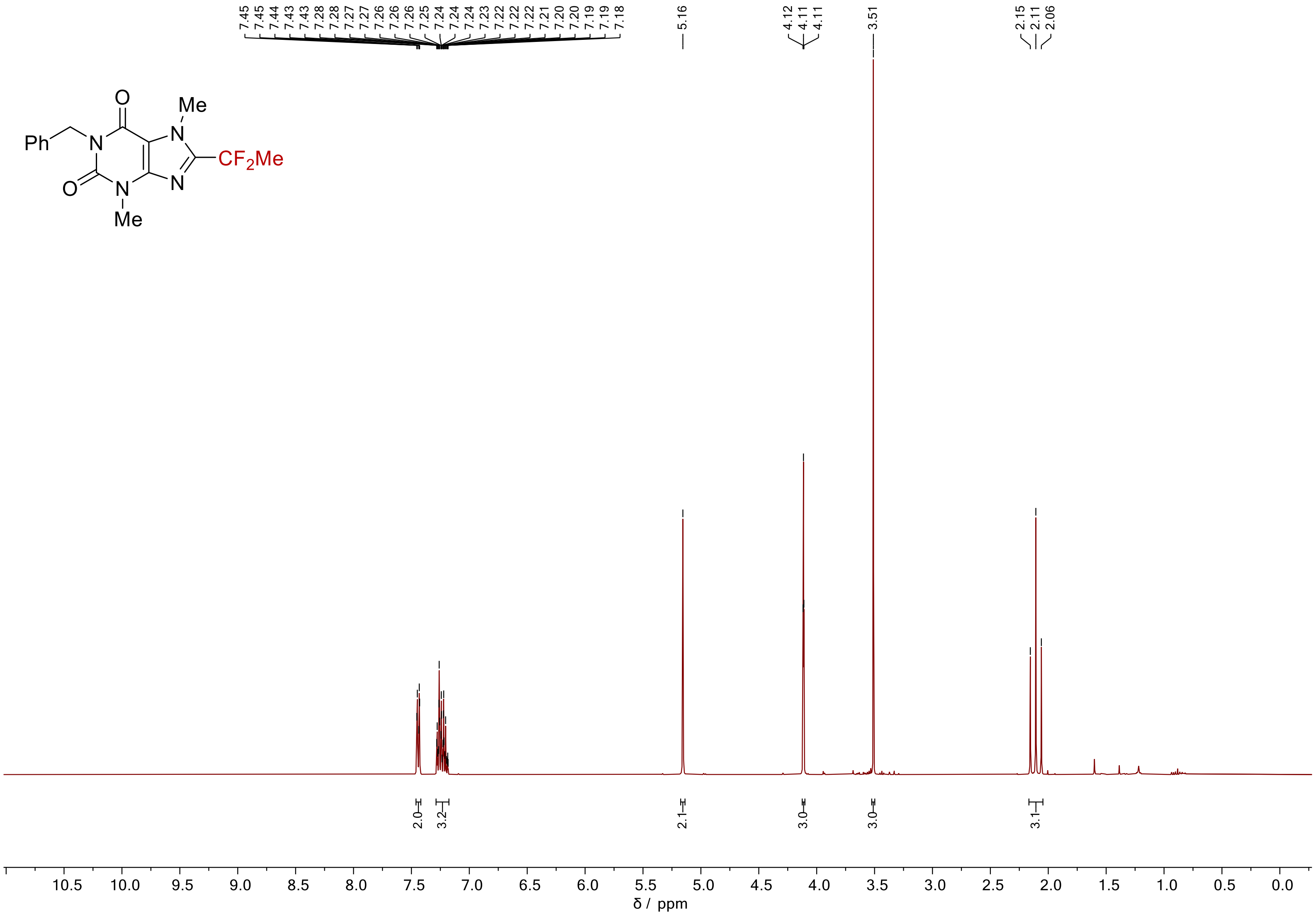

$^{13}$C NMR (101 MHz, $CDCl_3$)

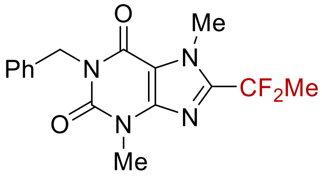

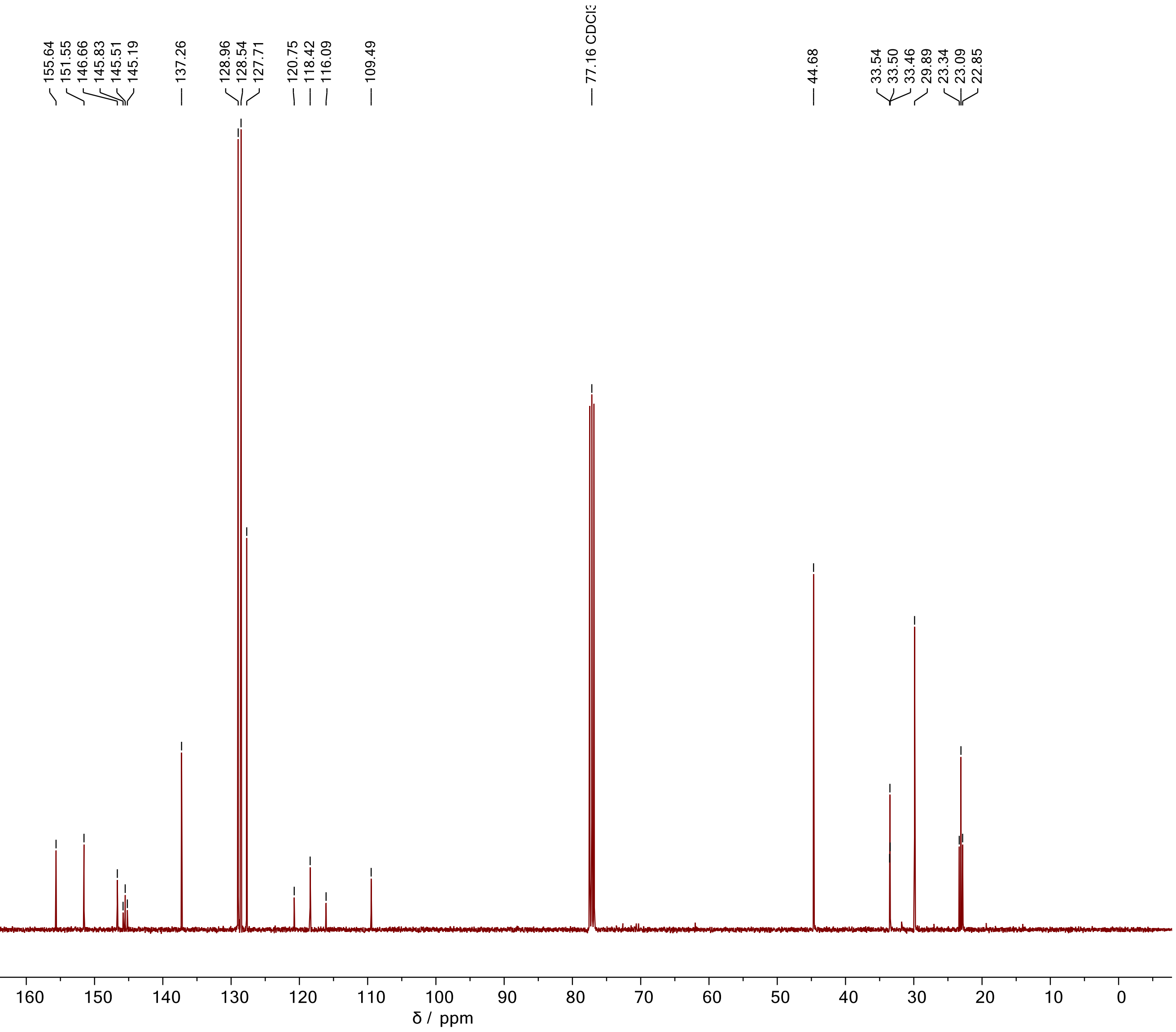

$^{19}F$ NMR (377 MHz, $CDCl_3$)

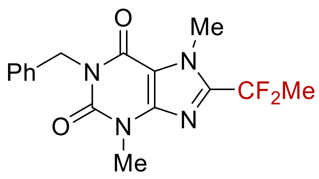


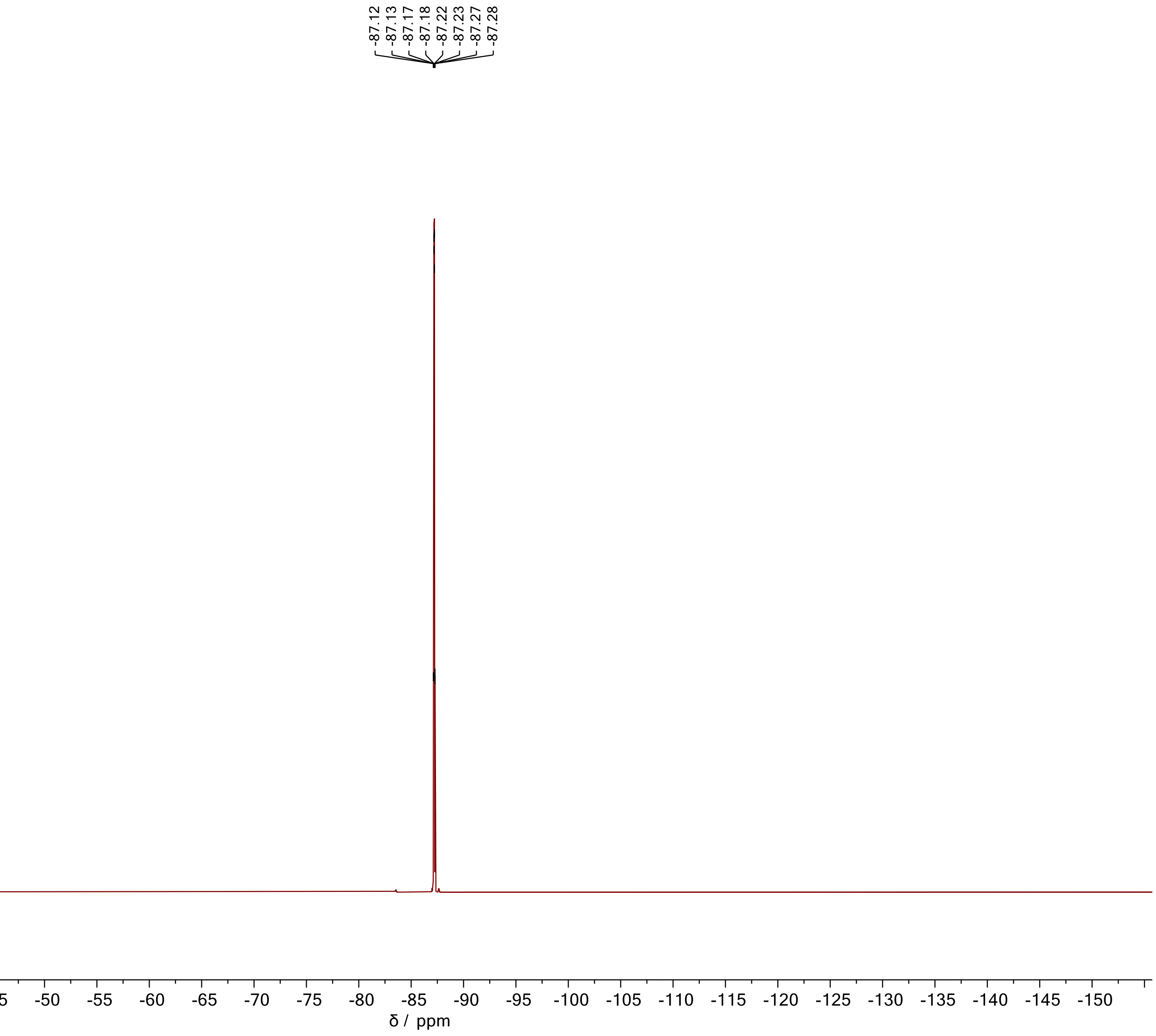

**8-(1,1-Difluoroethyl)-1,3-dimethyl-7-(*p*-tolyl)-3,7-dihydro-1*H*-purine-2,6-dione 26**

$^{1}H$ NMR (300 MHz, $CDCl_3$)

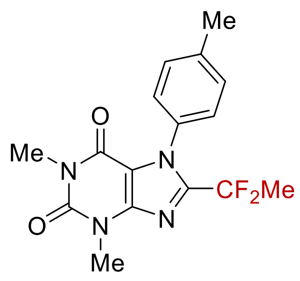


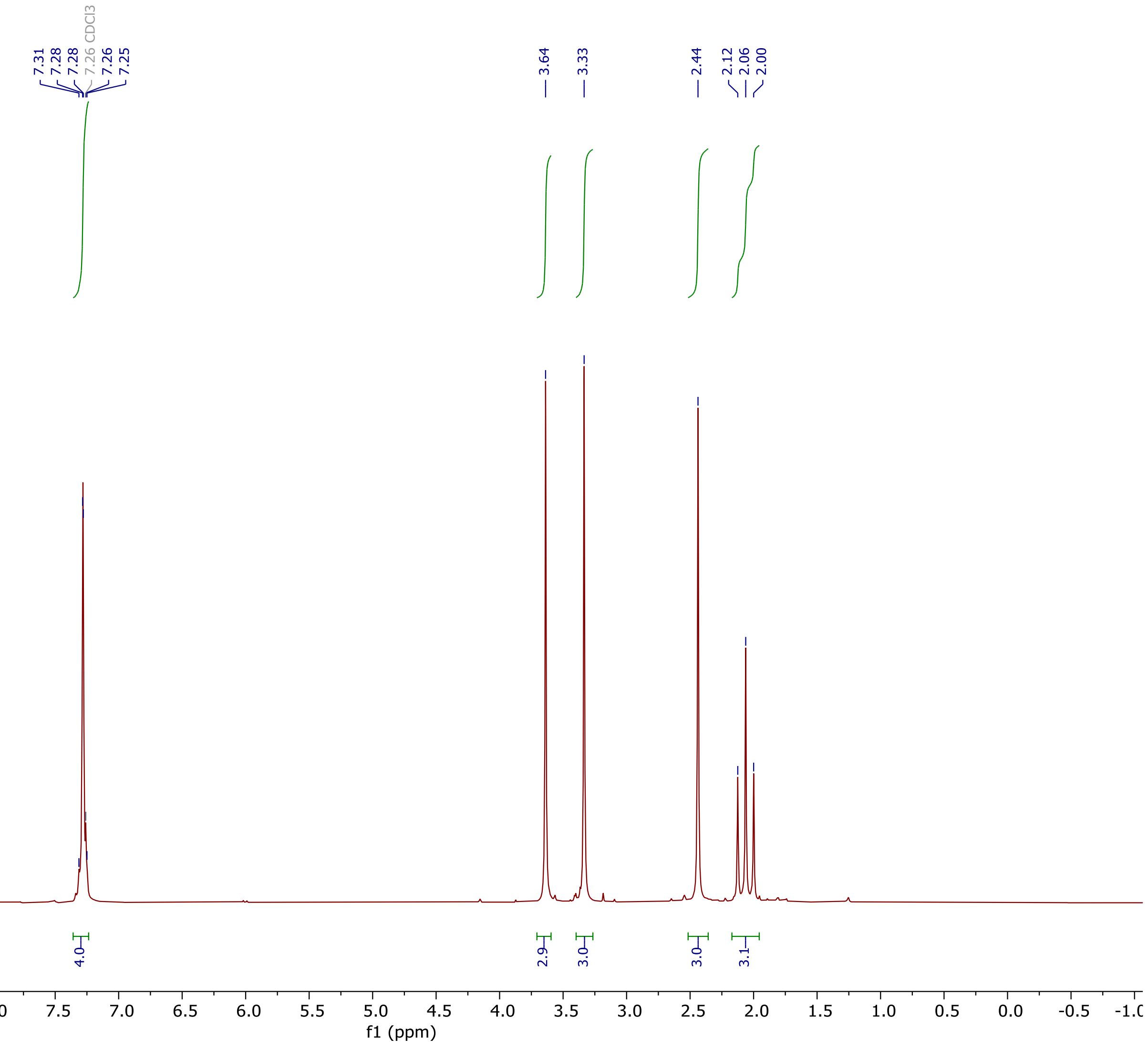

$^{13}C$ NMR (75 MHz, $CDCl_3$)

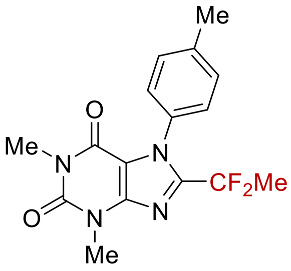


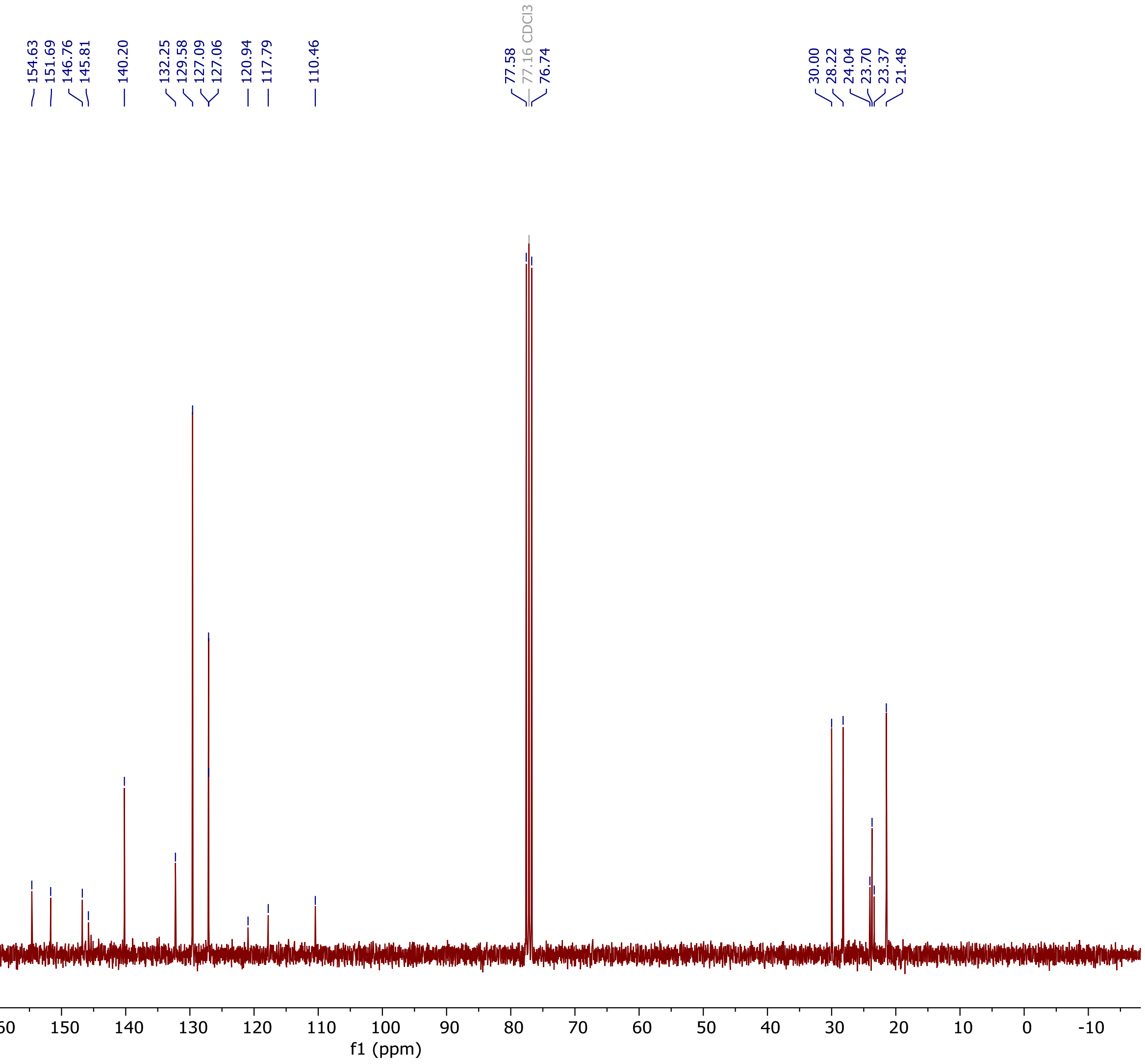

$^{19}$F NMR (282 MHz, $CDCl_3$)

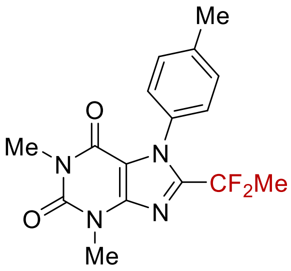

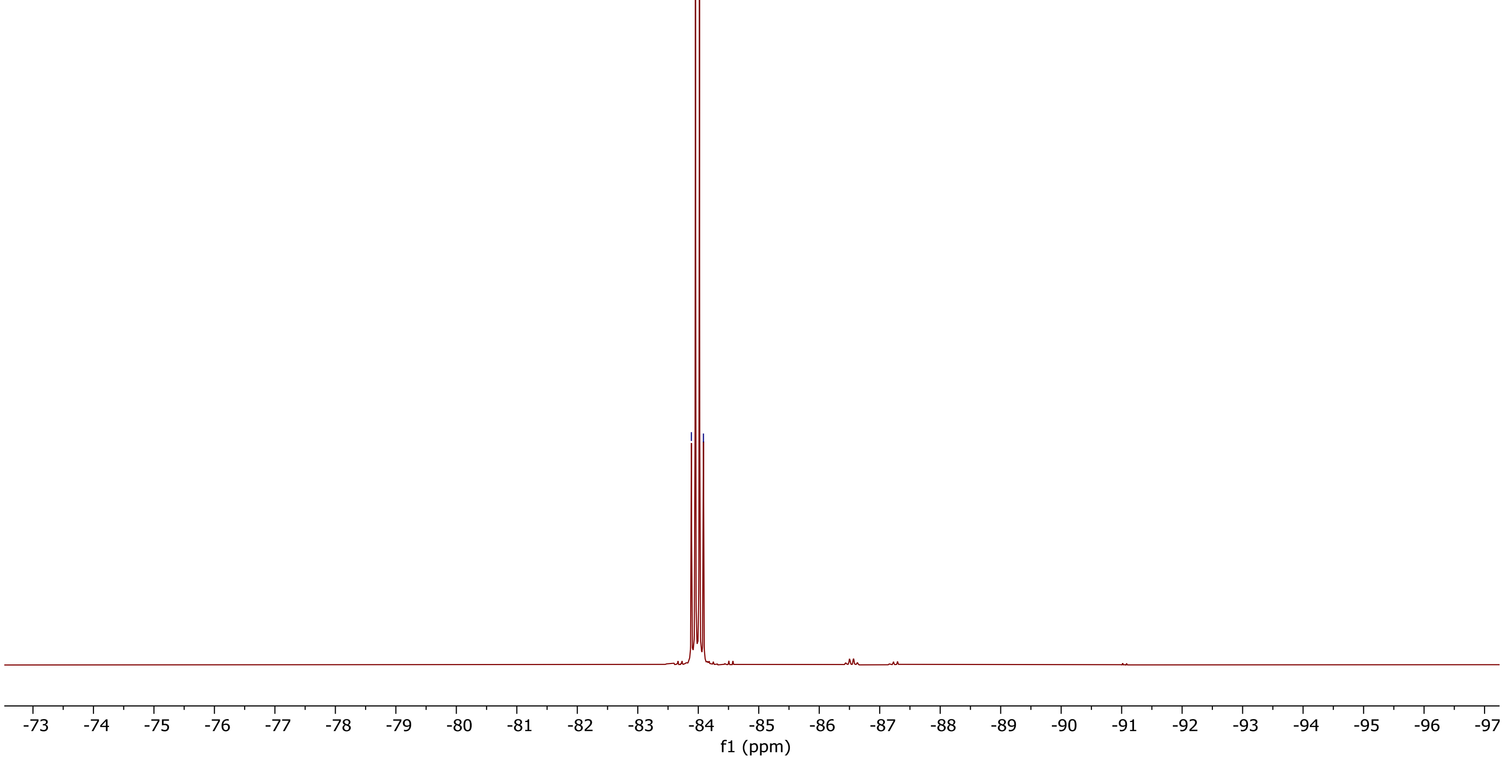

**7-((1,3-Dioxolan-2-yl)methyl)-8-(1,1-difluoroethyl)-1,3-dimethyl-3,7-dihydro-1*H*-purine-2,6-dione 27**

$^1$H NMR (300 MHz, $CDCl_3$)

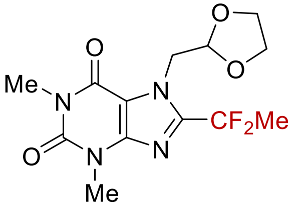

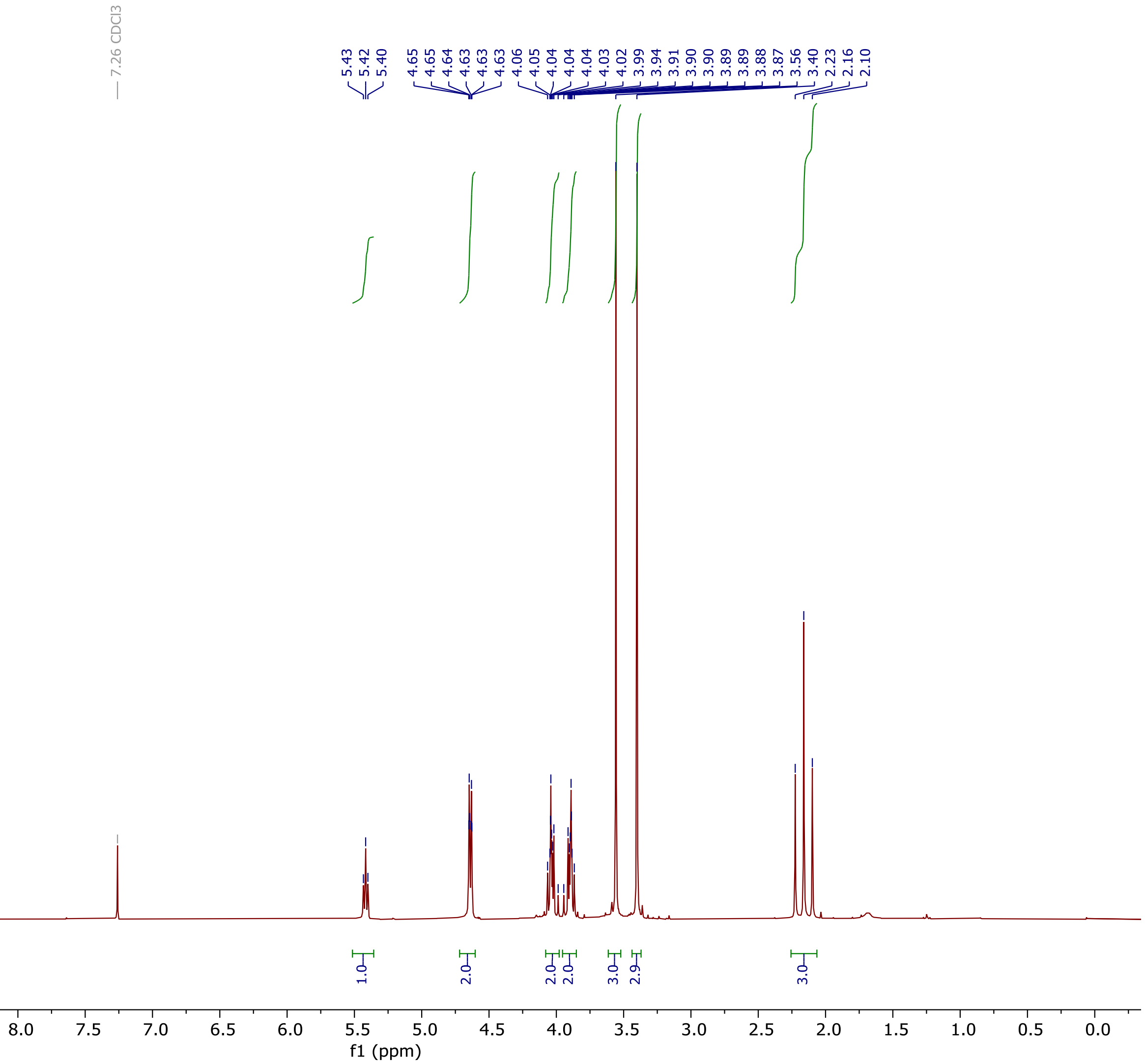

$^{13}C$ NMR (75 MHz, $CDCl_3$)

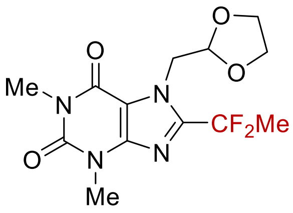


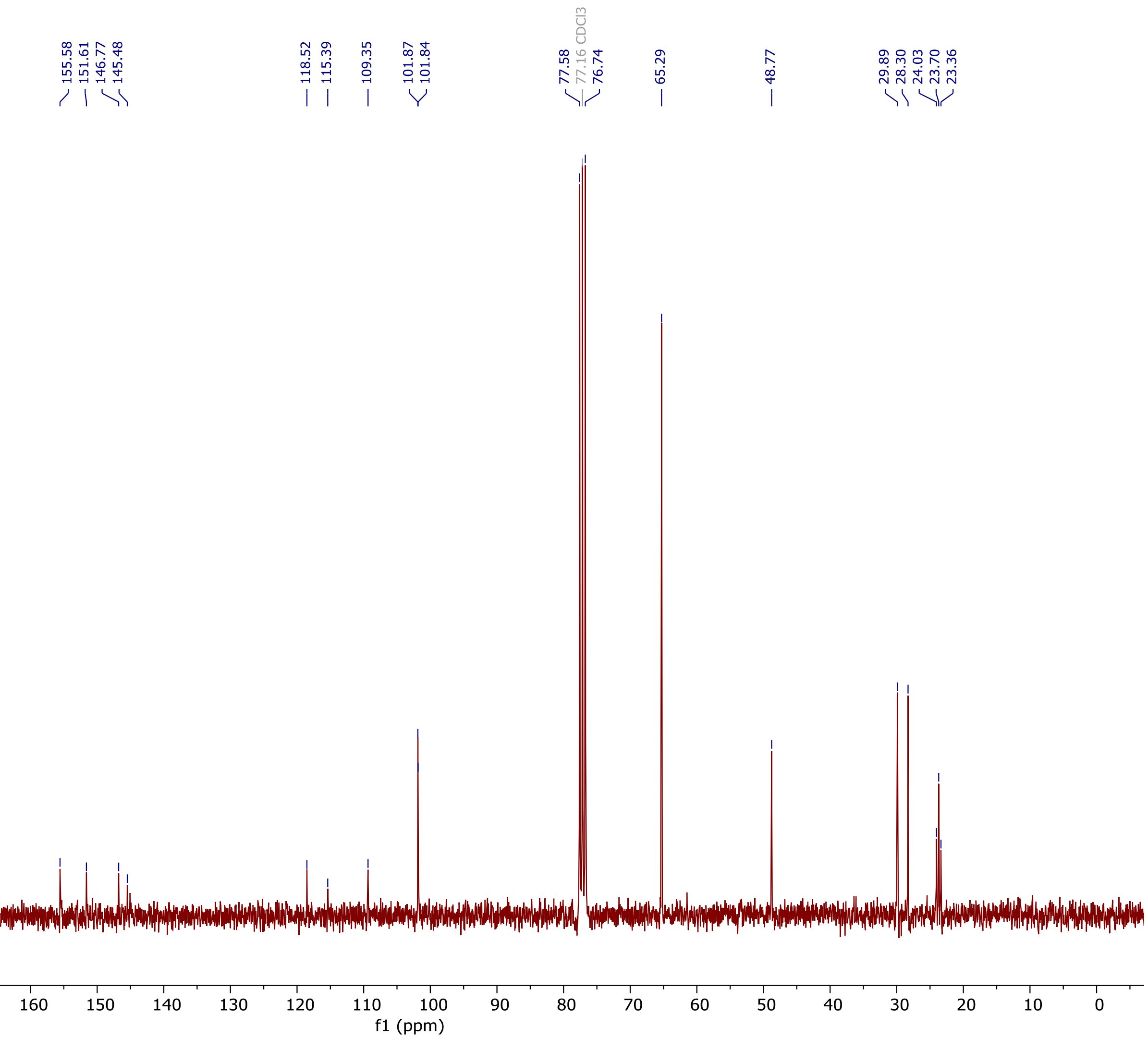

$^{19}$F NMR (282 MHz, $CDCl_3$)

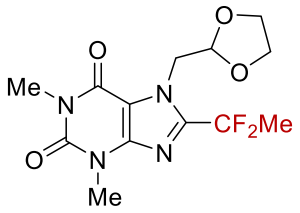


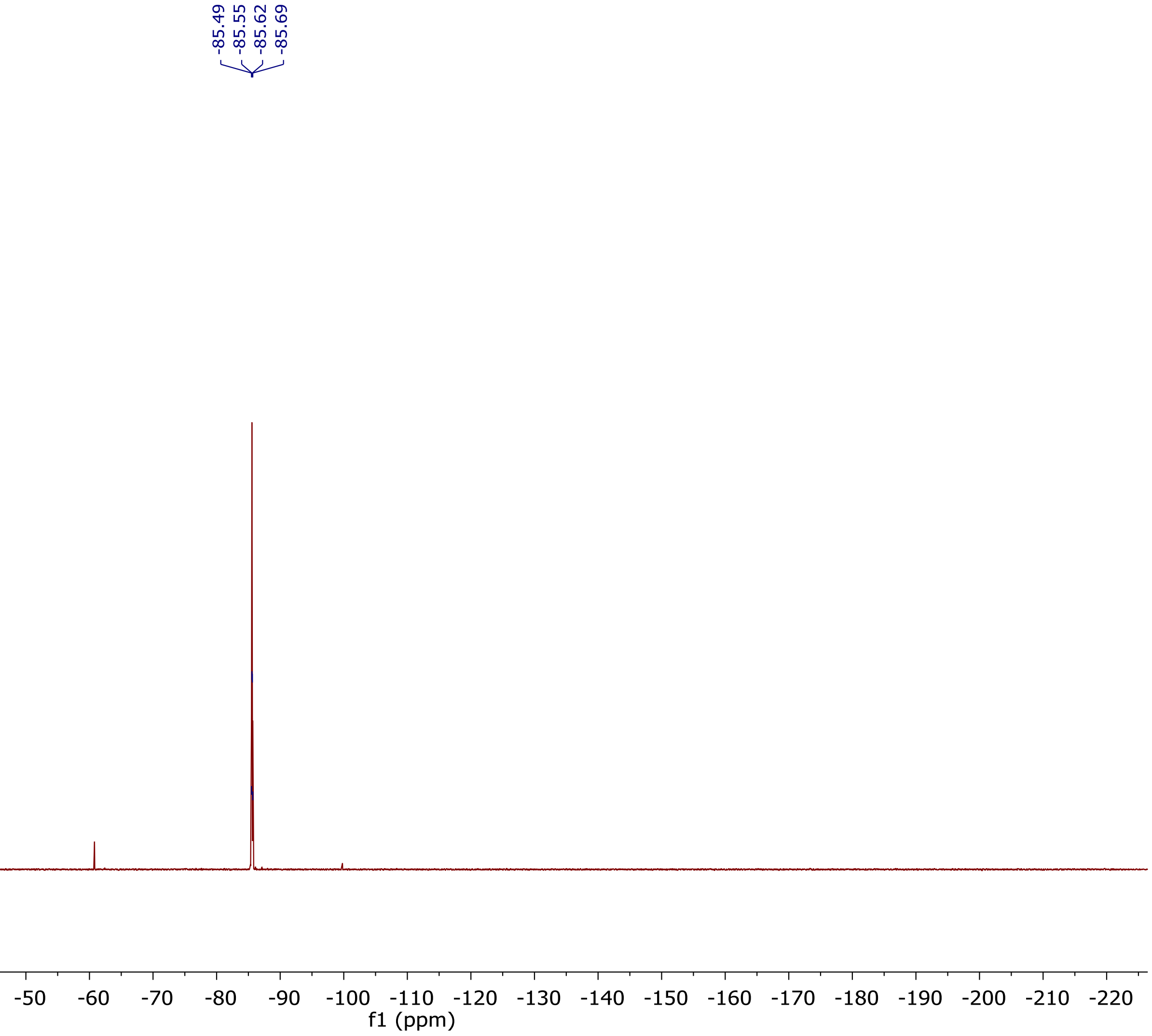

**Ethyl 2-(8-(1,1-difluoroethyl)-1,3-dimethyl-2,6-dioxo-1,2,3,6-tetrahydro-7*H*-purin-7-yl)acetate 28**

$^{1}$H NMR (300 MHz, $CDCl_3$)

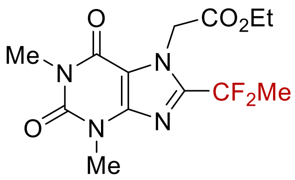


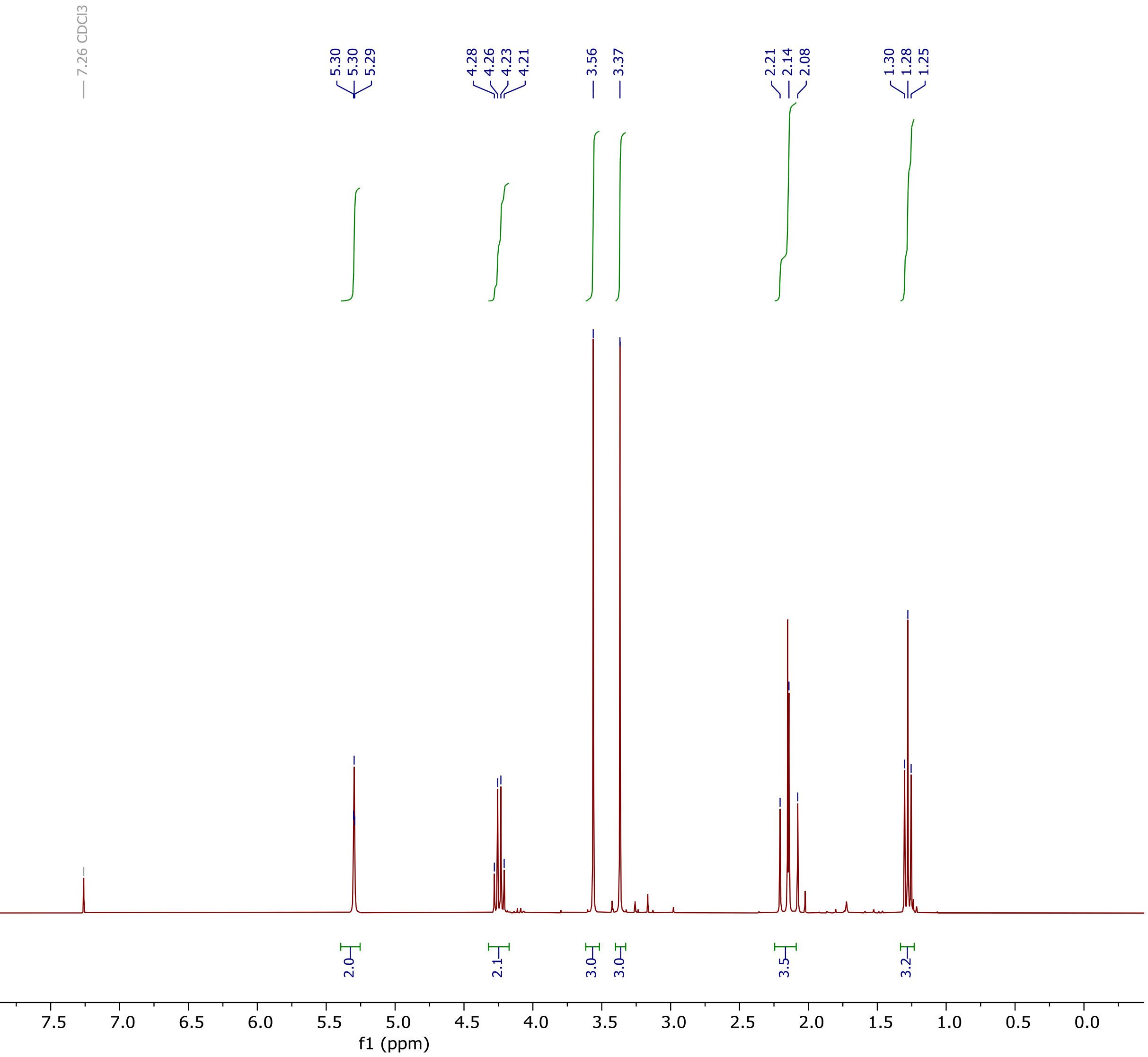

$^{13}C$ NMR (75 MHz, $CDCl_3$)

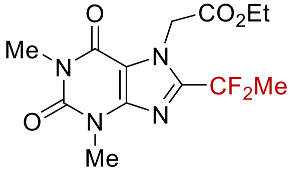


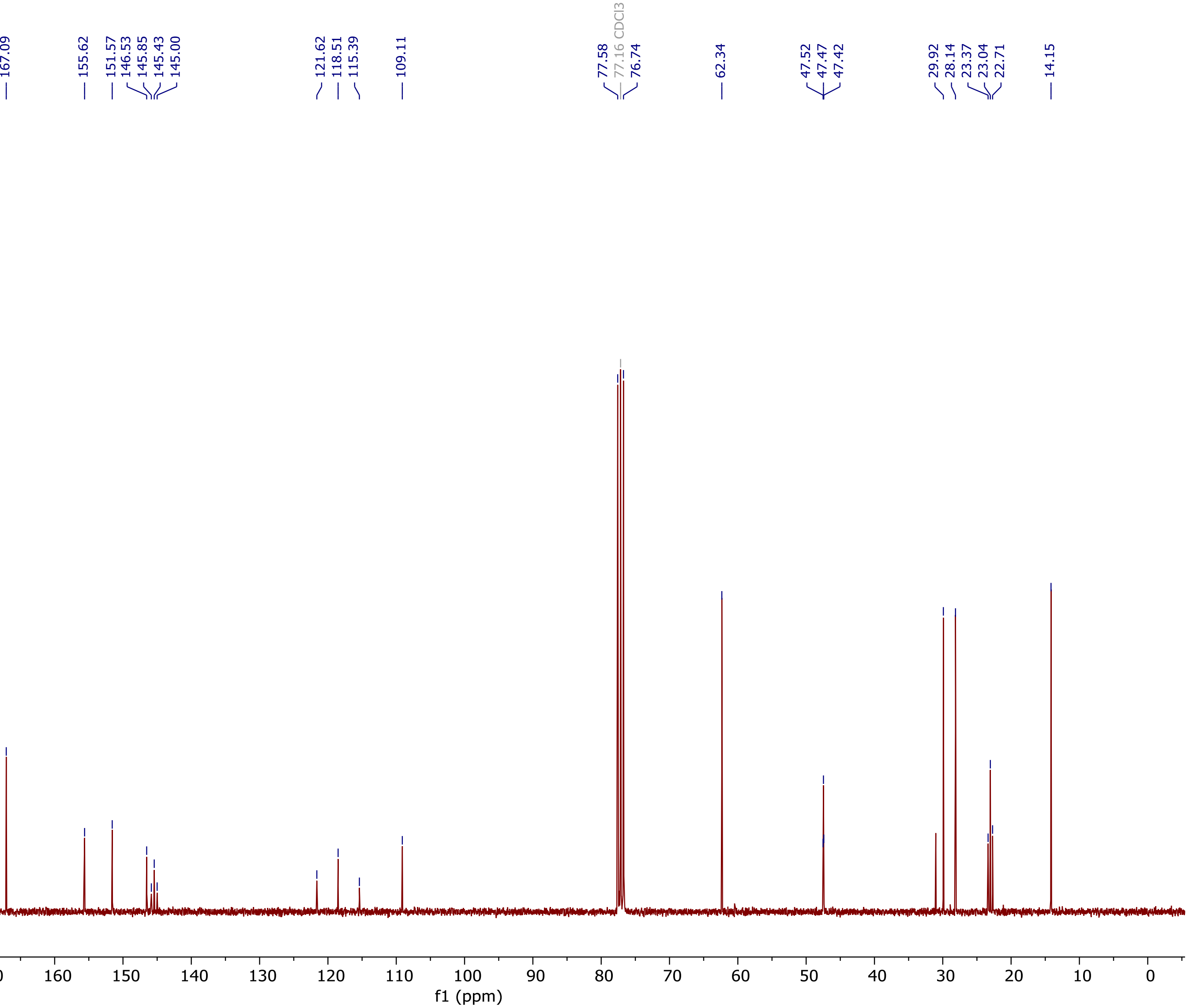

$^{19}F$ NMR (282 MHz, $CDCl_3$)

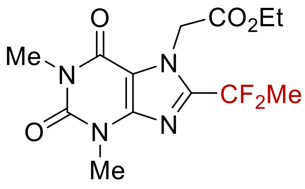


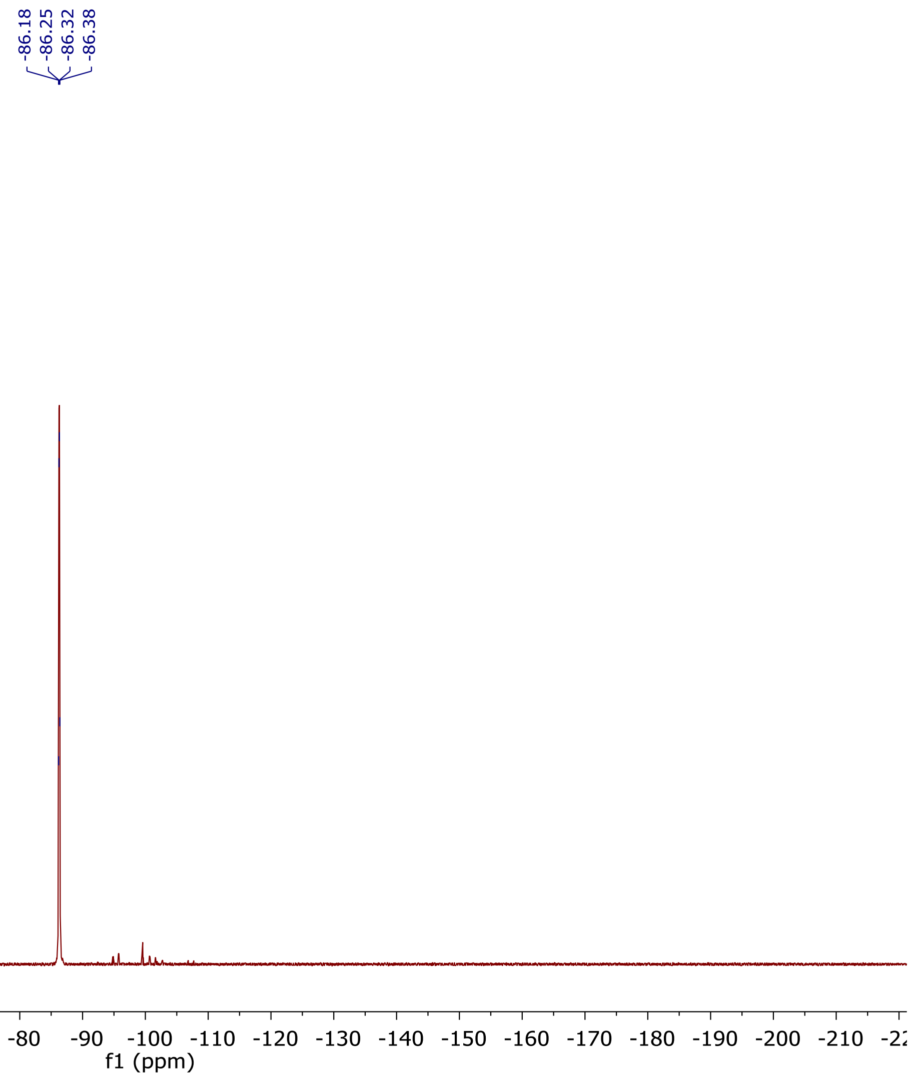

**8-(1,1-Difluoroethyl)-3,7-dimethyl-1-(5-oxohexyl)-3,7-dihydro-1*H*-purine-2,6-dione 29**

$^1$H NMR (300 MHz, $CDCl_3$)

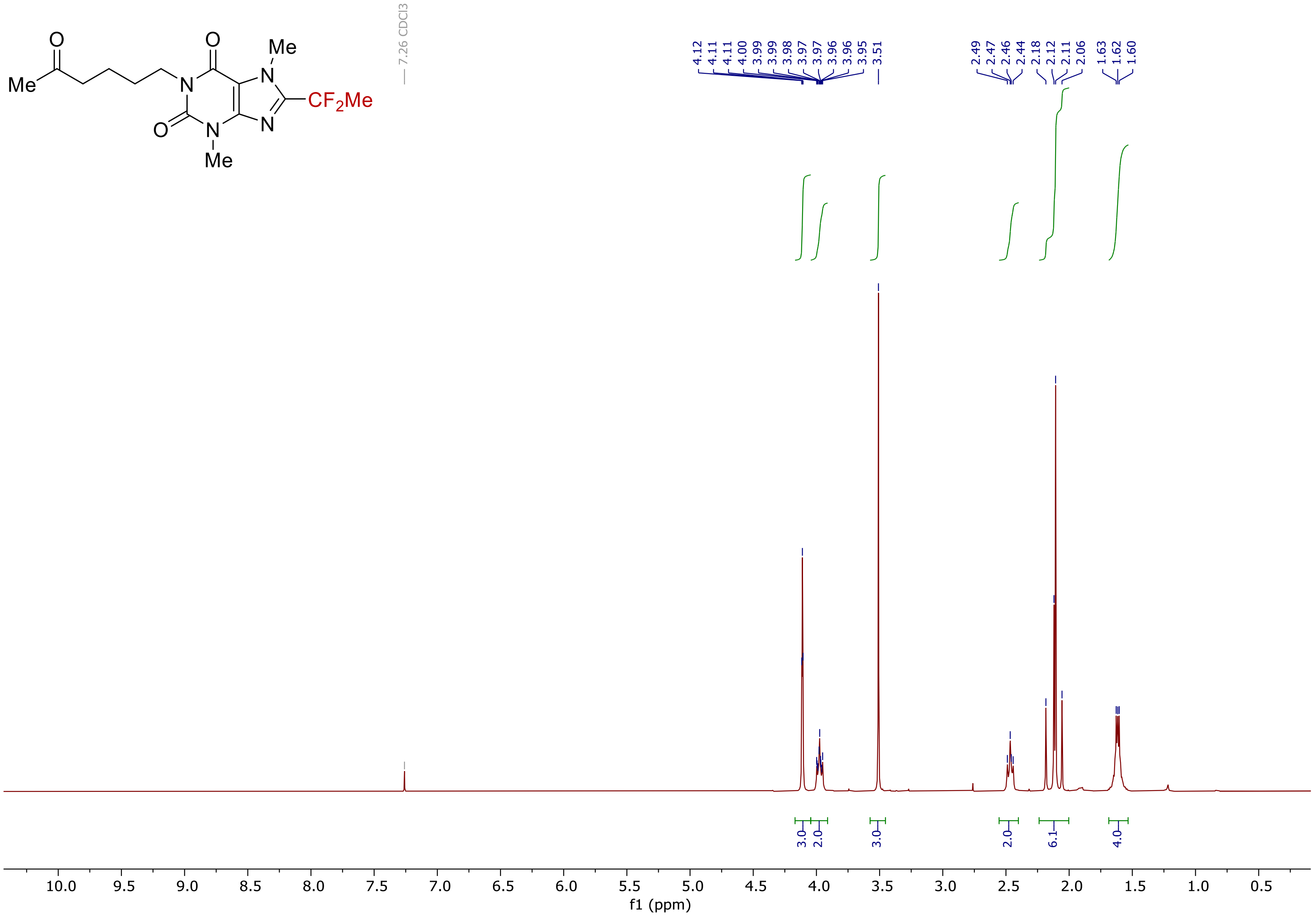

$^{13}C$ NMR (75 MHz, $CDCl_3$)

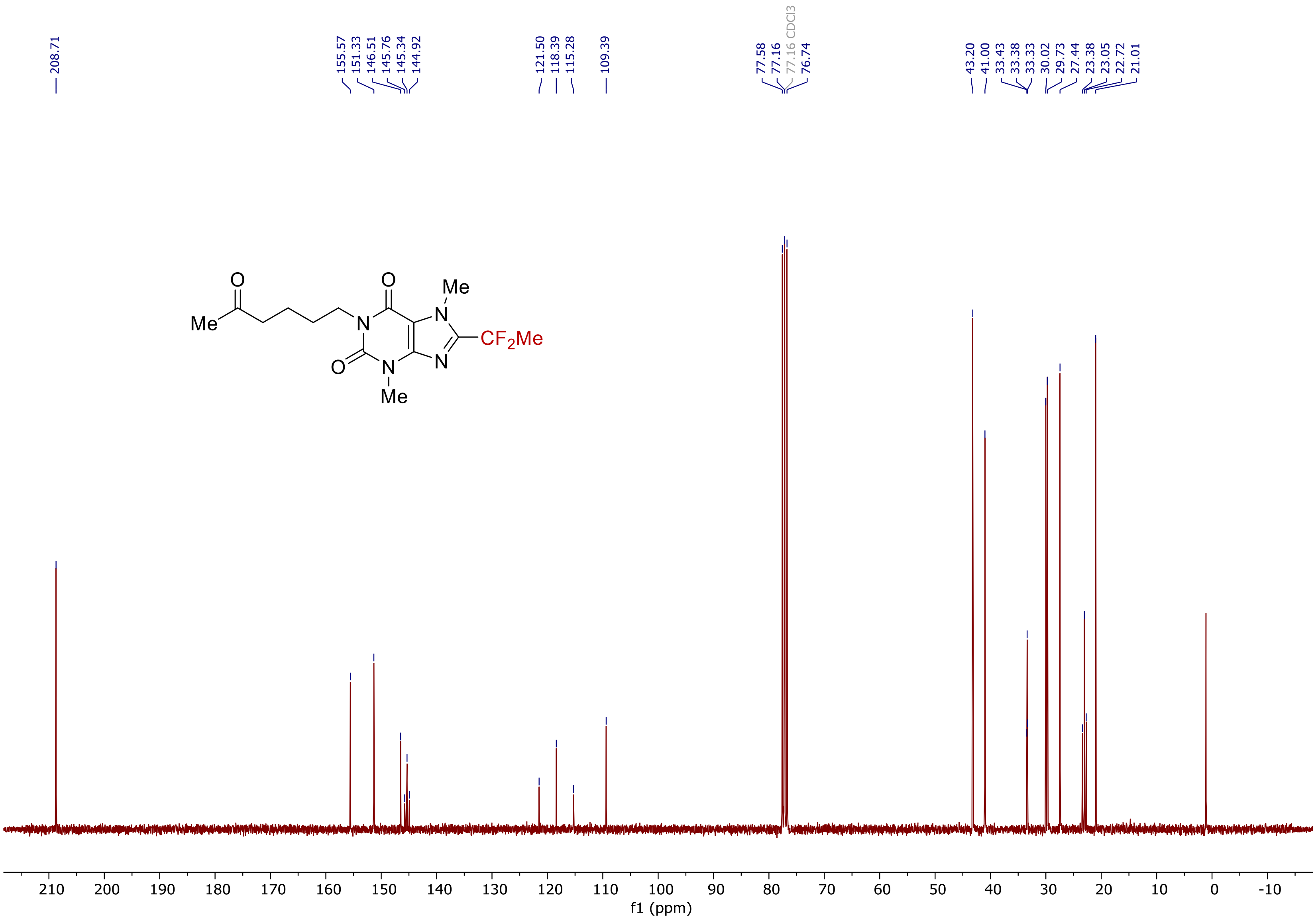

$^{19}F$ NMR (282 MHz, $CDCl_3$)

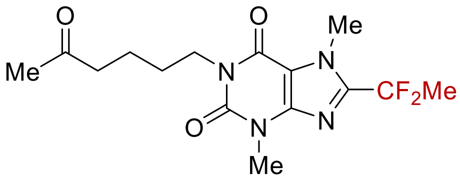


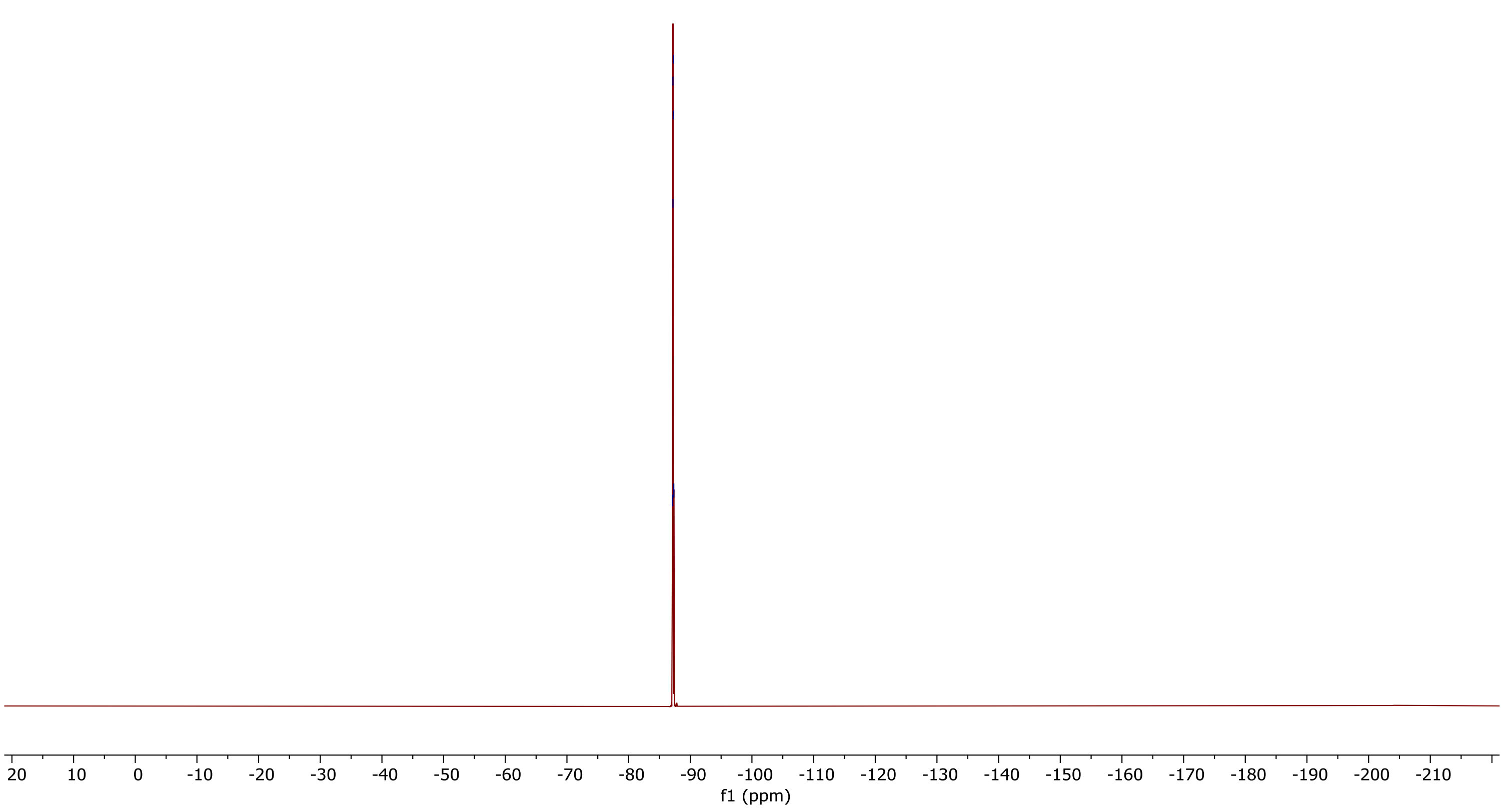

**4-(1,1,2,2-Tetrafluoroethyl)pyridazin-3(2*H*)-one 30**

$^{1}$H NMR (300 MHz, MeOD-d$_4$)

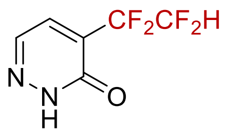


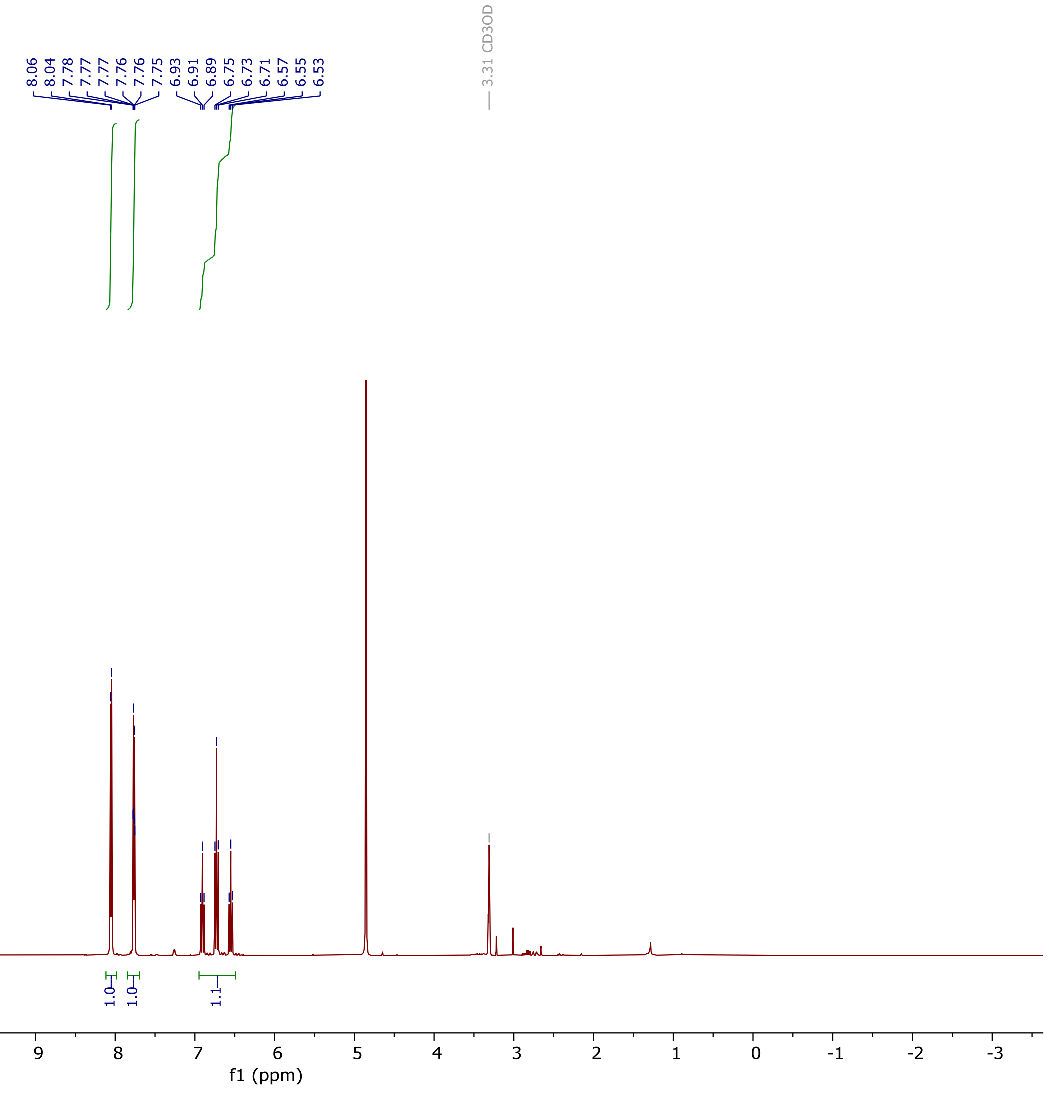

$^{13}C$ NMR (75 MHz, MeOD-d$_4$)

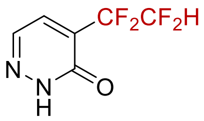


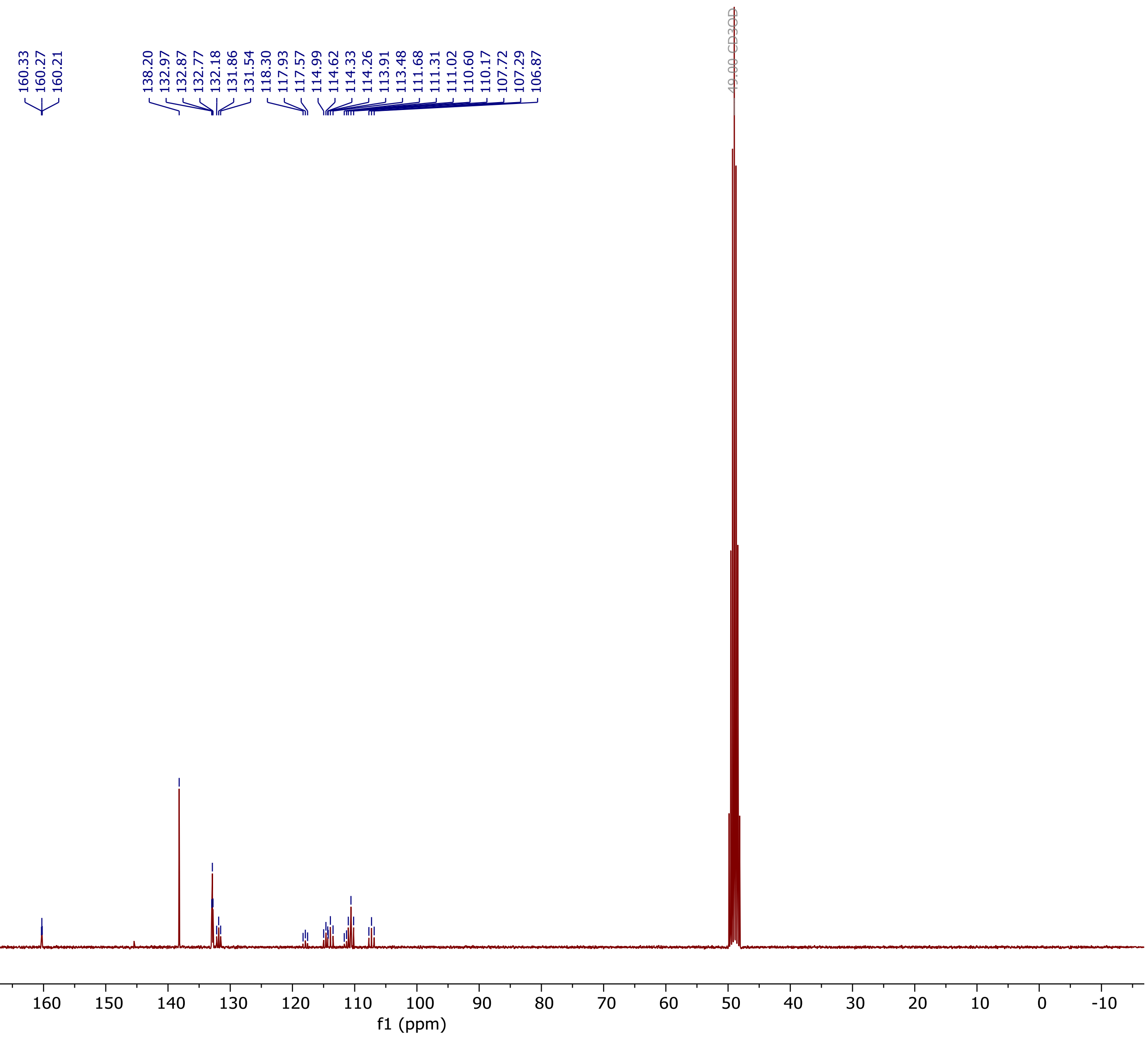

$^{19}$F NMR (282 MHz, MeOD-d$_4$)

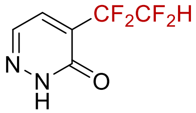


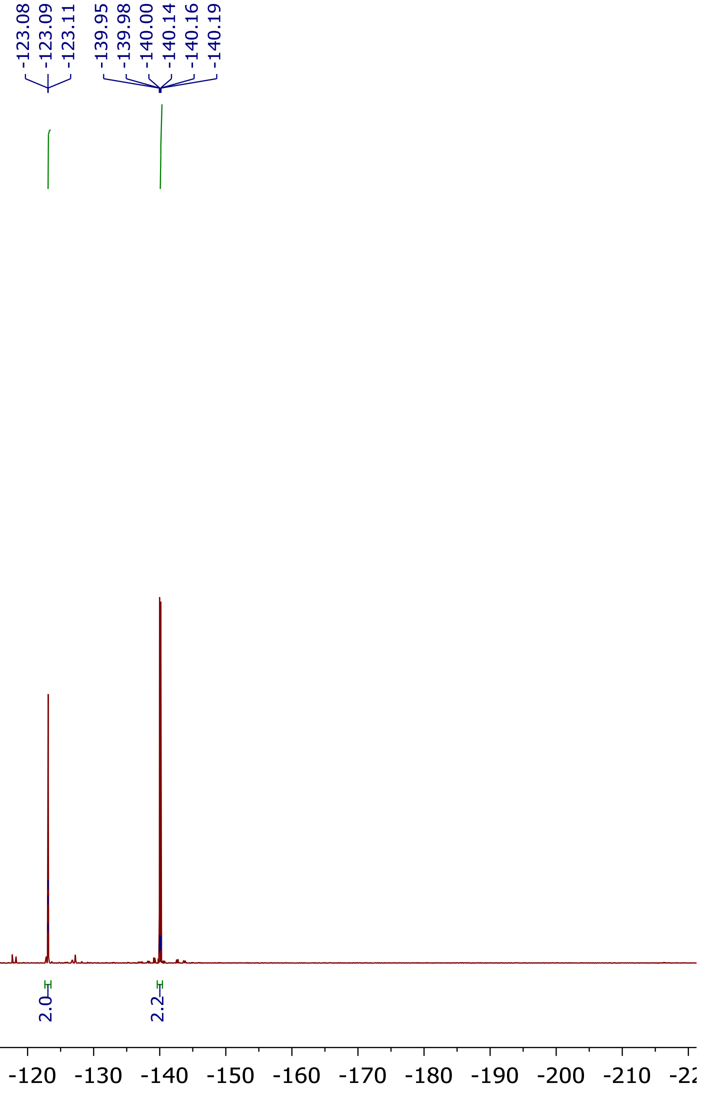

**6-(1,1,2,2-Tetrafluoroethyl)-1,2,4-triazine-3,5(2H,4H)-dione 31**

$^{1}H$ NMR (300 MHz, $CD_3CN$)

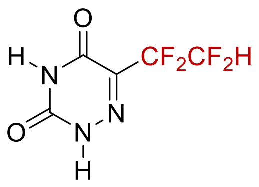


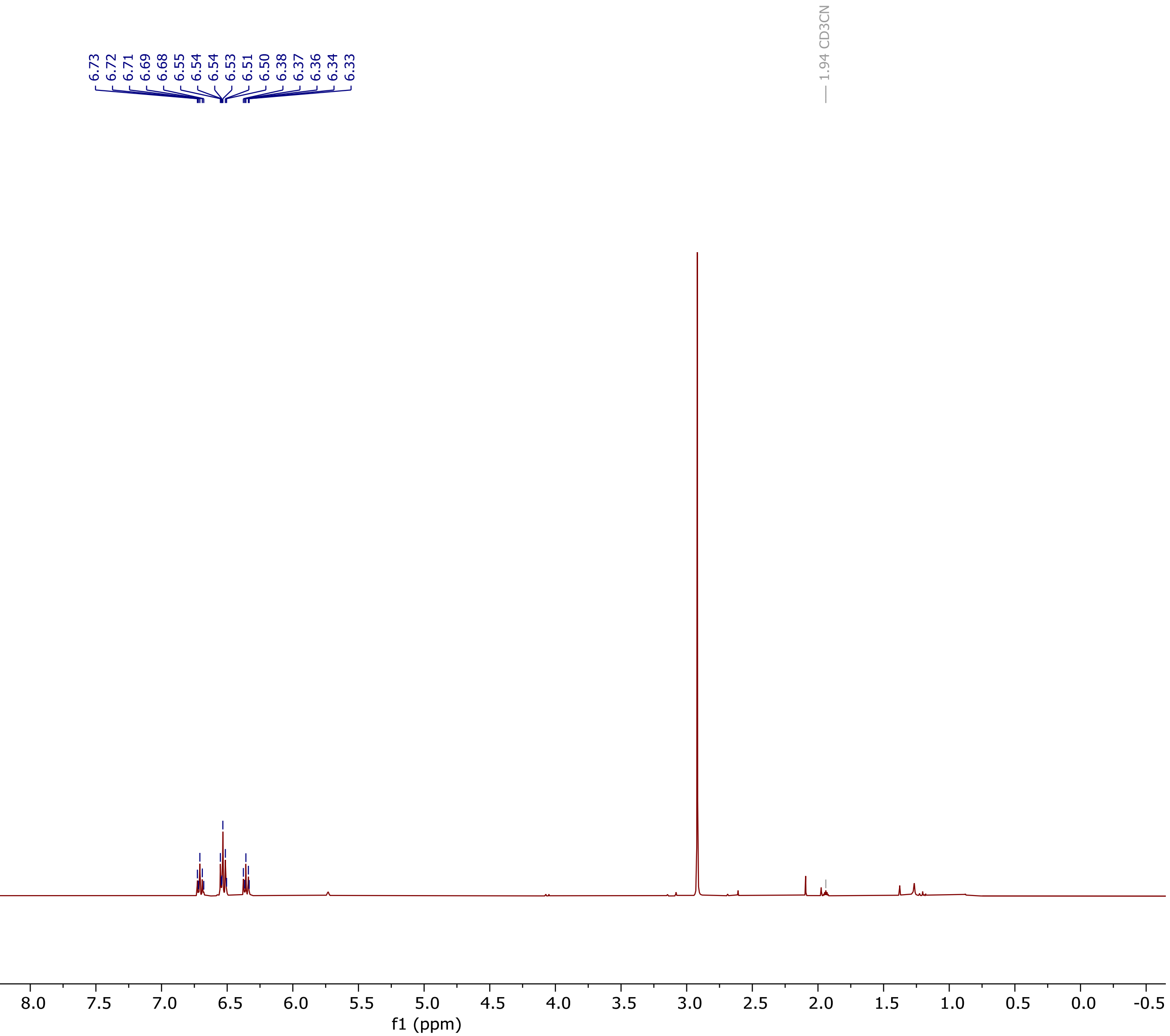

$^{13}C$ NMR (75 MHz, $CD_3CN$)

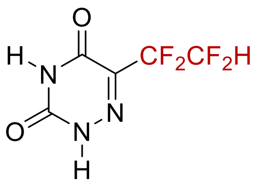

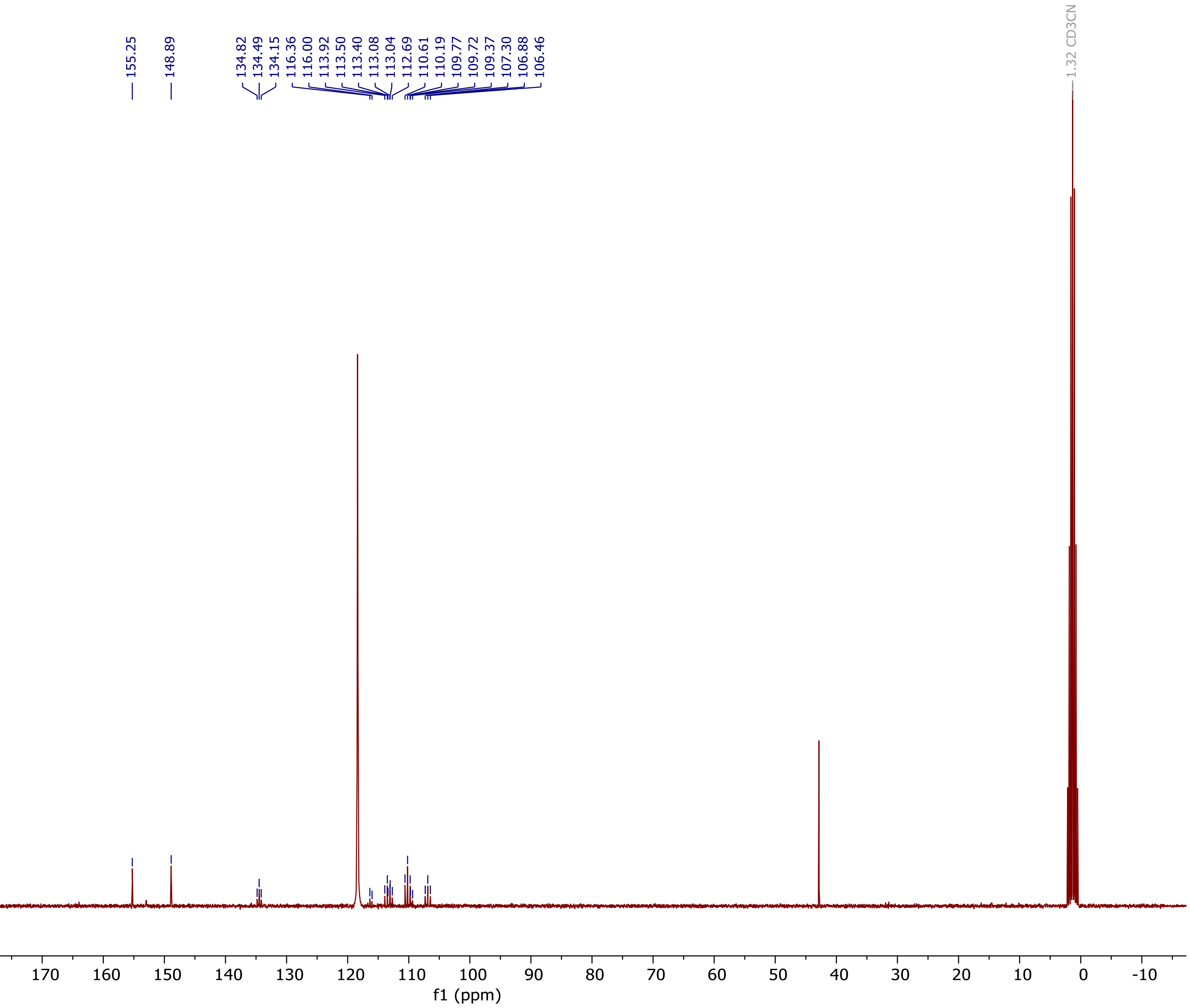

$^{19}F$ NMR (282 MHz, $CD_3CN$)

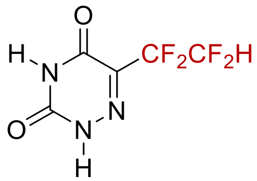


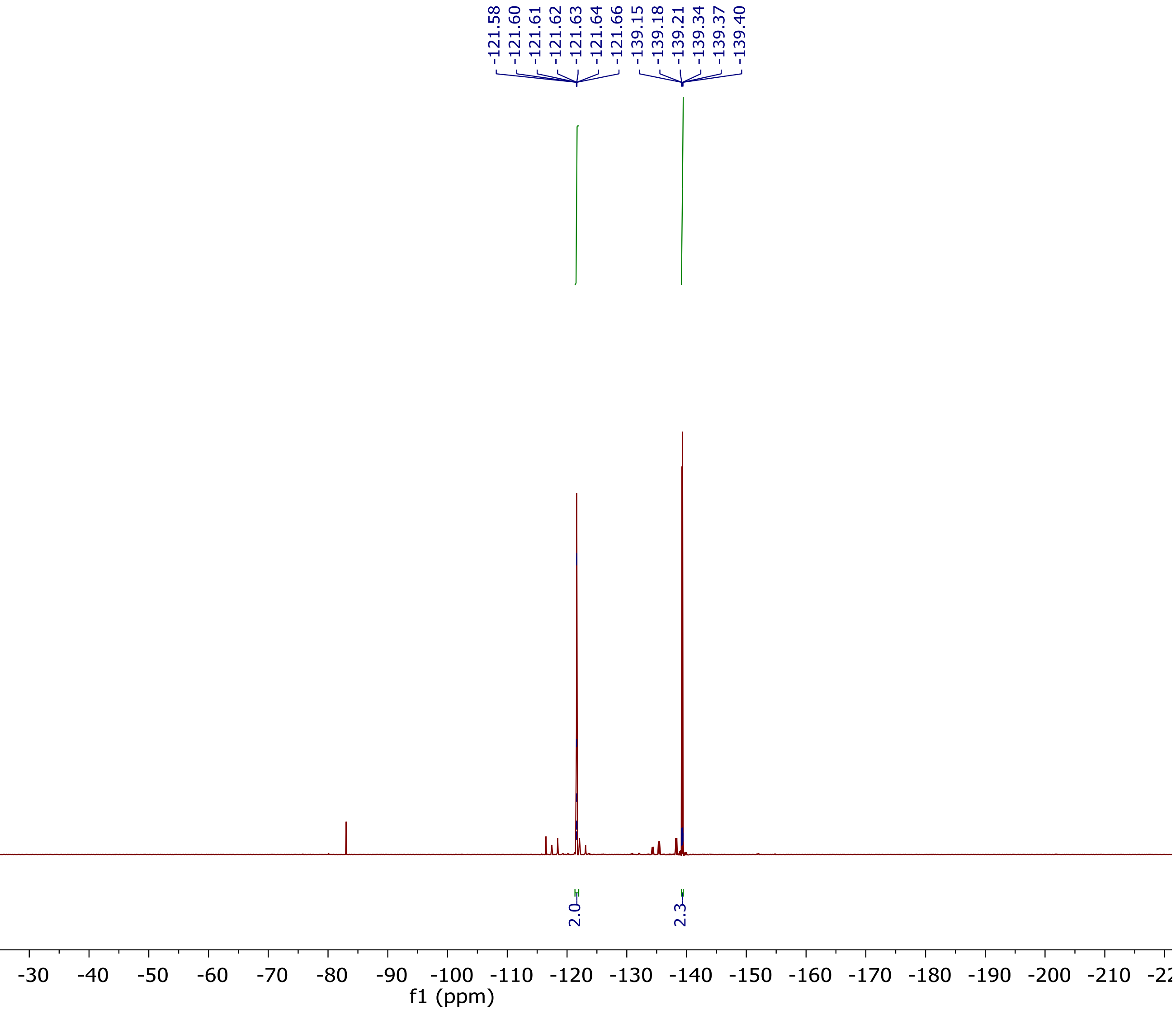

**5-(1,1,2,2-Tetrafluoroethyl)pyrimidine-2,4(1*H*,3*H*)-dione 32**

$^{1}$H NMR (300 MHz, $CD_3CN$)

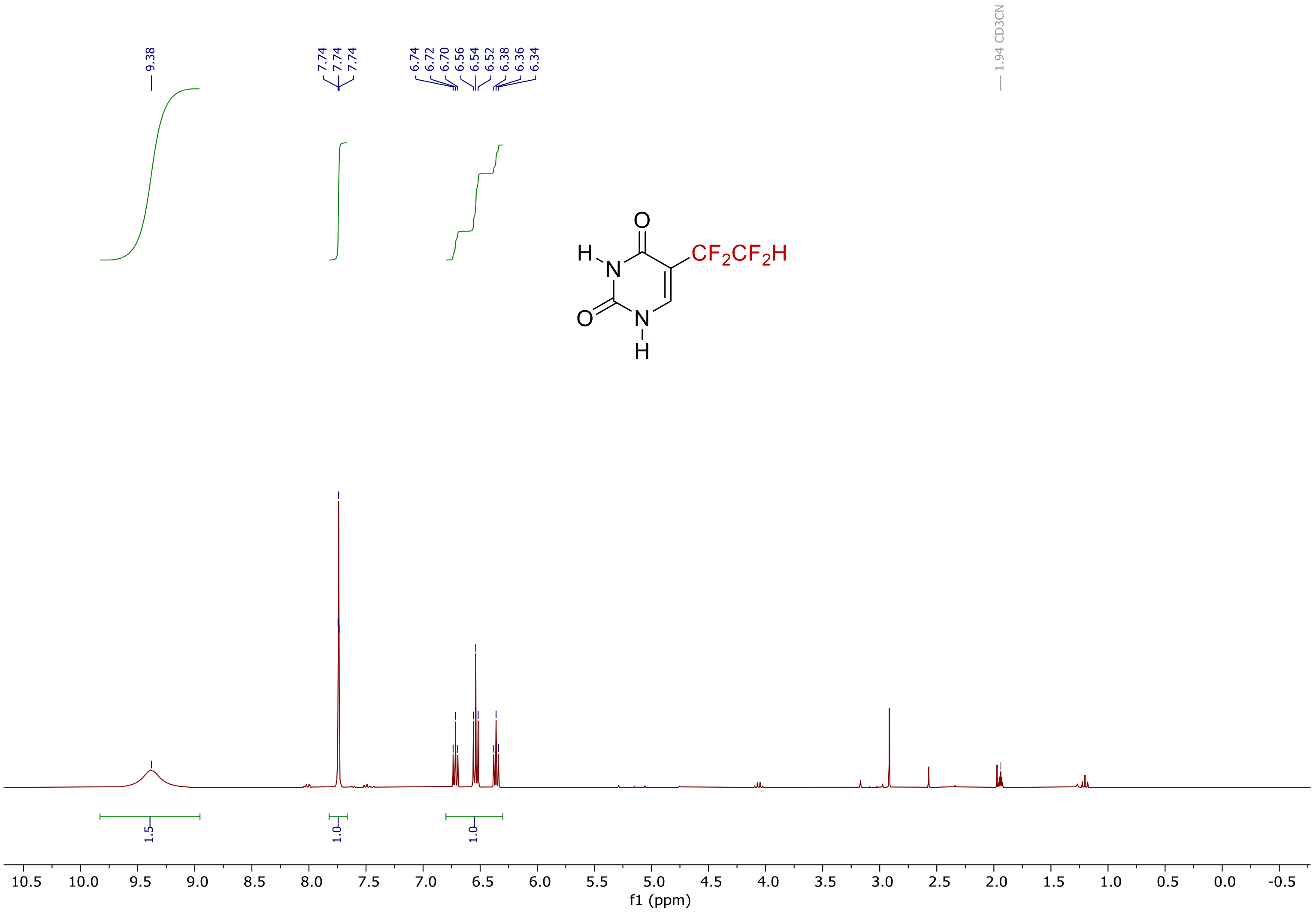

$^{13}C$ NMR (75 MHz, $CD_3CN$)

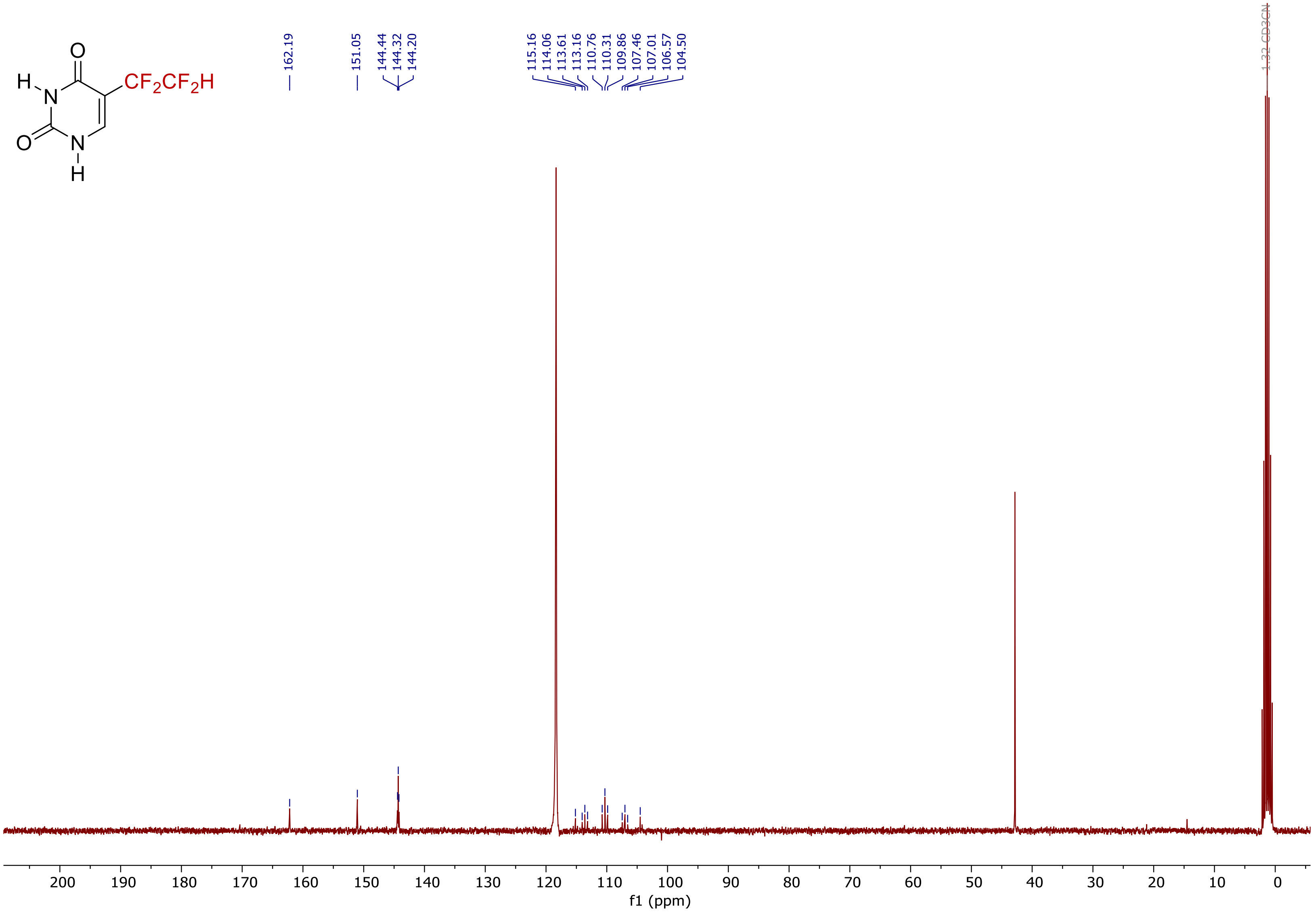

$^{19}$F NMR (282 MHz, $CD_3CN$)

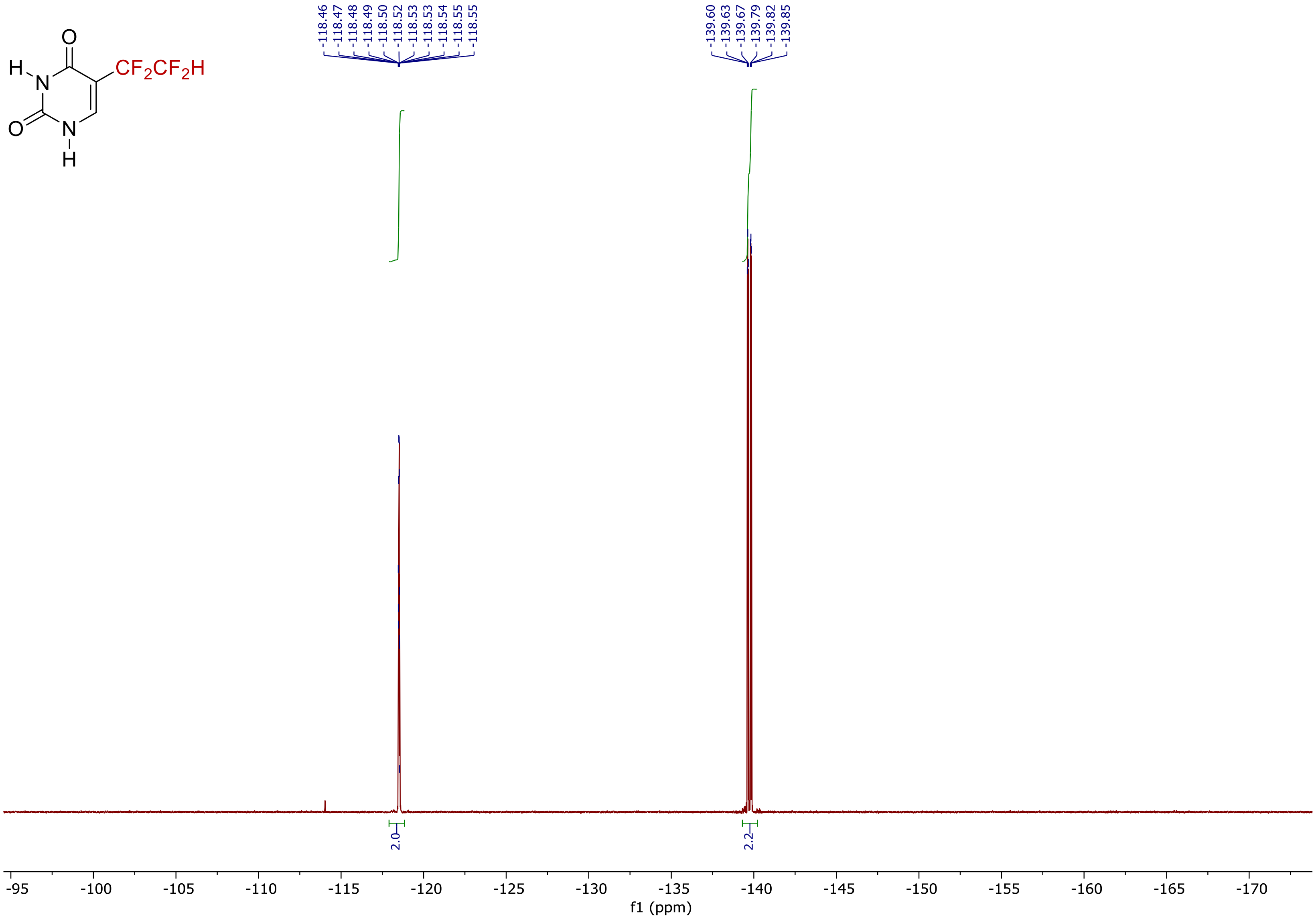

**6-Methyl-5-(1,1,2,2-tetrafluoroethyl)pyrimidine-2,4(1*H*,3*H*)-dione 33**

$^{1}$H NMR (300 MHz, MeOD-d$_4$)

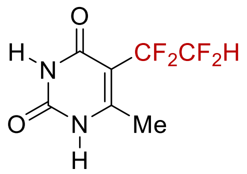


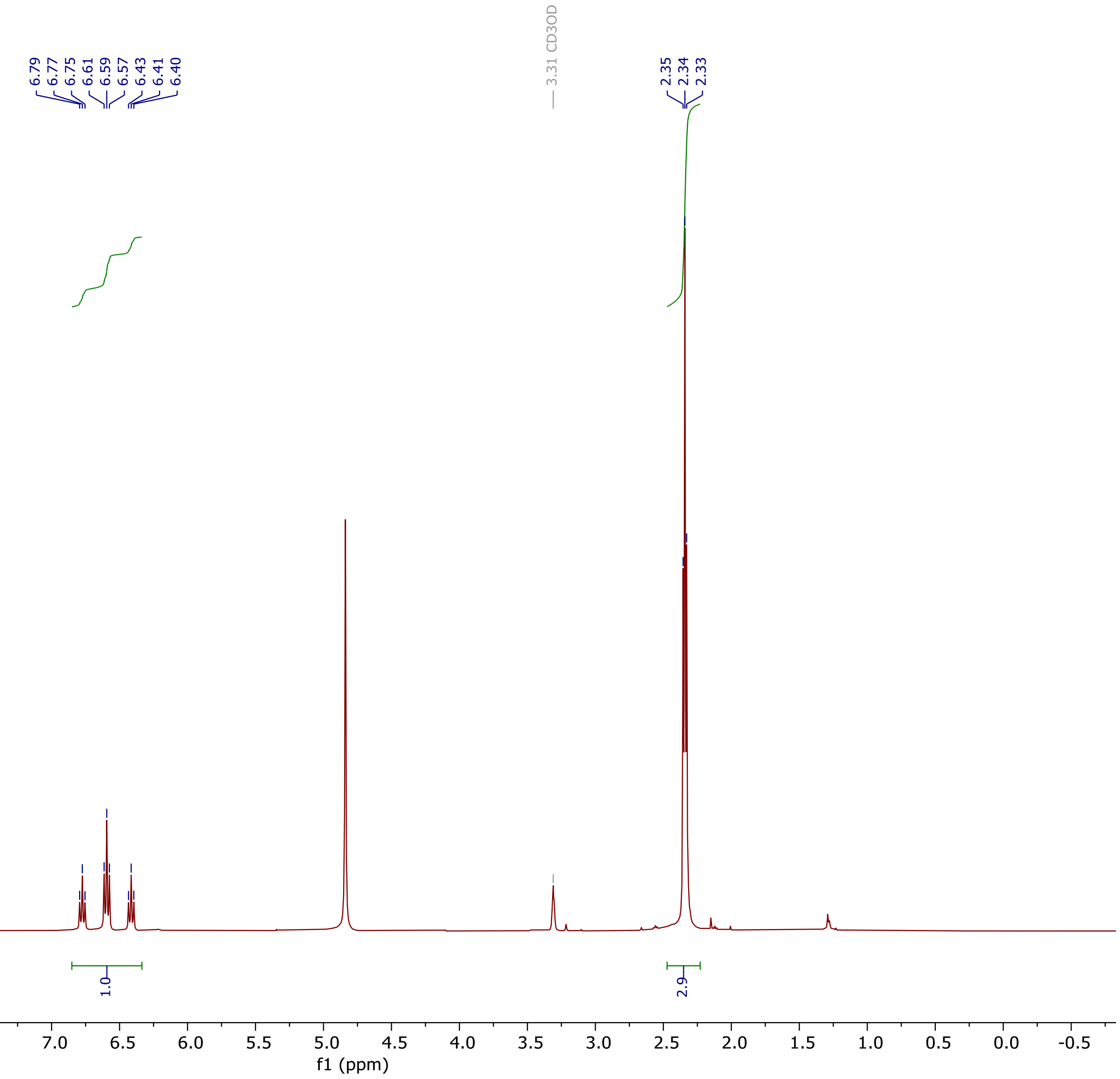

$^{13}C$ NMR (75 MHz, MeOD-d$_4$)

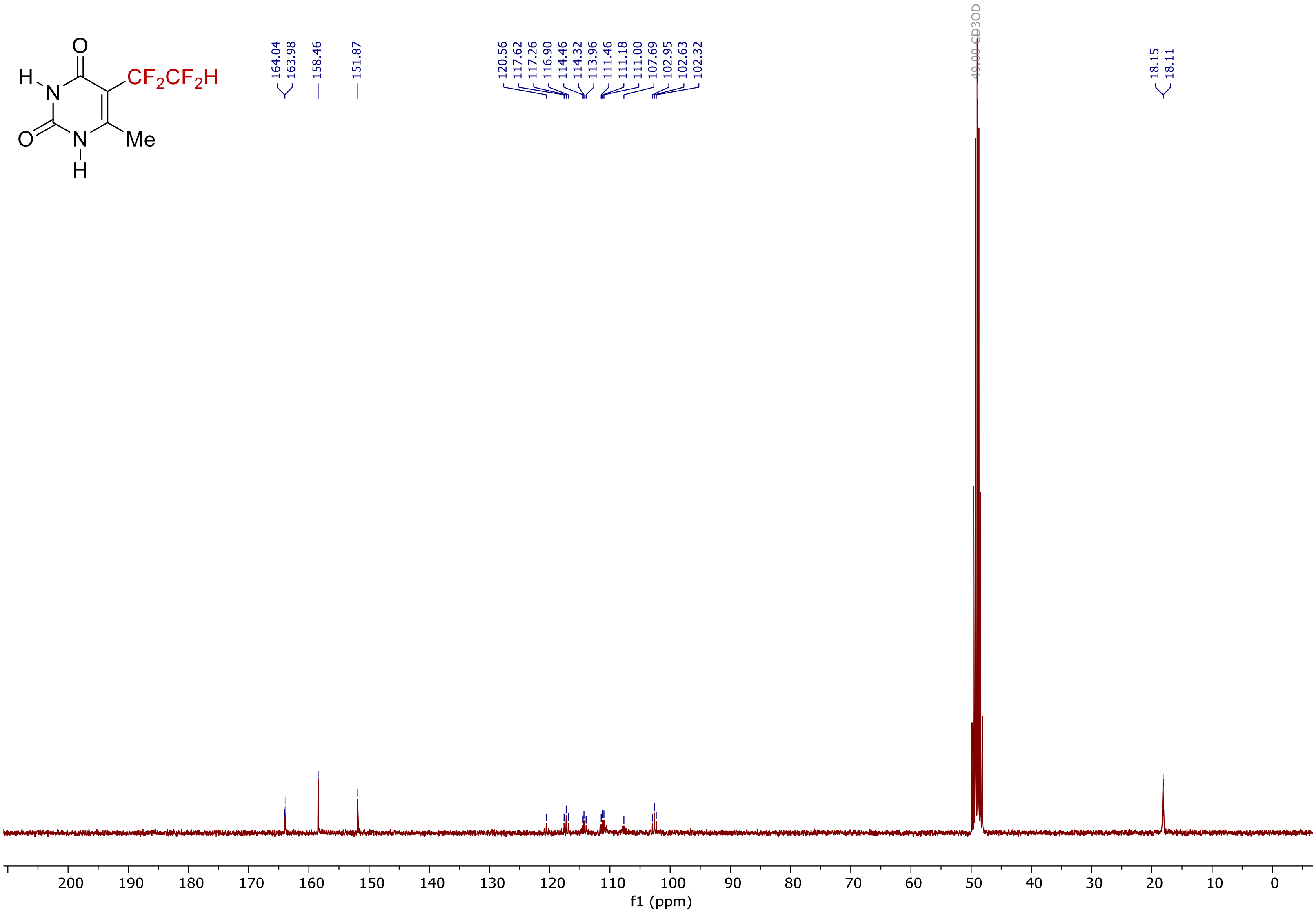

$^{19}F$ NMR (282 MHz, MeOH-$d_4$)

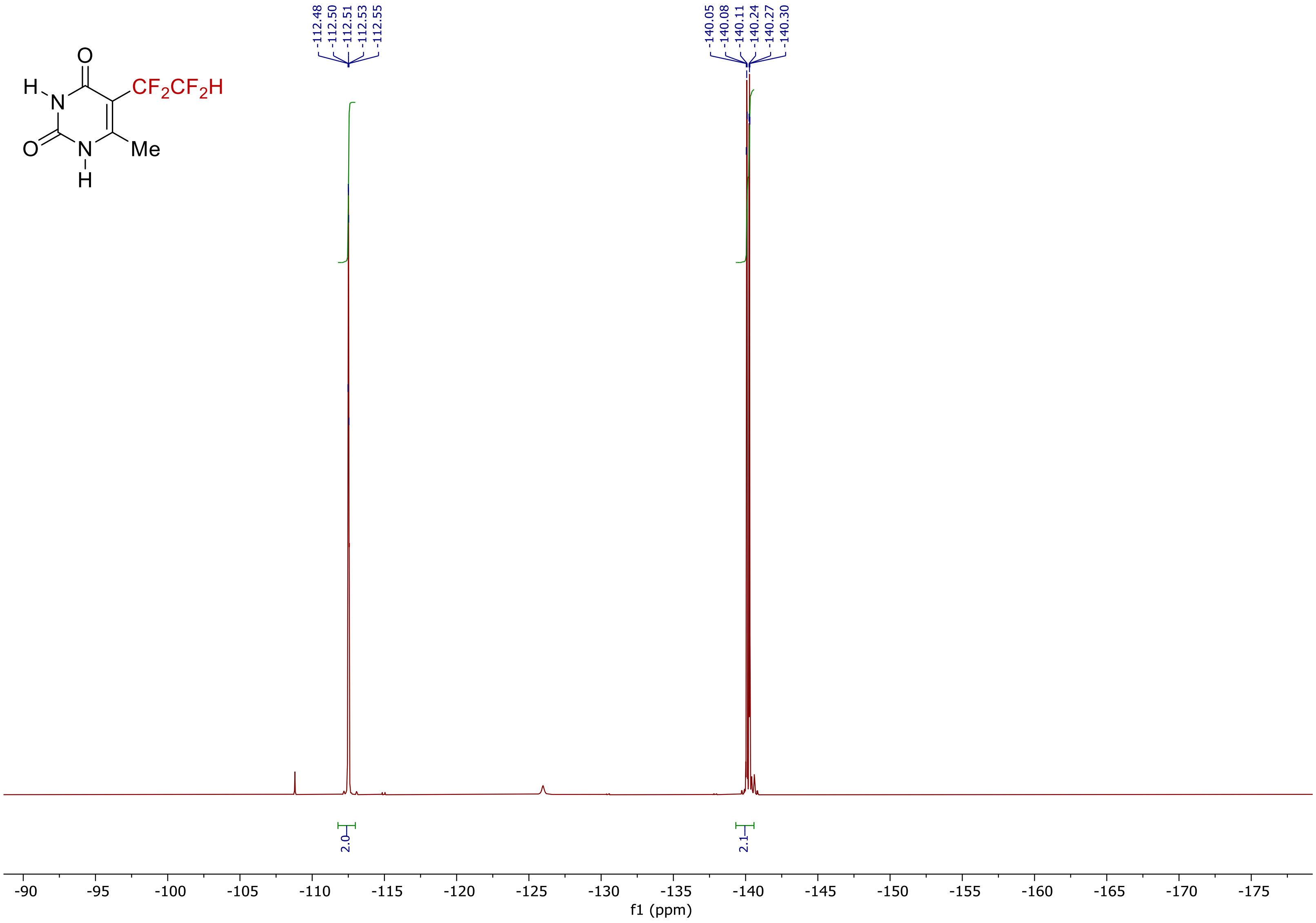

**3-(1,1,2,2-Tetrafluoroethyl)-1,5-dihydro-4*H*-pyrazolo[3,4-d]pyrimidin-4-one 34**

$^{1}$H NMR (400 MHz, MeOD-d$_4$)

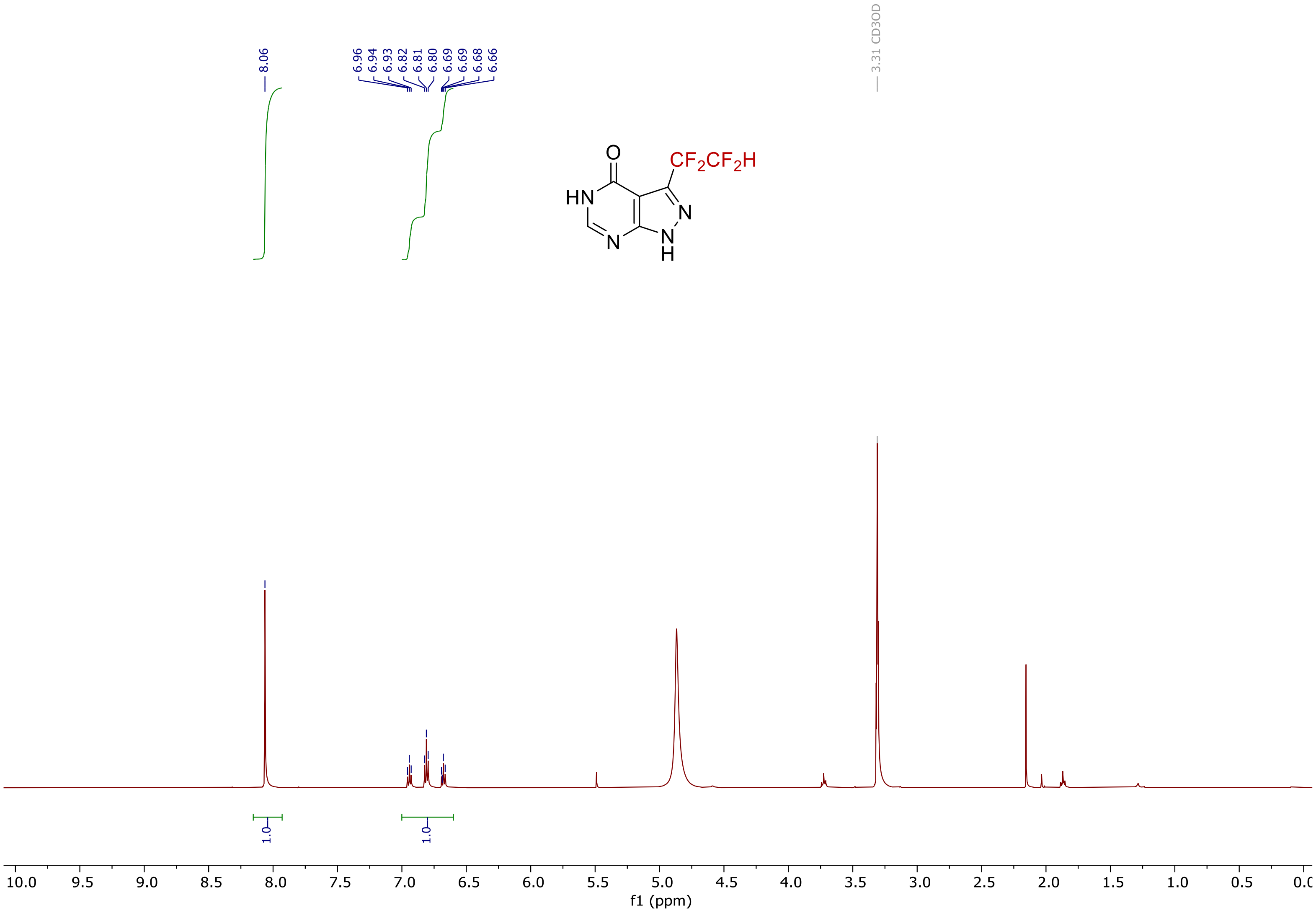

<sup></sup>

$^{13}$C NMR (101 MHz, MeOD-d$_4$)

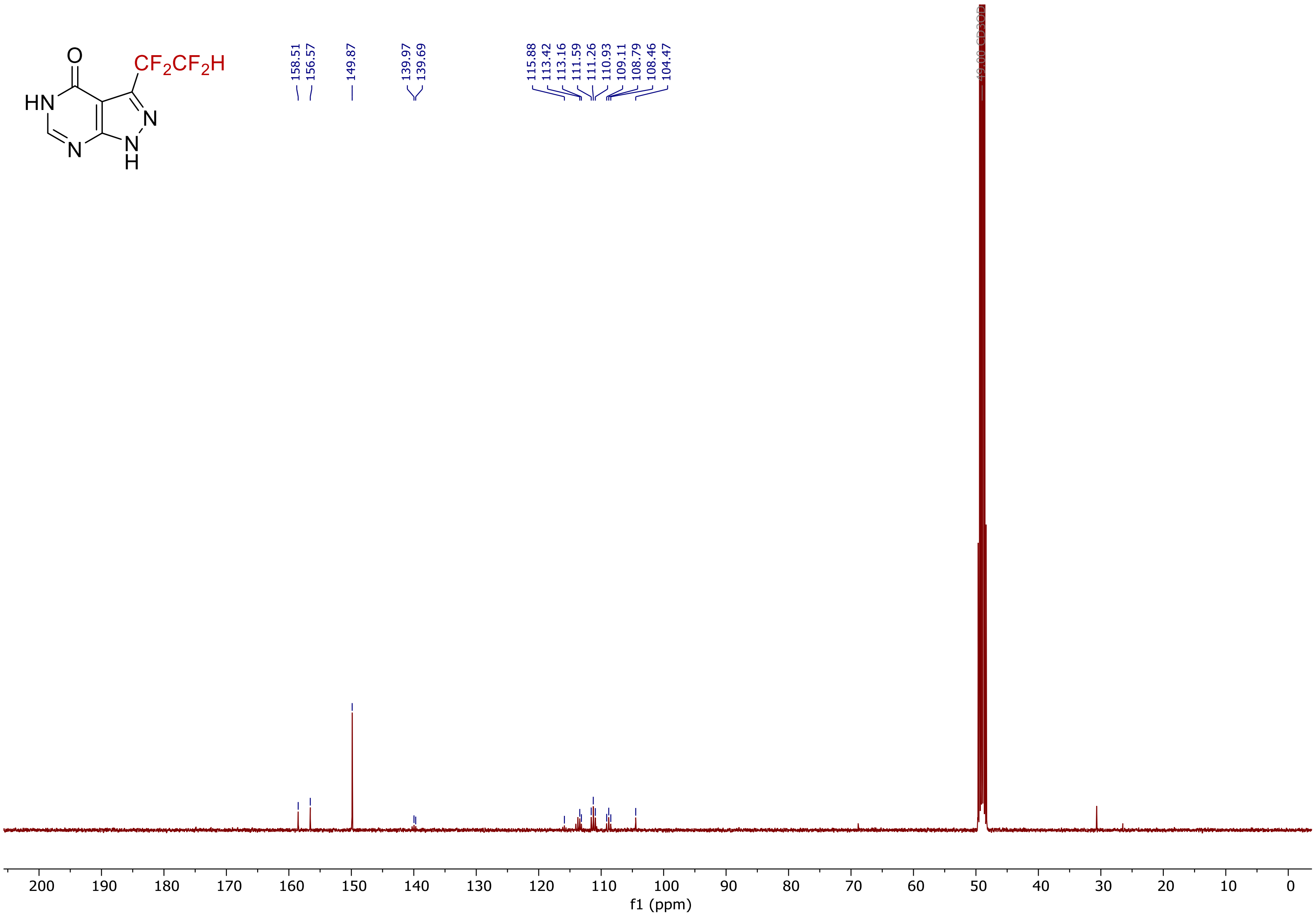

$^{19}$F NMR (377 MHz, MeOD-d$_4$)

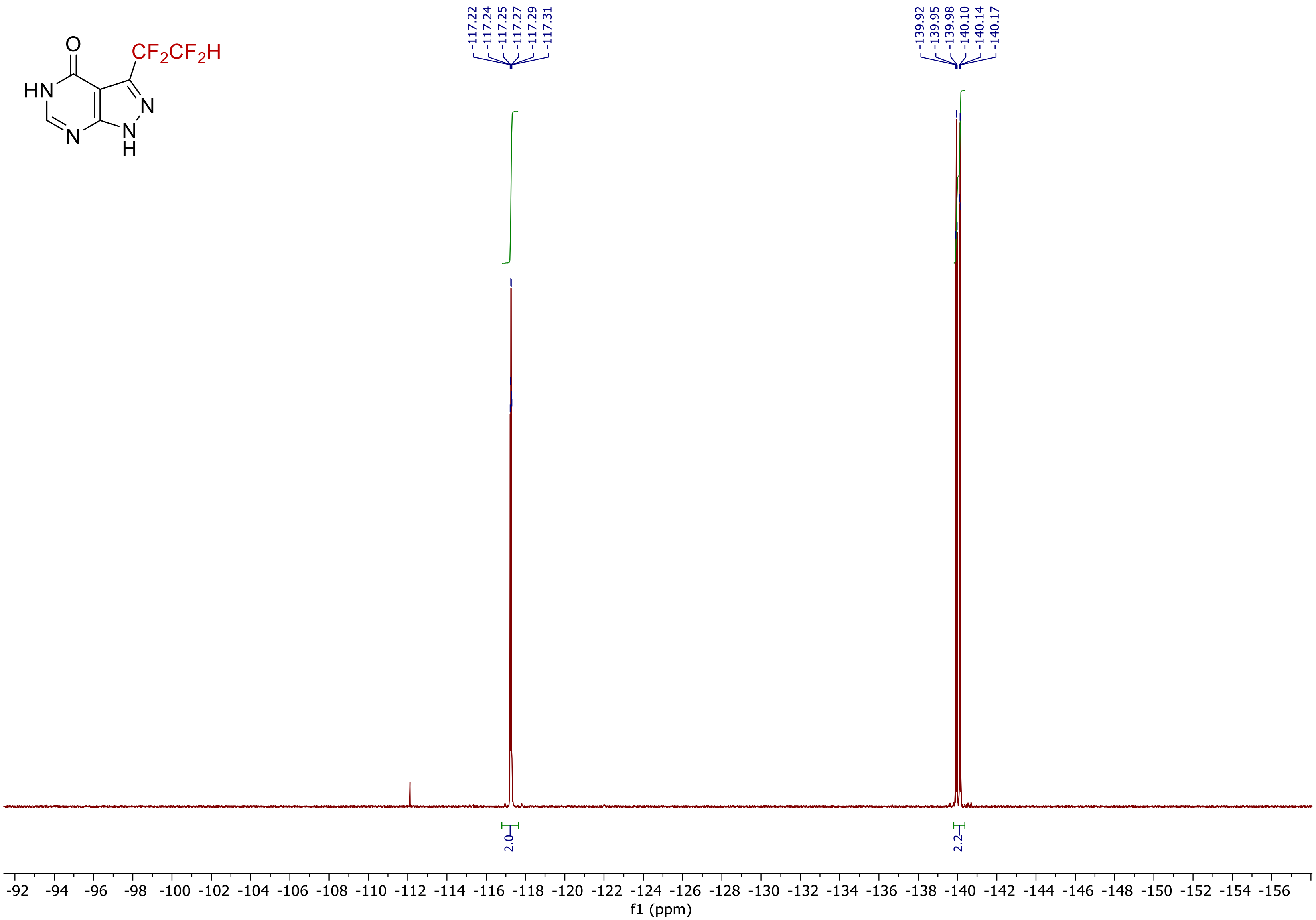

**6-Chloro-8-(difluoro(phenyl)methyl)-4,5-dihydro-9*H*-purin-2-amine 35**

$^1$H NMR (300 MHz, DMSO-d$_6$)

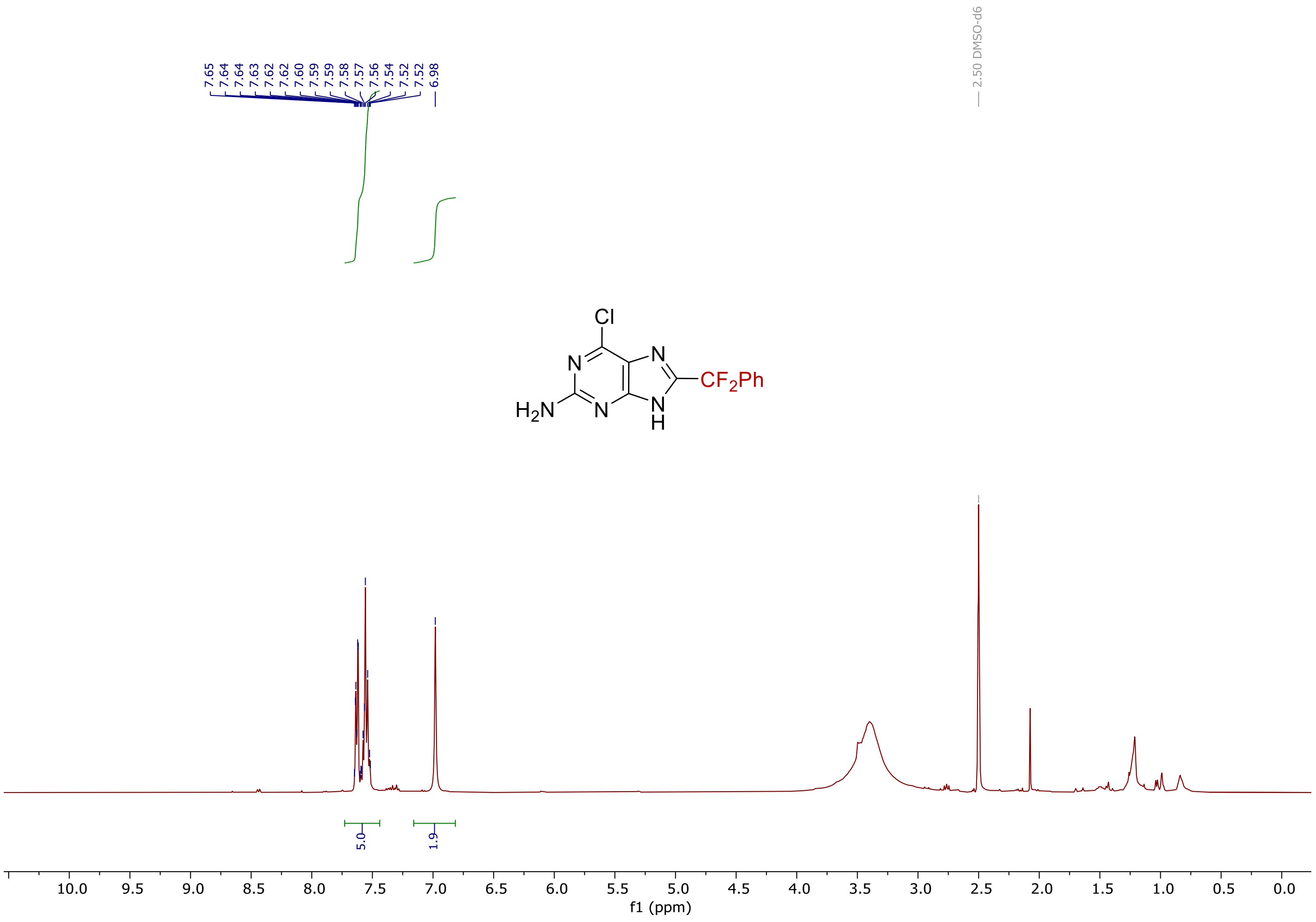

$^{13}$C NMR (75 MHz, DMSO-d$_6$)

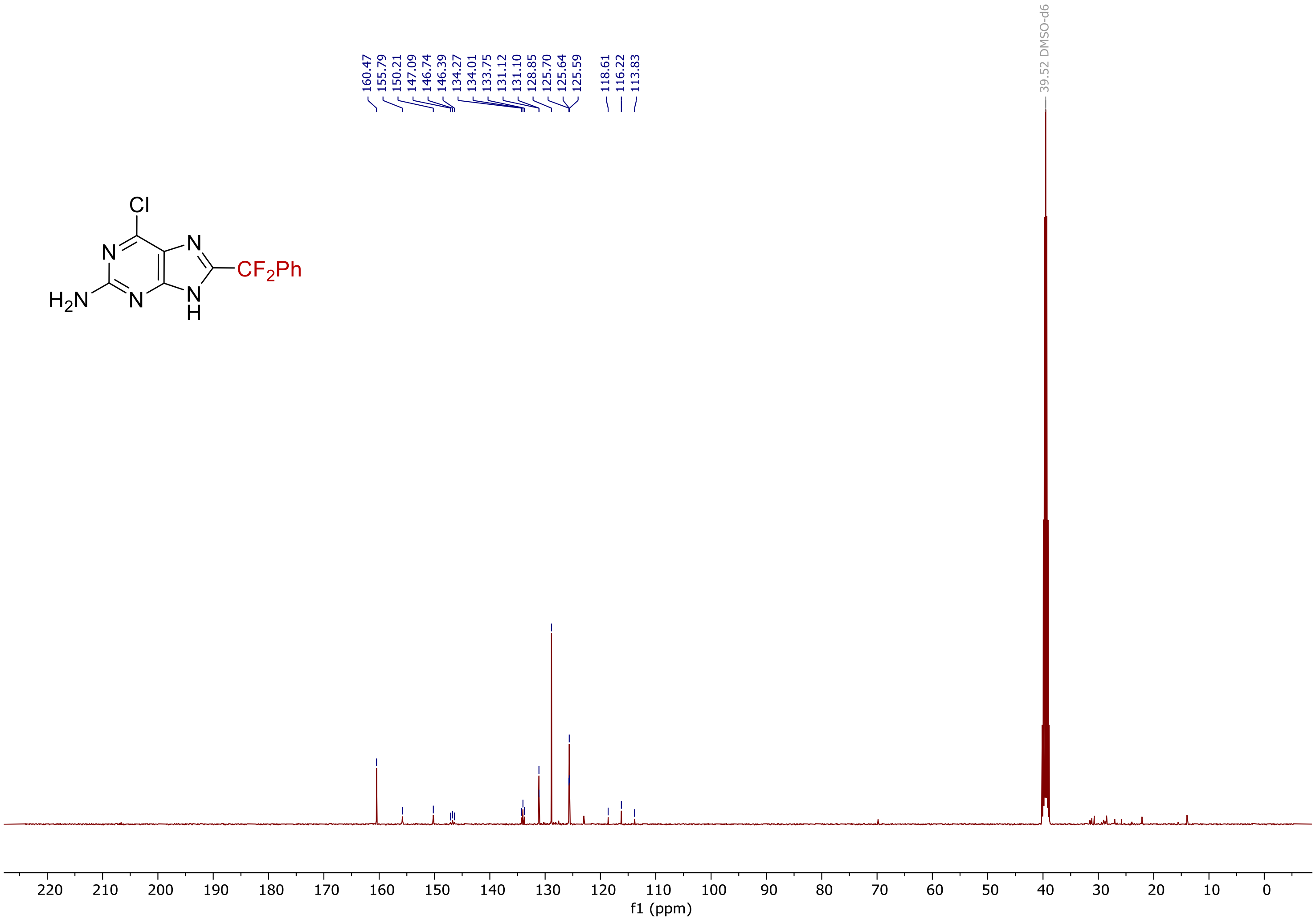

$^{19}F$ NMR (282 MHz, DMSO-d$_6$)

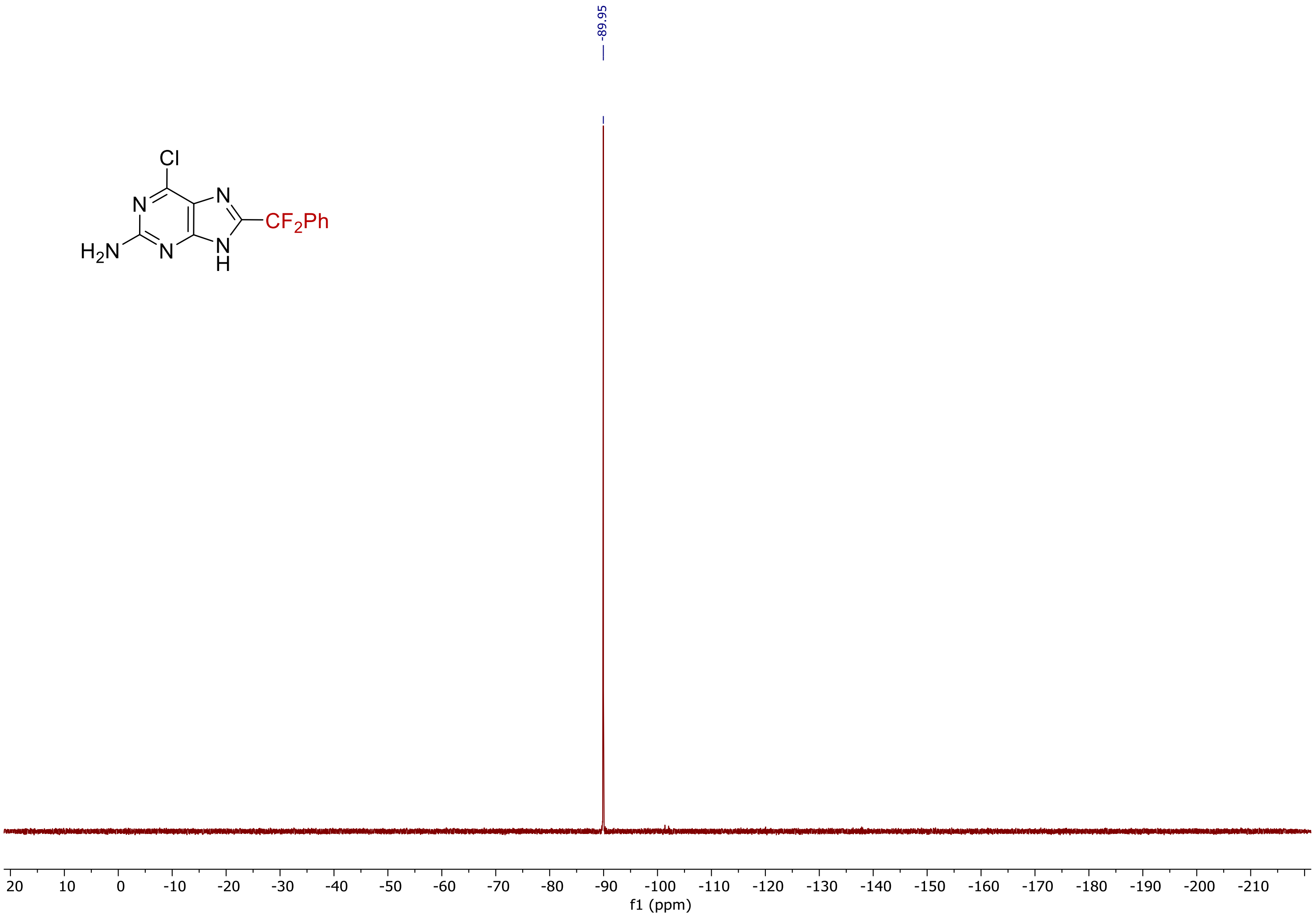

**(2*R*,3*S*,4*S*,5*S*)-2-(Acetoxymethyl)-5-(2-amino-8-(difluoro(phenyl)methyl)-6-oxo-1,4,5,6-tetrahydro-9*H*-purin-9-yl)tetrahydrofuran-3,4-diyl diacetate 36**

$^{1}$H NMR (300 MHz, DMSO-d$_6$)

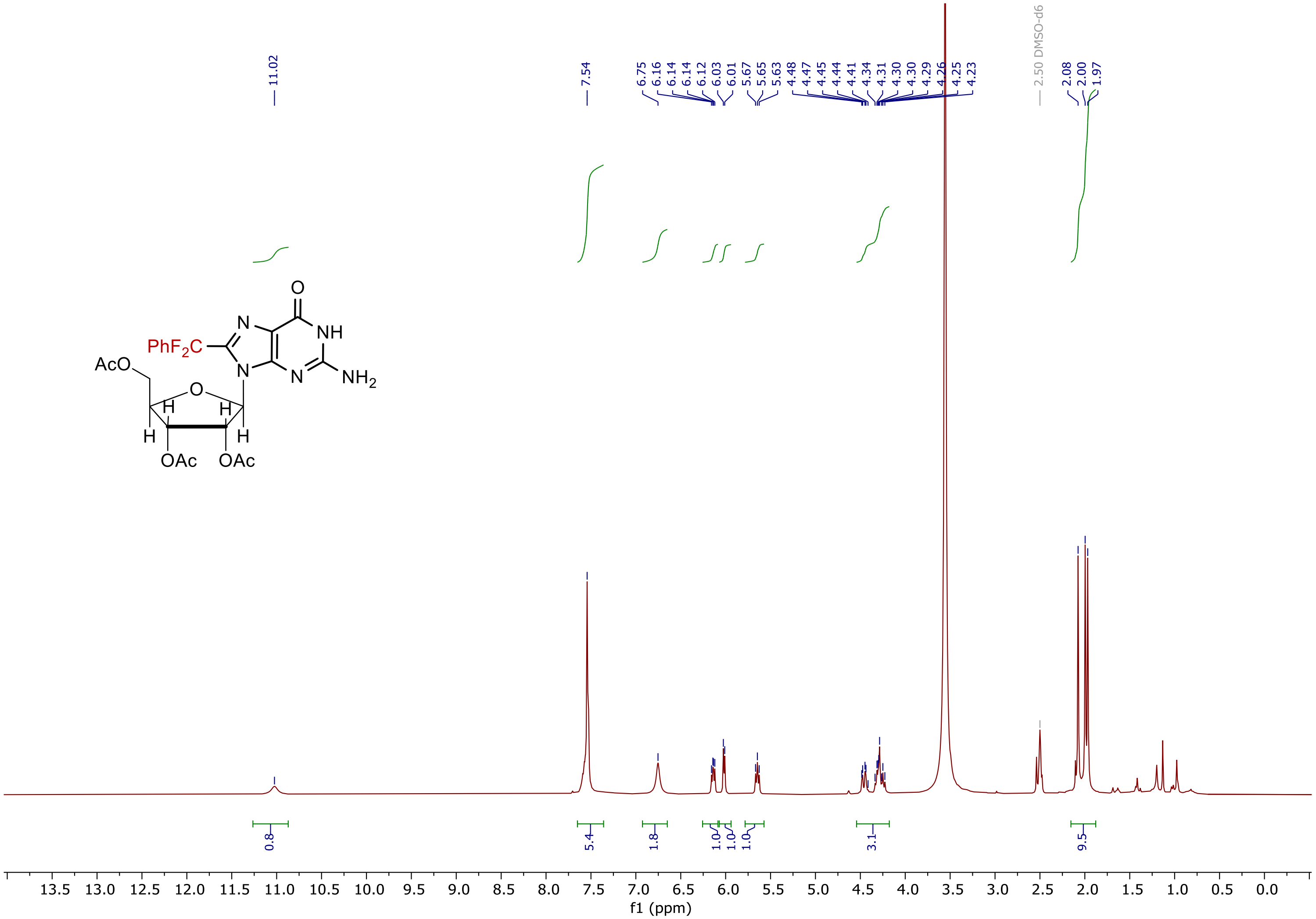

$^{13}C$ NMR (75 MHz, DMSO-$d_6$)

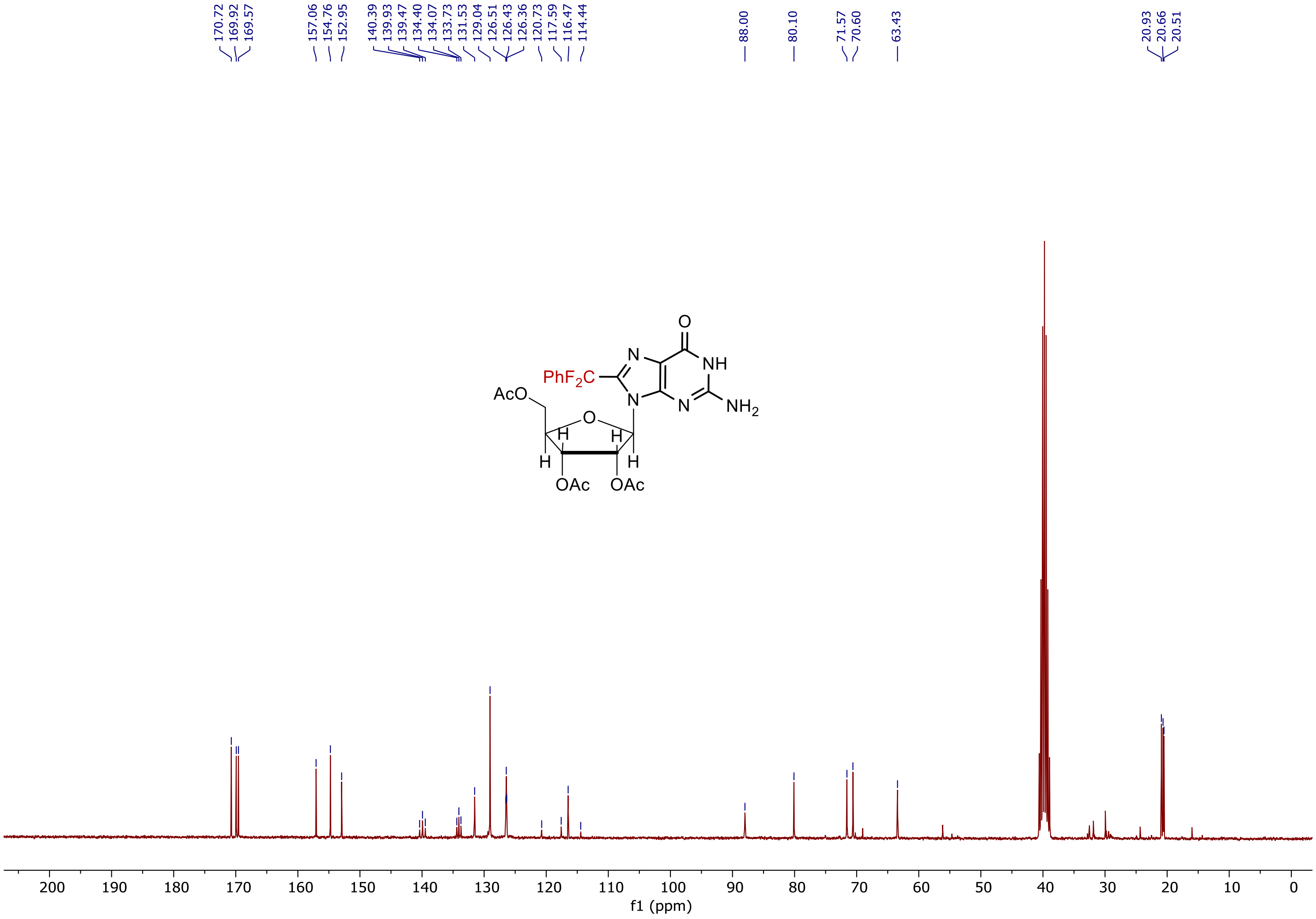

$^{19}F$ NMR (282 MHz, DMSO-$d_6$)

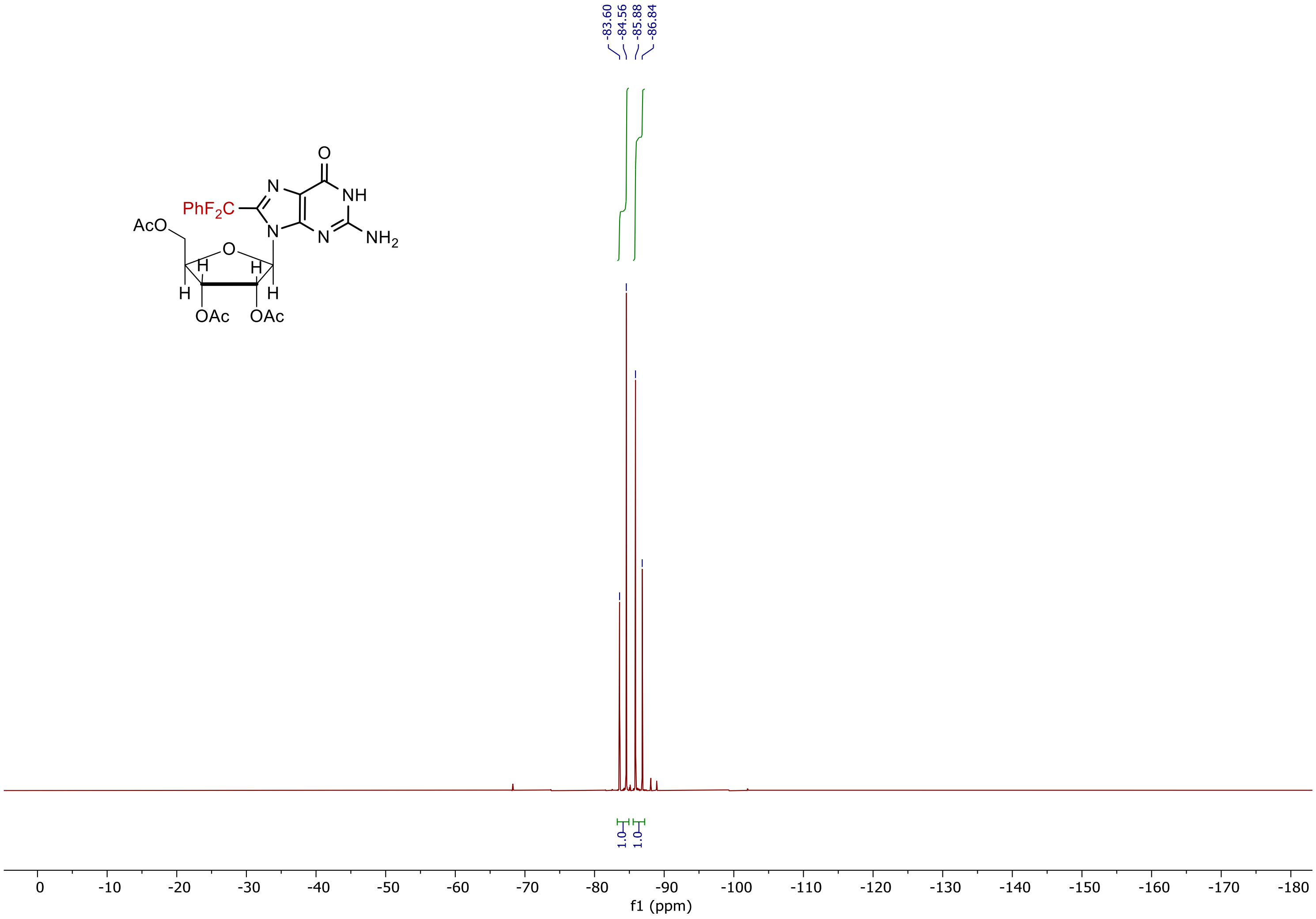

**2-Amino-8-(difluoro(phenyl)methyl)-9-((3a*R*,4*R*,6*R*,6a*R*)-6-(hydroxymethyl)-2,2-dimethyltetrahydrofuro[3,4-*d*][1,3]dioxol-4-yl)-1,4,5,9-tetrahydro-6*H*-purin-6-one 37**

$^1$H NMR (300 MHz, MeOD-d$_4$)

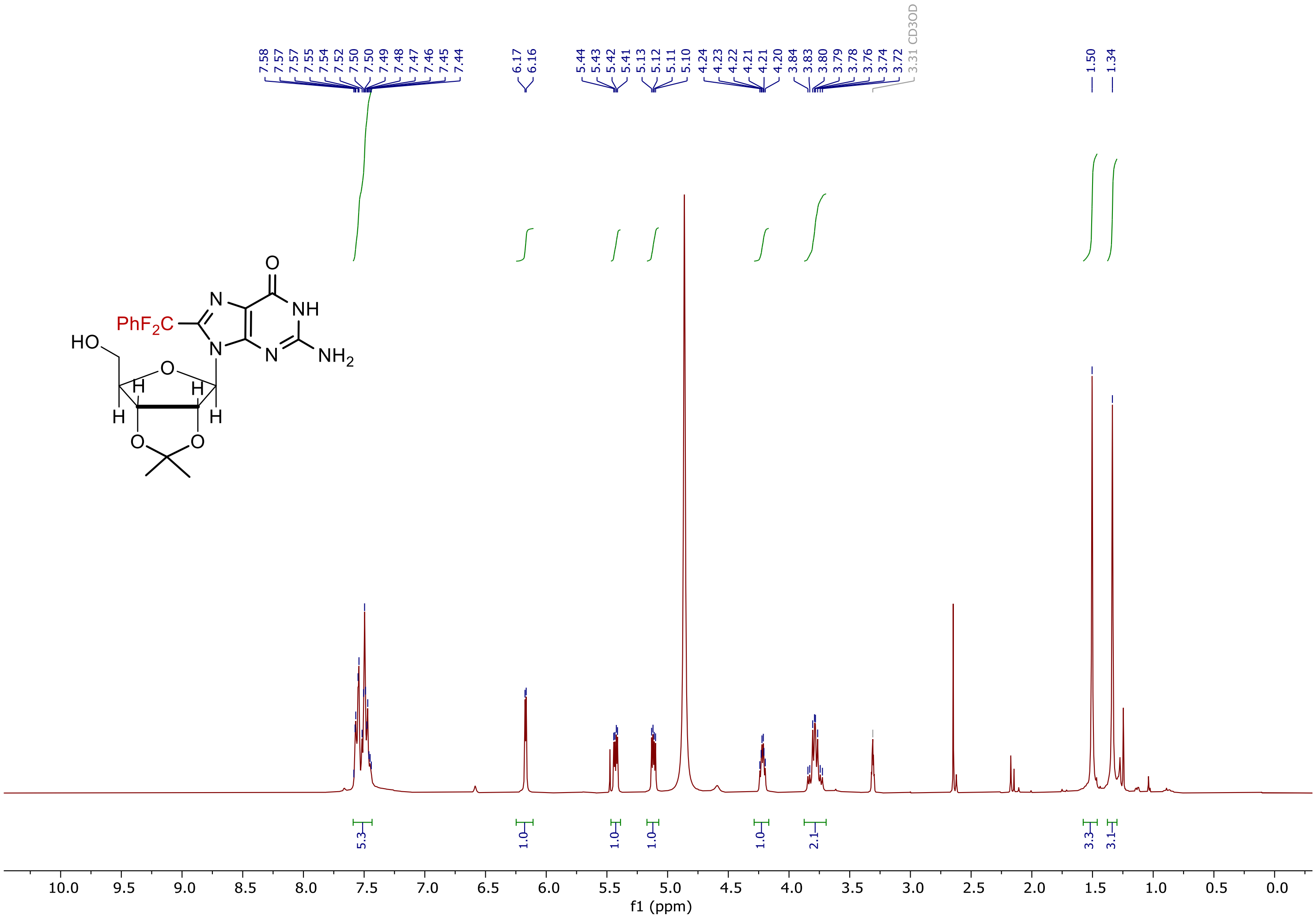

$^{13}C$ NMR (MeOD-d$_4$, 75 MHz)

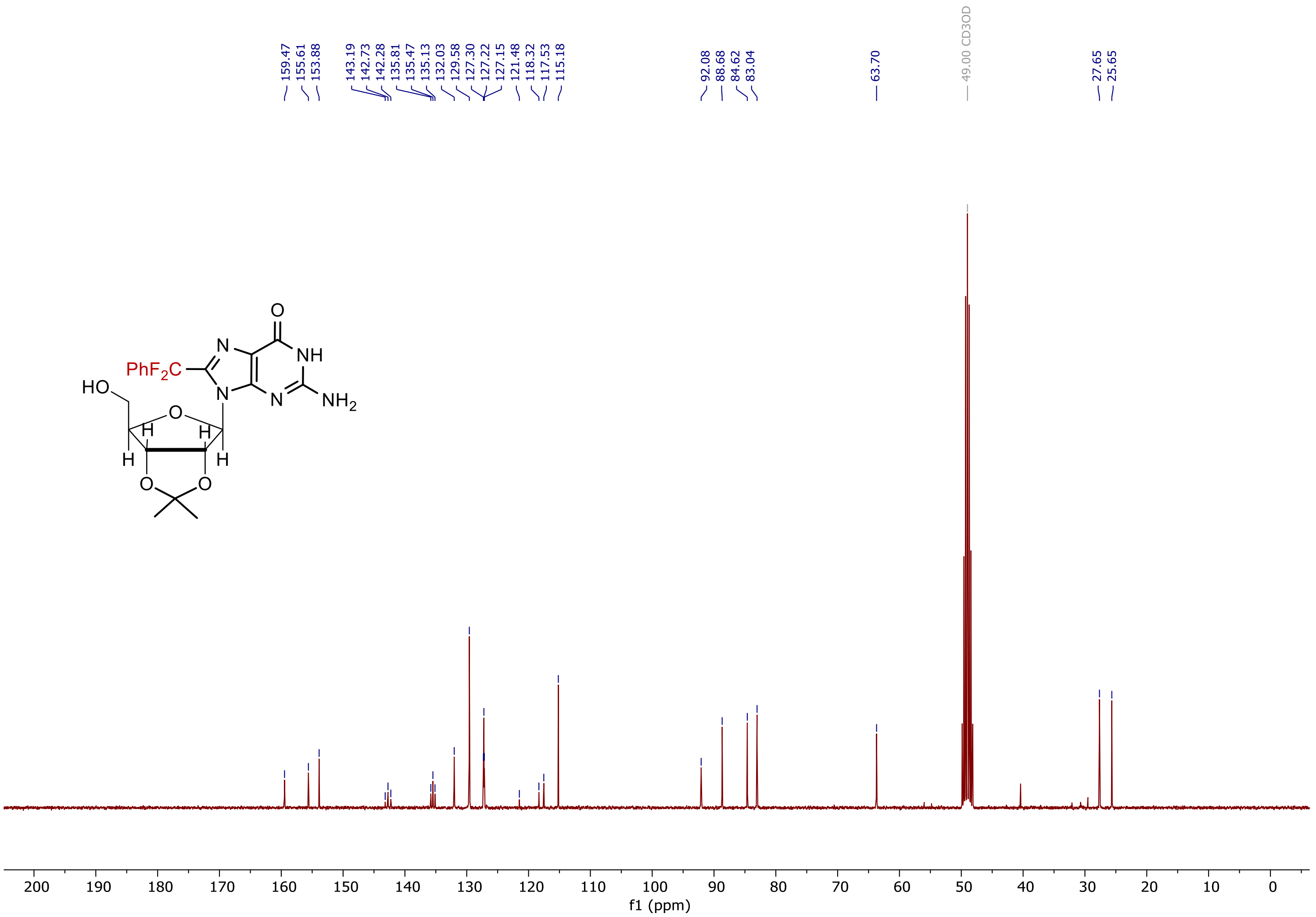

$^{19}$F NMR (MeOD-d$_4$, 282 MHz)

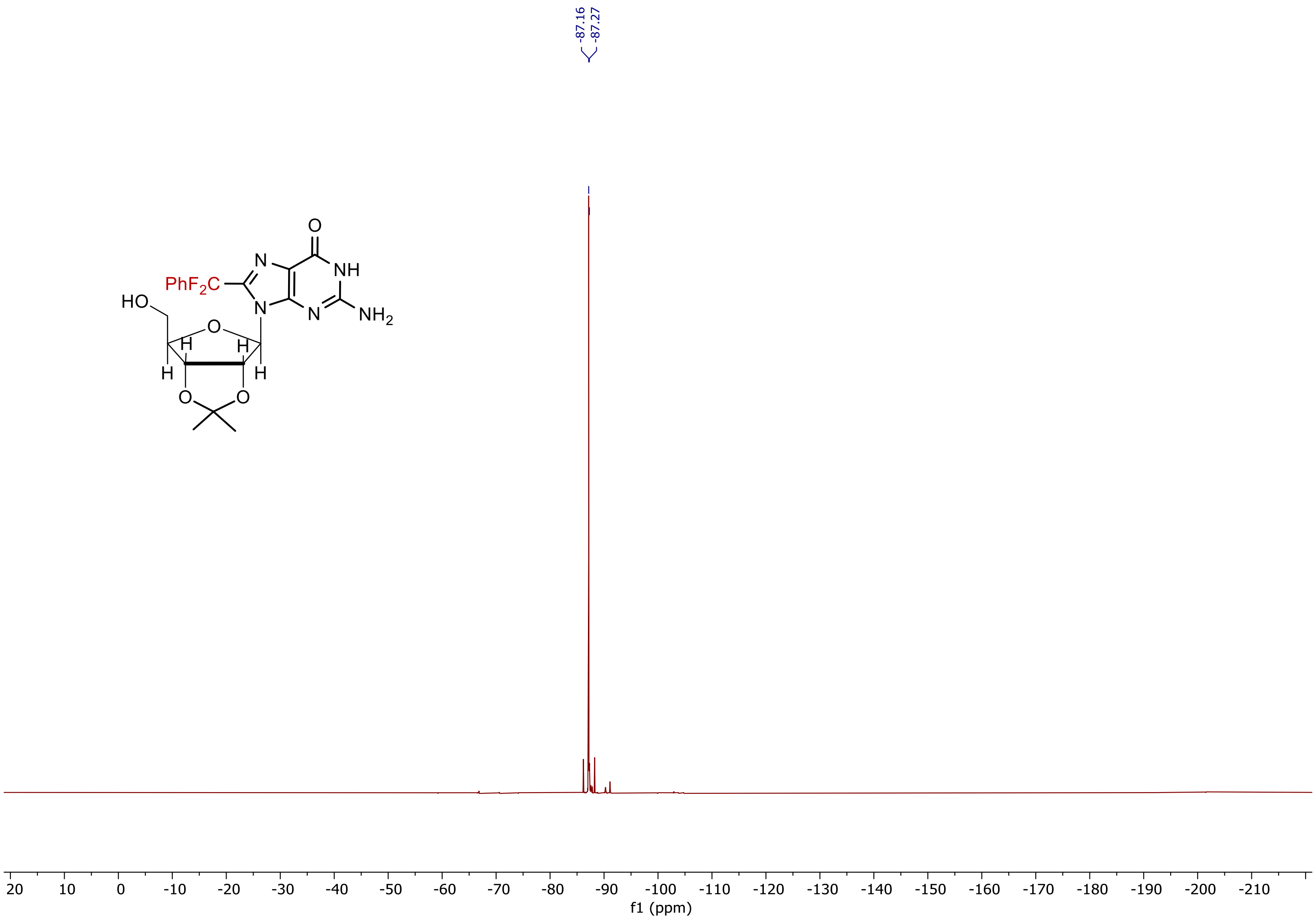

**2-Amino-8-(difluoro(phenyl)methyl)-9-((2-hydroxyethoxy)methyl)-1,4,5,9-tetrahydro-6*H*-purin-6-one 38**

$^1$H NMR (DMSO-d$_6$, 300 MHz)

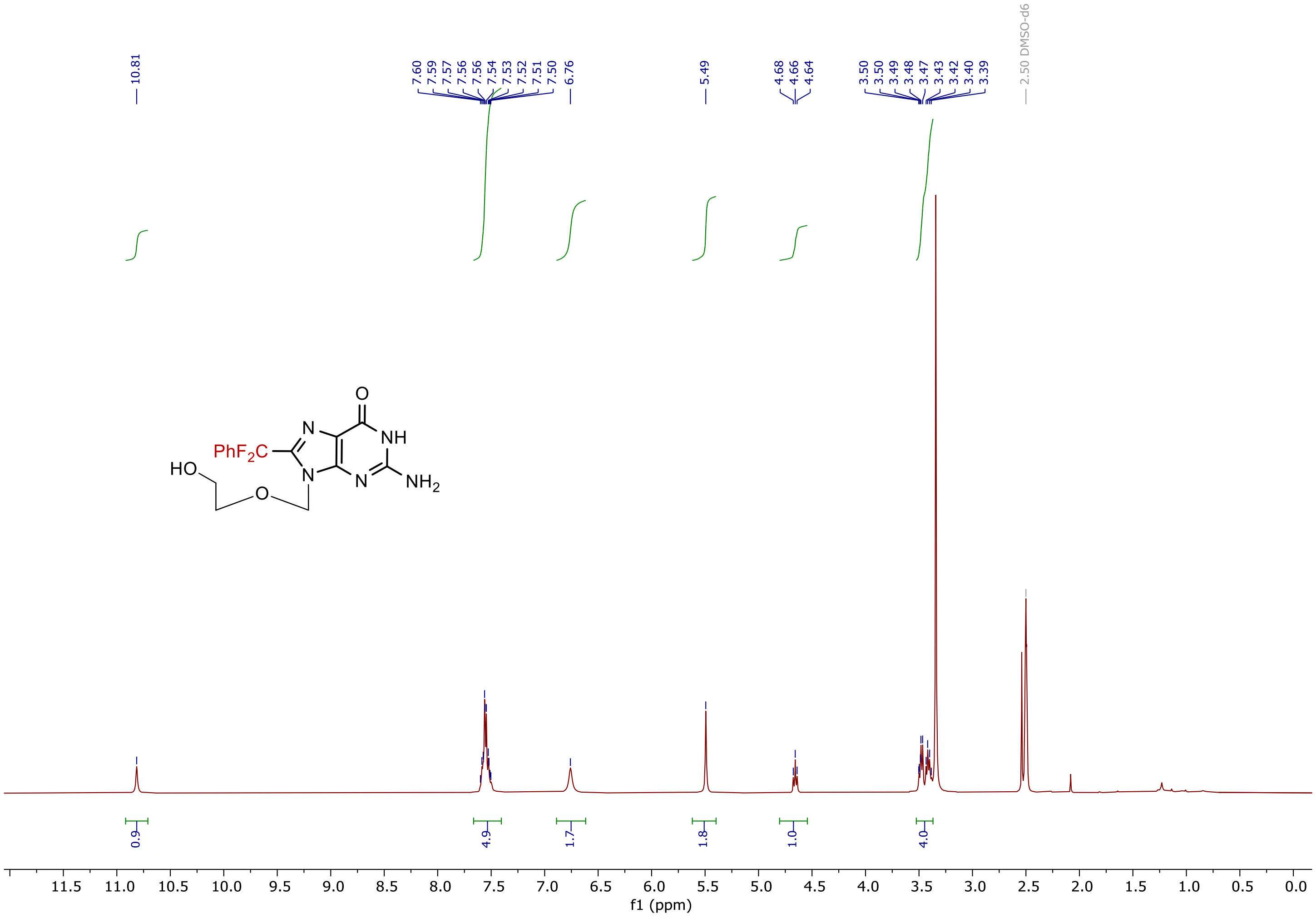

$^{13}C$ NMR (DMSO-$d_6$, 75 MHz)

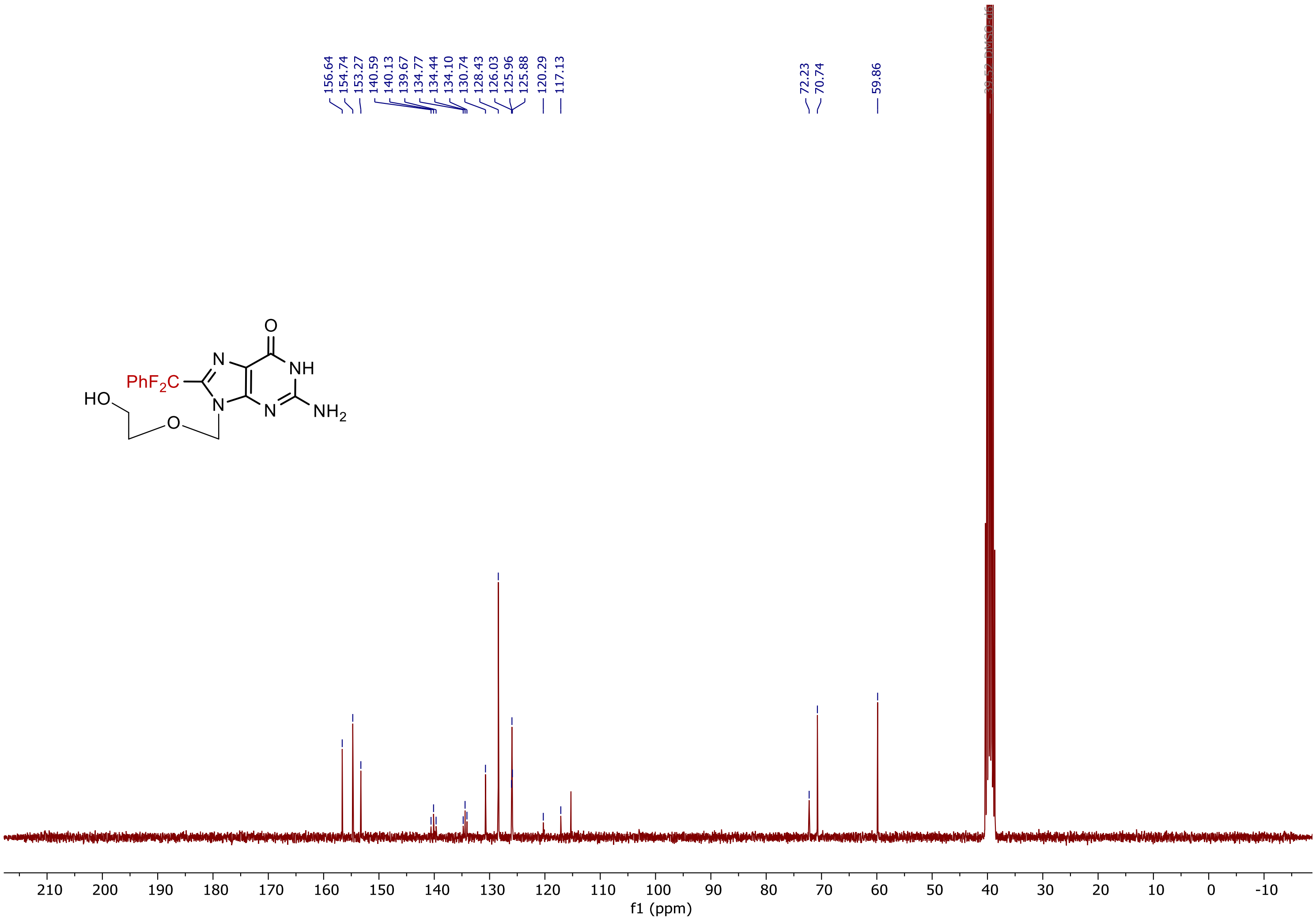

$^{19}F$ NMR (DMSO-$d_6$, 282 MHz)

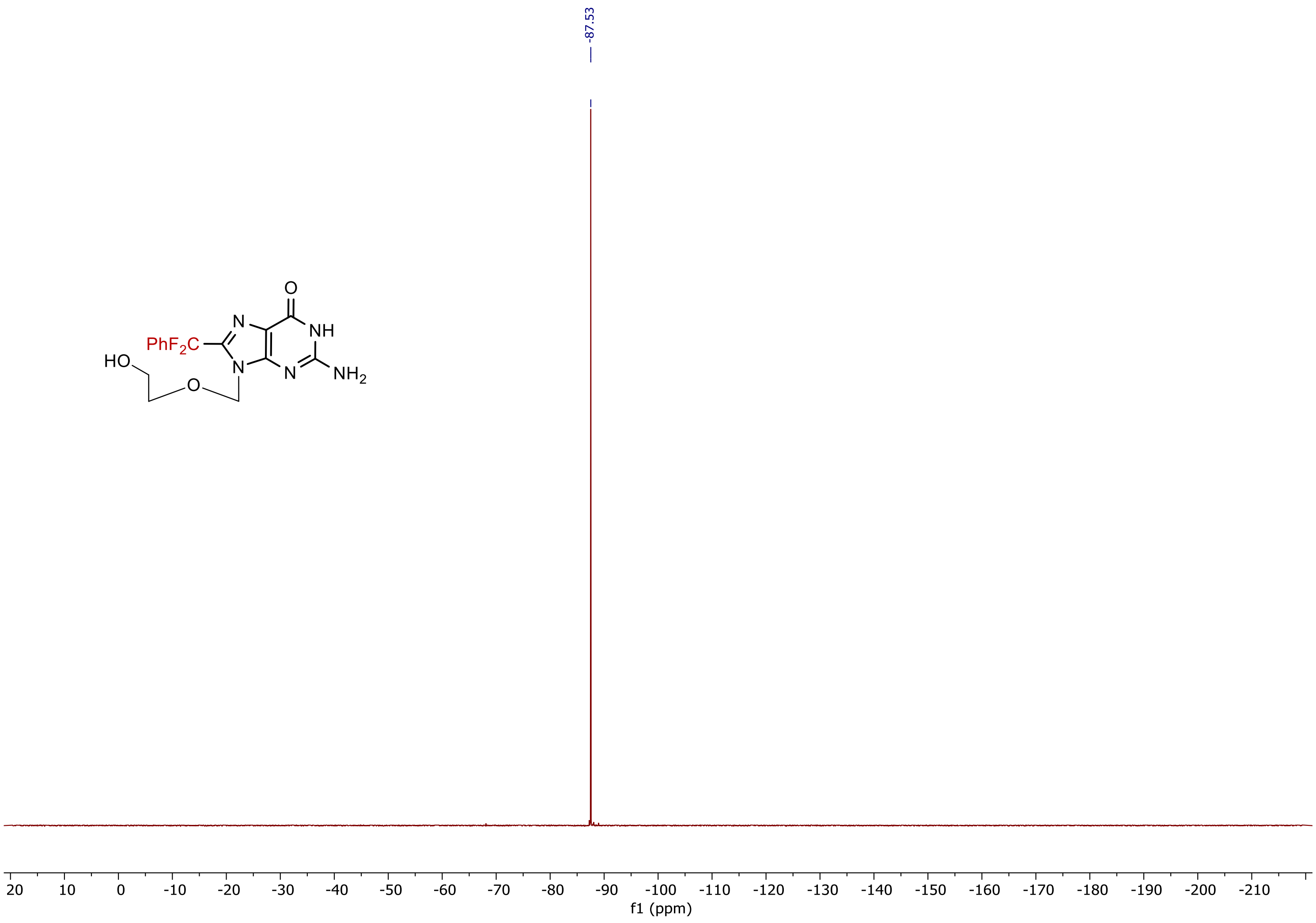

**1-((2*R*,4*S*,5*R*)-4-Hydroxy-5-(hydroxymethyl)tetrahydrofuran-2-yl)-5-(1,1,2,2-tetrafluoroethyl)pyrimidine-2,4(1*H*,3*H*)-dione 39**

$^{1}$H NMR (300 MHz, MeOD-d$_4$)

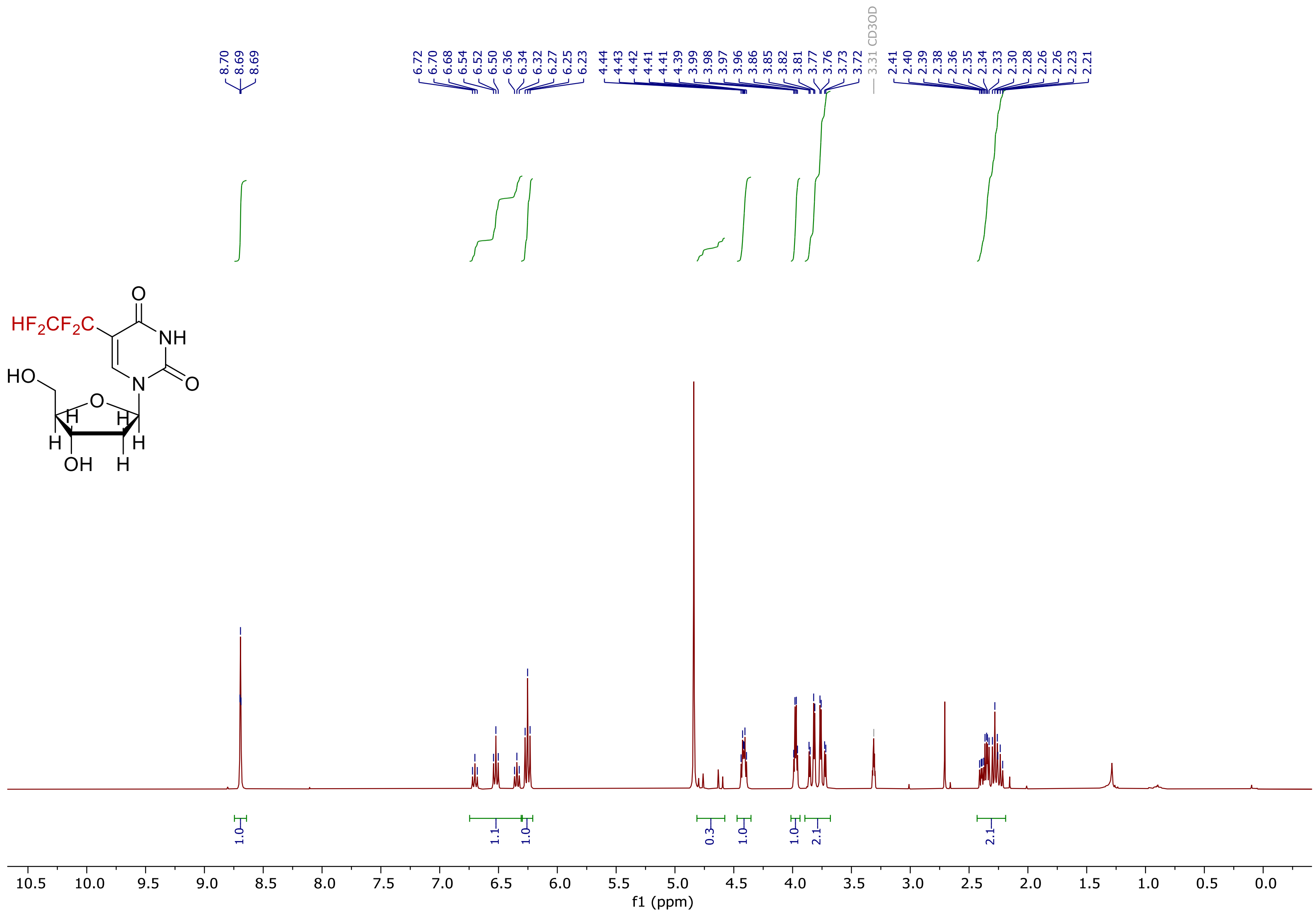

$^{13}C$ NMR (75 MHz, MeOD-d$_4$)

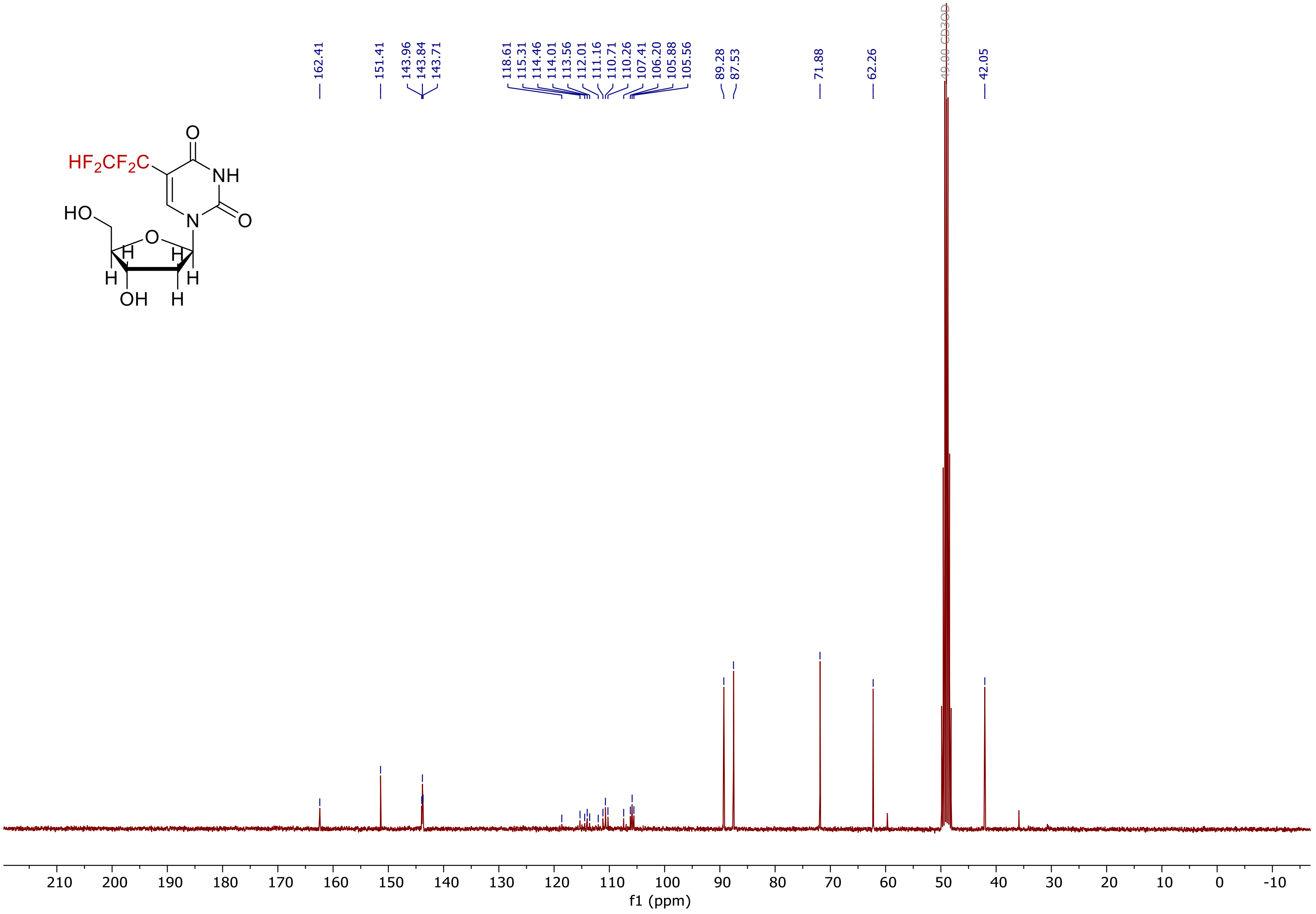

$^{19}$F NMR (282 MHz, MeOD-d$_4$)

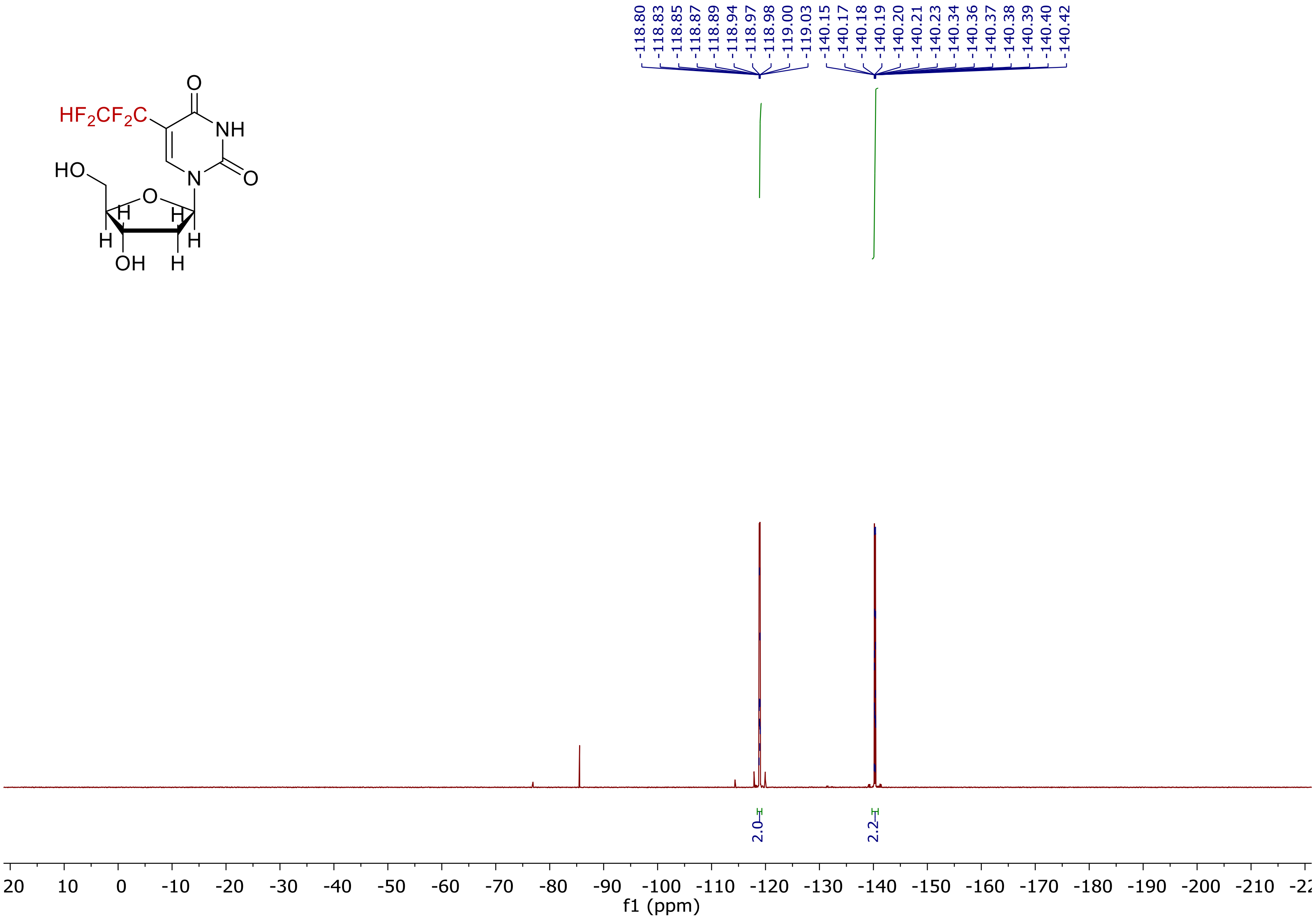

**1-((2*R*,4*S*,5*R*)-4-Hydroxy-5-(hydroxymethyl)tetrahydrofuran-2-yl)-5-(perfluoroethyl)pyrimidine-2,4(1*H*,3*H*)-dione 40**

$^{1}$H NMR (300 MHz, $CD_3CN$)

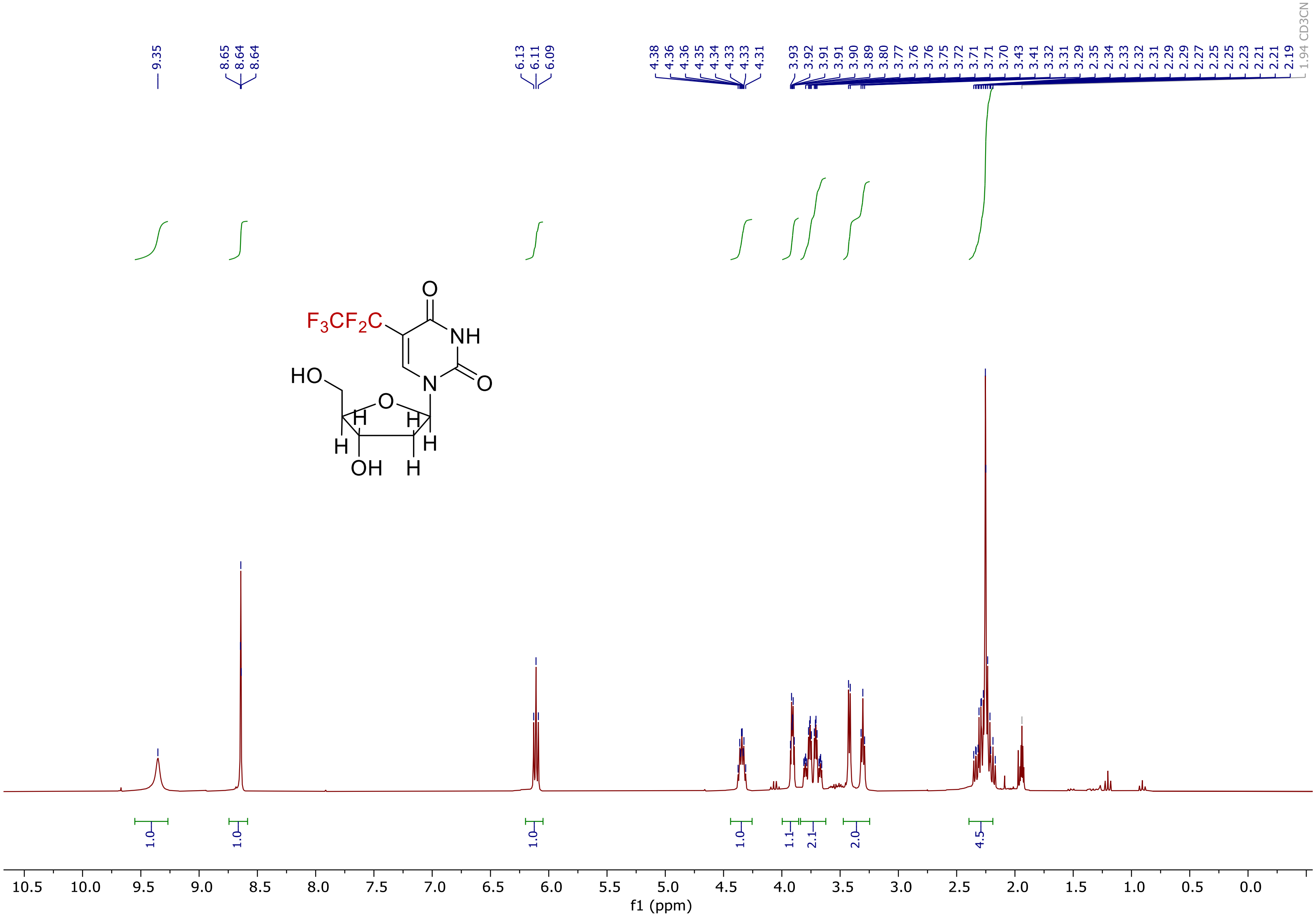

$^{13}C$ NMR (75 MHz, $CD_3CN$)

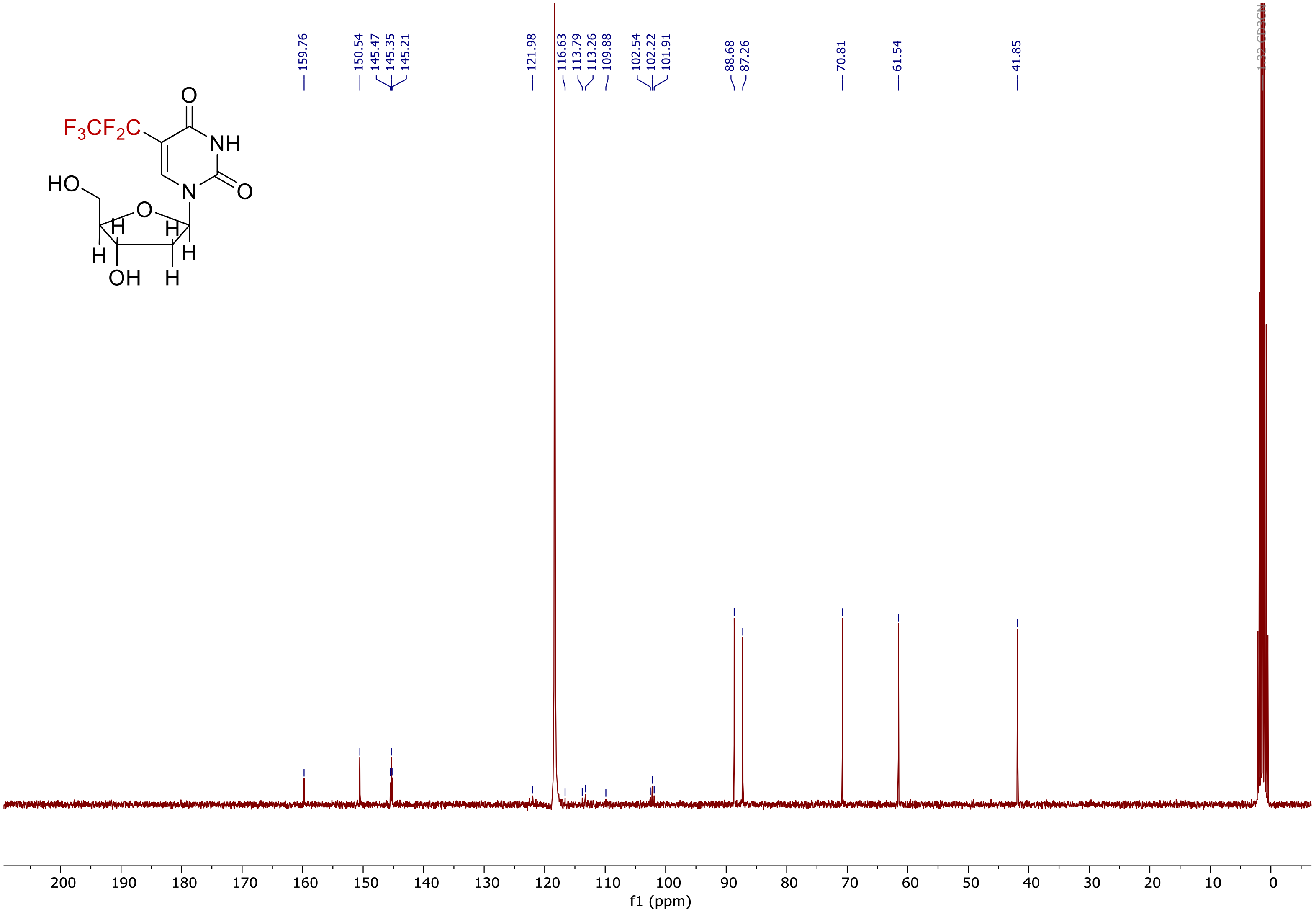

$^{19}$F NMR (282 MHz, $CD_3CN$)

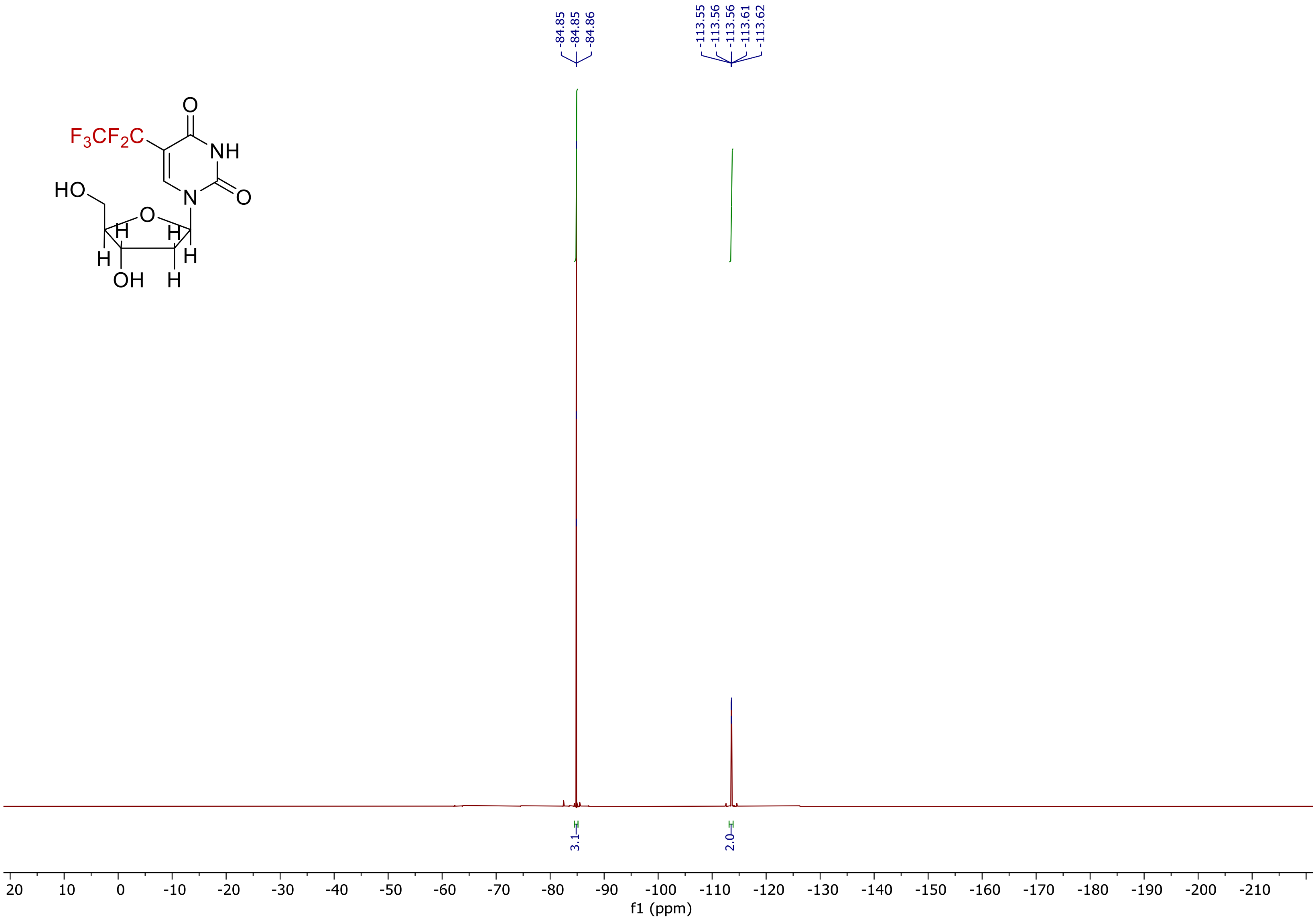

**1-((2*R*,3*R*,4*S*,5*R*)-3,4-Dihydroxy-5-(hydroxymethyl)tetrahydrofuran-2-yl)-5-(1,1,2,2-tetrafluoroethyl)pyrimidine-2,4(1*H*,3*H*)-dione 41**

$^{1}$H NMR (300 MHz, MeOD-d$_4$)

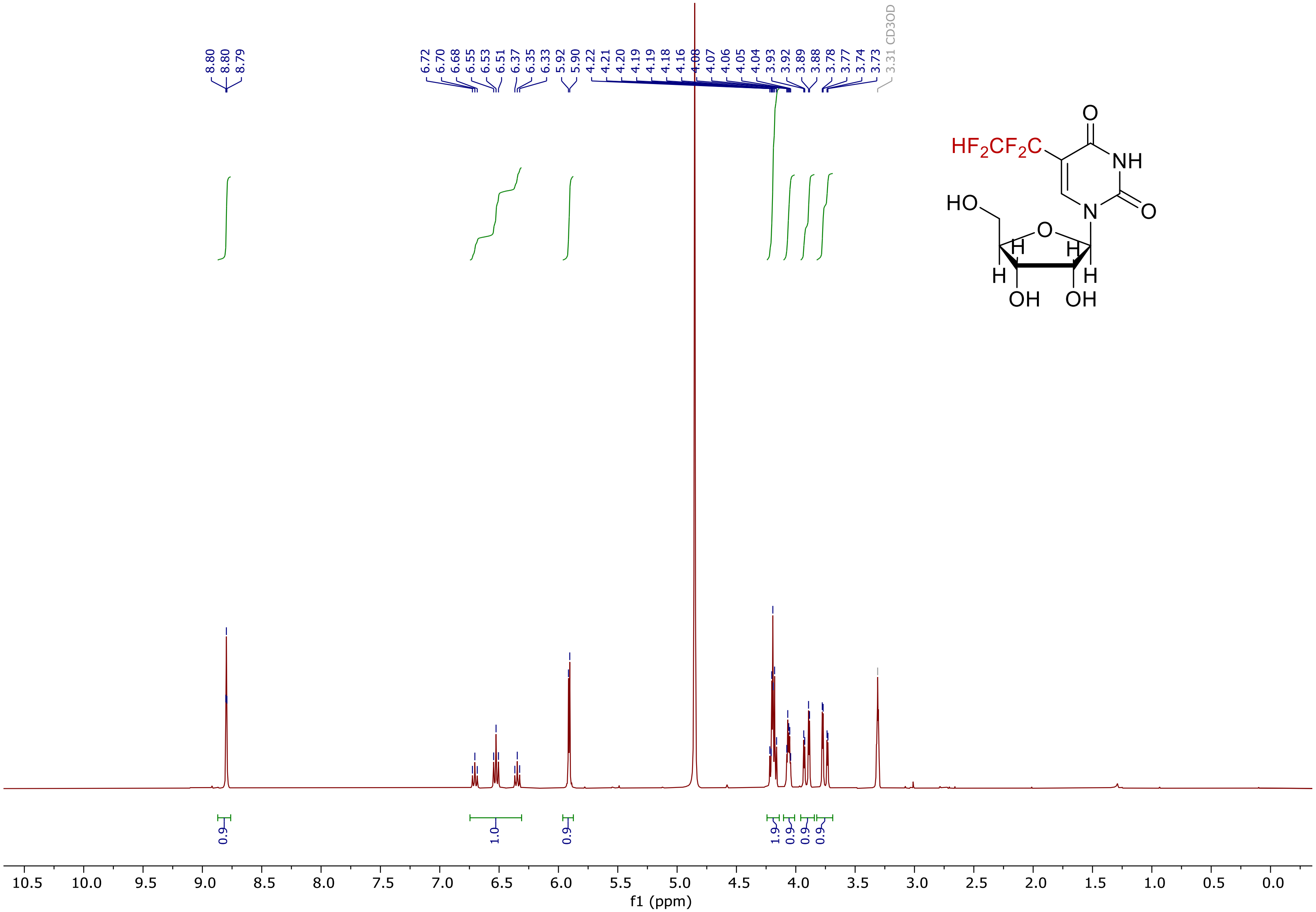

$^{13}C$ NMR (75 MHz, $CD_3CN$)

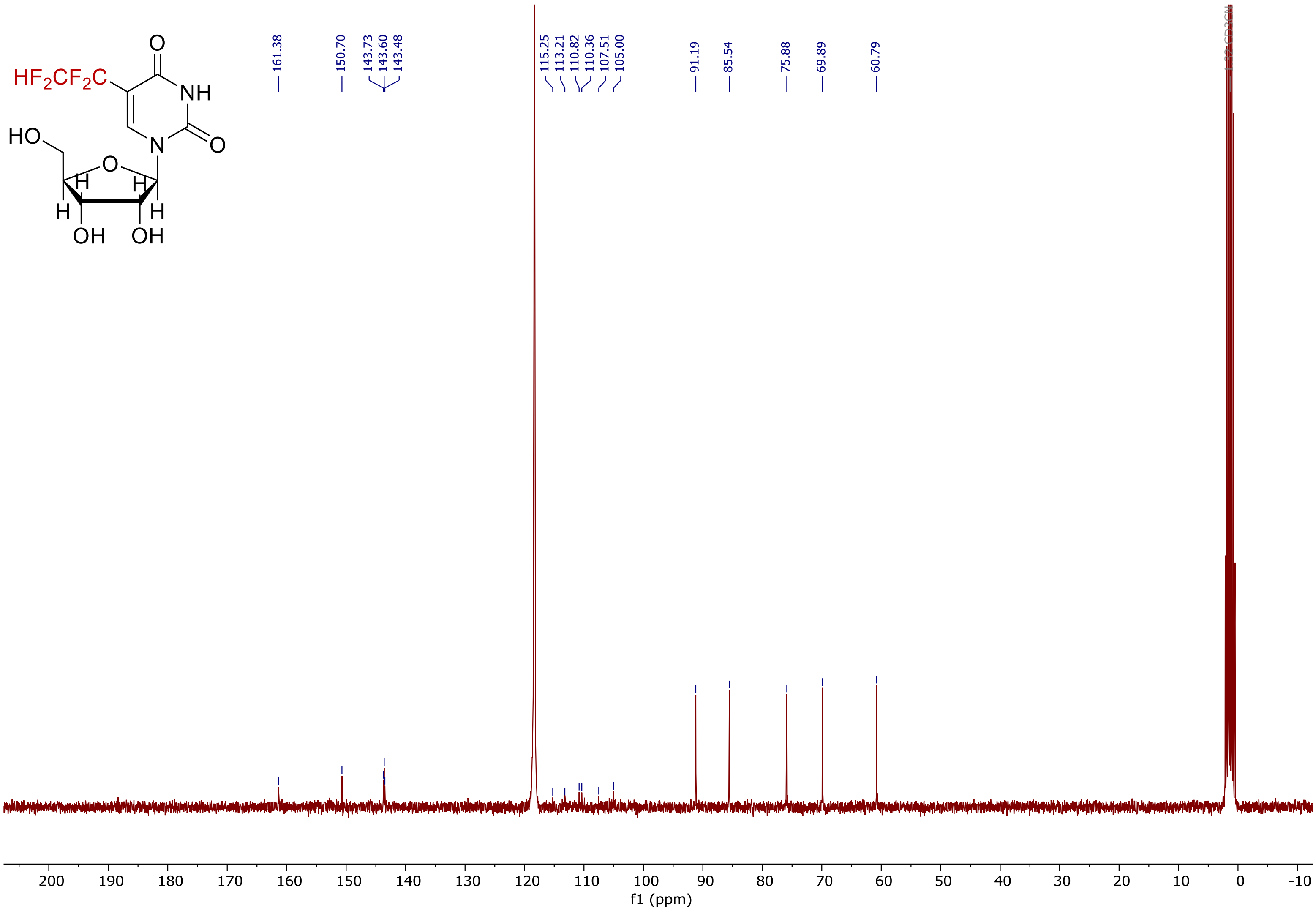

$^{19}$F NMR (282 MHz, MeOH-d$_4$)

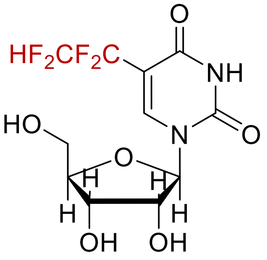


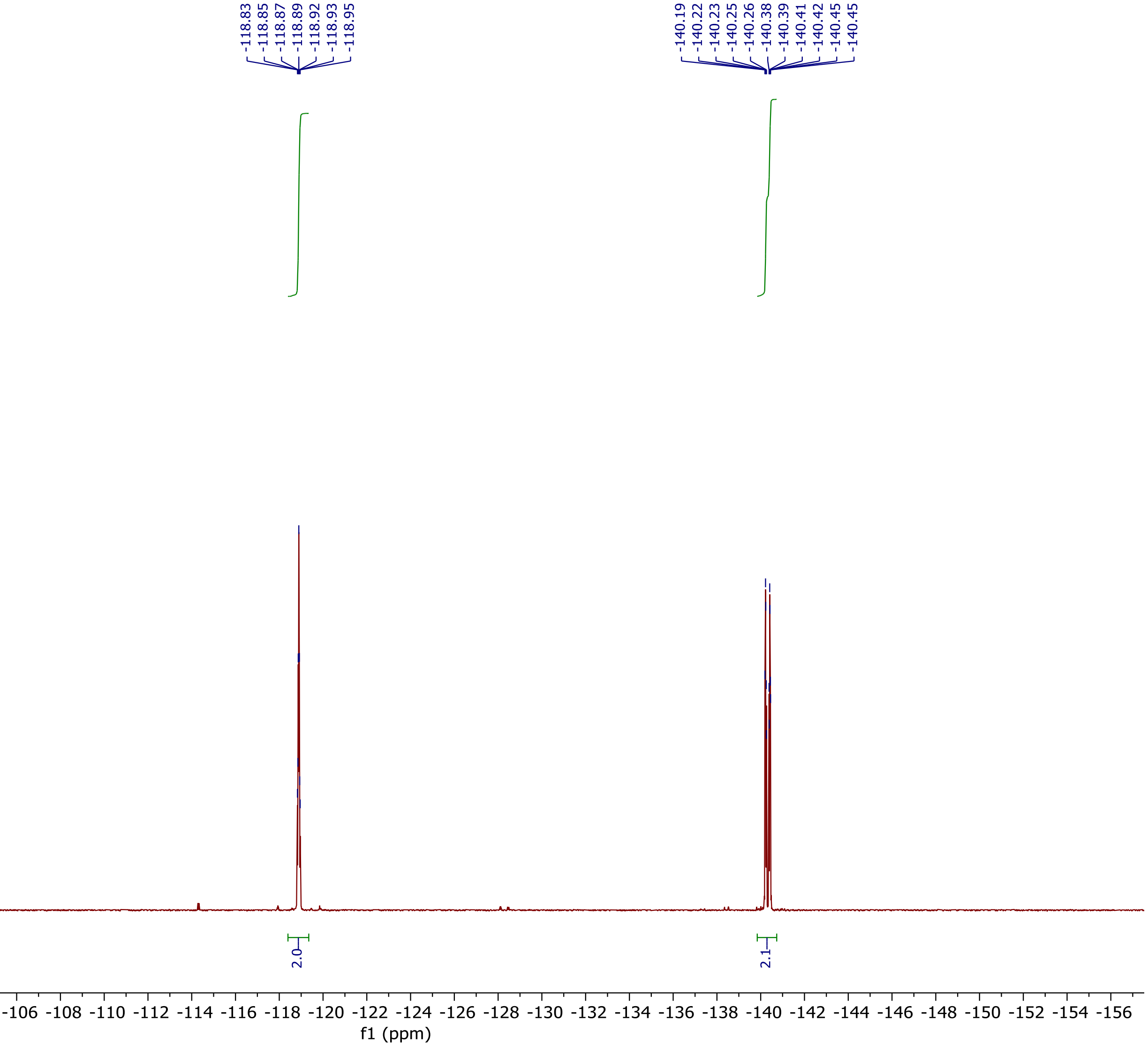

**2-Amino-8-(difluoro(phenyl)methyl)-9-((2R,3R,4S,5R)-3,4-dihydroxy-5-(hydroxymethyl)tetrahydrofuran-2-yl)-1,4,5,9-tetrahydro-6*H*-purin-6-one 42**

$^{1}H$ NMR (DMSO-$d_6$, 300 MHz)

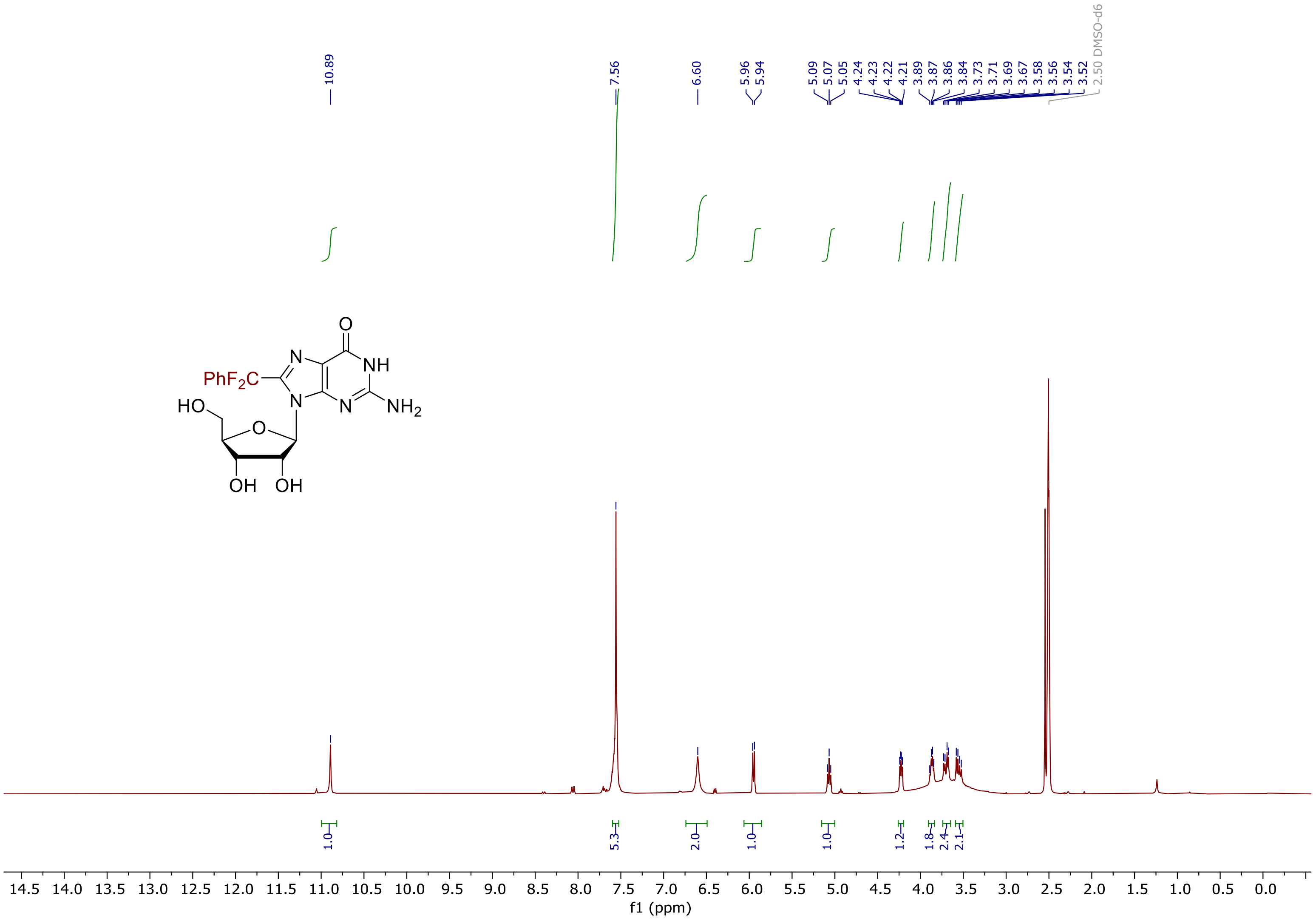

$^{13}C$ NMR (DMSO-$d_6$, 75 MHz)

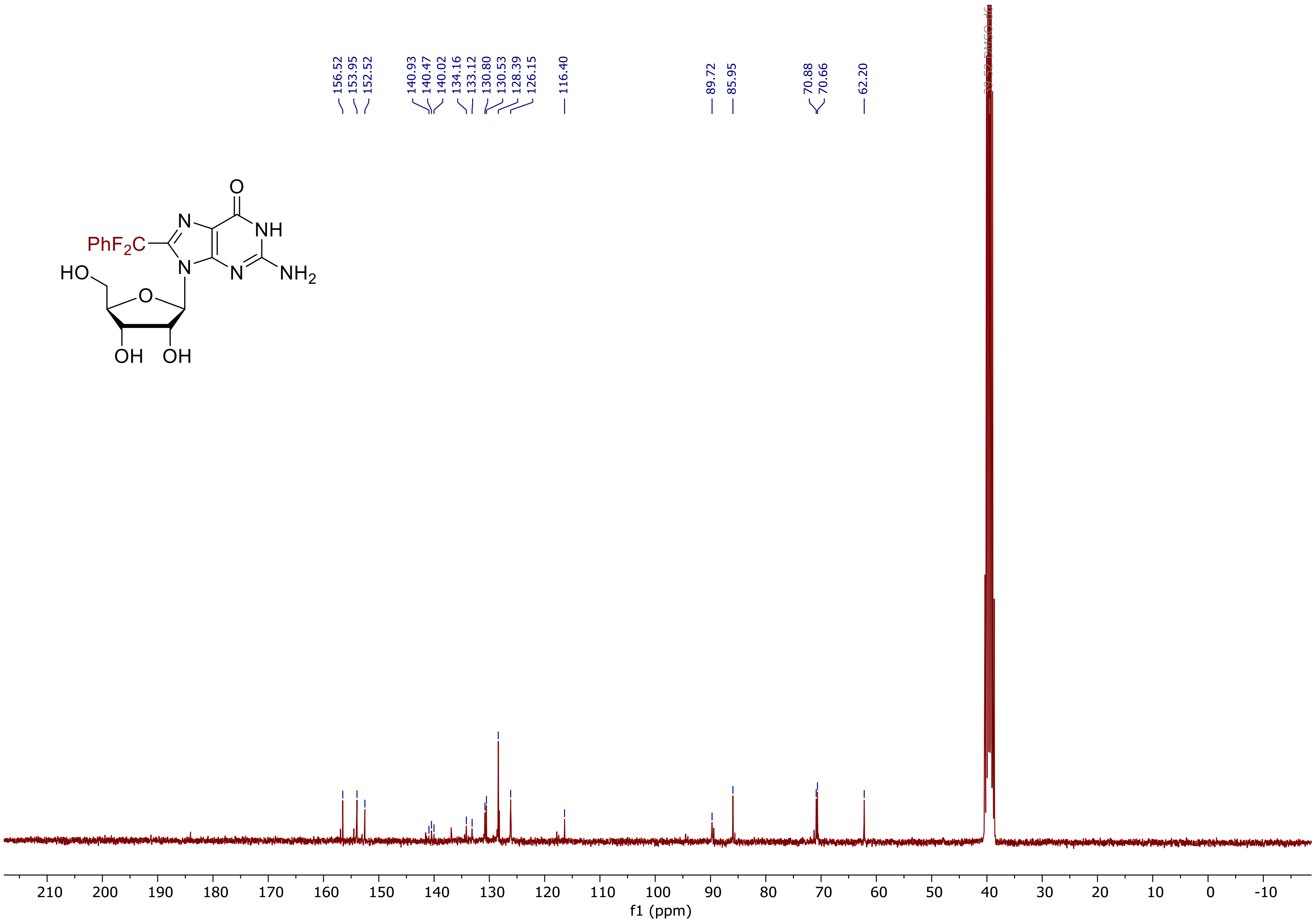

$^{19}F$ NMR (DMSO-$d_6$, 282 MHz)

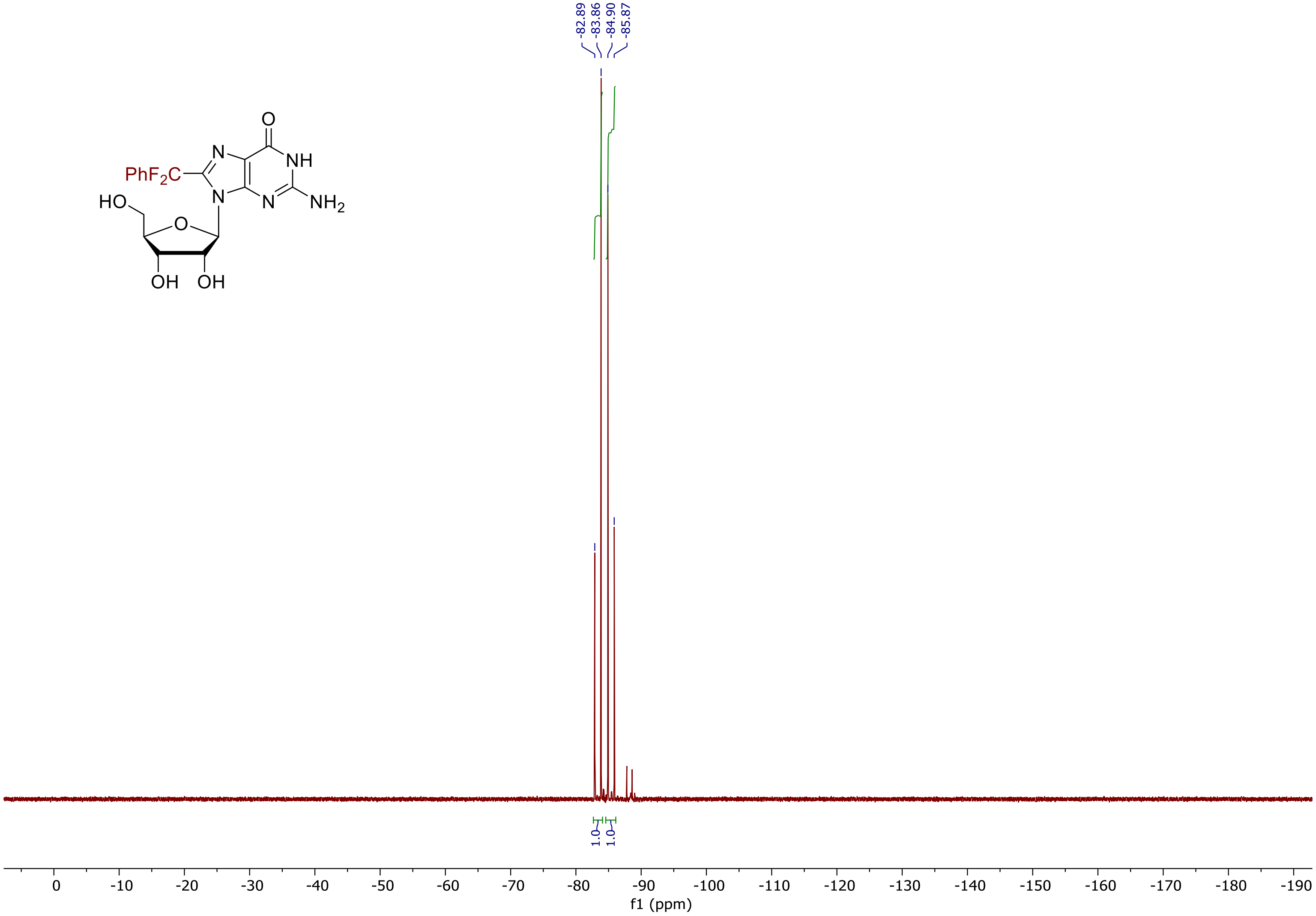

**((2*R*,3*S*,4*R*,5*R*)-5-(2,4-Dioxo-5-(1,1,2,2-tetrafluoroethyl)-3,4-dihydropyrimidin-1(2*H*)-yl)-3,4-dihydroxytetrahydrofuran-2-yl) methyl [*dihydrogen/triethylammonium*] phosphate 43**

$^{1}$H NMR (300 MHz, $D_2O$ + 1 drop DMSO)

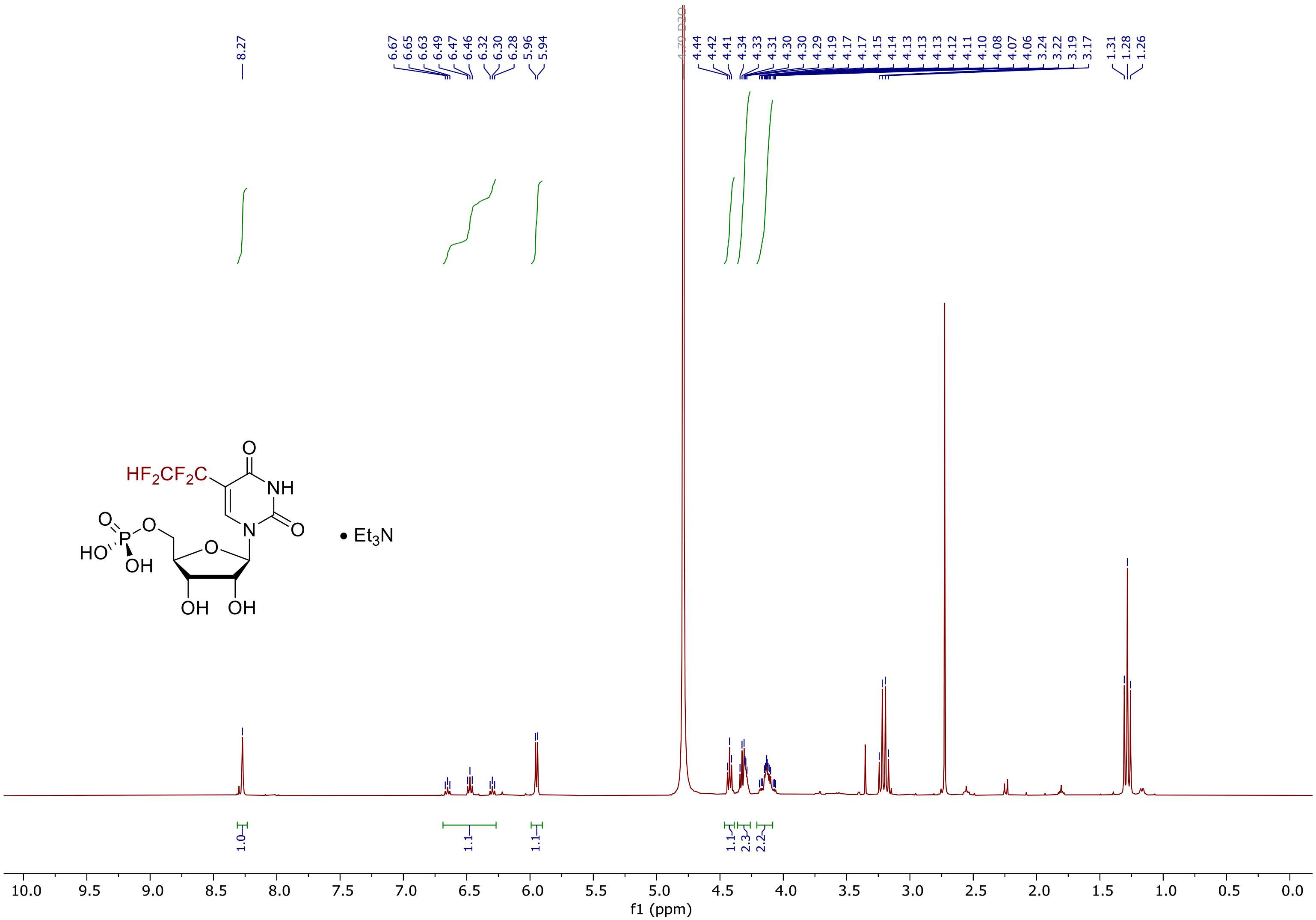

$^{13}C$ NMR (101 MHz, $D_2O$ + 1 drop DMSO)

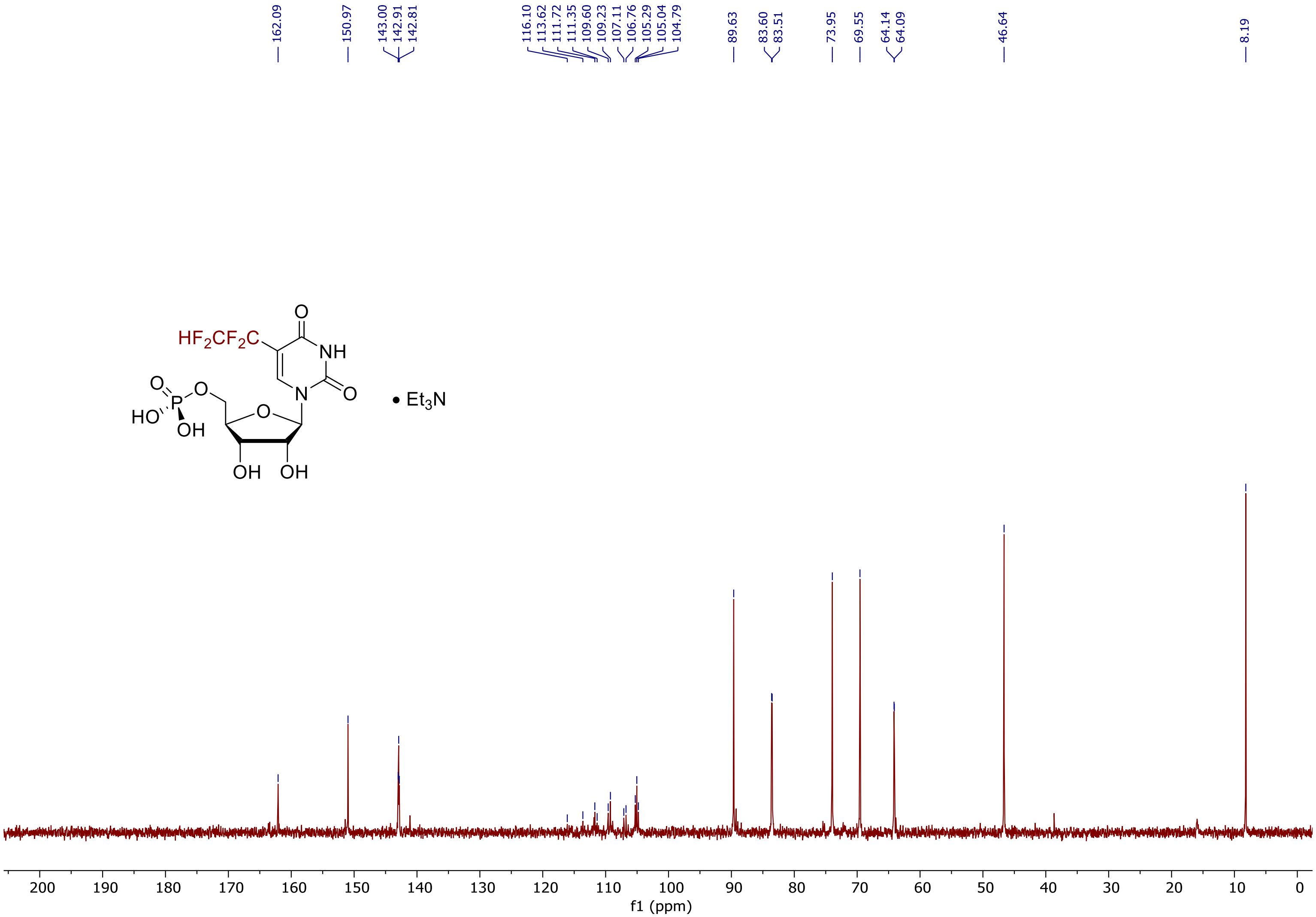

$^{19}$F NMR (282 MHz, $D_2O$)

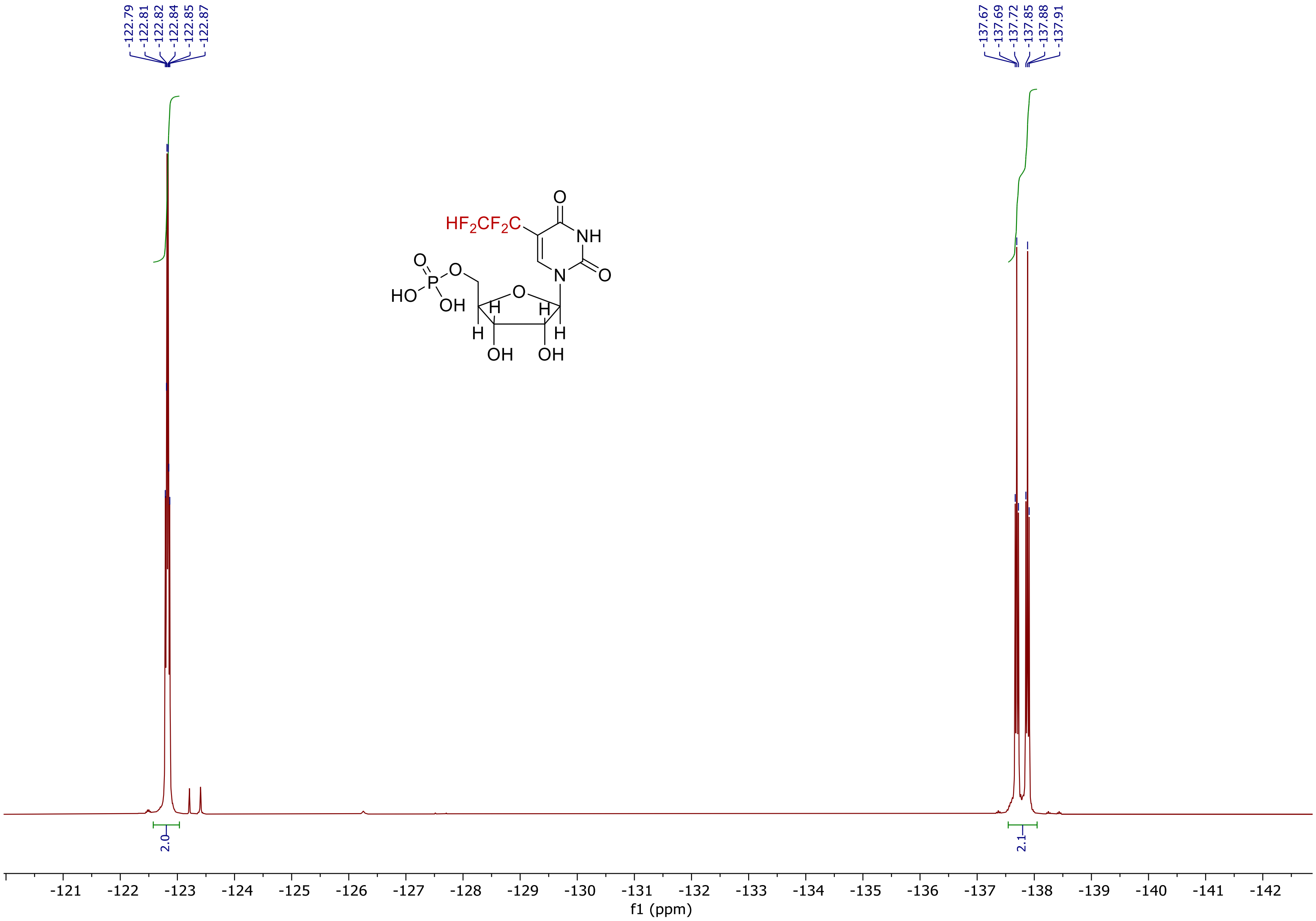

$^{31}P$ NMR (122 MHz, $D_2O$)

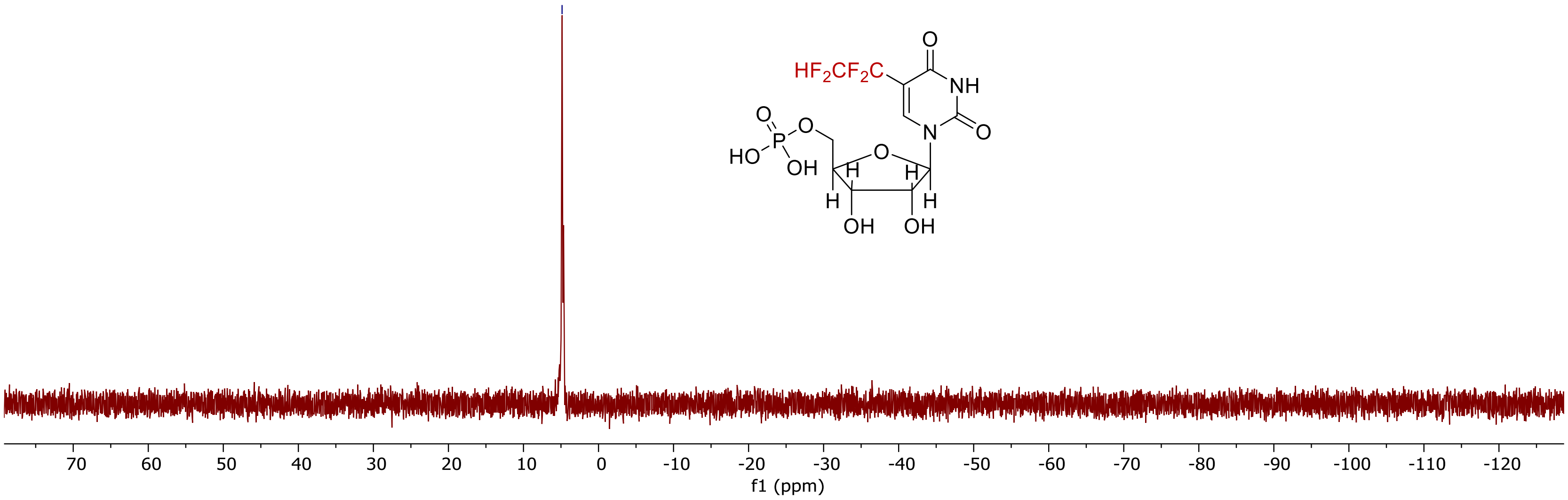

**1,1,2,2-tetrafluoro-1,2-diphenylethane 3’**

$^{1}$H NMR (300 MHz, $CDCl_3$)

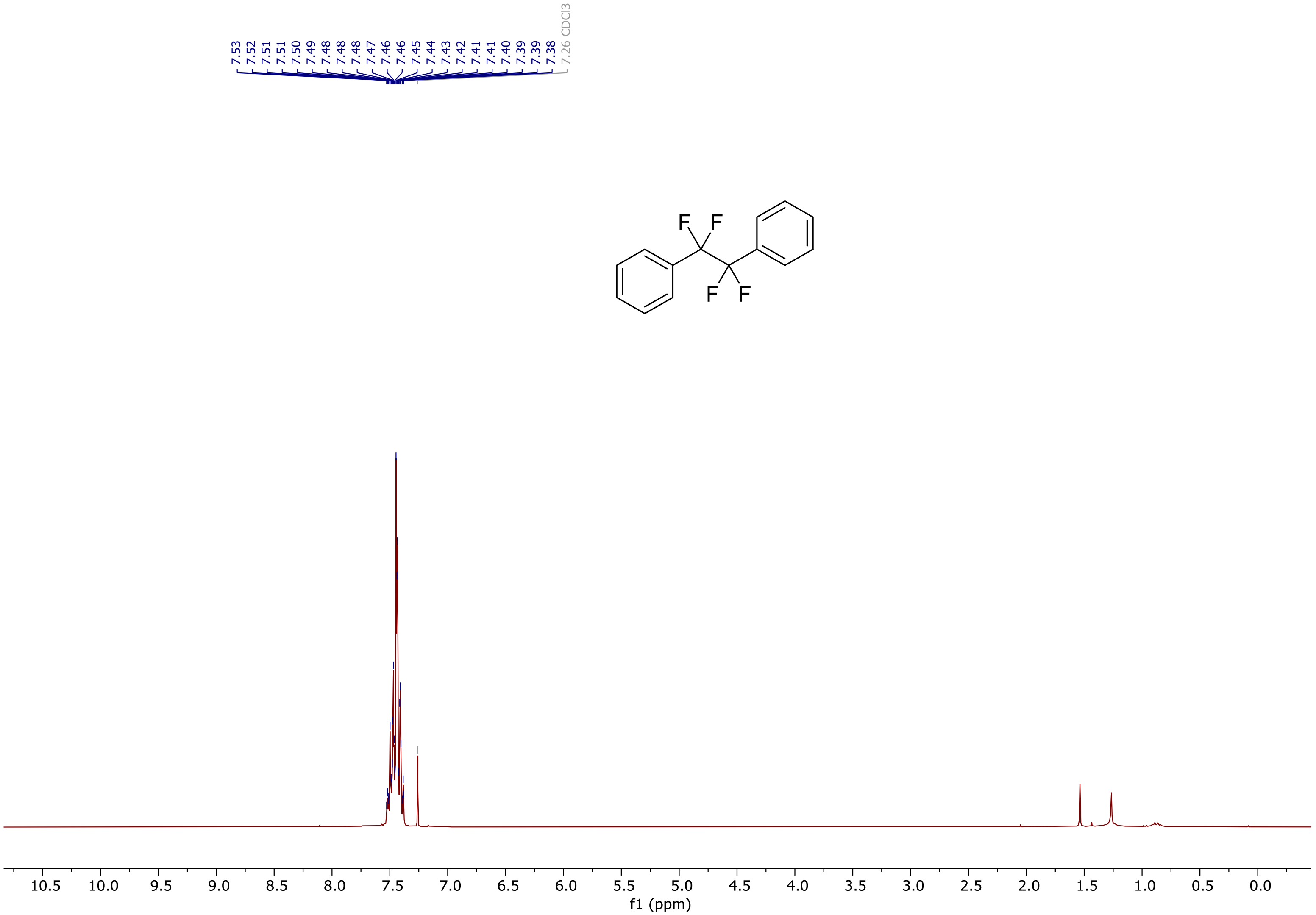

$^{13}C$ NMR ($CDCl_3$, 75 MHz)

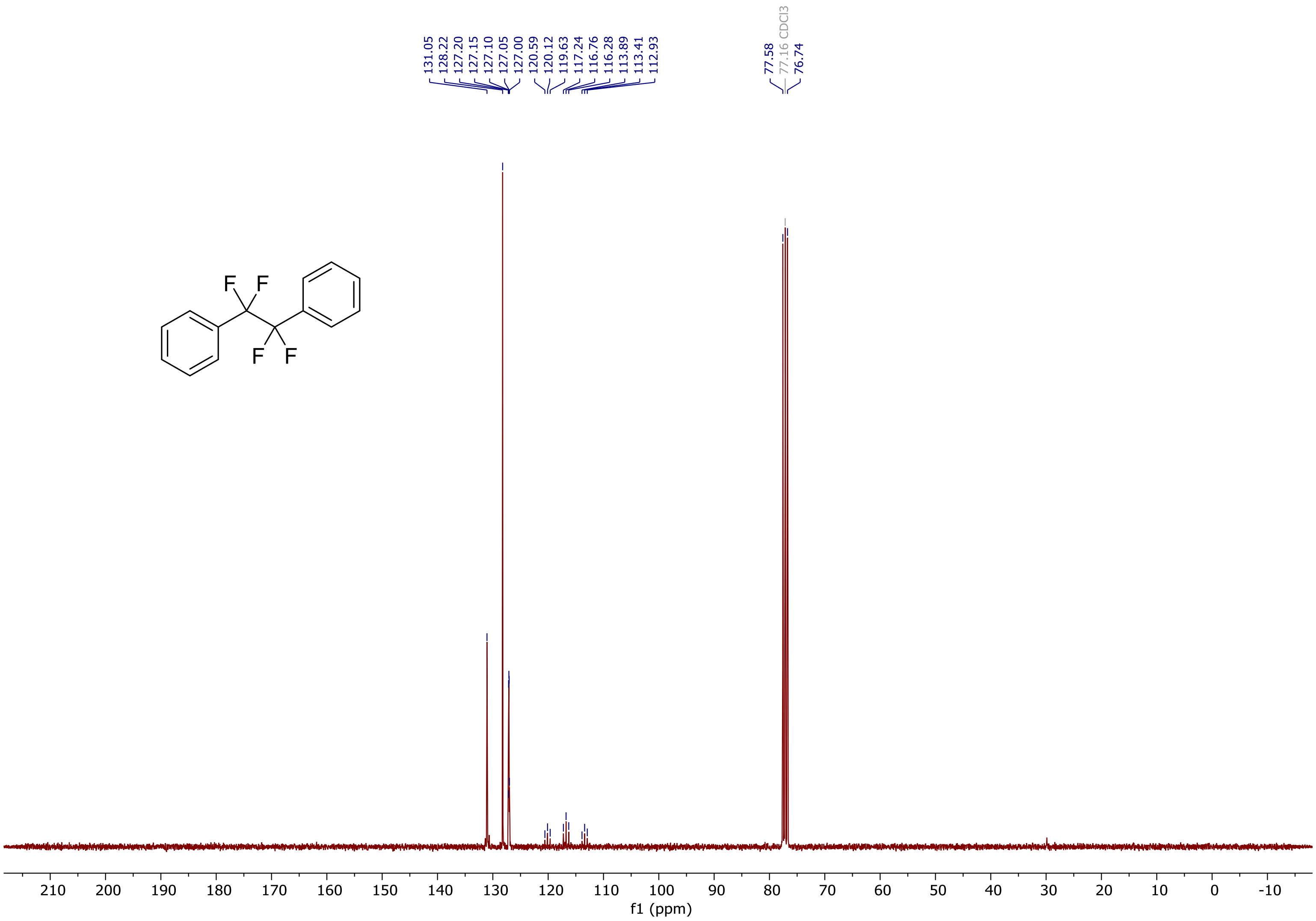

$^{19}F$ NMR (282 MHz, $CDCl_3$)

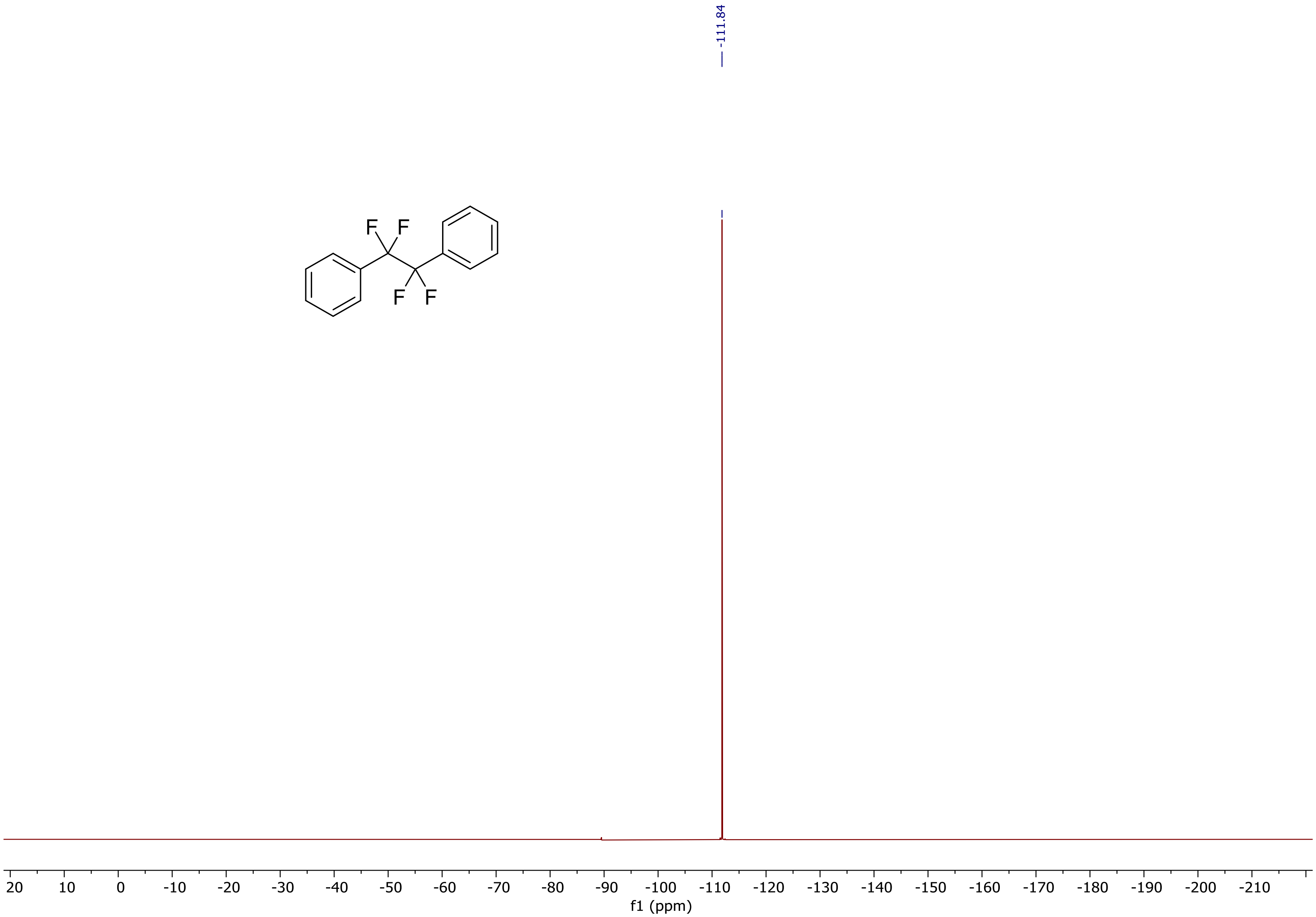

Supplement: Supplementary file 1 — Supporting Information [file ANIE-64-e202504143-s002.pdf]
